# Supplementary material for: The cost-effectiveness of preventing, diagnosing, and treating postpartum haemorrhage: A systematic review of economic evaluations
Source: PLoS Med. 2024 Sep 13;21(9):e1004461. doi: 10.1371/journal.pmed.1004461 (PMC11433145; doi:10.1371/journal.pmed.1004461)
Supplement: S2 Appendix — (DOCX) [file pmed.1004461.s002.docx]

**S2 Appendix: Search Strategies**

**Medline Search Strategy**

First Search: Ovid MEDLINE(R) <1946 to June Week 2 2023> on 22^nd^ June 2023 
Final Search: Ovid MEDLINE(R) <1946 to June Week 4 2024> on 11^th^ July 2024

| 1. | Postpartum Hemorrhage/ |
| --- | --- |
| 2. | ((postpartum or post-partum or postnatal) adj5 (h?em?or*ag* or bleed* or blood)).mp. |
| 3. | ((postpartal or post-partal or puerperal) adj3 (h?em?or*ag* or bleed* or blood)).mp. |
| 4. | ((obstetric or vaginal or c?esarian) adj delivery adj8 ((severe or severity or massive* or excessive* or extensive* or large scale or acute or unrelenting or overwhelming or uncontrolled or extreme or significant or heavy or heavier or debilitat* or prolonged or excess or dysfunctional or abnormal or abundant or profuse) adj3 (bleed* or blood loss* or blood flow* or h?em?or*ag*))).mp. |
| 5. | ((postbirth or post-birth or post-childbirth or post-child-birth or postlabo?r or post-labo?r or postdelivery or postdelivery) adj8 (h?em?or*ag* or bleed* or blood loss*)).mp. |
| 6. | ((after giving birth or after childbirth or after child-birth) adj8 (h?em?or*ag* or bleed* or blood loss*)).mp. |
| 7. | (following adj2 (birth or childbirth) adj8 ((severe or severity or massive* or excessive* or extensive* or large scale or acute or unrelenting or overwhelming or uncontrolled or extreme or significant or heavy or heavier or debilitat* or prolonged or excess or dysfunctional or abnormal or abundant or profuse) adj3 (bleed* or blood loss* or blood flow* or h?em?or*ag*))).mp. |
| 8. | 1 or 2 or 3 or 4 or 5 or 6 or 7 |
| 9. | "costs and cost analysis"/ or "cost allocation"/ or cost-benefit analysis/ or "cost control"/ or "cost savings"/ or cost-effectiveness analysis/ or economics, hospital/ or economics, medical/ or economics, nursing/ or economics, pharmaceutical/ |
| 10. | Health Care Rationing/ |
| 11. | Health Care Costs/ |
| 12. | "Drug Utilization Review"/ |
| 13. | "cost of illness"/ |
| 14. | (cost-effective* or cost-benefit* or benefit-cost? or economic evaluation* or pharmacoeconomic* or pharmaco-economic* or cost analys?s).mp. |
| 15. | ((economic* or cost or costs or costly or costing or price or prices or pricing) adj4 (evaluat* or analys#s or measure* or comparison*)).mp. |
| 16. | (value adj2 money).mp. |
| 17. | (cost audit* or cost containment or cost saving*).mp. |
| 18. | (cost* adj3 (audit* or containment or saving* or expenditure* or evaluat* or analys#s or evaluat* or analys#s or measure* or comparison*)).mp. |
| 19. | (cost* utility or marginal analys#s or affordabilit* or cost minimi#ation or cost-consequence* or cost efficienc*).mp. |
| 20. | ((financ* or money or monies or monetary or budget* or fiscal) adj4 (expenditure* or evaluat* or analys#s or measure* or comparison* or impact?)).mp. |
| 21. | (risk-benefit or "return on investment" or "willing* to pay").mp. |
| 22. | disability-adjusted life years/ or quality-adjusted life years/ |
| 23. | (QALY or DALY).mp. |
| 24. | ("disability-adjusted life year*" or "quality-adjusted life year*").mp. |
| 25. | "cost* and benefit*".mp. |
| 26. | 9 or 10 or 11 or 12 or 13 or 14 or 15 or 16 or 17 or 18 or 19 or 20 or 21 or 22 or 23 or 24 or 25 |
| 27. | 8 and 26 |
| 28. | Postpartum Hemorrhage/ec [Economics] |
| 29. | 27 or 28 |

**Embase Search Strategy**

First Search: Embase <1980 to 2023 Week 24> on 22^nd^ June 2023 
Final Search: Embase <1996 to 2024 Week 27> on 11^th^ July 2024

| 1. | postpartum hemorrhage/ |
| --- | --- |
| 2. | ((postpartum or post-partum or postnatal) adj5 (h?em?or*ag* or bleed* or blood)).mp. |
| 3. | ((postpartal or post-partal or puerperal) adj3 (h?em?or*ag* or bleed* or blood)).mp. |
| 4. | ((obstetric or vaginal or c?esarian) adj delivery adj8 ((severe or severity or massive* or excessive* or extensive* or large scale or acute or unrelenting or overwhelming or uncontrolled or extreme or significant or heavy or heavier or debilitat* or prolonged or excess or dysfunctional or abnormal or abundant or profuse) adj3 (bleed* or blood loss* or blood flow* or h?em?or*ag*))).mp. |
| 5. | ((postbirth or post-birth or post-childbirth or post-child-birth or postlabo?r or post-labo?r or postdelivery or postdelivery) adj8 (h?em?or*ag* or bleed* or blood loss*)).mp. |
| 6. | ((after giving birth or after childbirth or after child-birth) adj8 (h?em?or*ag* or bleed* or blood loss*)).mp. |
| 7. | (following adj2 (birth or childbirth) adj8 ((severe or severity or massive* or excessive* or extensive* or large scale or acute or unrelenting or overwhelming or uncontrolled or extreme or significant or heavy or heavier or debilitat* or prolonged or excess or dysfunctional or abnormal or abundant or profuse) adj3 (bleed* or blood loss* or blood flow* or h?em?or*ag*))).mp. |
| 8. | 1 or 2 or 3 or 4 or 5 or 6 or 7 |
| 9. | (cost-effective* or cost-benefit* or benefit-cost? or economic evaluation* or pharmacoeconomic* or pharmaco-economic* or cost analys?s).mp. |
| 10. | ((economic* or cost or costs or costly or costing or price or prices or pricing) adj4 (evaluat* or analys#s or measure* or comparison*)).mp. |
| 11. | (value adj2 money).mp. |
| 12. | (cost audit* or cost containment or cost saving*).mp. |
| 13. | ((financ* or money or monies or monetary or budget*) adj4 (expenditure* or evaluat* or analys#s or measure* or comparison* or impact?)).mp. |
| 14. | (cost* adj3 (audit* or containment or saving* or expenditure* or evaluat* or analys#s or evaluat* or analys#s or measure* or comparison*)).mp. |
| 15. | (cost* utility or marginal analys#s or affordabilit* or cost minimi#ation or cost-consequence* or cost efficienc*).mp. |
| 16. | ((financ* or money or monies or monetary or budget* or fiscal) adj4 (expenditure* or evaluat* or analys#s or measure* or comparison*)).mp. |
| 17. | (risk-benefit or "return on investment" or "willing* to pay").mp. |
| 18. | disability-adjusted life years/ or quality-adjusted life years/ |
| 19. | (QALY or DALY).mp. |
| 20. | "cost* and benefit*".mp. |
| 21. | "cost effectiveness analysis"/ or "cost control"/ or "cost benefit analysis"/ or "cost minimization analysis"/ or "cost of illness"/ or "cost utility analysis"/ or health economics/ or "health care cost"/ or pharmacoeconomics/ or "drug utilization review"/ or economic evaluation/ |
| 22. | 9 or 10 or 11 or 12 or 13 or 14 or 15 or 16 or 17 or 18 or 19 or 20 or 21 |
| 23. | 8 and 22 |

**Emcare Search Strategy**

First Search: Ovid Emcare (Nursing & Allied Health) on 22^nd^ June 2023 
Final Search: Ovid Emcare (Nursing & Allied Health) on 11^th^ July 2024

| 1. | postpartum hemorrhage/ |
| --- | --- |
| 2. | ((postpartum or post-partum or postnatal) adj5 (h?em?or*ag* or bleed* or blood)).mp. |
| 3. | ((postpartal or post-partal or puerperal) adj3 (h?em?or*ag* or bleed* or blood)).mp. |
| 4. | ((obstetric or vaginal or c?esarian) adj delivery adj8 ((severe or severity or massive* or excessive* or extensive* or large scale or acute or unrelenting or overwhelming or uncontrolled or extreme or significant or heavy or heavier or debilitat* or prolonged or excess or dysfunctional or abnormal or abundant or profuse) adj3 (bleed* or blood loss* or blood flow* or h?em?or*ag*))).mp. |
| 5. | ((postbirth or post-birth or post-childbirth or post-child-birth or postlabo?r or post-labo?r or postdelivery or postdelivery) adj8 (h?em?or*ag* or bleed* or blood loss*)).mp. |
| 6. | ((after giving birth or after childbirth or after child-birth) adj8 (h?em?or*ag* or bleed* or blood loss*)).mp. |
| 7. | (following adj2 (birth or childbirth) adj8 ((severe or severity or massive* or excessive* or extensive* or large scale or acute or unrelenting or overwhelming or uncontrolled or extreme or significant or heavy or heavier or debilitat* or prolonged or excess or dysfunctional or abnormal or abundant or profuse) adj3 (bleed* or blood loss* or blood flow* or h?em?or*ag*))).mp. |
| 8. | 1 or 2 or 3 or 4 or 5 or 6 or 7 |
| 9. | disability-adjusted life years/ or quality-adjusted life years/ |
| 10. | "cost effectiveness analysis"/ or "cost control"/ or "cost benefit analysis"/ or "cost minimization analysis"/ or "cost of illness"/ or "cost utility analysis"/ or health economics/ or "health care cost"/ or pharmacoeconomics/ or "drug utilization review"/ or economic evaluation/ |
| 11. | (cost-effective* or cost-benefit* or benefit-cost? or economic evaluation* or pharmacoeconomic* or pharmaco-economic* or cost analys?s).mp. |
| 12. | ((economic* or cost or costs or costly or costing or price or prices or pricing) adj4 (evaluat* or analys#s or measure* or comparison*)).mp. |
| 13. | (value adj2 money).mp. |
| 14. | (cost audit* or cost containment or cost saving*).mp. |
| 15. | ((financ* or money or monies or monetary or budget* or fiscal) adj4 (expenditure* or evaluat* or analys#s or measure* or comparison* or impact?)).mp. |
| 16. | (cost* adj3 (audit* or containment or saving* or expenditure* or evaluat* or analys#s or evaluat* or analys#s or measure* or comparison*)).mp. |
| 17. | (cost* utility or marginal analys#s or affordabilit* or cost minimi#ation or cost-consequence* or cost efficienc*).mp. |
| 18. | (risk-benefit or "return on investment" or "willing* to pay").mp. |
| 19. | (QALY or DALY).mp. |
| 20. | ("disability-adjusted life year*" or "quality-adjusted life year*").mp. |
| 21. | "cost* and benefit*".mp. |
| 22. | 9 or 10 or 11 or 12 or 13 or 14 or 15 or 16 or 17 or 18 or 19 or 20 or 21 |
| 23. | 8 and 22 |

**NHS EED Search Strategy**

NHS Economic Evaluation Database on 21^st^ June 2023. Repeated 11^th^ July 2024.

| 1 | MeSH DESCRIPTOR Postpartum Hemorrhage |
| --- | --- |
| 2 | MeSH DESCRIPTOR Costs and Cost Analysis |
| 3 | MeSH DESCRIPTOR Cost Allocation |
| 4 | MeSH DESCRIPTOR Cost-Benefit Analysis |
| 5 | MeSH DESCRIPTOR Cost Control |
| 6 | MeSH DESCRIPTOR Cost Savings |
| 7 | MeSH DESCRIPTOR Economics, Hospital |
| 8 | MeSH DESCRIPTOR Economics, Medical |
| 9 | MeSH DESCRIPTOR Economics, Nursing |
| 10 | MeSH DESCRIPTOR Economics, Pharmaceutical |
| 11 | MeSH DESCRIPTOR Health Care Rationing |
| 12 | MeSH DESCRIPTOR Health Care Costs |
| 13 | MeSH DESCRIPTOR Quality-Adjusted Life Years |
| 14 | MeSH DESCRIPTOR Cost of Illness |
| 15 | MeSH DESCRIPTOR Drug Utilization Review |
| 16 | (((postpartum or post-partum or postnatal) adj5 (haemorrhag* or hemorrhag* or bleed* or blood))) |
| 17 | (((postpartal or post-partal or puerperal) adj3 (haemorrhag* or hemorrhag* or bleed* or blood))) |
| 18 | #1 OR #16 OR #17 |
| 19 | ((cost-effective* or cost-benefit* or benefit-cost* or (economic evaluation*) or pharmacoeconomic* or pharmaco-economic* or cost analys*)) |
| 20 | (((economic* or cost* or price*) adj4 (evaluat* or analys* or measure* or comparison*))) |
| 21 | ((value adj2 money)) |
| 22 | (((cost audit*) or (cost containment) or (cost saving*))) |
| 23 | (((financ* or money or monies or monetary or budget*) adj4 (expenditure* or evaluat* or analys* or measure* or comparison* or impact*))) |
| 24 | (((cost* utility) or (marginal analys*) or affordabilit* or (cost minimisation) or (cost minimization) or cost-consequence* or (cost efficienc*))) |
| 25 | ((risk-benefit or (return on investment) or (willing* to pay))) |
| 26 | ((QALY or DALY)) |
| 27 | ((quality-adjusted life year*) or (disability-adjusted life year*)) |
| 28 | #19 OR #20 OR #21 OR #22 OR #23 OR #24 OR #25 OR #26 OR #27 |
| 29 | #2 OR #3 OR #4 OR #5 OR #6 OR #7 OR #8 OR #9 OR #10 OR #11 OR #12 OR #13 OR #14 OR #15 OR #28 |
| 30 | #18 AND #29 |

**CINAHL Search Strategy**

EBSCOhost Research Databases Search Screen - Advanced Search Database - CINAHL Complete on 20^th^ June 2023 and 11^th^ July 2024.

| **#** | **Query** | **Limiters/Expanders** |
| --- | --- | --- |
| S26 | S8 AND S25 | Search Modes- Boolean/Phrase |
| S25 | S9 OR S1O OR S11 OR S12 OR S13 OR S14 OR  S15 OR S16 OR S17 OR S18 OR 919 OR S20 OR S21 OR 922 OR S23 OR S24 | Search modes- Boolean/Phrase |
| S24 | "cost' and "benefit" | Search modes- SmartText Searching |
| S23 | QALY or DALY | Search modes- SmartText Searching |
| S22 | disability-adjusted life years OR quality-adjusted life years | Search Modes- Boolean/Phrase |
| S21 | risk-benefit OR "return on investment" or "willingness to pay" | Search modes- SmartText Searching |
| S20 | (financ* or money or monies or monetary or budget* or fiscal) n4 (expenditure* or evaluat* or analys#s or measure* or comparison*) | Search modes- SmartText Searching |
| S19 | (financ* or money or monies or monetary or budget* or fiscal) n4 (expenditure* or evaluat* or analys#s or measure* or comparison*) | Search modes- SmartText Searching |
| S18 | cost* utility or marginal analys#s or affordabilit* or cost minimisation or minimization or cost-consequence* or cost efficienc* | Search modes- SmartText Searching |
| S17 | cost* n3 (audit* or containment or saving* or expenditure* or evaluat* or analys#s or evaluat* or analys#s or measure* or comparison*) | Search modes- SmartText Searching |
| S16 | (financ* or money or monies or monetary or budget*) n4 (expenditure* or evaluat* or analys#s or measure* or comparison* or impact?) | Search modes- SmartText Searching |
| S15 | cost audit* or cost containment or cost saving* | Search modes- SmartText Searching |
| S14 | value n2 money | Search modes- SmartText Searching |
| S13 | (economic* or cost or costs or costly or costing or price or prices or pricing) n4 (evaluat* or analys#s or measure* or comparison*) | Search modes- SmartText Searching |
| S12 | cost-effective* or cost-benefit* or benefit-cost? or economic evaluation* or pharmacoeconomic* or pharmaco-economic* or cost analys?s | Search modes- SmartText Searching |
| S11 | Health Care Costs | Search Modes- Boolean/Phrase |
| S10 | Health Resource Allocation | Search Modes- Boolean/Phrase |
| S9 | "costs and cost analysis" or "cost allocation" or cost benefit analysis or "cost control" or "cost savings" or cost effectiveness analysis or economics, health care costs | Search Modes- Boolean/Phrase |
| S8 | S1 OR S2 OR S3 OR S4OR S5 OR S6 OR S7 | Search Modes- Boolean/Phrase |
| S7 | (following n2 (birth or childbirth) n8 ((severe or severity or massive* or excessive* or extensive* or large scale or acute or unrelenting or overwhelming or uncontrolled or extreme or significant or heavy or heavier or debilitat* or prolonged or excess or dysfunctional or abnormal or abundant or profuse) n3 (bleed* or blood loss* or blood flow*or haemorrhage or hemorrhage)) | Search modes- SmartText Searching |
| S6 | (after giving birth or after childbirth or after child-birth) n8 (haemorrhage or hemorrhage or bleed* or blood loss*) | Search modes- SmartText Searching |
| S5 | (postbirth or post-birth or post-childbirth or post-child-birth or postlabo?r or post-labo?r or postdelivery or postdelivery) n8 (haemorrhage or hemorrhage or bleed* or blood loss*) | Search modes- SmartText Searching |
| S4 | (obstetric delivery or vaginal delivery or c?esarian) n8 (severe or severity or massive* or excessive* or extensive* or large scale or acute or unrelenting or overwhelming or uncontrolled or extreme or significant or heavy or heavier or debilitat* or prolonged or excess or dysfunctional or abnormal or abundant or profuse) n3 (bleed* or blood loss* or blood flow* or haemorrhage or hemorrhage) | Search modes- SmartText Searching |
| S3 | (postpartal or post-partalor puerperal) n3 (haemorrhage or hemorrhage or bleed* or blood) | Search modes- SmartText Searching |
| S2 | (postpartum or post-partum or postnatal) n5 (haemorrhage or hemorrhage or bleed* or blood) | Search modes- SmartText Searching |
| S1 | Postpartum Hemorrhage | Search Modes- Boolean/Phrase |

**Global Index Medicus** **Search Strategy**

Search completed on 22^nd^ June 2023 and 11^th^ July 2024.

Topic 1:

cost-effective* or cost-benefit* or benefit-cost* or “economic evaluation” or pharmacoeconomic* or pharmaco-economic* or cost-utility or “marginal analysis” or ffordability* or “cost minimization” or “cost minimisation” or “cost efficiency” or “disability-adjusted life years” or “quality-adjusted life years” or QALY or DALY

topic 2:

“postpartum hemorrhage” or “postpartum haemorrhage” or “postpartal hemorrhage” or “postpartal haemorrhage” or postnatal haemorrhage” or “postnatal hemorrhage”

Topic 1 and 2 Together:

Tw:((tw:(cost-effective* OR cost-benefit* OR benefit-cost* OR “economic evaluation” OR pharmacoeconomic* OR pharmaco-economic* OR cost-utility OR “marginal analysis” OR ffordability* OR “cost minimization” OR “cost minimisation” OR “cost efficiency” OR “disability-adjusted life years” OR “quality-adjusted life years” OR qaly OR daly )) AND (tw:(“postpartum hemorrhage” OR “postpartum haemorrhage” OR “postpartal hemorrhage” OR “postpartal haemorrhage” OR postnatal haemorrhage” OR “postnatal hemorrhage” )))

**EconLit Search Strategy**

Search completed on 27^th^ June 2023 and 11^th^ July 2024.

| S12 | S1 OR S2 OR S3 OR S4 OR S5 OR S6 OR S7 OR S8 OR S9 OR S10 OR S11 |
| --- | --- |
| S11 | (“after giving birth” or “after childbirth” or “after child-birth”) N8 (“hemorrhage*” or “haemorrhage*” or “bleed*” or “blood loss*”) |
| S10 | (“after giving birth” or “after childbirth” or “after child-birth”) N8 (“hemorrhage*” or “haemorrhage*” or “bleed*” or “blood loss*”) |
| S9 | =((“postbirth” or “post-birth” or “post-childbirth” or “post-child-birth” or “postlabo?r” or “post-labo?r” or “postdelivery” or “postdelivery”) N8 (“hemorrhage*” or “haemorrhage*” or “bleed*” or “blood loss*”)) |
| S8 | =((“postbirth” or “post-birth” or “post-childbirth” or “post-child-birth” or “postlabo?r” or “post-labo?r” or “postdelivery” or “postdelivery”) N8 (“hemorrhage*” or “haemorrhage*” or “bleed*” or “blood loss*”)) |
| S7 | puerperal haemorrhag* OR puerperal hemorrhag* OR puerperal bleed* OR puerperal blood* |
| S6 | post-partal haemorrhag* OR post-partal hemorrhag* OR post-partal bleed* OR post-partal blood* |
| S5 | post-partal haemorrhag* OR post-partal hemorrhag* OR post-partal bleed* OR post-partal blood* |
| S4 | postpartal haemorrhag* OR postpartal hemorrhag* OR postpartal bleed* OR postpartal blood* |
| S3 | postnatal haemorrhag* OR postnatal hemorrhag* OR postnatal bleed* OR postnatal blood* |
| S2 | post-partum haemorrhag* OR post-partum hemorrhag* OR post-partum bleed* OR post-partum blood* |
| S1 | postpartum haemorrhag* OR postpartum hemorrhag* OR postpartum bleed* OR postpartum blood* |

**Web of Science Search Strategy**

Seaches completed on 27^th^ June 2023 and 11^th^ July 2024 on Web of Science Core Collection

| # | Search Query |
| --- | --- |
| 1 | ALL=(postpartum hemorrhage) |
| 2 | TS=((hemorrhage* or haemorrhage* or bleed* or blood) NEAR/5 (postpartum or post-partum or postnatal)) |
| 3 | TS=((postpartal or post-partal or puerperal) NEAR/3 (hemorrhage* or haemorrhage* or bleed* or blood)) |
| 4 | TS=((“postbirth” or “post-birth” or “post-childbirth” or “post-child-birth” or “postlabor” OR “postlabour” OR “post-labor” OR “post-labour” or “Post labour” or “post labor” or “postdelivery” or “postdelivery”) NEAR/8  (“hemorrhage*” or “haemorrhage*” or “bleed*” or “blood loss*”)) |
| 5 | TS=((“postbirth” or “post-birth” or “post-childbirth” or “post-child-birth” or “postlabo?r” or “post-labo?r” or “postdelivery” or “postdelivery”) NEAR/8 (“hemorrhage*” or “haemorrhage*” or “bleed*” or “blood loss*”)) |
| 6 | TS=((“after giving birth” or “after childbirth” or “after child-birth”) NEAR/8 (“hemorrhage*” or “haemorrhage*” or “bleed*” or “blood loss*”)) |
| 7 | #1 OR #2 OR #3 OR #4 OR #5 OR #6 |
| 8 | TS=(cost-effective* or cost-benefit* or benefit-cost? or economic evaluation* or pharmacoeconomic* or pharmaco-economic* or cost analys?s) |
| 9 | TS=((economic* or cost or costs or costly or costing or price or prices or pricing) NEAR/4 (evaluat* or analys?s or measure* or comparison*)) |
| 10 | TS=(value NEAR/2 money) |
| 11 | TS=(cost audit* or cost containment or cost saving*) |
| 12 | TS=((cost*) NEAR/3 (audit* or containment or saving* or expenditure* or evaluat* or analys?s or evaluat* or analys?s or measure* or comparison*)) |
| 13 | TS=(“cost* utility” or “marginal analys?s” or affordabilit* or “cost minimi?ation” or “cost-consequence*” or “cost efficienc*”) |
| 14 | TS=((financ* or money or monies or monetary or budget* or fiscal) NEAR/4 (expenditure* or evaluat* or analys?s or measure* or comparison* or impact?)) |
| 15 | TS=(risk-benefit or "return on investment" or "willing* to pay") |
| 16 | TS=(QALY or DALY or "disability-adjusted life year*" or "quality-adjusted life year*") |
| 17 | TS=(cost* and benefit*) |
| 18 | #17 OR #16 OR #15 OR #14 OR #13 OR #12 OR #11 OR #10 OR #9 OR #8 |
| 19 | #18 AND #7 |
