# Supplementary material for: The cost-effectiveness of preventing, diagnosing, and treating postpartum haemorrhage: A systematic review of economic evaluations
Source: PLoS Med. 2024 Sep 13;21(9):e1004461. doi: 10.1371/journal.pmed.1004461 (PMC11433145; doi:10.1371/journal.pmed.1004461)
Supplement: S3 Appendix — (DOCX) [file pmed.1004461.s003.docx]

**S3 Appendix: Data extraction variables**

Table A: Data extraction variables used for each study

| **Variable** | **Description** |
| --- | --- |
| **Study Aim** | The research question or the study aim |
| **Country** | The country of the study population or hypothetical population if a model was used.  If the intervention was tested over sites in multiple countries, all were recorded. |
| **Region (List)** | As defined by The World Bank analytical grouping  Drop Down Menu Options:   - East Asia & Pacific - Europe & Central Asia - Latin America & Caribbean - Middle East & North Africa - North America - South Asia - Sub-Saharan Africa - Multiple Regions |
| **Country income level (List)** | As defined by The World Bank for the 2024 fiscal year (from 2022 GNI data)  Drop Down Menu Options:   - High Income - Upper Middle Income - Lower Middle Income - Low Income - Not classified - Multiple |
| **Period of intervention** | Drop Down Menu Options:   - Antenatal Intervention - Intrapartum intervention - Postpartum intervention - Intervention covers multiple periods - N/A |
| **Population of interest** | The population in which the intervention was studied. If it is a modelling study, then the description of the hypothetical cohort being modelled was recorded |
| **Study setting** | The setting where the intervention and comparator were utilised (for example, hospitals, primary health centres, home births, etc). |
| **Intervention** | Description of the intervention.  (If multiple components, all were recorded) |
| **Comparator** | Description of the comparator.  (If multiple components, all were recorded) |
| **Number of Options Being Compared** | How many options did they compare in the study (intervention + comparators) |
| **Evaluation Type** | Review team’s assessment of the evaluation type undertaken, not necessarily the term used by study authors.   - Cost-effectiveness analysis - Cost-utility analysis - Cost-benefit Analysis - Cost-consequences analysis |
| **Study Design** | The study design used to complete the economic evaluation, such as an economic evaluation of an effectiveness study, or an economic evaluation based on modelling. |
| **Study Perspective** | The point of view taken to assess the health outcomes and costs. We recorded the perspective stated by study authors, not our interpretation of the costs and outcomes they included. |
| **Time Horizon** | The duration stated by study authors that they used to calculate the costs and health outcomes in the economic evaluation. |
| **Health Outcomes** | The health outcomes measured in the study. |
| **Health Outcome measure data source / methodology** | How health outcomes measured or modelled in the analysis. |
| **Cost data source / methodology** | How resource use and cost was measured or modelled in the analysis |
| **Currency of Costs** | Currency used to measure costs |
| **Year of Costs** | The year that all costs were converted to for the analysis |
| **Discounting** | If the duration of the follow up for the study or model is >1 year then the methodology for discounting future health utilities and costs was recorded. |
| **Incremental Cost effectiveness ratio (ICER)** | If the study calculated an ICER this was recorded. |
| **Results** | The difference in cost and health outcomes was recorded, including from sensitivity analyses |
| **Cost Effectiveness threshold** | If the study used a cost effectiveness or willingness to pay threshold was used in the study, this was recorded. |
| **Justification of threshold** | The justification for the above threshold, if used. |
| **Author conclusions** | The major points and conclusions made by the study authors. |

Abbreviations: GNI: Gross National Income. ICER: Incremental Cost-effectiveness Ratio.
