# Supplementary material for: The cost-effectiveness of preventing, diagnosing, and treating postpartum haemorrhage: A systematic review of economic evaluations
Source: PLoS Med. 2024 Sep 13;21(9):e1004461. doi: 10.1371/journal.pmed.1004461 (PMC11433145; doi:10.1371/journal.pmed.1004461)
Supplement: S4 Appendix — (DOCX) [file pmed.1004461.s004.docx]

**S4 Appendix: CHEC-Extended Questions**

Taken from Odnoletkova I, Goderis G, Pil L, Nobels F, Aertgeerts B, et al. (2014) Cost-Effectiveness of Therapeutic Education to Prevent the Development and Progression of Type 2 Diabetes: Systematic Review. J Diabetes Metab 5: 438 (Appendix)

Each article was scored out of 18, 19, or 20 depending on whether the questions on model structure (Q5) and utility values (Q13) were applicable to the study design.

Table A: CHEC-Extended Questions

| **#** | **Checklist Questions** |
| --- | --- |
| 1 | Is the study population *clearly* described? |
| 2 | Are competing alternatives *clearly* described? |
| 3 | Is a *well-defined* research question posed in answerable form? |
| 4 | Is the economic study design appropriate to the stated objective? |
| 5 | Are the structural assumptions and the validation methods of the model properly reported? |
| 6 | Is the chosen time horizon appropriate in order to include relevant costs and consequences? |
| 7 | Is the actual perspective chosen *appropriate*? |
| 8 | Are *all important and relevant* costs for each alternative identified? |
| 9 | Are all costs measured *appropriately* in physical units? |
| 10 | Are costs valued *appropriately*? |
| 11 | Are *all important and relevant* outcomes for each alternative identified? |
| 12 | Are all outcomes measured *appropriately*? |
| 13 | Are outcomes valued *appropriately*? |
| 14 | Is *an appropriate* incremental analysis of costs and outcomes of alternatives performed? |
| 15 | Are all future costs and outcomes discounted *appropriately*? |
| 16 | Are all important variables, whose values are uncertain, *appropriately* subjected to sensitivity analysis? |
| 17 | Do the conclusions follow from the data reported? |
| 18 | Does the study discuss the generalizability of the results to other settings and patient/client groups? |
| 19 | Does the article/report indicate that there is no potential conflict of interest of study researcher(s) and funder(s)? |
| 20 | Are ethical and distributional issues discussed *appropriately*? |
