# Supplementary material for: The cost-effectiveness of preventing, diagnosing, and treating postpartum haemorrhage: A systematic review of economic evaluations
Source: PLoS Med. 2024 Sep 13;21(9):e1004461. doi: 10.1371/journal.pmed.1004461 (PMC11433145; doi:10.1371/journal.pmed.1004461)
Supplement: S5 Appendix — (DOCX) [file pmed.1004461.s005.docx]

**S5 Appendix: Excluded Studies**

The following table contains a list of studies that might appear to meet the inclusion criteria when reviewing the title or abstract, but which were excluded. The reason for exclusion is also recorded.

Table A: Excluded Studies

| **Study** | **Explanation** |
| --- | --- |
| Achana FA, Fleming KM, Tata LJ, Sultan AA, Petrou S. Peripartum hysterectomy: an economic analysis of direct healthcare costs using routinely collected data. BJOG: An International Journal of Obstetrics & Gynaecology. 2018 Jun;125(7):874-83. | - Study design. - Explanation: Costing analysis only - not a complete economic analysis |
| Adam T, Lim SS, Mehta S, Bhutta ZA, Fogstad H, Mathai M, Zupan J, Darmstadt GL. Cost effectiveness analysis of strategies for maternal and neonatal health in developing countries. Bmj. 2005 Nov 10;331(7525):1107. | - Population and study design. - Explanation: Population is women and neonates, not specifically women at risk of PPH or experiencing PPH. - Study design – no comparator for PPH interventions. |
| Bandara S, Angala P, Haloob R. Carbitocin: A cost-effective tool to save lives!. BJOG - An International Journal of Obstetrics and Gynaecology 2017 Mar 1 (Vol. 124, pp. 27-27). | - Conference abstract only |
| Begum RA, Masud SB, Anderson R. Mentorship program and labour room protocol challenge to reduce the PPH, eclampsia, and CSR in Bangladesh, 2 years' experience. BJOG: An International Journal of Obstetrics and Gynaecology 2019 Jun 1 (Vol. 126, pp. 122-122). | - Conference abstract only |
| Benson AE, Rollins MD, Warrick CM, Metcalf RA, Falkner D, Cail K, Clark EA, Einerson BD. 35: Implementation of a new transfusion preparedness strategy on labor and delivery: safety and cost. American Journal of Obstetrics & Gynecology. 2020 Jan 1;222(1):S29-30. | - Conference abstract only |
| Bridges A, Doshi U, Caughey AB. Bakri balloon vs. jada device for treatment of postpartum hemorrhage: a cost-effective analysis. American Journal of Obstetrics & Gynecology. 2023 Jan 1;228(1):S600. | - Conference abstract only |
| Burke TF, Ahn R, Nelson BD, Hines R, Kamara J, Oguttu M, Dulo L, Achieng E, Achieng B, Natarajan A, Maua J. A postpartum haemorrhage package with condom uterine balloon tamponade: a prospective multi‐centre case series in Kenya, Sierra Leone, Senegal, and Nepal. BJOG: An International Journal of Obstetrics & Gynaecology. 2016 Aug;123(9):1532-40. | - Study design - Explanation: No economic analysis |
| Chatterjee S, Sarkar A, Rao KD. Using Misoprostol for Primary versus Secondary Prevention of Postpartum Haemorrhage–Do Costs Matter?. Plos one. 2016 Oct 18;11(10):e0164718. | - Study Design - Explanation: Partial economic evaluation only |
| Contreras I, Maldonado D, Gomez S, Olvera S, Dupuis R. Cost Effectiveness Analysis Of The Use Of Human Fibrinogen (Clottafact®) In Masive Post-Partum Hemorragea In Mexico. Value in Health. 2014 May 1;17(3):A160. | - Conference abstract only |
| Darwish AM, Abdallah MM, Shaaban OM, Ali MK, Khalaf M, Sabra AM. Bakri balloon versus condom-loaded Foley’s catheter for treatment of atonic postpartum hemorrhage secondary to vaginal delivery: a randomized controlled trial. The Journal of Maternal-Fetal & Neonatal Medicine. 2018 Mar 19;31(6):747-53. | - Study design - Explanation: No economic analysis |
| Dazelle WD, Ebner M, Kazma J, Ahmadzia HK. 972 Preventing postpartum hemorrhage in vaginal and cesarean deliveries: an expanded cost-effectiveness analysis of tranexamic acid. American Journal of Obstetrics & Gynecology. 2021 Feb 1;224(2):S602-3. | - Conference abstract only |
| Dumont A, Bodin C, Hounkpatin B, Popowski T, Traoré M, Perrin R, Rozenberg P. Uterine balloon tamponade as an adjunct to misoprostol for the treatment of uncontrolled postpartum haemorrhage: a randomised controlled trial in Benin and Mali. BMJ open. 2017 Sep 1;7(9):e016590. | - Study design - Explanation: No economic analysis |
| El Gelany SA, Soltan MH. External aortic compression device, manual aortic compression and el minya air inflated balloon: simple, cost-effective, and saving many lives in low resource settings. International Journal of Gynecology & Obstetrics. 2012 Oct;119:S335-. | - Conference abstract only |
| García GD, Garcia-Contreras F, Constantino-Casas P, Nevarez-Sida A, Lopez-Gonzalez N, Garcia-Constantino M, Zuñiga M. PIH6 economic evaluation of carbetocine for the prevention of uterine atony in patients with risk factors in Mexico. Value in Health. 2006;6(9):A254. | - Conference abstract only |
| Garg R, Yadav A. Condom balloon tamponade for postpartum hemorrhage in developing countries: Cost-effective boon for saving mothers. Journal of South Asian Federation of Obstetrics and Gynaecology. 2022 Mar;14(2):189-91. | - Study design - Explanation: Not an economic evaluation |
| Herrick T, Mvundura M, Burke TF, Abu-Haydar E. A low-cost uterine balloon tamponade for management of postpartum hemorrhage: modeling the potential impact on maternal mortality and morbidity in sub-Saharan Africa. BMC pregnancy and childbirth. 2017 Dec;17:1-6. | - Study design - Explanation: No economic analysis |
| Hofmeyr GJ, Middleton K, Singata‐Madliki M. Randomized feasibility study of suction‐tube uterine tamponade for postpartum hemorrhage. International Journal of Gynecology & Obstetrics. 2019 Sep;146(3):339-43. | - Study design - Explanation: No economic analysis |
| Howard DC, Skeith A, Caughey AB. Cost-Effectiveness of Prophylactic Tranexamic Acid Use in Women at Increased Risk of Postpartum Hemorrhage [22B]. Obstetrics & Gynecology. 2019 May 1;133:26S. | - Conference abstract only |
| Jain R, Agrawal S, Verma K, Jain A, Baid M. Comparison of intramuscular methylergometrine, rectal misoprostol, and low-dose intravenous oxytocin in active management of the third stage of labor. Tzu-Chi Medical Journal. 2019 Jul;31(3):158. | - Study design - Explanation: Not an economic evaluation – only states cost of medication dose. |
| Joshi B, Shetty S, Moray KV, Chaurasia H, Sachin O. POSC50 Cost-Effectiveness of Addition of Intravenous Tranexamic Acid to Management of Primary Post-Partum Haemorrhage in Indian Public Health Settings. Value in Health. 2022 Jan 1;25(1):S95-6. | - Conference abstract only |
| Kaya B, Guralp O, Tuten A, Unal O, Celik MO, Dogan A. Which uterine sparing technique should be used for uterine atony during cesarean section? The Bakri balloon or the B-Lynch suture?. Archives of gynecology and obstetrics. 2016 Sep;294:511-7. | - Study design - Explanation: No economic analysis |
| Kent E, Nguyen N, Griffin E, Munro E, Caughey A. 412: Prophylactic internal iliac balloon occlusion in reducing morbidity and mortality in placenta accreta: a decision and cost-effectiveness analysis. American Journal of Obstetrics & Gynecology. 2015 Jan 1;212(1):S215. | - Conference abstract only |
| Kerr NL, Hauswald M, Tamrakar SR, Wachter DA, Baty GM. An inexpensive device to treat postpartum hemorrhage: a preliminary proof of concept study of health provider opinion and training in Nepal. BMC pregnancy and childbirth. 2014 Dec;14(1):1-7. | - Population - Explanation: intervention trialled on non-pregnant participants. |
| Kostyuk A, Akanov A, Almadiyeva A. Clinical effectiveness and cost-effectiveness of carbetocin for the prevention of postpartum hemorrhage. Value in Health. 2016 May 1;19(3):A173. | - Conference abstract only |
| Lubinga SJ, Atukunda EC, Wasswa-Ssalongo G, Babigumira JB. Potential cost-effectiveness of prenatal distribution of misoprostol for prevention of postpartum hemorrhage in Uganda. PloS one. 2015 Nov 11;10(11):e0142550. | - Conference abstract only |
| Marotta C, Di Gennaro F, Pisani L, Pisani V, Senesie J, Bah S, Koroma MM, Caracciolo C, Putoto G, Amatucci F, Borgonovi E. Cost-utility of intermediate obstetric critical care in a resource-limited setting: a value-based analysis. Annals of Global Health. 2020;86(1). | - Study design - Explanation: No comparator used in economic evaluation. |
| Mills F, Siu E, Poinas AC, Chamy C. A cost-minimization analysis of carbetocin for the prevention of postpartum hemorrhage in Canada. Value in Health. 2014 May 1;17(3):A161. | - Conference abstract only |
| Mishra N, Gulabani K, Agrawal S, Shrivastava C. Efficacy and feasibility of Chhattisgarh balloon and conventional condom balloon tamponade: a 2-year prospective study. The Journal of Obstetrics and Gynecology of India. 2019 Oct;69:133-41. | - Study design - Explanation: Incomplete economic analysis |
| Mishra N, Shrivastava C, Agrawal S, Gulabani K. The CG balloon is an innovative condom balloon tamponade for the management of postpartum hemorrhage in low-resource settings. Int J Gynecol Obstet. 2016 Jun 1;133(3):377-8. | - Study design - Explanation: Not an economic evaluation |
| Moosivand A, Moghadam MF, Khedmati J, Mehralian G. Cost–utility analysis of carbetocin versus oxytocin for managing postpartum hemorrhage. Value in Health. 2016 May 1;19(3):A177. | - Conference abstract only |
| Osagie O, Gautam N, Stoddard K, Thakrar S. Audit on management of post-partum anaemia: cost-effectiveness analysis. InANAESTHESIA 2021 Jan 1 (Vol. 76, pp. 76-76). | - Conference abstract only |
| Pacocha K, Pieniazek I, Sobkowski M, Celewicz Z, Kalinka J, Szymanowski K, Serafin M, Szpak K, Grzymala-Figura A, Walczak J, Bierut A. Carbetocin In Prevention of Uterine Atony Following Delivery by Cesarean Section in Population Who Experienced Postpartum Hemorrhage: Costs in Polish Settings. Value in Health. 2016 May 1;19(3):A176. | - Conference abstract only |
| Perosky J, Richter R, Rybak O, Gans-Larty F, Mensah MA, Danquah A, Debpuur D, Kolbilla D, Ofosu A, Anderson F, Marzano D. A low-cost simulator for learning to manage postpartum hemorrhage in rural Africa. Simulation in Healthcare. 2011 Feb 1;6(1):42-7. | - Study design - Explanation: Not an economic evaluation |
| Purwosunu Y, Sarkoen W, Arulkumaran S, Segnitz J. Control of postpartum hemorrhage using vacuum-induced uterine tamponade. Obstetrics & Gynecology. 2016 Jul 1;128(1):33-6. | - Study design - Explanation: No economic analysis |
| Ransom SB, Fundaro G, Dombrowski MP. The cost-effectiveness of routine type and screen admission testing for expected vaginal delivery. Obstetrics & Gynecology. 1998 Oct 1;92(4):493-5. | - Study design - Explanation: Partial economic evaluation only. |
| Resch S, Guha M, Ward Z, Zarate SS, Borovac-Pinheiro A, Omotayo M, Garg L, Hansel S, Burke T. Cost-effectiveness of postpartum haemorrhage first response bundle and non-surgical interventions for refractory postpartum haemorrhage in India: an ex-ante modelling study. The Lancet Global Health. 2020 Apr 1;8:S42. | - Conference abstract only - Due to high relevance of topic, authors were contacted to request full analysis. - No response received from authors. |
| Rogers RG, Gardner MO, Tool KJ, Ainsley J, Gilson G. Active management of labor: a cost analysis of a randomized controlled trial. Western Journal of Medicine. 2000 Apr;172(4):240. | - Study design - Explanation: Partial economic evaluation only. |
| Rueda C, Caceres LA, Navas CA. Cost effectiveness of carbetocin compared with oxytocin to prevent postpartum hemorrhage due to uterine atony in patients with risk factors in Colombia [In Spanish]. In VALUE IN HEALTH 2013 Nov 1 (Vol. 16, No. 7, pp. A709-A710).  Alternate title in Spanish: “Costo-Efectividad De Carbetocina En Comparación Con Oxitocina Para Prevenir Hemorragia Posparto Por Atonía Uterina En Pacientes Con Factores De Riesgo En Colombia” | - Conference abstract only |
| Schmidt EM, Hersh AR, Skeith AE, Tuuli MG, Cahill AG, Caughey AB. Extending the second stage of labor in nulliparous women with epidural analgesia: a cost-effectiveness analysis. The Journal of Maternal-Fetal & Neonatal Medicine. 2022 Sep 17;35(18):3495-501. | - Indication - Explanation: Intervention intended to reduce progression to caesarean section, not reduce PPH. |
| Schmidt EM, Hersh AR, Tuuli M, Cahill AG, Caughey AB. Considering Criteria for Active Phase Labor Management of Nulliparous Women: A Cost-Effectiveness Analysis. American journal of perinatology. 2021 May 3;40(01):099-105. | - Indication - Explanation: intervention is intended to reduce the number of caesarean sections, not reduce the risk or incidence of PPH. |
| Seligman B, Liu X. Economic assessment of interventions for reducing postpartum hemorrhage in developing countries. Abt Associates; 2006. | - Grey literature |
| Shaw EH, Bækgaard E, Siassakos D, Draycott TJ. PL. 16 does the use of carbetocin reduce recovery times at caesarean section? An audit of outcomes following routine introduction of carbetocin at Southmead Hospital. Archives of Disease in Childhood-Fetal and Neonatal Edition. 2013 Apr 1;98(Suppl 1):A59-. | - Conference abstract only |
| Shaw EH, Bækgaard E, Siassakos D, Draycott TJ. PL. 19 cost comparison of routine carbetocin use at caesarean section. Archives of Disease in Childhood-Fetal and Neonatal Edition. 2013 Apr 1;98(Suppl 1):A59-60. | - Conference abstract only |
| Sikirica V, Broder MS, Chang E, Hinoul P, Robinson D, Wilson M. Clinical and economic impact of adhesiolysis during repeat cesarean delivery. Acta obstetricia et gynecologica Scandinavica. 2012 Jun;91(6):719-25. | - Indication - Explanation: the intervention was not to intended to improve the prevention, diagnosis or treatment of PPH. |
| Snegovskikh D, Walton Z, Souzdalnitski D. Point-of-care thromboelastometry in the management of acute obstetric hemorrhage. Current Obstetrics and Gynecology Reports. 2016 Sep;5:244-9. | - Review/Commentary article. |
| Theron GB. Management of postpartum hemorrhage with free‐flow pressure controlled uterine balloon. International Journal of Gynecology & Obstetrics. 2018 Sep;142(3):371-3. | - Study design - Explanation: No economic analysis |
| Theunissen F, Cleps I, Goudar S, Qureshi Z, Owa OO, Mugerwa K, Piaggio G, Gülmezoglu AM, Nakalembe M, Byamugisha J, Osoti A. Cost of hospital care of women with postpartum haemorrhage in India, Kenya, Nigeria and Uganda: a financial case for improved prevention. Reproductive health. 2021 Dec;18:1-8. | - Study design. - Explanation: Partial economic evaluation only. |
| Wohling J, Edge N, Pena-Leal D, Utama D, Wang R, Edge D, Dekker G, Mol B. Cost Comparison of Carbetocin Compared to Oxytocin as Primary Postpartum Haemorrhage (PPH) Prophylaxis at Caesarean Section. In Australian & New Zealand Journal of Obstetrics & Gynaecology 2018 Sep 1 (Vol. 58, pp. 88-88). | - Conference abstract only |
| Wong MS, Gregory KD, Almario CV. 755: Economic analysis of tranexamic acid: applying the WOMAN trial data to a contemporary US population. American Journal of Obstetrics & Gynecology. 2019 Jan 1;220(1):S495-6. | - Conference abstract only |

Abbreviations: PPH: Postpartum haemorrhage.
