# Supplementary material for: The cost-effectiveness of preventing, diagnosing, and treating postpartum haemorrhage: A systematic review of economic evaluations
Source: PLoS Med. 2024 Sep 13;21(9):e1004461. doi: 10.1371/journal.pmed.1004461 (PMC11433145; doi:10.1371/journal.pmed.1004461)
Supplement: S6 Appendix — (DOCX) [file pmed.1004461.s006.docx]

**S6 Appendix: Characteristics of Included Studies**

Table A: Characteristics of included studies presented by intervention type

| **Preventative Interventions for PPH** | | | | | | | | | | |
| --- | --- | --- | --- | --- | --- | --- | --- | --- | --- | --- |
| **Study** | **Country** | **Study setting** | **Population of interest** | **Study aim** | **Study design** | **Year of costs** | **Main Study Outcomes** | **Study perspective** | **Time horizon** | **Overall CHEC-E Quality Assessment** |
| Pickering et al., 2019 [1] | United Kingdom | Hospitals | VB Only. Hypothetical cohort of women delivering in the UK in a hospital. | To compare the relative cost -effectiveness of the full range of uterotonic drugs available for the prevention of PPH in VB. | Model (decision analytic) | 2016 | - Number of PPH cases averted (>500ml) - Number of severe PPH cases averted (>1000ml) - Number with major surgical outcomes | NHS - health system perspective | Immediate postpartum period | High (18/19) |
| Gallos et al., 2019 [2] | United Kingdom | Hospitals | VB & CS.  Hypothetical cohort of women delivering in an obstetric unit. | To identify the most effective and cost-effective uterotonic drug(s) to prevent PPH and generate a ranking according to their effectiveness and side-effect profile. | Model (decision analytic) | 2016 | - Number with PPH (>500ml) - Number with PPH (>1000ml) - Maternal deaths - Number requiring additional uterotonic - Number requiring blood transfusion - Number with uterotonic side effects | NHS - health system perspective | Immediate post-partum period | High (18/19) |
| Barrett et al., 2022 [3] | Canada | Hospitals | VB & CS.  Women giving birth in hospitals in the greater Toronto area of Canada, both at low risk and high risk of PPH. | To evaluate the annual cost of using carbetocin instead of oxytocin for primary PPH prevention at a Greater Toronto Area hospital. | Model (decision analytic) | 2020 | - Number without PPH - Number requiring additional uterotonics - Number requiring blood transfusion - Number requiring ICU admission | Hospital | Not stated | Moderate (11/19) |
| Cook et al., 2023 [4] | India | Multiple settings | VB & CS.  Hypothetical cohort of 100,000 women giving birth in public health facilities in India (primary, secondary, and tertiary facilities). | To evaluate the cost-effectiveness and budget impact of selected uterotonics for PPH prophylaxis. | Model (decision analytic) | 2021 | - DALYs averted - Number of deaths - Number of PPH events | Public Health system | Lifetime | High (18/20) |
| You et al., 2022 [5] | China | Hospitals | VB & CS.  Hypothetical cohort of women in third trimester delivering in Hong Kong public hospitals. | To examine the cost-effectiveness of carbetocin for the prevention of PPH. | Model (decision analytic) | 2022 | - QALYs Gained - Number of PPHs - Number requiring emergency hysterectomy - Deaths | Public health system | Postpartum hospitalization period | High (17/20) |
| Gil-Rojas et al., 2018 [6] | Colombia | Not stated | VB & CS.  Hypothetical cohort of women in Colombia with at least 1 risk factor for uterine atony. | To assess the cost-effectiveness of carbetocin versus oxytocin for prevention of PPH due to uterine atony after VB/CS in women with risk factors for bleeding. | Model (decision analytic) | 2016 | - QALYs gained - Number of PPH prevented (>500ml), - Number requiring additional uterotonics - Number requiring surgery - Number of deaths | Health system perspective | 1 year | High (16/20) |
| Briones et al., 2020 [7] | Philippines | Hospitals | VB & CS.  Women giving birth in Philippines public hospitals. | To evaluate the cost-utility and budget impact of carbetocin compared with oxytocin for PPH prophylaxis in both VB and CS in public hospitals. | Model (decision analytic) | 2019 | - QALYs Gained | Societal | 6 weeks | High (19/20) |
| Luni et al., 2017 [8] | United Kingdom | Hospitals | CS Only.   Women undergoing CS at Poole hospital in the UK from Nov 2014 to October 2015. | To report on the prospective evaluation of the use of carbetocin following CS following its introduction in October 2014. | EE of effectiveness study | Not stated | - Volume of blood loss (measured quantitatively) - Number requiring additional uterotonic - Number requiring admission to HDU/ICU - Length of stay in recovery room | Not stated | Not stated | Low (6/18) |
| van der Nelson et al., 2017 [9] | United Kingdom | Hospitals | CS Only. Hypothetical cohort of 1500 women undergoing elective or emergency CS in UK | To assess the cost-effectiveness of carbetocin for PPH prophylaxis at CS. | Model (decision analytic) | Not stated | - Number of PPH events | NHS - health system perspective | 1 year | High (18/20) |
| Wohling et al., 2019 [10] | Australia | Hospitals | CS Only.  A population of 2491 women with singleton pregnancies undergoing CS in a level 5 Australian hospital from 2008-2010. | To examine whether carbetocin confers clinical or economic benefit compared to oxytocin at CS in an all-risk Australian population. | EE of effectiveness study (retrospective cohort study) | Not stated | - Number of PPH >1000 ml - Number requiring transfusion - Number requiring secondary uterotonic | Not stated | Not stated | Moderate (9/18) |
| Caceda et al., 2018 [11] | Peru | Hospitals | CS Only.  Women undergoing CS in hospitals in Peru. | To compare the cost-effectiveness of carbetocin vs oxytocin for the prevention of PPH after CS. | Model (decision analytic) | 2015 | - QALYs gained - Number of PPH - Number of transfusions | Payer | 1 year | Moderate (12/20) |
| Henriquez-Trujillo et al., 2017 [12] | Ecuador | Hospitals | CS only.   Hypothetical cohort of women in Ecuador undergoing CS. | To compare the cost of carbetocin with that of oxytocin for the prevention and management of haemorrhage following CS in Ecuador. | Model (decision analytic) | 2015 | - DALYs averted | Public third-party payer | 1 year | High (16/20) |
| Voon et al., 2018 [13] | Malaysia | Hospitals | CS Only.   Hypothetical cohort of 3000 women undergoing CS in a high delivery rate hospital. | To examine the cost-effectiveness of carbetocin compared with oxytocin after CS, in a tertiary unit with high volumes of delivery. | Model (decision analytic) | 2016 | - Number of PPH events - Number requiring additional uterotonic - Number requiring transfusion | Ministry of Health | 24 hours | High (15/19) |
| Higgins et al., 2011 [14] | United Kingdom | Hospitals | CS Only.   165 women undergoing CS in Royal Bolton Hospital. | To describe a clinical and financial evaluation undertaken at a District General Hospital during the introduction of carbetocin for prophylaxis against PPH during CS. | EE of effectiveness study | Not stated | - PPH episodes - Number with postoperative hypotension. - Number requiring additional uterotonics - Perioperative change in Hb - Time spent in post-op recovery - Number with side effects | Not stated | Not stated (but appears to be from CS, until the time patient taken to post-delivery ward) | Low (7/18) |
| Matthijsse et al., 2022 [15] | United Kingdom | Hospitals | VB Only.   Hypothetical cohort of 100 women in UK. | To assess the cost per PPH event avoided attributable to the utilization of carbetocin monotherapy versus oxytocin monotherapy for the prevention of PPH following VB. | Model (decision analytic) | 2019 | - Number of PPH cases averted - QALYs gained | NHS and Personal Social Services - health system perspective | 30 days | High (16/20) |
| Vlassoff et al., 2016 [16] | Senegal | home birth/ "subcentre" with traditional birth attendants only | VB Only.   Two hypothetical cohorts of 150,000 women delivering over a one-year period. | To compare the use of oxytocin and misoprostol for the prevention of PPH in a community-based setting. | Model | 2013 | - Number requiring referral to a hospital or health centre for haemorrhage (as proxy for number with PPH). | Health system | 1 year | Moderate (10/19) |
| Diaz et al., 2009 [17] | Peru | Multiple Settings | VB & CS. Women giving birth in one of 74 hospitals and health centres chosen for the intervention. | To evaluate the impact of a programme (PARSalud) designed to reduce maternal mortality in Peru. | Model | Not stated | - Number of PPH cases averted | Not stated | Not stated | Low (6/19) |
| Tsu et al., 2009 [18] | Vietnam | Multiple settings | VB Only.   All women aged 18 and over undergoing VB that completed 2nd stage of labour in either commune or district hospital settings in Thanh Hoa Province. | To estimate the costs and cost-effectiveness of introducing routine AMTSL for the health system in Vietnam. | Model | 2004 | - Rates of PPH - Duration of third stage - Number requiring additional treatment | Health system | Not stated | High (15/19) |
| Pichon-Riviere et al., 2015 [19] | Multiple countries | Health facility | VB & CS.  Hypothetical cohorts of women delivering in each Latin American and Caribbean country. | To evaluate the cost-effectiveness of oxytocin administered via ampoules vs. switching to Uniject administration in Latin America and the Caribbean. | Model (decision analytic) | 2013 | - QALYs gained - Number with PPH - Number requiring post-delivery hysterectomy - Number of maternal deaths | Health system | Lifetime | High (19/20) |
| Carvalho et al., 2020 [20] | Bangladesh and Ethiopia | Multiple settings | VB Only.   Hypothetical cohorts of women in Bangladesh or Ethiopia, giving birth in hospitals, health centres, at home or in private hospitals. | To estimate the potential impact, costs, and cost-effectiveness of introducing an inhaled oxytocin product in two high-burden countries. | Model (decision analytic) | 2017 | - Maternal deaths - Number with non-severe PPH (500–1000ml blood loss) - Number with severe PPH (> 1000 ml) - PPH-related maternal death - Maternal death-related child deaths | Limited societal perspective (did not include loss of productivity) | Lifetime | High (17/19) |
| Sutherland et al., 2009 [21]  Note: Only the assessment of misoprostol met the inclusion criteria for this review (prenatal iron results excluded). | India | Home birth | VB Only.   A hypothetical cohort of 10,000 women delivering via home birth in rural India. | To determine the cost-effectiveness of prenatal iron supplementation and misoprostol use as interventions to prevent maternal mortality in home births in rural India. | Model (microsimulation) | 2008 | - Deaths due to haemorrhage | Not stated | Not stated | Moderate (11/19) |
| Sutherland et al., 2010 [22]  Note: this study appears in both prevention and treatment sections of this review. | India | Home birth | VB Only.   Hypothetical cohort of 10,000 women delivering at home or the community with unskilled providers in India. | To compare the cost-effectiveness of community-based distribution of misoprostol for prevention with misoprostol for treatment of PPH. | Model (microsimulation) | 2009 | - Deaths due to haemorrhage - DALYs averted - Number with severe anaemia | Health sector | Lifetime | Moderate (14/20) |
| Goldie et al., 2010 [23] | India | Multiple settings | VB & CS.  Hypothetical cohort of women becoming pregnant, receiving antenatal care, and delivering in the following settings: home, birthing centre, facility with basic emergency obstetric care, or a facility with advanced obstetric care. | To estimate the clinical and population-level benefits associated with a comprehensive set of strategies to improve the safety of pregnancy and childbirth in India. | Model (decision analytic) | 2006 | - Maternal mortality ratio | Not specified | Lifetime | High (16/19) |
| Lubinga et al., 2015 [24] | Uganda | Multiple settings | VB Only.   Women in Uganda delivering in a situation where there is no oxytocin (no trained worker or no supply). | To examine the cost-effectiveness of prenatal community distribution of misoprostol to pregnant mothers as a strategy to increase access to uterotonics for prevention of PPH. | Model (decision analytic) | 2012 | - DALYs averted - Number with PPH - Deaths due to PPH | Government (public health system payer)  and modified societal perspective | Lifetime | High (18/20) |
| Prata et al., 2010 [25]   Note: Only the comparison of ANC vs ANC-miso interventions met inclusion criteria for this review. | 34 countries in Sub-Saharan Africa | Multiple settings | VB/CS Not Specified. Hypothetical population of 500,000 women in Sub-Saharan Africa divided into low, medium and high infrastructure settings. | To guide policy-decision makers in prioritising the different components of safe motherhood programs in resource-scarce settings. | Model (simulation) | 2007 | - Maternal deaths averted | Health system | Not stated | Low (9/19) |
| Lang et al., 2015 [26] | "International" | Multiple settings | VB/CS not specified.  Hypothetical cohort of 1000 deliveries (40% hospital, 60% community setting). | To evaluate the costs and health outcomes of oral misoprostol to prevent PPH in settings where injectable uterotonics are not available. | Model | 2012 | - Number with PPH (>500ml) - Number with severe PPH ( ≥1000 mL) - Number requiring additional uterotonics - Number requiring transfusion - Number with uterotonic side effects | International health system | Not stated | Moderate (12/19) |
| Fullerton et al., 2006 [27] | Guatemala and Zambia | Hospitals | VB/CS Not Specified.   Hypothetical cohort of women delivering in hospitals in both rural and urban settings in either Guatemala or Zambia. | To calculate the net benefit of using AMTSL (uterotonic not specified) rather than EMTSL for mothers in Guatemala and Zambia. | Model (decision analytic) | 2004 | - Number with PPH - Maternal deaths - Number requiring additional uterotonic - Number requiring transfusion | Health facility | Not stated | Moderate (14/19) |
| Dazelle et al., 2023 [28] | United states | Not stated | VB & CS.  Hypothetical cohort of 3.8 million delivering women with an average age of 28y (matching USA deliveries in 2018). | To provide preliminary estimates of the cost-effectiveness of routine prophylaxis with TXA in a US-based, risk-stratified population. | Model (Markov) | 2020 | - QALYs gained - Number with PPH | Societal and Health system | Lifetime | High (19/20) |
| Durand-Zaleski et al., 2021 [29] | France | Hospitals | VB Only.   Women who were planned for VB of singleton fetus at 35 or more weeks gestation in 15 urban university hospitals. | To investigate whether the administration of tranexamic acid would reduce the cost of VB and be a cost-effective strategy. | EE of an effectiveness study | 2016 | - Number with PPH (>500ml) - Number of provider assessed clinically significant events - Number with PPH (>1000ml) | Hospital perspective | 3 months | High (17/18) |
| Sentilhes et al., 2023 [30] | France | Hospitals | CS Only.  Women aged 18 and over delivering in 27 French maternity hospitals. | To determine whether the administration of tranexamic acid reduces the cost of CS and is cost-effective. | EE of effectiveness study | 2019 | - Length of stay - Proportion transferred to ICU - Proportion re-hospitalised - Proportion with a complication - Total cost of hospital stay - Cost per additional CS delivery without complication | Hospital | 90 days post delivery | High (16/18) |
| Denison et al., 2019 [31,32] | United Kingdom | Hospitals | VB Only. Women with retained placenta following VB despite waiting 60 minutes after physiological management or 30 minutes after AMTSL. | To determine whether sublingual GTN is clinically effective and cost-effective for the management of retained placenta in a placebo-controlled double-blind pragmatic UK-wide RCT. | EE from effectiveness study | FY 2015/16 | - Number requiring manual removal of placenta - Blood loss - Patient satisfaction | NHS - health system perspective | Until 6 weeks post-partum | High (15/18) |
| Sharma et al., 2023 [33] | India | Hospital | VB only.  Women following VB without cervical or vaginal tears. | To assess the change in the incidence and outcomes of atonic PPH when using a negative intrauterine pressure suction device in addition to AMTSL. | EE of effectiveness study | Not stated. | - Blood loss - Proportion with atonic PPH - Haemoglobin - Haematocrit - Proportion requiring transfusion or blood products - Expenditure on blood products - Patient satisfaction - Doctor satisfaction | Not stated | Not stated | Moderate (9/18) |
| Hong et al., 2022 [34] | China | Hospitals | CS Only.   Women with placental accreta spectrum disorder undergoing CS in Ningbo Women and Children’s Hospital in Zhejiang, China. | To evaluate Internal Iliac Artery balloon occlusion during CS in patients with a diagnosis of placenta accreta spectrum. | EE of observational study | Not stated | - Estimated blood loss - Number requiring hysterectomy - Blood transfusion volume - Operating time - Number requiring intraoperative haemostatic approaches - Number with surgical complications - length of maternal stay | Not stated | Not stated | Low (4/18) |
| Niola et al., 2017 [35] | Italy | Hospitals | CS Only.   Women with placental implant abnormalities delivering in an Italian referral centre for high-risk pregnancies. | To compare standard endovascular embolization performed postpartum in CS with a novel embolization approach performed electively in selected high-risk patients. | EE of observational study | Not stated | - Percent requiring hysterectomy - Percent requiring transfusion | Not stated | Not stated | Low (8/18) |
| Xue et al., 2019 [36] | China | Hospitals | CS Only.   Women aged 25-40, undergoing elective CS (met the selected criteria for CS) - at Jiaxing Maternal and Child Health Care Hospital. | To examine the benefits of Multidisciplinary Enhanced Recovery after Surgery (MDT-ERAS) in CS. | EE of effectiveness study | Not stated | - Number with PPH - Rates of complications - Time to mobilising - Time to bowels opening - Duration of urinary catheter in place | Not reported | Not stated | Low (7/18) |
| **Diagnostic Interventions for PPH** | | | | | | | | | | |
| **Study** | **Country** | **Study setting (List)** | **Population of interest** | **Study aim** | **Study design** | **Year of costs** | **Main Study Outcomes** | **Study perspective** | **Time horizon** | **Overall CHEC-extended Quality Assessment** |
| Katz et al., 2020 [37] | United States of America | Hospitals | VB and CS. All women delivering in Mount Sinai Hospital. | To assess if adding quantitative measurement of blood loss would change the volume of blood lost and subsequent resource use and cost. | EE of effectiveness study | Not stated | - Percentage diagnosed with PPH - Volume of blood loss - Resource Utilization (treatments and blood products) | Not stated | Not Stated | Low (7/18) |
| **Treatment Interventions for PPH** | | | | | | | | | | |
| **Study** | **Country** | **Study setting (List)** | **Population of interest** | **Study aim** | **Study design** | **Year of costs** | **Main Study Outcomes** | **Study perspective** | **Time horizon** | **Overall CHEC-extended Quality Assessment** |
| Bradley et al., 2007 [38] | Sub-Saharan Africa (no country specified) | Not specified but a setting only staffed with TBAs | VB/CS not stated. Hypothetical cohort of 10,000 women in labour in Sub-Saharan Africa. | To test the cost-effectiveness of training TBAs to recognize PPH and administer a rectal dose of misoprostol in areas with low access to modern delivery facilities. | Model (decision analytic) | 2005 | - Number of PPH (>750ml blood loss) averted - Number requiring IV fluids - Number requiring blood transfusion | Health system | 1 year | High (16/19) |
| Howard et al., 2022 [39] | United States | Hospitals | VB and CS. Theoretical cohort of pregnant women in the United States undergoing treatment for acute PPH. | To strengthen the evidence that TXA should be recognized as a first-line treatment for PPH and whether early administration is a cost-effective strategy for reducing maternal morbidity and mortality. | Model (decision analytic) | 2019 | - QALYs gained - Deaths - Number requiring postpartum laparotomy or reoperation | Societal & Healthcare system | Lifetime | High (18/20) |
| Sudhof et al., 2019 [40] | United States | Hospitals | VB and CS.  Women with diagnosed PPH requiring second line uterotonics. | To demonstrate the cost-effectiveness of routine TXA administration in the treatment of PPH in the USA. | Model (decision analytic) | 2018 | - QALYs gained - Number of haemorrhage related deaths averted - Number of laparotomies averted | Healthcare system & Societal | From delivery until 6 weeks post-partum | High (18/20) |
| Joshi et al., 2023 [41] | India | Multiple settings | VB/CS not stated. Hypothetical cohort of women of reproductive age (with median age of 21 years at first childbirth) experiencing PPH after delivery at any Indian public health facility. | To determine the cost-effectiveness of administering IV TXA to women experiencing PPH within 3h of birth in addition to existing management strategies. | Model (decision analytic) | FY 2019/20 | - QALYs gained - Maternal deaths - Number requiring surgery - Number requiring ICU | Disaggregated societal | Lifetime | High (20/20) |
| Li et al., 2018 [42] | Nigeria & Pakistan | Hospitals | VB and CS. Women with PPH following delivery in Nigeria or Pakistan. | To evaluate the cost-effectiveness of TXA for treatment of PPH in Nigeria and Pakistan. | Model (decision analytic) | 2016 | - QALYs gained - Survival | Healthcare Provider | Lifetime | High (19/20) |
| Downing et al., 2015 [43] | Zambia & Zimbabwe | Primary Health Centres | VB/CS not stated. Women with obstetric haemorrhage >500ml (of any kind, except antepartum haemorrhage with viable fetus) presenting to primary health care settings. | To evaluate the cost-effectiveness of early NASG application at the primary health care level prior to transport compared to later NASG application at the referral hospital. | EE of effectiveness study | 2010 | - DALYs averted - Proportion requiring transfusion - Proportion requiring uterotonics | "Payer" | Lifetime | Moderate (13/20) |
| Sutherland et al., 2013 [44] | Egypt & Nigeria | Hospitals | VB/CS not stated.  Hypothetical cohort of 1000 women with hypovolemic shock due to obstetric haemorrhage in either Nigeria or Egypt. | To assess the cost-effectiveness of NASG for obstetric haemorrhage in tertiary hospitals in Egypt and Nigeria. | Model | 2010 | - DALYs averted - Maternal deaths - Number with severe maternal morbidity - Number with anaemia - Number with severe anaemia | Not stated | Lifetime | High (15/20) |
| Mvundura et al., 2017 [45] | Kenya | Multiple settings | VB/CS not stated. Hypothetical cohort of 1.5 million women delivering in Kenya (based on 2015 figures). | To evaluate the cost-effectiveness of condom-UBT for control of severe PPH due to uterine atony versus standard PPH care in Kenya. | Model (decision analytic) | 2015 | - DALYs averted - Number requiring hospital transfer - Number requiring hysterectomy - Maternal deaths | Health system | Lifetime | Moderate (13/20) |
| Joshi et al., 2021 [46] | India | Multiple - Primary, Secondary, and Tertiary level facilities | VB Only. Hypothetical cohort of Indian women with median age 21 years at first childbirth accessing public health facilities for atonic PPH. | To determine the most cost-effective UBT device for atonic PPH management in the Indian context. | Model (decision analytic) | 2017 | - DALYs averted | Disaggregated Societal | Lifetime | High (19/20) |
| Edwards et al., 2023 [47] | United Kingdom | Hospitals | VB Only. Women with PPH following delivery that did not respond to initial therapy. | To conduct early economic modelling to explore whether the PPH Butterfly device is likely to be cost-effective in comparison to standard treatment of PPH. | Model (decision analytic) | FY 2017/2018 | - Further blood loss >1000mls after device insertion - Number requiring transfer to theatre - Number requiring transfusion - Number requiring ICU - Number progressing to massive haemorrhage (≥2000 ml or transfusion of ≥4 units) - Maternal death | NHS - health system perspective | Not stated | High (15/19) |
| Snegovskikh et al., 2018 [48] | United States | Hospitals | VB and CS. Women with severe PPH treated at Yale-New Haven hospital. | To compare clinical outcomes and hospital costs for patients with severe PPH managed with and without the PCVT-guided transfusion protocol. | EE of effectiveness study | Not stated | - Estimated blood loss - Number requiring hysterectomy - Length of stay - Number requiring ICU | Not stated | Not stated | Low (8/18) |
| Einerson et al., 2017 [49] | United States | Hospitals | VB and CS. Hypothetical cohort of women in the USA (excluding those with accreta or previa). | To evaluate the cost-effectiveness of common obstetric transfusion preparedness strategies to minimise emergency-release transfusions. | Model (decision analytic) | 2015 | - Number of emergency-release transfusions prevented | Hospital | Until discharge for delivery admission | High (15/19) |
| Prick et al., 2014 [50] | Netherlands | Hospitals | VB and CS. Women with acute anaemia after PPH. | To assess the economic consequences of red blood cell transfusion compared to non-intervention. | EE of effectiveness study | 2013 | - Fatigue score measured by MFI - EuroQol-5D scores - Number with transfusion reactions - Number with physical complications | Hospital | Up to 6 weeks post-partum | High (17/19) |
| Khan et al., 2018 [51] | United Kingdom | Hospitals | CS Only. Women undergoing CS, either elective or emergency, at risk of haemorrhage. | To determine if routine use of cell salvage during CS in women at risk of haemorrhage is cost-effective in comparison with standard practice. | Model (decision analytic) | FY 2014/2015 | - Episodes of donor blood transfusion | NHS - health system perspective | Within-trial (until discharge) | High (18/19) |
| Lim et al., 2018 [52] | United States | Hospitals | CS only. Hypothetical cohort of women undergoing scheduled CS. | To determine under what circumstances the use of cell salvage strategies in obstetric haemorrhage during CS is cost-effective. | Model (Markov) | 2012 | - QALYs gained | Societal | Lifetime | High (18/20) |
| Ries et al., 2020 [53] | Switzerland | Hospitals | VB only. Women with PPH after VB in Basel University Hospital. | To compare the blood loss, and clinical management of severe PPH from VB before and after the implementation of the D-A-C-H treatment algorithm for PPH. | EE of effectiveness study | Not stated | - Estimated blood loss - Number requiring various surgical interventions - Time intervals between delivery and the initiation of a specific treatment | Not stated | Not stated | Low (8/18) |
| Franke et al., 2024 [54] | Madagascar | Primary Health Centres | VB or CS not stated.  Women with complications during or following delivery. | To describe case characteristics, service characteristics, and to analyse the costs and cost-effectiveness of an emergency obstetric referral system in rural Madagascar. | Model | 2020 | - ICER per life year saved per referral type - ICER per life year saved overall | Health Care Provider | Lifetime | High (17/19) |
| **Bundle Interventions for PPH: prevention and treatment bundles** | | | | | | | | | | |
| **Study** | **Country** | **Study setting (List)** | **Population of interest** | **Study aim** | **Study design** | **Year of costs** | **Main Study Outcomes** | **Study perspective** | **Time horizon** | **Overall CHEC-extended Quality Assessment** |
| Seim et al., 2023 [55] | Niger | Hospitals and Health centres | VB and CS. All women delivering in hospitals or health centres in Niger. | To describe the outcomes of the 72-month (2015-20) national implementation of a strategy to reduce death from bleeding at childbirth in Niger. | EE of effectiveness study | 2013 | - Costs - Incident rate ratio of PPH - Number of maternal deaths - DALYs averted | Not stated | Not stated | Low (5/19) |
| Wiesehan et al., 2023 [56] | United States of America | Hospitals | VB and CS. A hypothetical cohort of women delivering in Californian hospitals. | To model the cost-effectiveness of the statewide perinatal quality collaborative initiative to reduce severe maternal morbidity from PPH. | Model (decision analytic and Markov) | 2021 | - Costs - QALYs gained - Number of severe maternal morbidity events - Number requiring hysterectomy - Maternal Mortality | Health System | Lifetime | High (19/20) |
| **Bundle Interventions for PPH: early detection and treatment bundles** | | | | | | | | | | |
| **Study** | **Country** | **Study setting (List)** | **Population of interest** | **Study aim** | **Study design** | **Year of costs** | **Main Study Outcomes** | **Study perspective** | **Time horizon** | **Overall CHEC-extended Quality Assessment** |
| Dale et al., 2022 [57] | Wales | Hospitals | VB and CS. All women delivering in Obstetric Units in Wales. | To compare the incremental cost and outcomes of OBS Cymru (the quality improvement initiative) to standard PPH care in Wales. | EE of effectiveness study | 2019 | - Costs - Episodes of PPH >1000mL - Episodes of PPH >2500mL | Health System | From delivery until discharge from hospital | Moderate (14/19) |
| Williams et al., 2024 [58] | Kenya, Nigeria, South Africa, Tanzania | Hospitals | VB only. Women undergoing VB in one of the 78 hospitals included in the study. | To determine the cost-effectiveness of early diagnosis of PPH with calibrated drapes and management of PPH using the WHO first-response treatment bundle. | EE of effectiveness study. | 2022 | - Cases of PPH>1000mL averted - DALYs averted | Health System | Lifetime | High (18/19) |

Abbreviations: AMTSL: Active management of the third stage of labour. ANC: Antenatal care. CS: Caesarean section. D-A-C-H: Germany (Deutschland), Austria (Austria), Switzerland (Confoederatio Helvetica). DALYs: Disability adjusted life years. EE: Economic evaluation. EMTSL: Expectant management of the third stage of labour. FY: Fiscal Year. GTN: Glyceryl trinitrate. HDU: High Dependency Unit. Hb: Haemoglobin. ICER: Incremental cost-effectiveness ratio. ICU: Intensive care unit. IV: Intravenous. MDT-ERAS: Multidisciplinary Enhanced Recovery after Surgery. MFI: Multidimensional Fatigue Inventory. Multidimensional Fatigue Inventory. NASG: Non-pneumatic anti-shock garment. NHS: National health service. PCVT: Point-of-care viscoelastic testing. PPH: Postpartum haemorrhage. QALYs: Quality-adjusted life years. RCT: Randomised control trial. TBA: Traditional birth attendants. TXA: Tranexamic acid. UBT: Uterine balloon tamponade. VB: Vaginal birth. WHO: World Health Organization.

# **References**

1. Pickering K, Gallos ID, Williams H, Price MJ, Merriel A, Lissauer D, et al. Uterotonic drugs for the prevention of postpartum haemorrhage: a cost-effectiveness analysis. Pharmacoecon Open. 2019;3:163–76. doi: 10.1007/s41669-018-0108-x.

2. Gallos I, Williams H, Price M, Pickering K, Merriel A, Tobias A, et al. Uterotonic drugs to prevent postpartum haemorrhage: a network meta-analysis. Health Technol Assess. 2019;23(9). doi: 10.3310/hta23090.

3. Barrett J, Ko S, Jeffery W. Cost implications of using carbetocin injection to prevent postpartum hemorrhage in a Canadian urban Hospital. J Obstet Gynaecol Can. 2022;44(3):272–8. doi: 10.1016/j.jogc.2021.09.022.

4. Cook JR, Saxena K, Taylor C, Jacobs JL. Cost-effectiveness and budget impact of heat-stable carbetocin compared to oxytocin and misoprostol for the prevention of postpartum hemorrhage (PPH) in women giving birth in India. BMC Health Serv Res. 2023;23(1):267. doi: 10.1186/s12913-023-09263-4.

5. You JH, Leung T-y. Cost-effectiveness analysis of carbetocin for prevention of postpartum hemorrhage in a low-burden high-resource city of China. PLoS One. 2022;17(12):e0279130. doi: 10.1371/journal.pone.0279130.

6. Gil-Rojas Y, Lasalvia P, Hernández F, Castañeda-Cardona C, Rosselli D. Cost-effectiveness of Carbetocin versus Oxytocin for Prevention of Postpartum Hemorrhage Resulting from Uterine Atony in Women at high-risk for bleeding in Colombia. Rev Bras Ginecol Obstet. 2018;40:242–50. doi: 10.1055/s-0038-1655747.

7. Briones JR, Talungchit P, Thavorncharoensap M, Chaikledkaew U. Economic evaluation of carbetocin as prophylaxis for postpartum hemorrhage in the Philippines. BMC Health Serv Res. 2020;20:1–12. doi: 10.1186/s12913-020-05834-x.

8. Luni Y, Borakati A, Matah A, Skeats K, Eedarapalli P. A prospective cohort study evaluating the cost-effectiveness of carbetocin for prevention of postpartum haemorrhage in caesarean sections. J Obstet Gynaecol Can. 2017;37(5):601–4. doi: 10.1080/01443615.2017.1284188.

9. Van Der Nelson HA, Draycott T, Siassakos D, Yau CW, Hatswell AJ. Carbetocin versus oxytocin for prevention of post-partum haemorrhage at caesarean section in the United Kingdom: an economic impact analysis. Eur J Obstet Gynecol Reprod Biol. 2017;210:286-91. doi: 10.1016/j.ejogrb.2017.01.004.

10. Wohling J, Edge N, Pena‐Leal D, Wang R, Mol BW, Dekker G. Clinical and financial evaluation of carbetocin as postpartum haemorrhage prophylaxis at caesarean section: A retrospective cohort study. Aust N Z J Obstet Gynaecol. 2019;59(4):501–7. doi: 10.1111/ajo.12907.

11. Caceda SI, Ramos RR, Saborido CM. Pharmacoeconomic study comparing carbetocin with oxytocin for the prevention of hemorrhage following cesarean delivery in Lima, Peru. J Comp Eff Res. 2018;7(1):49-55. doi: 10.2217/cer-2017-0012.

12. Henríquez-Trujillo AR, Lucio-Romero RA, Bermúdez-Gallegos K. Analysis of the cost–effectiveness of carbetocin for the prevention of hemorrhage following cesarean delivery in Ecuador. J Comp Eff Res. 2017;6(6):529–36. doi: 10.2217/cer-2017-0004.

13. Voon HY, Shafie AA, Bujang MA, Suharjono HN. Cost effectiveness analysis of carbetocin during cesarean section in a high volume maternity unit. J Obstet Gynaecol Res. 2018;44(1):109–16. doi: 10.1111/jog.13486.

14. Higgins L, Mechery J, Tomlinson A. Does carbetocin for prevention of postpartum haemorrhage at caesarean section provide clinical or financial benefit compared with oxytocin? J Obstet Gynaecol. 2011;31(8):732–9. doi: 10.3109/01443615.2011.595982.

15. Matthijsse S, Andersson FL, Gargano M, Yip Sonderegger YL. Cost-effectiveness analysis of carbetocin versus oxytocin for the prevention of postpartum hemorrhage following vaginal birth in the United Kingdom. J Med Econ. 2022;25(1):129–37. doi: 10.1080/13696998.2022.2027669.

16. Vlassoff M, Diallo A, Philbin J, Kost K, Bankole A. Cost-effectiveness of two interventions for the prevention of postpartum hemorrhage in Senegal. Int J Gynaecol Obstet. 2016;133(3):307–11. doi: 10.1016/j.ijgo.2015.10.015.

17. Jose Diaz J, Jaramillo M. Evaluating interventions to reduce maternal mortality: evidence from Peru's PARSalud programme. J Dev Effect. 2009;1(4):387–412. doi: 10.1080/19439340903380872.

18. Tsu VD, Levin C, Tran MP, Hoang MV, Luu HT. Cost-effectiveness analysis of active management of third-stage labour in Vietnam. Health Policy Plan. 2009;24(6):438–44. doi: 10.1093/heapol/czp020.

19. Pichon-Riviere A, Glujovsky D, Garay OU, Augustovski F, Ciapponi A, Serpa M, et al. Oxytocin in uniject disposable auto-disable injection system versus standard use for the prevention of postpartum hemorrhage in latin America and the Caribbean: a cost-effectiveness analysis. PLoS One. 2015;10(6):e0129044. doi: 10.1371/journal.pone.0129044.

20. Carvalho N, Hoque ME, Oliver VL, Byrne A, Kermode M, Lambert P, et al. Cost-effectiveness of inhaled oxytocin for prevention of postpartum haemorrhage: a modelling study applied to two high burden settings. BMC Med. 2020;18(1):1–18. doi: 10.1186/s12916-020-01658-y.

21. Sutherland T, Bishai DM. Cost-effectiveness of misoprostol and prenatal iron supplementation as maternal mortality interventions in home births in rural India. Int J Gynaecol Obstet. 2009;104(3):189–93. doi: 10.1016/j.ijgo.2008.10.011.

22. Sutherland T, Meyer C, Bishai DM, Geller S, Miller S. Community-based distribution of misoprostol for treatment or prevention of postpartum hemorrhage: cost-effectiveness, mortality, and morbidity reduction analysis. Int J Gynaecol Obstet. 2010;108(3):289–94. doi: 10.1016/j.ijgo.2009.11.007.

23. Goldie SJ, Sweet S, Carvalho N, Natchu UCM, Hu D. Alternative strategies to reduce maternal mortality in India: a cost-effectiveness analysis. PLoS Med. 2010;7(4):e1000264. doi: 10.1371/journal.pmed.1000264.

24. Lubinga SJ, Atukunda EC, Wasswa-Ssalongo G, Babigumira JB. Potential cost-effectiveness of prenatal distribution of misoprostol for prevention of postpartum hemorrhage in Uganda. PLoS One. 2015;10(11):e0142550. doi: 10.1371/journal.pone.0142550.

25. Prata N, Sreenivas A, Greig F, Walsh J, Potts M. Setting priorities for safe motherhood interventions in resource-scarce settings. Health Policy. 2010;94(1):1–13. doi: 10.1016/j.healthpol.2009.08.012.

26. Lang DL, Zhao F-L, Robertson J. Prevention of postpartum haemorrhage: cost consequences analysis of misoprostol in low-resource settings. BMC Pregnancy Childbirth. 2015;15(1):1–9. doi: 10.1186/s12884-015-0749-z.

27. Fullerton JT, Frick KD, Fogarty LA, Fishel JD, Vivio DM. Active management of third stage of labour saves facility costs in Guatemala and Zambia. J Health Popul Nutr. 2006;24(4):540.

28. Dazelle WD, Ebner MK, Kazma J, Potarazu SN, Ahmadzia HK. Tranexamic acid for the prevention of postpartum hemorrhage: a cost-effectiveness analysis. J Thromb Thrombolysis. 2023:1–9. doi: 10.1007/s11239-023-02814-w.

29. Durand‐Zaleski I, Deneux‐Tharaux C, Seco A, Malki M, Frenkiel J, Sentilhes L, et al. An economic evaluation of tranexamic acid to prevent postpartum haemorrhage in women with vaginal delivery: the randomised controlled TRAAP trial. BJOG. 2021;128(1):114–20. doi: 10.1111/1471-0528.16456.

30. Sentilhes L, Bénard A, Madar H, Froeliger A, Petit S, Deneux-Tharaux C. Tranexamic acid for reduction of blood loss after Caesarean delivery: a cost-effectiveness analysis of the TRAAP2 trial. Br J Anaesth. 2023;131(5):893-900. doi: 10.1016/j.bja.2023.07.028.

31. Denison FC, Carruthers KF, Hudson J, McPherson G, Chua GN, Peace M, et al. Nitroglycerin for treatment of retained placenta: A randomised, placebo-controlled, multicentre, double-blind trial in the UK. PLoS Med. 2019;16(12):e1003001. doi: 10.1371/journal.pmed.1003001.

32. Denison FC, Carruthers KF, Hudson J, McPherson G, Scotland G, Brook-Smith S, et al. Glyceryl trinitrate to reduce the need for manual removal of retained placenta following vaginal delivery: the GOT-IT RCT. Health Technol Assess. 2019;23(70):1–72. doi: 10.3310/hta23700.

33. Sharma JC, Kollabathula P, Jindal S, Anupma A, Sarkar A, Jaggarwal S, et al. Application of a Negative Intrauterine Pressure Suction Device for Prophylactic Management of Atonic Postpartum Hemorrhage: A Quality Improvement Study. Cureus. 2023;15(7):e42631. doi: 10.7759/cureus.42631.

34. Hong L, Chen A, Chen J, Li X, Zhuang W, Shen Y, et al. The clinical evaluation of IIA balloon occlusion in caesarean delivery for patients with PAS: a retrospective study. BMC Pregnancy Childbirth. 2022;22(1):103. doi: 10.1186/s12884-022-04434-3.

35. Niola R, Giurazza F, Torbica A, Schena E, Silvestre M, Maglione F. Predelivery uterine arteries embolization in patients with placental implant anomalies: a cost-effective procedure. Radiol Med. 2017;122:77–9. doi: 10.1007/s11547-016-0690-x.

36. Xue L, Zhang J, Shen H, Hou Y, Ai L, Cui X. The application of rapid rehabilitation model of multidisciplinary cooperation in cesarean section and the evaluation of health economics. Zhonghua Yi Xue Za Zhi. 2019;99(42):3335–9. doi: 10.3760/cma.j.issn.0376-2491.2019.42.012.

37. Katz D, Wang R, O'Neil L, Gerber C, Lankford A, Rogers T, et al. The association between the introduction of quantitative assessment of postpartum blood loss and institutional changes in clinical practice: an observational study. Int J Obstet Anesth. 2020;42:4–10. doi: 10.1016/j.ijoa.2019.05.006.

38. Bradley SE, Prata N, Young-Lin N, Bishai D. Cost-effectiveness of misoprostol to control postpartum hemorrhage in low-resource settings. Int J Gynaecol Obstet. 2007;97(1):52–6. doi: 10.1016/j.ijgo.2006.12.005.

39. Howard DC, Jones AE, Skeith A, Lai J, D'Souza R, Caughey AB. Tranexamic acid for the treatment of postpartum hemorrhage: a cost-effectiveness analysis. Am J Obstet Gynecol MFM. 2022;4(3):100588. doi: 10.1016/j.ajogmf.2022.100588.

40. Sudhof LS, Shainker SA, Einerson BD. Tranexamic acid in the routine treatment of postpartum hemorrhage in the United States: a cost-effectiveness analysis. Am J Obstet Gynecol. 2019;221(3):275. e1–. e12. doi: 10.1016/j.ajog.2019.06.030.

41. Joshi BN, Shetty SS, Moray KV, Chaurasia H, Sachin O. Cost-effectiveness and budget impact of adding tranexamic acid for management of post-partum hemorrhage in the Indian public health system. BMC Pregnancy Childbirth. 2023;23(1):9. doi: 10.1186/s12884-022-05308-4.

42. Li B, Miners A, Shakur H, Roberts I. Tranexamic acid for treatment of women with post-partum haemorrhage in Nigeria and Pakistan: a cost-effectiveness analysis of data from the WOMAN trial. Lancet Glob Health. 2018;6(2):e222–e8. doi: 10.1016/S2214-109X(17)30467-9.

43. Downing J, El Ayadi A, Miller S, Butrick E, Mkumba G, Magwali T, et al. Cost-effectiveness of the non-pneumatic anti-shock garment (NASG): evidence from a cluster randomized controlled trial in Zambia and Zimbabwe. BMC Health Serv Res. 2015;15(1):1–10. doi: 10.1186/s12913-015-0694-6.

44. Sutherland T, Downing J, Miller S, Bishai DM, Butrick E, Fathalla MM, et al. Use of the non-pneumatic anti-shock garment (NASG) for life-threatening obstetric hemorrhage: a cost-effectiveness analysis in Egypt and Nigeria. PloS One. 2013;8(4):e62282. doi: 10.1371/journal.pone.0062282.

45. Mvundura M, Kokonya D, Abu‐Haydar E, Okoth E, Herrick T, Mukabi J, et al. Cost‐effectiveness of condom uterine balloon tamponade to control severe postpartum hemorrhage in Kenya. Int J Gynaecol Obstet. 2017;137(2):185–91. doi: 10.1002/ijgo.12125.

46. Joshi BN, Shetty SS, Moray KV, Sachin O, Chaurasia H. Cost-effectiveness of uterine balloon tamponade devices in managing atonic post-partum hemorrhage at public health facilities in India. PLoS One. 2021;16(8):e0256271. doi: 10.1371/journal.pone.0256271.

47. Edwards RT, Ezeofor V, Bryning L, Anthony BF, Charles JM, Weeks A. Prevention of postpartum haemorrhage: Economic evaluation of the novel butterfly device in a UK setting. Eur J Obstet Gynecol Reprod Biol. 2023;283:149–57. doi: 10.1016/j.ejogrb.2023.02.020.

48. Snegovskikh D, Souza D, Walton Z, Dai F, Rachler R, Garay A, et al. Point-of-care viscoelastic testing improves the outcome of pregnancies complicated by severe postpartum hemorrhage. J Clin Anesth. 2018;44:50–6. doi: 10.1016/j.jclinane.2017.10.003.

49. Einerson BD, Stehlikova Z, Nelson RE, Bellows BK, Kawamoto K, Clark EA. Transfusion preparedness strategies for obstetric hemorrhage: a cost-effectiveness analysis. Obstet Gynecol. 2017;130(6):1347–55. doi: 10.1097/AOG.0000000000002359.

50. Prick B, Duvekot J, Van Der Moer P, van Gemund N, Van Der Salm P, Jansen A, et al. Cost‐effectiveness of red blood cell transfusion vs. non‐intervention in women with acute anaemia after postpartum haemorrhage. Vox Sang. 2014;107(4):381–8. doi: 10.1111/vox.12181.

51. Khan KS, Moore P, Wilson M, Hooper R, Allard S, Wrench I, et al. A randomised controlled trial and economic evaluation of intraoperative cell salvage during caesarean section in women at risk of haemorrhage: the SALVO (cell SALVage in Obstetrics) trial. Health Technol Assess. 2018;22(2):1–88. doi: 10.3310/hta22020.

52. Lim G, Melnyk V, Facco FL, Waters JH, Smith KJ. Cost-effectiveness analysis of intraoperative cell salvage for obstetric hemorrhage. Anesthesiology. 2018;128(2):328–37. doi: 10.1097/ALN.0000000000001981.

53. Ries J-J, Jeker L, Neuhaus M, Vogt DR, Girard T, Hoesli I. Implementation of the D-A-CH postpartum haemorrhage algorithm after severe postpartum bleeding accelerates clinical management: A retrospective case series. Eur J Obstet Gynecol Reprod Biol. 2020;247:225–31. doi: 10.1016/j.ejogrb.2020.01.001.

54. Franke MA, Nordmann K, Frühauf A, Ranaivoson RM, Rebaliha M, Rapanjato Z, et al. Inter-facility transfers for emergency obstetrical and neonatal care in rural Madagascar: a cost-effectiveness analysis. BMJ Open. 2024;14(4):e081482. doi: 10.1136/bmjopen-2023-081482.

55. Seim AR, Alassoum Z, Souley I, Bronzan R, Mounkaila A, Ahmed LA. The effects of a peripartum strategy to prevent and treat primary postpartum haemorrhage at health facilities in Niger: a longitudinal, 72-month study. Lancet Glob Health. 2023;11(2):e287–e95. doi: 10.1016/S2214-109X(22)00518-6.

56. Wiesehan EC, Keesara SR, Krissberg JR, Main EK, Goldhaber-Fiebert JD. State perinatal quality collaborative for reducing severe maternal morbidity from hemorrhage: a cost-effectiveness analysis. Obstet Gynecol. 2023;141(2):387–94. doi: 10.1097/AOG.0000000000005060.

57. Dale M, Bell SF, O’Connell S, Scarr C, James K, John M, et al. What is the economic cost of providing an all Wales postpartum haemorrhage quality improvement initiative (OBS Cymru)? A cost-consequences comparison with standard care. Pharmacoecon Open. 2022;6(6):847–57. doi: 10.1007/s41669-022-00362-2

58. Williams EV, Goranitis I, Oppong R, Perry SJ, Devall AJ, Martin JT, et al. A cost-effectiveness analysis of early detection and bundled treatment of postpartum hemorrhage alongside the E-MOTIVE trial. Nat Med. 2024. doi: 10.1038/s41591-024-03069-5.
