# Supplementary material for: The cost-effectiveness of preventing, diagnosing, and treating postpartum haemorrhage: A systematic review of economic evaluations
Source: PLoS Med. 2024 Sep 13;21(9):e1004461. doi: 10.1371/journal.pmed.1004461 (PMC11433145; doi:10.1371/journal.pmed.1004461)
Supplement: S7 Appendix — (DOCX) [file pmed.1004461.s007.docx]

**S7 Appendix: CHEC-Extended Scores**

Table A: CHEC-Extended scores for studies assessing preventative interventions for PPH

| **Study** | **Patient population** | **Competing alternatives** | **Research question** | **Economic study design** | **Model description** | **Time horizon** | **Study perspective** | **Costs identification** | **Costs measurement** | **Costs valuation** | **Outcomes identification** | **Outcomes measurement** | **Outcomes valuation** | **Incremental CE analysis** | **Discounting** | **Uncertainty analysis** | **Conclusions** | **Generalizability** | **Conflict of interests** | **Ethical issues** | **Score** |  |
| --- | --- | --- | --- | --- | --- | --- | --- | --- | --- | --- | --- | --- | --- | --- | --- | --- | --- | --- | --- | --- | --- | --- |
| Pickering et al., 2019 [1] | ✓ | ✓ | ✓ | ✓ | ✓ | ✓ | ✓ | ✓ | ✓ | ✓ | ✓ | ✓ | N | ✓ | ✓ | ✓ | ✓ | ✓ | ✓ | X | 18/19 | High |
| Gallos et al., 2019 [2] | ✓ | ✓ | ✓ | ✓ | ✓ | ✓ | ✓ | ✓ | ✓ | ✓ | ✓ | ✓ | N | ✓ | ✓ | ✓ | ✓ | X | ✓ | ✓ | 18/19 | High |
| Barrett et al., 2021 [3] | ✓ | X | ✓ | ✓ | ✓ | X | X | ✓ | ✓ | ✓ | X | ✓ | N | X | ✓ | X | X | ✓ | ✓ | X | 11/19 | Moderate |
| Cook et al., 2023 [4] | ✓ | ✓ | ✓ | ✓ | ✓ | ✓ | ✓ | ✓ | ✓ | ✓ | ✓ | ✓ | ✓ | ✓ | ✓ | ✓ | ✓ | ✓ | X | X | 18/20 | High |
| You et al., 2022 [5] | ✓ | ✓ | ✓ | ✓ | ✓ | ✓ | ✓ | ✓ | ✓ | X | ✓ | ✓ | ✓ | ✓ | ✓ | ✓ | ✓ | ✓ | X | X | 17/20 | High |
| Gil-Rojas et al., 2018 [6] | ✓ | ✓ | ✓ | ✓ | ✓ | ✓ | ✓ | ✓ | ✓ | X | ✓ | ✓ | ✓ | ✓ | ✓ | ✓ | ✓ | X | X | X | 16/20 | High |
| Briones et al., 2020 [7] | ✓ | ✓ | ✓ | ✓ | ✓ | ✓ | ✓ | ✓ | ✓ | ✓ | ✓ | ✓ | ✓ | ✓ | ✓ | ✓ | ✓ | ✓ | ✓ | X | 19/20 | High |
| Luni et al., 2017 [8] | ✓ | ✓ | ✓ | X | N | X | X | X | X | X | ✓ | ✓ | N | X | X | X | X | X | ✓ | X | 6/18 | Low |
| van der Nelson et al., 2017 [9] | ✓ | ✓ | ✓ | ✓ | ✓ | ✓ | ✓ | ✓ | ✓ | ✓ | ✓ | ✓ | ✓ | ✓ | ✓ | X | ✓ | ✓ | ✓ | X | 18/20 | High |
| Wohling et al., 2019 [10] | ✓ | ✓ | ✓ | X | N | X | X | X | ✓ | X | ✓ | ✓ | N | ✓ | X | X | ✓ | X | ✓ | X | 9/18 | Moderate |
| Caceda et al., 2018 [11] | X | ✓ | ✓ | ✓ | X | ✓ | ✓ | ✓ | ✓ | ✓ | X | X | X | ✓ | ✓ | ✓ | X | X | ✓ | X | 12/20 | Moderate |
| Henriquez-Trujillo et al., 2017 [12] | ✓ | ✓ | ✓ | ✓ | ✓ | ✓ | ✓ | ✓ | X | ✓ | X | ✓ | ✓ | ✓ | ✓ | ✓ | X | ✓ | ✓ | X | 16/20 | High |
| Voon et al., 2018 [13] | ✓ | ✓ | ✓ | ✓ | ✓ | X | ✓ | ✓ | ✓ | ✓ | X | ✓ | N | ✓ | ✓ | X | ✓ | X | ✓ | ✓ | 15/19 | High |
| Higgins et al., 2011 [14] | ✓ | ✓ | ✓ | X | N | X | X | X | X | X | ✓ | ✓ | N | X | ✓ | X | X | X | ✓ | X | 7/18 | Low |
| Matthijsse et al., 2022 [15] | ✓ | ✓ | ✓ | ✓ | ✓ | ✓ | ✓ | ✓ | ✓ | ✓ | ✓ | ✓ | X | X | ✓ | ✓ | ✓ | ✓ | X | X | 16/20 | High |
| Vlassoff et al., 2016 [16] | ✓ | ✓ | ✓ | ✓ | X | X | ✓ | X | ✓ | ✓ | X | X | N | ✓ | ✓ | X | X | X | ✓ | X | 10/19 | Moderate |
| Diaz et al., 2009 [17] | X | ✓ | ✓ | ✓ | X | X | X | X | X | X | ✓ | ✓ | N | ✓ | X | X | X | X | X | X | 6/19 | Low |
| Tsu et al., 2009 [18] | ✓ | ✓ | ✓ | ✓ | ✓ | X | ✓ | ✓ | ✓ | ✓ | ✓ | ✓ | N | ✓ | ✓ | X | ✓ | ✓ | X | X | 15/19 | High |
| Pichon-Riviere et al., 2015 [19] | ✓ | ✓ | ✓ | ✓ | ✓ | ✓ | ✓ | ✓ | ✓ | ✓ | ✓ | ✓ | ✓ | ✓ | ✓ | ✓ | ✓ | ✓ | ✓ | X | 19/20 | High |
| Carvalho et al., 2020 [20] | ✓ | ✓ | ✓ | ✓ | ✓ | ✓ | ✓ | ✓ | ✓ | ✓ | ✓ | ✓ | N | ✓ | ✓ | ✓ | ✓ | ✓ | X | X | 17/19 | High |
| Sutherland et al., 2009 [21] | ✓ | ✓ | ✓ | ✓ | ✓ | X | X | X | ✓ | ✓ | ✓ | ✓ | N | ✓ | X | X | X | ✓ | X | X | 11/19 | Moderate |
| Sutherland et al., 2010 [22] | ✓ | ✓ | ✓ | ✓ | ✓ | ✓ | ✓ | X | ✓ | ✓ | X | ✓ | ✓ | ✓ | ✓ | X | ✓ | X | X | X | 14/20 | Moderate |
| Goldie et al., 2010 [23] | ✓ | X | ✓ | ✓ | ✓ | ✓ | X | ✓ | ✓ | ✓ | ✓ | ✓ | N | ✓ | ✓ | ✓ | ✓ | X | ✓ | ✓ | 16/19 | High |
| Lubinga et al., 2016 [24] | ✓ | ✓ | ✓ | ✓ | ✓ | ✓ | ✓ | ✓ | ✓ | ✓ | ✓ | ✓ | ✓ | ✓ | ✓ | ✓ | ✓ | X | ✓ | X | 18/20 | High |
| Prata et al., 2010 [25] | ✓ | X | ✓ | ✓ | ✓ | X | ✓ | X | X | X | ✓ | ✓ | N | X | X | X | ✓ | X | X | ✓ | 9/19 | Low |
| Lang et al., 2015 [26] | ✓ | ✓ | ✓ | ✓ | ✓ | X | ✓ | X | ✓ | ✓ | ✓ | ✓ | N | X | X | X | ✓ | X | ✓ | X | 12/19 | Moderate |
| Fullerton et al., 2006 [27] | ✓ | ✓ | ✓ | ✓ | ✓ | X | ✓ | ✓ | ✓ | ✓ | ✓ | ✓ | N | ✓ | X | X | ✓ | ✓ | X | X | 14/19 | Moderate |
| Dazelle et al., 2023 [28] | ✓ | ✓ | ✓ | ✓ | ✓ | ✓ | ✓ | ✓ | ✓ | ✓ | ✓ | ✓ | ✓ | ✓ | ✓ | ✓ | ✓ | ✓ | ✓ | X | 19/20 | High |
| Durand-Zaleski et al., 2020 [29] | ✓ | ✓ | ✓ | ✓ | N | ✓ | ✓ | ✓ | ✓ | ✓ | ✓ | ✓ | N | ✓ | ✓ | ✓ | ✓ | ✓ | ✓ | X | 17/18 | High |
| Sentilhes et al., 2023 [30] | ✓ | ✓ | ✓ | ✓ | N | ✓ | ✓ | ✓ | ✓ | ✓ | ✓ | ✓ | N | ✓ | ✓ | X | ✓ | ✓ | ✓ | X | 16/18 | High |
| Denison et al., 2019 [31,32] | ✓ | ✓ | ✓ | ✓ | N | ✓ | ✓ | ✓ | ✓ | ✓ | ✓ | ✓ | N | X | ✓ | ✓ | ✓ | X | ✓ | X | 15/18 | High |
| Sharma et al., 2023 [33] | ✓ | ✓ | ✓ | ✓ | N | X | X | X | X | X | ✓ | ✓ | N | X | X | X | ✓ | X | ✓ | ✓ | 9/18 | Moderate |
| Hong et al., 2022 [34] | ✓ | ✓ | X | X | N | X | X | X | X | X | ✓ | X | N | X | X | X | X | X | ✓ | X | 4/18 | Low |
| Niola et al., 2017 [35] | X | ✓ | ✓ | X | N | X | X | ✓ | ✓ | X | X | ✓ | N | X | X | X | ✓ | ✓ | ✓ | X | 8/18 | Low |
| Xue et al., 2019 [36] | ✓ | ✓ | ✓ | X | N | X | X | X | X | X | ✓ | ✓ | N | X | X | X | ✓ | X | ✓ | X | 7/18 | Low |
|  |  |  |  |  |  |  |  |  |  |  |  |  |  |  |  |  |  |  |  |  |  |  |
| Yes / Rather yes | ✓ |  |  |  |  |  |  |  |  |  |  |  |  |  |  |  |  |  |  |  |  |  |
| No / Rather no | X |  |  |  |  |  |  |  |  |  |  |  |  |  |  |  |  |  |  |  |  |  |
| Not Applicable | N |  |  |  |  |  |  |  |  |  |  |  |  |  |  |  |  |  |  |  |  |  |
| Unable to be assessed | ? |  |  |  |  |  |  |  |  |  |  |  |  |  |  |  |  |  |  |  |  |  |

Abbreviations: CE: Cost-effectiveness. PPH: Postpartum haemorrhage.

Table B: CHEC-Extended scores for studies assessing diagnostic interventions for PPH

| **Study** | **Patient population** | **Competing alternatives** | **Research question** | **Economic study design** | **Model description** | **Time horizon** | **Study perspective** | **Costs identification** | **Costs measurement** | **Costs valuation** | **Outcomes identification** | **Outcomes measurement** | **Outcomes valuation** | **Incremental CE analysis** | **Discounting** | **Uncertainty analysis** | **Conclusions** | **Generalizability** | **Conflict of interests** | **Ethical issues** | **Score** |  |
| --- | --- | --- | --- | --- | --- | --- | --- | --- | --- | --- | --- | --- | --- | --- | --- | --- | --- | --- | --- | --- | --- | --- |
| Katz et al., 2020 [37] | ✓ | ✓ | X | X | N | ✓ | X | X | X | X | X | ✓ | N | X | ✓ | X | X | ✓ | ✓ | X | 7/18 | Low |
|  |  |  |  |  |  |  |  |  |  |  |  |  |  |  |  |  |  |  |  |  |  |  |
| Yes / Rather yes | ✓ |  |  |  |  |  |  |  |  |  |  |  |  |  |  |  |  |  |  |  |  |  |
| No / Rather no | X |  |  |  |  |  |  |  |  |  |  |  |  |  |  |  |  |  |  |  |  |  |
| Not Applicable | N |  |  |  |  |  |  |  |  |  |  |  |  |  |  |  |  |  |  |  |  |  |
| Unable to be assessed | ? |  |  |  |  |  |  |  |  |  |  |  |  |  |  |  |  |  |  |  |  |  |

Abbreviations: CE: Cost-effectiveness. PPH: Postpartum haemorrhage.

Table C: CHEC-Extended scores for studies assessing treatment interventions for PPH

| **Study** | **Patient population** | **Competing alternatives** | **Research question** | **Economic study design** | **Model description** | **Time horizon** | **Study perspective** | **Costs identification** | **Costs measurement** | **Costs valuation** | **Outcomes identification** | **Outcomes measurement** | **Outcomes valuation** | **Incremental CE analysis** | **Discounting** | **Uncertainty analysis** | **Conclusions** | **Generalizability** | **Conflict of interests** | **Ethical issues** | **Score** |  |
| --- | --- | --- | --- | --- | --- | --- | --- | --- | --- | --- | --- | --- | --- | --- | --- | --- | --- | --- | --- | --- | --- | --- |
| Bradley et al., 2007 [38] | ✓ | ✓ | ✓ | ✓ | ✓ | ✓ | ✓ | ✓ | ✓ | ✓ | ✓ | ✓ | N | ✓ | ✓ | X | ✓ | ✓ | X | X | 16/19 | High |
| Sutherland et al., 2010 [22] | ✓ | ✓ | ✓ | ✓ | ✓ | ✓ | ✓ | X | ✓ | ✓ | X | ✓ | ✓ | ✓ | ✓ | X | ✓ | X | X | X | 14/20 | Moderate |
| Howard et al., 2022 [39] | ✓ | ✓ | ✓ | ✓ | ✓ | ✓ | ✓ | X | ✓ | ✓ | ✓ | ✓ | ✓ | ✓ | ✓ | ✓ | ✓ | ✓ | ✓ | X | 18/20 | High |
| Sudhof et al., 2019 [40] | X | ✓ | ✓ | ✓ | ✓ | ✓ | ✓ | ✓ | ✓ | ✓ | ✓ | ✓ | ✓ | ✓ | ✓ | ✓ | ✓ | ✓ | ✓ | X | 18/20 | High |
| Joshi et al., 2023 [41] | ✓ | ✓ | ✓ | ✓ | ✓ | ✓ | ✓ | ✓ | ✓ | ✓ | ✓ | ✓ | ✓ | ✓ | ✓ | ✓ | ✓ | ✓ | ✓ | ✓ | 20/20 | High |
| Li et al., 2018 [42] | ✓ | ✓ | ✓ | ✓ | ✓ | ✓ | ✓ | ✓ | ✓ | ✓ | ✓ | ✓ | ✓ | ✓ | ✓ | ✓ | ✓ | ✓ | ✓ | X | 19/20 | High |
| Downing et al., 2015 [43] | ✓ | ✓ | ✓ | ✓ | ✓ | ✓ | X | ✓ | ✓ | X | ✓ | ✓ | X | ✓ | X | X | ✓ | X | ✓ | X | 13/20 | Moderate |
| Sutherland et al., 2013 [44] | ✓ | ✓ | ✓ | ✓ | X | X | X | ✓ | ✓ | ✓ | ✓ | ✓ | ✓ | ✓ | ✓ | X | ✓ | ✓ | ✓ | X | 15/20 | High |
| Mvundura et al., 2017 [45] | ✓ | ✓ | ✓ | ✓ | ✓ | X | ✓ | X | ✓ | ✓ | ✓ | X | ✓ | ✓ | X | X | ✓ | X | ✓ | X | 13/20 | Moderate |
| Joshi et al., 2021 [46] | ✓ | ✓ | ✓ | ✓ | ✓ | ✓ | ✓ | ✓ | ✓ | ✓ | ✓ | ✓ | ✓ | ✓ | ✓ | ✓ | ✓ | ✓ | ✓ | X | 19/20 | High |
| Edwards et al., 2023 [47] | ✓ | ✓ | ✓ | ✓ | ✓ | X | ✓ | ✓ | ✓ | ✓ | ✓ | ✓ | N | ✓ | ✓ | X | X | ✓ | ✓ | X | 15/19 | High |
| Snegovskikh et al., 2017 [48] | ✓ | ✓ | ✓ | ✓ | N | X | X | ✓ | X | X | ✓ | ✓ | N | X | X | X | ✓ | X | X | X | 8/18 | Low |
| Einerson et al., 2017 [49] | ✓ | ✓ | ✓ | ✓ | ✓ | ✓ | ✓ | X | ✓ | ✓ | X | ✓ | N | ✓ | ✓ | ✓ | ✓ | X | ✓ | X | 15/19 | High |
| Prick et al., 2014 [50] | ✓ | ✓ | ✓ | ✓ | N | ✓ | ✓ | ✓ | ✓ | ✓ | ✓ | ✓ | ✓ | ✓ | ✓ | ✓ | ✓ | X | ✓ | X | 17/19 | High |
| Khan et al., 2018 [51] | ✓ | ✓ | ✓ | ✓ | ✓ | X | ✓ | ✓ | ✓ | ✓ | ✓ | ✓ | N | ✓ | ✓ | ✓ | ✓ | ✓ | ✓ | ✓ | 18/19 | High |
| Lim et al., 2018 [52] | ✓ | ✓ | ✓ | ✓ | ✓ | ✓ | ✓ | X | ✓ | ✓ | ✓ | ✓ | ✓ | ✓ | ✓ | ✓ | ✓ | ✓ | ✓ | X | 18/20 | High |
| Ries et al., 2020 [53] | ✓ | ✓ | ✓ | ✓ | N | X | X | X | X | X | ✓ | ✓ | N | X | X | X | ✓ | X | ✓ | X | 8/18 | Low |
| Franke et al., 2024 [54] | ✓ | ✓ | ✓ | ✓ | ✓ | ✓ | ✓ | ✓ | ✓ | ✓ | ✓ | X | N | ✓ | ✓ | X | ✓ | ✓ | ✓ | ✓ | 17/19 | High |
|  |  |  |  |  |  |  |  |  |  |  |  |  |  |  |  |  |  |  |  |  |  |  |
| Yes / Rather yes | ✓ |  |  |  |  |  |  |  |  |  |  |  |  |  |  |  |  |  |  |  |  |  |
| No / Rather no | X |  |  |  |  |  |  |  |  |  |  |  |  |  |  |  |  |  |  |  |  |  |
| Not Applicable | N |  |  |  |  |  |  |  |  |  |  |  |  |  |  |  |  |  |  |  |  |  |
| Unable to be assessed | ? |  |  |  |  |  |  |  |  |  |  |  |  |  |  |  |  |  |  |  |  |  |

Abbreviations: CE: Cost-effectiveness. PPH: Postpartum haemorrhage

Table D: CHEC-Extended scores for studies assessing bundles for PPH prevention, diagnosis, and treatment

| **Study** | **Patient population** | **Competing alternatives** | **Research question** | **Economic study design** | **Model description** | **Time horizon** | **Study perspective** | **Costs identification** | **Costs measurement** | **Costs valuation** | **Outcomes identification** | **Outcomes measurement** | **Outcomes valuation** | **Incremental CE analysis** | **Discounting** | **Uncertainty analysis** | **Conclusions** | **Generalizability** | **Conflict of interests** | **Ethical issues** | **Score** |  |
| --- | --- | --- | --- | --- | --- | --- | --- | --- | --- | --- | --- | --- | --- | --- | --- | --- | --- | --- | --- | --- | --- | --- |
| Seim et al., 2023 [55] | X | ✓ | X | X | N | X | X | X | ✓ | X | ✓ | X | X | ✓ | X | X | X | X | ✓ | X | 5/19 | Low |
| Wiesehan et al., 2023 [56] | ✓ | ✓ | ✓ | ✓ | ✓ | ✓ | ✓ | ✓ | ✓ | ✓ | ✓ | ✓ | ✓ | ✓ | ✓ | ✓ | ✓ | ✓ | ✓ | X | 19/20 | High |
| Dale et al., 2022 [57] | X | ✓ | ✓ | ✓ | ✓ | ✓ | ✓ | X | ✓ | ✓ | ✓ | ✓ | N | X | ✓ | ✓ | ✓ | X | ✓ | X | 14/19 | Moderate |
| Williams et al., 2024 [58] | ✓ | ✓ | ✓ | ✓ | N | ✓ | ✓ | ✓ | ✓ | ✓ | ✓ | ✓ | ✓ | ✓ | ✓ | ✓ | ✓ | ✓ | ✓ | X | 18/19 | High |
|  |  |  |  |  |  |  |  |  |  |  |  |  |  |  |  |  |  |  |  |  |  |  |
| Yes / Rather yes | ✓ |  |  |  |  |  |  |  |  |  |  |  |  |  |  |  |  |  |  |  |  |  |
| No / Rather no | X |  |  |  |  |  |  |  |  |  |  |  |  |  |  |  |  |  |  |  |  |  |
| Not Applicable | N |  |  |  |  |  |  |  |  |  |  |  |  |  |  |  |  |  |  |  |  |  |
| Unable to be assessed | ? |  |  |  |  |  |  |  |  |  |  |  |  |  |  |  |  |  |  |  |  |  |

Abbreviations: CE: Cost-effectiveness. PPH: Postpartum haemorrhage.

# **References**

1. Pickering K, Gallos ID, Williams H, Price MJ, Merriel A, Lissauer D, et al. Uterotonic drugs for the prevention of postpartum haemorrhage: a cost-effectiveness analysis. Pharmacoecon Open. 2019;3:163–76. doi: 10.1007/s41669-018-0108-x.

2. Gallos I, Williams H, Price M, Pickering K, Merriel A, Tobias A, et al. Uterotonic drugs to prevent postpartum haemorrhage: a network meta-analysis. Health Technol Assess. 2019;23(9). doi: 10.3310/hta23090.

3. Barrett J, Ko S, Jeffery W. Cost implications of using carbetocin injection to prevent postpartum hemorrhage in a Canadian urban Hospital. J Obstet Gynaecol Can. 2022;44(3):272–8. doi: 10.1016/j.jogc.2021.09.022.

4. Cook JR, Saxena K, Taylor C, Jacobs JL. Cost-effectiveness and budget impact of heat-stable carbetocin compared to oxytocin and misoprostol for the prevention of postpartum hemorrhage (PPH) in women giving birth in India. BMC Health Serv Res. 2023;23(1):267. doi: 10.1186/s12913-023-09263-4.

5. You JH, Leung T-y. Cost-effectiveness analysis of carbetocin for prevention of postpartum hemorrhage in a low-burden high-resource city of China. PLoS One. 2022;17(12):e0279130. doi: 10.1371/journal.pone.0279130.

6. Gil-Rojas Y, Lasalvia P, Hernández F, Castañeda-Cardona C, Rosselli D. Cost-effectiveness of Carbetocin versus Oxytocin for Prevention of Postpartum Hemorrhage Resulting from Uterine Atony in Women at high-risk for bleeding in Colombia. Rev Bras Ginecol Obstet. 2018;40:242–50. doi: 10.1055/s-0038-1655747.

7. Briones JR, Talungchit P, Thavorncharoensap M, Chaikledkaew U. Economic evaluation of carbetocin as prophylaxis for postpartum hemorrhage in the Philippines. BMC Health Serv Res. 2020;20:1–12. doi: 10.1186/s12913-020-05834-x.

8. Luni Y, Borakati A, Matah A, Skeats K, Eedarapalli P. A prospective cohort study evaluating the cost-effectiveness of carbetocin for prevention of postpartum haemorrhage in caesarean sections. J Obstet Gynaecol Can. 2017;37(5):601–4. doi: 10.1080/01443615.2017.1284188.

9. Van Der Nelson HA, Draycott T, Siassakos D, Yau CW, Hatswell AJ. Carbetocin versus oxytocin for prevention of post-partum haemorrhage at caesarean section in the United Kingdom: an economic impact analysis. Eur J Obstet Gynecol Reprod Biol. 2017;210:286-91. doi: 10.1016/j.ejogrb.2017.01.004.

10. Wohling J, Edge N, Pena‐Leal D, Wang R, Mol BW, Dekker G. Clinical and financial evaluation of carbetocin as postpartum haemorrhage prophylaxis at caesarean section: A retrospective cohort study. Aust N Z J Obstet Gynaecol. 2019;59(4):501–7. doi: 10.1111/ajo.12907.

11. Caceda SI, Ramos RR, Saborido CM. Pharmacoeconomic study comparing carbetocin with oxytocin for the prevention of hemorrhage following cesarean delivery in Lima, Peru. J Comp Eff Res. 2018;7(1):49-55. doi: 10.2217/cer-2017-0012.

12. Henríquez-Trujillo AR, Lucio-Romero RA, Bermúdez-Gallegos K. Analysis of the cost–effectiveness of carbetocin for the prevention of hemorrhage following cesarean delivery in Ecuador. J Comp Eff Res. 2017;6(6):529–36. doi: 10.2217/cer-2017-0004.

13. Voon HY, Shafie AA, Bujang MA, Suharjono HN. Cost effectiveness analysis of carbetocin during cesarean section in a high volume maternity unit. J Obstet Gynaecol Res. 2018;44(1):109–16. doi: 10.1111/jog.13486.

14. Higgins L, Mechery J, Tomlinson A. Does carbetocin for prevention of postpartum haemorrhage at caesarean section provide clinical or financial benefit compared with oxytocin? J Obstet Gynaecol. 2011;31(8):732–9. doi: 10.3109/01443615.2011.595982.

15. Matthijsse S, Andersson FL, Gargano M, Yip Sonderegger YL. Cost-effectiveness analysis of carbetocin versus oxytocin for the prevention of postpartum hemorrhage following vaginal birth in the United Kingdom. J Med Econ. 2022;25(1):129–37. doi: 10.1080/13696998.2022.2027669.

16. Vlassoff M, Diallo A, Philbin J, Kost K, Bankole A. Cost-effectiveness of two interventions for the prevention of postpartum hemorrhage in Senegal. Int J Gynaecol Obstet. 2016;133(3):307–11. doi: 10.1016/j.ijgo.2015.10.015.

17. Jose Diaz J, Jaramillo M. Evaluating interventions to reduce maternal mortality: evidence from Peru's PARSalud programme. J Dev Effect. 2009;1(4):387–412. doi: 10.1080/19439340903380872.

18. Tsu VD, Levin C, Tran MP, Hoang MV, Luu HT. Cost-effectiveness analysis of active management of third-stage labour in Vietnam. Health Policy Plan. 2009;24(6):438–44. doi: 10.1093/heapol/czp020.

19. Pichon-Riviere A, Glujovsky D, Garay OU, Augustovski F, Ciapponi A, Serpa M, et al. Oxytocin in uniject disposable auto-disable injection system versus standard use for the prevention of postpartum hemorrhage in latin America and the Caribbean: a cost-effectiveness analysis. PLoS One. 2015;10(6):e0129044. doi: 10.1371/journal.pone.0129044.

20. Carvalho N, Hoque ME, Oliver VL, Byrne A, Kermode M, Lambert P, et al. Cost-effectiveness of inhaled oxytocin for prevention of postpartum haemorrhage: a modelling study applied to two high burden settings. BMC Med. 2020;18(1):1–18. doi: 10.1186/s12916-020-01658-y.

21. Sutherland T, Bishai DM. Cost-effectiveness of misoprostol and prenatal iron supplementation as maternal mortality interventions in home births in rural India. Int J Gynaecol Obstet. 2009;104(3):189–93. doi: 10.1016/j.ijgo.2008.10.011.

22. Sutherland T, Meyer C, Bishai DM, Geller S, Miller S. Community-based distribution of misoprostol for treatment or prevention of postpartum hemorrhage: cost-effectiveness, mortality, and morbidity reduction analysis. Int J Gynaecol Obstet. 2010;108(3):289–94. doi: 10.1016/j.ijgo.2009.11.007.

23. Goldie SJ, Sweet S, Carvalho N, Natchu UCM, Hu D. Alternative strategies to reduce maternal mortality in India: a cost-effectiveness analysis. PLoS Med. 2010;7(4):e1000264. doi: 10.1371/journal.pmed.1000264.

24. Lubinga SJ, Atukunda EC, Wasswa-Ssalongo G, Babigumira JB. Potential cost-effectiveness of prenatal distribution of misoprostol for prevention of postpartum hemorrhage in Uganda. PLoS One. 2015;10(11):e0142550. doi: 10.1371/journal.pone.0142550.

25. Prata N, Sreenivas A, Greig F, Walsh J, Potts M. Setting priorities for safe motherhood interventions in resource-scarce settings. Health Policy. 2010;94(1):1–13. doi: 10.1016/j.healthpol.2009.08.012.

26. Lang DL, Zhao F-L, Robertson J. Prevention of postpartum haemorrhage: cost consequences analysis of misoprostol in low-resource settings. BMC Pregnancy Childbirth. 2015;15(1):1–9. doi: 10.1186/s12884-015-0749-z.

27. Fullerton JT, Frick KD, Fogarty LA, Fishel JD, Vivio DM. Active management of third stage of labour saves facility costs in Guatemala and Zambia. J Health Popul Nutr. 2006;24(4):540.

28. Dazelle WD, Ebner MK, Kazma J, Potarazu SN, Ahmadzia HK. Tranexamic acid for the prevention of postpartum hemorrhage: a cost-effectiveness analysis. J Thromb Thrombolysis. 2023:1–9. doi: 10.1007/s11239-023-02814-w.

29. Durand‐Zaleski I, Deneux‐Tharaux C, Seco A, Malki M, Frenkiel J, Sentilhes L, et al. An economic evaluation of tranexamic acid to prevent postpartum haemorrhage in women with vaginal delivery: the randomised controlled TRAAP trial. BJOG. 2021;128(1):114–20. doi: 10.1111/1471-0528.16456.

30. Sentilhes L, Bénard A, Madar H, Froeliger A, Petit S, Deneux-Tharaux C. Tranexamic acid for reduction of blood loss after Caesarean delivery: a cost-effectiveness analysis of the TRAAP2 trial. Br J Anaesth. 2023;131(5):893-900. doi: 10.1016/j.bja.2023.07.028.

31. Denison FC, Carruthers KF, Hudson J, McPherson G, Chua GN, Peace M, et al. Nitroglycerin for treatment of retained placenta: A randomised, placebo-controlled, multicentre, double-blind trial in the UK. PLoS Med. 2019;16(12):e1003001. doi: 10.1371/journal.pmed.1003001.

32. Denison FC, Carruthers KF, Hudson J, McPherson G, Scotland G, Brook-Smith S, et al. Glyceryl trinitrate to reduce the need for manual removal of retained placenta following vaginal delivery: the GOT-IT RCT. Health Technol Assess. 2019;23(70):1–72. doi: 10.3310/hta23700.

33. Sharma JC, Kollabathula P, Jindal S, Anupma A, Sarkar A, Jaggarwal S, et al. Application of a Negative Intrauterine Pressure Suction Device for Prophylactic Management of Atonic Postpartum Hemorrhage: A Quality Improvement Study. Cureus. 2023;15(7):e42631. doi: 10.7759/cureus.42631.

34. Hong L, Chen A, Chen J, Li X, Zhuang W, Shen Y, et al. The clinical evaluation of IIA balloon occlusion in caesarean delivery for patients with PAS: a retrospective study. BMC Pregnancy Childbirth. 2022;22(1):103. doi: 10.1186/s12884-022-04434-3.

35. Niola R, Giurazza F, Torbica A, Schena E, Silvestre M, Maglione F. Predelivery uterine arteries embolization in patients with placental implant anomalies: a cost-effective procedure. Radiol Med. 2017;122:77–9. doi: 10.1007/s11547-016-0690-x.

36. Xue L, Zhang J, Shen H, Hou Y, Ai L, Cui X. The application of rapid rehabilitation model of multidisciplinary cooperation in cesarean section and the evaluation of health economics. Zhonghua Yi Xue Za Zhi. 2019;99(42):3335–9. doi: 10.3760/cma.j.issn.0376-2491.2019.42.012.

37. Katz D, Wang R, O'Neil L, Gerber C, Lankford A, Rogers T, et al. The association between the introduction of quantitative assessment of postpartum blood loss and institutional changes in clinical practice: an observational study. Int J Obstet Anesth. 2020;42:4–10. doi: 10.1016/j.ijoa.2019.05.006.

38. Bradley SE, Prata N, Young-Lin N, Bishai D. Cost-effectiveness of misoprostol to control postpartum hemorrhage in low-resource settings. Int J Gynaecol Obstet. 2007;97(1):52–6. doi: 10.1016/j.ijgo.2006.12.005.

39. Howard DC, Jones AE, Skeith A, Lai J, D'Souza R, Caughey AB. Tranexamic acid for the treatment of postpartum hemorrhage: a cost-effectiveness analysis. Am J Obstet Gynecol MFM. 2022;4(3):100588. doi: 10.1016/j.ajogmf.2022.100588.

40. Sudhof LS, Shainker SA, Einerson BD. Tranexamic acid in the routine treatment of postpartum hemorrhage in the United States: a cost-effectiveness analysis. Am J Obstet Gynecol. 2019;221(3):275. e1–. e12. doi: 10.1016/j.ajog.2019.06.030.

41. Joshi BN, Shetty SS, Moray KV, Chaurasia H, Sachin O. Cost-effectiveness and budget impact of adding tranexamic acid for management of post-partum hemorrhage in the Indian public health system. BMC Pregnancy Childbirth. 2023;23(1):9. doi: 10.1186/s12884-022-05308-4.

42. Li B, Miners A, Shakur H, Roberts I. Tranexamic acid for treatment of women with post-partum haemorrhage in Nigeria and Pakistan: a cost-effectiveness analysis of data from the WOMAN trial. Lancet Glob Health. 2018;6(2):e222–e8. doi: 10.1016/S2214-109X(17)30467-9.

43. Downing J, El Ayadi A, Miller S, Butrick E, Mkumba G, Magwali T, et al. Cost-effectiveness of the non-pneumatic anti-shock garment (NASG): evidence from a cluster randomized controlled trial in Zambia and Zimbabwe. BMC Health Serv Res. 2015;15(1):1–10. doi: 10.1186/s12913-015-0694-6.

44. Sutherland T, Downing J, Miller S, Bishai DM, Butrick E, Fathalla MM, et al. Use of the non-pneumatic anti-shock garment (NASG) for life-threatening obstetric hemorrhage: a cost-effectiveness analysis in Egypt and Nigeria. PloS One. 2013;8(4):e62282. doi: 10.1371/journal.pone.0062282.

45. Mvundura M, Kokonya D, Abu‐Haydar E, Okoth E, Herrick T, Mukabi J, et al. Cost‐effectiveness of condom uterine balloon tamponade to control severe postpartum hemorrhage in Kenya. Int J Gynaecol Obstet. 2017;137(2):185–91. doi: 10.1002/ijgo.12125.

46. Joshi BN, Shetty SS, Moray KV, Sachin O, Chaurasia H. Cost-effectiveness of uterine balloon tamponade devices in managing atonic post-partum hemorrhage at public health facilities in India. PLoS One. 2021;16(8):e0256271. doi: 10.1371/journal.pone.0256271.

47. Edwards RT, Ezeofor V, Bryning L, Anthony BF, Charles JM, Weeks A. Prevention of postpartum haemorrhage: Economic evaluation of the novel butterfly device in a UK setting. Eur J Obstet Gynecol Reprod Biol. 2023;283:149–57. doi: 10.1016/j.ejogrb.2023.02.020.

48. Snegovskikh D, Souza D, Walton Z, Dai F, Rachler R, Garay A, et al. Point-of-care viscoelastic testing improves the outcome of pregnancies complicated by severe postpartum hemorrhage. J Clin Anesth. 2018;44:50–6. doi: 10.1016/j.jclinane.2017.10.003.

49. Einerson BD, Stehlikova Z, Nelson RE, Bellows BK, Kawamoto K, Clark EA. Transfusion preparedness strategies for obstetric hemorrhage: a cost-effectiveness analysis. Obstet Gynecol. 2017;130(6):1347–55. doi: 10.1097/AOG.0000000000002359.

50. Prick B, Duvekot J, Van Der Moer P, van Gemund N, Van Der Salm P, Jansen A, et al. Cost‐effectiveness of red blood cell transfusion vs. non‐intervention in women with acute anaemia after postpartum haemorrhage. Vox Sang. 2014;107(4):381–8. doi: 10.1111/vox.12181.

51. Khan KS, Moore P, Wilson M, Hooper R, Allard S, Wrench I, et al. A randomised controlled trial and economic evaluation of intraoperative cell salvage during caesarean section in women at risk of haemorrhage: the SALVO (cell SALVage in Obstetrics) trial. Health Technol Assess. 2018;22(2):1–88. doi: 10.3310/hta22020.

52. Lim G, Melnyk V, Facco FL, Waters JH, Smith KJ. Cost-effectiveness analysis of intraoperative cell salvage for obstetric hemorrhage. Anesthesiology. 2018;128(2):328–37. doi: 10.1097/ALN.0000000000001981.

53. Ries J-J, Jeker L, Neuhaus M, Vogt DR, Girard T, Hoesli I. Implementation of the D-A-CH postpartum haemorrhage algorithm after severe postpartum bleeding accelerates clinical management: A retrospective case series. Eur J Obstet Gynecol Reprod Biol. 2020;247:225–31. doi: 10.1016/j.ejogrb.2020.01.001.

54. Franke MA, Nordmann K, Frühauf A, Ranaivoson RM, Rebaliha M, Rapanjato Z, et al. Inter-facility transfers for emergency obstetrical and neonatal care in rural Madagascar: a cost-effectiveness analysis. BMJ Open. 2024;14(4):e081482. doi: 10.1136/bmjopen-2023-081482.

55. Seim AR, Alassoum Z, Souley I, Bronzan R, Mounkaila A, Ahmed LA. The effects of a peripartum strategy to prevent and treat primary postpartum haemorrhage at health facilities in Niger: a longitudinal, 72-month study. Lancet Glob Health. 2023;11(2):e287–e95. doi: 10.1016/S2214-109X(22)00518-6.

56. Wiesehan EC, Keesara SR, Krissberg JR, Main EK, Goldhaber-Fiebert JD. State perinatal quality collaborative for reducing severe maternal morbidity from hemorrhage: a cost-effectiveness analysis. Obstet Gynecol. 2023;141(2):387–94. doi: 10.1097/AOG.0000000000005060.

57. Dale M, Bell SF, O’Connell S, Scarr C, James K, John M, et al. What is the economic cost of providing an all Wales postpartum haemorrhage quality improvement initiative (OBS Cymru)? A cost-consequences comparison with standard care. Pharmacoecon Open. 2022;6(6):847–57. doi: 10.1007/s41669-022-00362-2

58. Williams EV, Goranitis I, Oppong R, Perry SJ, Devall AJ, Martin JT, et al. A cost-effectiveness analysis of early detection and bundled treatment of postpartum hemorrhage alongside the E-MOTIVE trial. Nat Med. 2024. doi: 10.1038/s41591-024-03069-5.
