# Supplementary material for: The cost-effectiveness of preventing, diagnosing, and treating postpartum haemorrhage: A systematic review of economic evaluations
Source: PLoS Med. 2024 Sep 13;21(9):e1004461. doi: 10.1371/journal.pmed.1004461 (PMC11433145; doi:10.1371/journal.pmed.1004461)
Supplement: S8 Appendix — (DOCX) [file pmed.1004461.s008.docx]

**S8 Appendix: Analysis of effectiveness data used in included studies**

Cost-effectiveness analyses are highly dependent on the underlying effectiveness data used in their calculations. In assessing studies included in this review, we observed instances where the effectiveness estimate used in the cost-effectiveness calculation were not consistent with latest evidence – for example, a cost-effectiveness analysis assumed an intervention could significantly reduce a health outcome, when more recent evidence from a systematic review indicated there was likely no effect.

For all included studies, we aimed to systematically identify the effectiveness estimates used in economic calculations and compare this to best available effectiveness evidence. In doing so, we aimed to identify any studies that may have drawn cost-effectiveness conclusions based on superseded effectiveness data. For each study, we extracted the main effectiveness parameters used. We also collated and compared the effectiveness estimates used in the corresponding WHO recommendation, as well as systematic reviews, for that intervention.

The following sections addressing PPH prevention, diagnosis, treatment, and bundles are structured as follows:

- Current WHO recommendations on topic.
- A brief discussion on the effectiveness evidence and sources identified.
- Key effectiveness estimates extracted from systematic reviews (usually as a risk ratio or odds ratio) and 95% confidence intervals presented in tables.
- A comparison of the effectiveness data used in each included study, with the effectiveness data identified in key systematic reviews.

## **Prevention of PPH**

**Current WHO Recommendations on the prevention of PPH**

- Administration of an effective uterotonic after birth in women following vaginal birth or caesarean section [1]. Oxytocin (10 IU, IM/IV) is the agent of choice when multiple uterotonic options are available [2].
- In settings where oxytocin is unavailable (or its quality cannot be guaranteed), the use of other injectable uterotonics (carbetocin, or if appropriate ergometrine/methylergometrine, or oxytocin and ergometrine fixed-dose combination) or oral misoprostol is recommended [1].
- In settings where skilled health personnel are not present to administer injectable uterotonics, the administration of misoprostol (400 μg or 600 μg, PO) by community health workers and lay health workers is recommended for the prevention of PPH [1].
- Controlled cord traction by skilled health professionals for vaginal births [3].
- Controlled cord traction for removal of the placenta in caesarean section [3].
- Late cord clamping (performed after 1 to 3 minutes after birth) for all births (early cord clamping is not recommended, unless the neonate is asphyxiated and needs to move immediately for resuscitation) [3].
- In settings where women give birth outside of a health facility and in the absence of skilled health personnel, a strategy of antenatal distribution of misoprostol to pregnant women for self-administration is recommended for prevention of postpartum haemorrhage, only with targeted monitoring and evaluation [4].

**Effectiveness Evidence for preventative interventions included in this review**

Uterotonics for the prevention of PPH

- One Cochrane review on the effectiveness and comparative effectiveness of numerous uterotonics for the prevention of PPH was published in 2018: Gallos et al., 2018 [5].
- The same analysis was republished as part of a UK Health Technology Assessment in 2019 [6].
- As some of the figures differed slightly between the publications, and depending on whether the risk ratio results were taken from the direct or NMA analysis, both are included here for completeness.
- Other reviews have been published focusing on single uterotonic agents:
  - Oxytocin: Salati et al., 2019 [7]
  - Carbetocin: Kalafat et al., 2021 [8]
- Effectiveness estimates from these reviews are presented below:

Table A: Effectiveness estimates from systematic reviews - Carbetocin vs Oxytocin in mixed delivery

| **Outcome** | **RR (95% CI)** | **Source** |
| --- | --- | --- |
| RR of PPH >500ml | 0.75 (0.58 - 0.98) | Gallos et al., 2018 [5]  (Direct Analysis) |
|  | 0.72 (0.52 - 1.00) | Gallos et al., 2019 [6]  (NMA Analysis) |
| RR of PPH >1000ml | 0.73 (0.45 - 1.19) | Gallos et al., 2018 [5]  (Direct Analysis) |
|  | 0.70 (0.38 - 1.28) | Gallos et al., 2019 [6]  (NMA Analysis) |
| RR of requiring additional uterotonics | 0.48 (0.34 - 0.68) | Gallos et al., 2018 [5]  (Direct Analysis) |
|  | 0.51 (0.37 - 0.71) | Gallos et al., 2019 [6]  (NMA Analysis) |
| RR of requiring transfusion | 0.68 (0.38 - 1.22) | Gallos et al., 2018 [5]  (Direct Analysis) |
|  | 0.64 (0.33 - 1.25) | Gallos et al., 2019 [6]  (NMA Analysis) |

Abbreviations: CI: Confidence Interval. NMA: Network meta-analysis. PPH: Postpartum haemorrhage. RR: relative risk

Table B: Effectiveness estimates from systematic reviews - Carbetocin vs Oxytocin - Vaginal Birth

| **Outcome** | **RR (95% CI)** | **Source** |
| --- | --- | --- |
| RR of PPH >500ml | 0.67 (0.34 - 1.3) | Gallos et al., 2018 [5]  (Direct Analysis) |
|  | 0.61 (0.34 - 1.07) | Gallos et al., 2019 [6]  (NMA Analysis) |
|  | 0.44 (0.14 - 1.38) | Kalafat et al., 2021 [8] |
| RR of PPH >1000ml | 0.68 (0.21 - 2.2) | Gallos et al., 2018 [5]  (Direct Analysis) |
|  | 0.53 (0.12 - 2.34) | Gallos et al., 2019 [6]  (NMA Analysis) |
| RR of requiring additional uterotonics | 0.54 (0.3 - 0.99) | Gallos et al., 2018 [5]  (Direct Analysis) |
|  | 0.52 (0.31 - 0.90) | Kalafat et al., 2021 [8] |
| RR of requiring transfusion | 1.04 (0.52 - 2.1) | Gallos et al., 2018 [5]  (Direct Analysis) |
|  | 0.67 (0.09 - 4.80) | Kalafat et al., 2021 [8] |

Abbreviations: CI: Confidence Interval. NMA: Network meta-analysis. PPH: Postpartum haemorrhage. RR: relative risk

Table C: Effectiveness estimates from systematic reviews - Carbetocin vs Oxytocin - Caesarean Section

| **Outcome** | **RR (95% CI)** | **Source** |
| --- | --- | --- |
| RR of PPH >500ml | 0.71 (0.47 - 1.07) | Gallos et al., 2018 [5]  (Direct Analysis) |
|  | 0.77 (0.52 - 1.14) | Gallos et al., 2019 [6]  (NMA Analysis) |
| RR of PPH >1000ml | 0.62 (0.31 - 1.23) | Gallos et al., 2018 [5]  (Direct Analysis) |
|  | 0.73 (0.38 - 1.41) | Gallos et al., 2019 [6]  (NMA Analysis) |
|  | 0.83 (0.55 - 1.28) | Kalafat et al., 2021 [8] |
| RR of requiring additional uterotonics | 0.45 (0.27 - 0.74) | Gallos et al., 2018 [5]  (Direct Analysis) |
|  | 0.43 (0.30 - 0.59) | Kalafat et al., 2021 [8] |
| RR of requiring transfusion | 0.5 (0.23 - 1.1) | Gallos et al., 2018 [5]  (Direct Analysis) |
|  | 0.57 (0.33 - 0.96) | Kalafat et al., 2021 [8] |

Abbreviations: CI: Confidence Interval. NMA: Network meta-analysis. PPH: Postpartum haemorrhage. RR: relative risk

Table D: Effectiveness estimates from systematic reviews - Misoprostol vs Placebo - Mixed Delivery

| **Medication Strategy** | **RR (95% CI)** | **Source** |
| --- | --- | --- |
| RR of PPH >500ml | 0.75 (0.59 - 0.94) | Gallos et al., 2018 [5] (Direct Analysis) |
|  | 0.61 (0.50 - 0.74) | Gallos et al., 2019 [6] (NMA Analysis) |
| RR of PPH >1000ml | 0.73 (0.56 - 0.95) | Gallos et al., 2018 [5] (Direct Analysis) |
|  | 0.73 (0.61 - 0.88) | Gallos et al., 2019 [6] (NMA Analysis) |
| RR of requiring additional uterotonics | 0.67 (0.52 - 0.87) | Gallos et al., 2018 [5] (Direct Analysis) |
|  | 0.43 (0.31 - 0.59) | Gallos et al., 2019 [6] (NMA Analysis) |
| RR of requiring transfusion | 0.46 (0.15 - 1.47) | Gallos et al., 2018 [5] (Direct Analysis) |
|  | 0.49 (0.32 - 0.75) | Gallos et al., 2019 [6] (NMA Analysis) |
| Maternal Death | 1 (0.1 - 9.59) | Gallos et al., 2018 [5] (Direct Analysis) |
|  | 0.98 (0.23 - 4.12) | Gallos et al., 2019 [6] (NMA analysis) |

 Abbreviations: CI: Confidence Interval. NMA: Network meta-analysis. PPH: Postpartum haemorrhage. RR: relative risk

Table E: Effectiveness estimates from systematic reviews - Misoprostol vs Placebo - Vaginal Birth

| **Medication Strategy** | **RR (95% CI)** | **Source** |
| --- | --- | --- |
| RR of PPH >500ml | 0.75 (0.59 - 0.94) | Gallos et al., 2018 [5] (Direct Analysis) |
|  | 0.60 (0.48 - 0.75) | Gallos et al., 2019 [6] (NMA analysis) |
| RR of PPH >1000ml | 0.73 (0.56 - 0.95) | Gallos et al., 2018 [5] (Direct Analysis) |
|  | 0.76 (0.64 - 0.89) | Gallos et al., 2019 [6] (NMA analysis) |

 Abbreviations: CI: Confidence Interval. NMA: Network meta-analysis. PPH: Postpartum haemorrhage. RR: relative risk

Table F: Effectiveness estimates from systematic reviews - Misoprostol vs Placebo - Caesarean Section

| **Medication Strategy** | **RR (95% CI)** | **Source** |
| --- | --- | --- |
| RR of PPH >500ml | 0.75 (0.29 - 1.95) | Gallos et al., 2019 [6] (NMA analysis) |
| RR of PPH >1000ml | 0.86 (0.02 - 25.86) | Gallos et al., 2019 [6] (NMA analysis) |

Abbreviations: CI: Confidence Interval. NMA: Network meta-analysis. PPH: Postpartum haemorrhage. RR: relative risk

Table G: Effectiveness estimates from systematic reviews - Misoprostol vs Oxytocin - Mixed Delivery

| **Medication Strategy** | **RR (95% CI)** | **Source** |
| --- | --- | --- |
| RR of PPH >500ml | 1.08 (0.94 - 1.24) | Gallos et al., 2018 [5] (Direct Analysis) |
|  | 1.08 (0.95 - 1.23) | Gallos et al., 2019 [6] (NMA analysis) |
| RR of PPH >1000ml | 1.26 (1.11 - 1.43) | Gallos et al., 2018 [5] (Direct Analysis) |
|  | 1.21 (1.01 - 1.44) | Gallos et al., 2019 [6] (NMA analysis) |
| RR of requiring additional uterotonics | 1.01 (0.85 - 1.2) | Gallos et al., 2018 [5] (Direct Analysis) |
|  | 1.07 (0.89 - 1.29) | Gallos et al., 2019 [6] (NMA analysis) |
| RR of requiring blood transfusion | 0.81 (0.65 - 1.0) | Gallos et al., 2018 [5] (Direct Analysis) |
|  | 0.89 (0.69 - 1.16) | Gallos et al., 2019 [6] (NMA analysis) |

Abbreviations: CI: Confidence Interval. NMA: Network meta-analysis. PPH: Postpartum haemorrhage. RR: relative risk

Table H: Effectiveness estimates from systematic reviews - Misoprostol vs Oxytocin - Vaginal Birth

| **Medication Strategy** | **RR (95% CI)** | **Source** |
| --- | --- | --- |
| RR of PPH >500ml | 1.08 (0.9 - 1.31) | Gallos et al., 2018 [5] (Direct Analysis) |
|  | 1.09 (0.94 - 1.27) | Gallos et al., 2019 [6] (NMA analysis) |
| RR of PPH >1000ml | 1.31 (1.15 - 1.49) | Gallos et al., 2018 [5] (Direct Analysis) |
|  | 1.28 (1.14 - 1.44) | Gallos et al., 2019 [6] (NMA analysis) |
| RR of requiring additional uterotonics | 1.06 (0.86 - 1.3) | Gallos et al., 2018 [5] (Direct Analysis) |
| RR of requiring blood transfusion | 0.83 (0.67 - 1.03) | Gallos et al., 2018 [5] (Direct Analysis) |

Abbreviations: CI: Confidence Interval. NMA: Network meta-analysis. PPH: Postpartum haemorrhage. RR: relative risk

Table I: Effectiveness estimates from systematic reviews - Misoprostol vs Oxytocin - Caesarean Section

| **Medication Strategy** | **RR (95% CI)** | **Source** |
| --- | --- | --- |
| RR of PPH >500ml | 1.07 (0.92 - 1.25) | Gallos et al., 2018 [5] (Direct Analysis) |
|  | 1.03 (0.81 - 1.31) | Gallos et al., 2019 [6] (NMA analysis) |
| RR of PPH >1000ml | 0.83 (0.54 - 1.26) | Gallos et al., 2018 [5] (Direct Analysis) |
|  | 0.75 (0.48 - 1.20) | Gallos et al., 2019 [6] (NMA analysis) |
| RR of requiring additional uterotonics | 0.89 (0.69 - 1.16) | Gallos et al., 2018 [5] (Direct Analysis) |
| RR of requiring blood transfusion | 0.48 (0.19 - 1.21) | Gallos et al., 2018 [5] (Direct Analysis) |

Abbreviations: CI: Confidence Interval. NMA: Network meta-analysis. PPH: Postpartum haemorrhage. RR: relative risk

Table J: Effectiveness estimates from systematic reviews - Oxytocin vs Placebo - Mixed Delivery

| **Medication Strategy** | **RR (95% CI)** | **Source** |
| --- | --- | --- |
| RR of PPH >500ml | 0.61 (0.52 - 0.71) | Gallos et al., 2018 [5] (Direct Analysis) |
|  | 0.56 (0.46 - 0.68) | Gallos et al., 2019 [6] (NMA analysis) |
| RR of PPH >1000ml | 0.61 (0.52 - 0.73) | Gallos et al., 2018 [5] (Direct Analysis) |
|  | 0.60 (0.51 - 0.72) | Gallos et al., 2019 [6] (NMA analysis) |

Abbreviations: CI: Confidence Interval. NMA: Network meta-analysis. PPH: Postpartum haemorrhage. RR: relative risk

Table K: Effectiveness estimates from systematic reviews - Oxytocin vs Placebo - Vaginal Birth

| **Medication Strategy** | **RR (95% CI)** | **Source** |
| --- | --- | --- |
| RR of PPH >500ml | 0.61 (0.52 - 0.72) | Gallos et al., 2018 [5] (Direct Analysis) |
|  | 0.55 (0.44 - 0.68) | Gallos et al., 2019 [6] (NMA analysis) |
|  | 0.51 (0.37 - 0.72) | Salati et al., 2019 [7] |
| RR of PPH >1000ml | 0.61 (0.51 - 0.72) | Gallos et al., 2018 [5] (Direct Analysis) |
|  | 0.59 (0.50 - 0.69) | Gallos et al., 2019 [6] (NMA analysis) |
|  | 0.59 (0.42 - 0.83) | Salati et al., 2019 [7] |

Abbreviations: CI: Confidence Interval. NMA: Network meta-analysis. PPH: Postpartum haemorrhage. RR: relative risk

**Tranexamic Acid for the Prevention of PPH**

- One Cochrane review on the effectiveness of tranexamic acid for preventing PPH was published in 2015 [9].
  - This review reported that tranexamic acid decreased postpartum blood loss and the need for transfusion, based on studies of mixed quality. However, there was insufficient evidence on potential side effects.
- Other, non-Cochrane systematic reviews have since been published in in 2017 [10], 2019 [11], and 2023 [12]. These all concluded that prophylactic tranexamic acid reduces blood loss and the occurrence of PPH.
- Effectiveness estimates from these reviews are presented below:

Table L: Effectiveness estimates from systematic reviews - Tranexamic Acid vs Placebo - Mixed Delivery

| **Medication Strategy** | **OR/RR (95% CI)** | **Source** |
| --- | --- | --- |
| **OR** of PPH >500ml | **OR** – 0.34 (0.24 - 0.47) | Al-dardery et al., 2023 [12] |
| RR of PPH >500ml | 0.52 (0.42 - 0.63) | Novikova et al., 2015 [9] |
| RR of PPH >1000ml | 0.40 (0.23 - 0.71) | Novikova et al., 2015 [9] |
| RR of “PPH” (no volume specified) | 0.32 (0.19 - 0.55) | Li et al., 2017 [10] |
| RR of requiring additional uterotonics | 0.48 (0.34 - 0.68) | Novikova et al., 2015 [9] |

Abbreviations: CI: Confidence Interval. OR: Odds ratio. PPH: Postpartum haemorrhage. RR: relative risk

Table M: Effectiveness estimates from systematic reviews - Tranexamic Acid vs Placebo - Vaginal Birth

| **Medication Strategy** | **RR (95% CI)** | **Source** |
| --- | --- | --- |
| RR of “PPH” (no volume specified) | 0.37 (0.20 - 0.67) | Li et al., 2017 [10] |
| RR of “Severe PPH” (no volume specified) | 0.33 (0.3 - 3.17) | Li et al., 2017 [10] |

Abbreviations: CI: Confidence Interval. PPH: Postpartum haemorrhage. RR: relative risk

Table N: Effectiveness estimates from systematic reviews - Tranexamic Acid vs Placebo - Caesarean Section

| **Medication Strategy** | **RR (95% CI)** | **Source** |
| --- | --- | --- |
| RR of “PPH” (no volume specified) | 0.32 (0.16 - 0.61) | Li et al., 2017 [10] |
| RR of “Severe PPH” (no volume specified) | 0.31 (0.18 - 0.51) | Li et al., 2017 [10] |
| RR of massive haemorrhage with TXA | 0.39 (0.30 - 0.51) | Wang et al., 2019 [11] |
| RR of requiring additional uterotonics | 0.40 (0.30 - 0.55) | Wang et al., 2019 [11] |
| RR of requiring blood transfusion | 0.29 (0.18 - 0.49) | Wang et al., 2019 [11] |

Abbreviations: CI: Confidence Interval. PPH: Postpartum haemorrhage. RR: relative risk TXA: Tranexamic acid.

**Glyceryl trinitrate (GTN)**

- There are no Cochrane reviews on the effectiveness of GTN for the management of retained placenta and subsequent PPH.
- We could not identify any systematic reviews on the effectiveness of GTN for the management of retained placenta and subsequent PPH.
- One relevant RCT was identified – the GOT-IT RCT, which the included CE analysis is based on [13].
  - GTN was not effective at managing retained placenta or reducing blood loss of >1000ml [13].

**Negative Intrauterine Pressure Devices**

- No Cochrane or other systematic reviews on the effectiveness of negative intrauterine pressure devices was identified.
- One large observational study utilising postmarket data for the JADA® System reported that in the device succesfully arrested bleeding in 92.5% of vaginal births and 83.7% of cesarean births where it was used [14].
- Other studies assessing the JADA® System or other negative intrauterine pressure devices have only been conducted in very small study populations [15].

**Intravascular occlusion for minimising haemorrhage in placenta accreta spectrum**

- Both The International Federation of Gynecology and Obstetrics (FIGO) and the International Society for Abnormally Invasive Placenta (IS-AIP) have stated that the effectiveness of prophylactic arterial balloon catheters remains to be confirmed and that larger, prospective controlled trials are needed [16,17].
- One systematic review and meta-analysis on the topic concluded that endovascular intervention was effective at reducing haemorrhage in deliveries complicated by abnormal placental implantation [18], but the vast majority of included studies were not randomized and the conclusions may be open to significant bias.

Table O: Comparison of the effectiveness measures used in included economic evaluations on PPH prevention with the latest effectiveness data from systematic reviews and meta-analyses in the literature

| **Study (Intervention/s & Comparator/s)** | **Outcome** | **Figure observed in study, or parameter used in model**  (variation or ranges used) | **Source of evidence cited in article** | **Consistency with current effectiveness evidence** | **Comments on significant discrepancies** |
| --- | --- | --- | --- | --- | --- |
| **Gallos et al., 2019 [6]**  Interventions:   1. Oxytocin 2. Carbetocin 3. Ergometrine plus oxytocin 4. Ergometrine 5. Misoprostol plus oxytocin 6. Misoprostol | Probability of successful PPH prevention when using the following medications:   1. Oxytocin 2. Carbetocin 3. Ergometrine plus oxytocin 4. Ergometrine 5. Misoprostol plus oxytocin 6. Misoprostol | Oxytocin 0.908  Carbetocin 0.944  Ergometrine plus oxytocin 0.936  Ergometrine 0.891  Misoprostol plus oxytocin 0.931  Misoprostol 0.899 | Authorship team’s Network Meta analysis | All RRs used in the cost-effectiveness model were obtained from the authorship team’s Network Meta-Analysis, which is the benchmark for the majority of other studies (see below). |  |
| **Pickering et al., 2019 [19]**  Interventions:   1. Oxytocin 2. Carbetocin 3. Ergometrine plus oxytocin 4. Ergometrine 5. Misoprostol plus oxytocin 6. Misoprostol | Probability of successful PPH prevention when using the following medications:   1. Oxytocin 2. Carbetocin 3. Ergometrine plus oxytocin 4. Ergometrine 5. Misoprostol plus oxytocin 6. Misoprostol | Oxytocin 0.908  Carbetocin 0.944  Ergometrine plus oxytocin 0.936  Ergometrine 0.891  Misoprostol plus oxytocin 0.931  Misoprostol 0.899 | Authorship team’s Network Meta analysis | This publication is a shorter version of the above study and uses almost identical effectiveness data. |  |
| **Barrett et al., 2022 [20]**  Interventions:  1. Carbetocin as first line prophylactic agent  2. Oxytocin as first line prophylactic agent | Occurrence rate of “No PPH” in VB | Carbetocin: 94.8%, or alternatively 5.2% with PPH  Oxytocin: 88.4%, or alternatively 11.6% with PPH  Calculated RR of PPH occurring: 0.45 | Gallos et al., 2018 [5] | The RR used in the model is **lower** than the point estimates published in Gallos et al., 2018 (0.67), and Gallos et al., 2019 (0.61) for PPH>500ml in VB, but within the 95% CIs.  The RR used in the model is **similar** to the RR point estimate published in Kalafat et al., 2021 (0.44) for PPH>500ml. |  |
|  | Occurrence rate of “additional uterotonic agent” in VB | Carbetocin: 4%  Oxytocin: 10.1%  Calculated RR: 0.40 | Gallos et al., 2018 [5] | The RR used in the model is **lower** than the point estimate in Gallos et al., 2018 (0.54) and Kalafat et al., 2021 (0.52) but is within the 95% CIs. |  |
|  | Occurrence rate of “blood transfusion” in VB | Carbetocin: 1.08%  Oxytocin: 1.35%  Calculated RR: 0.80 | Gallos et al., 2018 [5] | The RR used in the model is **lower** than the point estimate in Gallos et al., 2018 (1.04) and **higher** than the point estimate in Kalafat et al., 2021 (0.67) but is within the 95% CIs of both. |  |
|  | Occurrence rate of “No PPH” in CS | Carbetocin: 86.3%, or alternatively 13.7% with PPH  Oxytocin: 69.6%, or alternatively 30.4% with PPH  Calculated RR: 0.45 | Gallos et al., 2018 [5] | The RR used in the model is **lower** than point estimates in Gallos et al., 2018 (0.71) and Gallos et al., 2019 (0.77) for PPH >500ml in CS and **is outside the 95% CI of both**.  The RR used in the model is **lower** than the point estimates in Gallos et al., 2018 (0.62), Gallos et al 2019., (0.73) and Kalafat et al., 2021 (0.83) for PPH >1000ml in CS. It is within the 95% CI for both Gallos publications, but **outside the 95% CI** of the RR published in Kalafat et al., 2021. | The discrepancies between the effectiveness estimates used and the latest figures in systematic reviews may have resulted in this study **overestimating** the effectiveness of carbetocin relative to oxytocin in preventing PPH in CS.  This may have resulted in carbetocin appearing **more cost-effective** relative to oxytocin in this analysis. |
|  | Occurrence rate of “additional uterotonic agent” in CS | Carbetocin: 7.1%  Oxytocin: 22.3%  Calculated RR: 0.32 | Gallos et al., 2018 [5] | The RR used in the model is **lower** than the point estimate in Gallos et al., 2018 (0.45) and Kalafat et al., 2021 (0.43) but is within the 95% CI of both. |  |
|  | Occurrence rate of “blood transfusion” in CS | Carbetocin: 5.94%  Oxytocin: 7.29%  Calculated RR: 0.81 | Gallos et al., 2018 [5] | The RR used in the model is **higher** than the point estimate in Gallos et al., 2018 (0.50) and Kalafat et al., 2021 (0.57) but within the 95% CI of both. |  |
| **Cook et al., 2023 [21]**  Interventions:  1. Carbetocin  2. Oxytocin  3. Misoprostol | Occurrence rate of “Mild/moderate PPH” in VB | Carbetocin: 6.1 - 6.4%  Oxytocin: 9.2 - 9.6%  (Range over health care settings)  Calculated RR: 0.66 | Adjusted figures from Gallos et al., 2018 [5] to account for anaemia rates in India for those attending PHC sites. | The RR used is **consistent** with the point estimate RR for PPH>500ml published in Gallos et al., 2018 and 2019 (0.67 and 0.61 respectively). |  |
|  | Occurrence rate of “additional uterotonic agent” in VB | Carbetocin: 5.2%  Oxytocin: 11.6%  Calculated RR: 0.45 | Adjusted figures from Gallos et al., 2018 [5] to account for anaemia rates in India for those attending PHC sites. | The RR used is s**lightly lower** than the point estimate RR published in Gallos et al., 2018 (0.54) and Kalafat et al., 2021 (0.52), but within the 95% CI of both. |  |
|  | Occurrence rate of “blood transfusion” in VB | Carbetocin: 1.2%  Oxytocin: 1.5%  Calculated RR: 0.80 | Adjusted figures from Gallos et al., 2018 [5] to account for anaemia rates in India for those attending PHC sites. | The RR used is **lower** than the RR point estimate Gallos et al., 2018 (1.04) and **higher** than the RR point estimate in Kalafat et al., (0.67) but within the 95% CI of both. |  |
|  | Occurrence rate of “Mild/moderate PPH” in CS | Carbetocin: 31.9 - 32.19%  Oxytocin: 47.1 - 47.13%  (Range over health care settings)  Calculated RR: 0.68 | Adjusted figures from Gallos et al., 2018 [5] to account for anaemia rates in India for those attending PHC sites. | The RR used is **slightly lower** than the point estimates published in Gallos et al., 2018 and 2019, (0.71 and 0.77 respectively) for PPH>500ml but is within the 95% CIs.  The RR used is **similar to** the point estimates published in Gallos et al., 2018 and 2019, (0.62 and 0.73 respectively) for PPH>1000ml. |  |
|  | Occurrence rate of “additional uterotonic agent” in CS | Carbetocin: 13.7%  Oxytocin: 30.4%  Calculated RR: 0.45 | Adjusted figures from Gallos et al., 2018 [5] to account for anaemia rates in India for those attending PHC sites. | The RR used is **identical** to the RR published in Gallos et al., 2018, and very similar to the RR published in Kalafat et al., 2021 (0.43). |  |
|  | Occurrence rate of “blood transfusion” in CS | Carbetocin: 6.6%  Oxytocin: 8.1%  Calculated RR: 0.81 | Adjusted figures from Gallos et al., 2018 [5] to account for anaemia rates in India for those attending PHC sites. | The RR used is **higher** than the RR published in Gallos et al., 2018 (0.5) and Kalafat et al., 2021 (0.57), but is within the 95% CI for both. |  |
| **You et al., 2022 [22]**  Interventions:  1. Carbetocin (100 μg IV)  2. Oxytocin (10 IU IV bolus) | Relative risk of PPH >500ml in VB and CS. | 0.72 (0.56 - 0.93) | Gallos et al., 2018 [5] | The point estimate and range of RR used are **very similar** to the RR published in Gallos et al., 2018 and 2019 for mixed delivery (0.75 and 0.72 respectively). |  |
| **Gil-Rojas et al., 2018 [23]**  Interventions:  1. Carbetocin (100 μg)  2. Oxytocin (5 - 10 IU IM for VB or 5IU followed by 30IU infusion for CS) | Relative risk of PPH in carbetocin group compared to oxytocin in VB | 0.950 (0.43 - 2.09) | Boucher et al., 2004 [24] | The RR point estimate used is **higher** than the RR published in Gallos et al., 2018, 2019 and Kalafat et al., 2021 (0.67, 0.61, 0.44 respectively) for PPH of >500ml in VB but is within the stated 95% CIs.  The RR point estimate used is **higher** than the RR published in Gallos et al., 2018 and 2019 (0.53, 0.68 respectively) for PPH of >1000ml in VB but is within the 95% CI. |  |
|  | Relative risk of needing additional uterotonics in carbetocin group compared to oxytocin in VB | 0.93 (0.44 - 1.94) | Boucher et al., 2004 [24] | The RR used is **higher** than the RR published in Gallos et al., 2018 (0.54) but is within stated 95% CI.  The RR used is **higher** than the RR published in Kalafat et al., 2021 (0.52) and is **outside of the stated 95% CI.** | The discrepancies between the effectiveness estimates used and the latest figures in systematic reviews may have resulted in this study **underestimating** the effectiveness of carbetocin relative to oxytocin in preventing the need for additional uterotonics.  This may have resulted in carbetocin appearing **less cost-effective** relative to oxytocin in this analysis. |
|  | Relative risk of PPH in carbetocin group compared to oxytocin in CS | 0.52 (0.29 - 0.93) | Borruto et al., 2009 [25] | The RR used is **lower** than the RR published in Gallos et al., 2018 and 2019 (0.71, 0.77 respectively) for PPH of >500ml in CS but is just within the stated 95% CI of both.  The RR used is **lower** than the RR published in Gallos et al., 2018, Gallos et al., 2019 (0.62 & 0.73) for PPH >1000ml in CS but is within the 95% CI of both.  The RR used is **lower** than the RR published in Kalafat et al., 2021 (0.83) for PPH of >1000ml in CS **and is outside the stated 95% CI**. | The discrepancies between the effectiveness estimates used and the latest figures in systematic reviews may have resulted in this study **overestimating** the effectiveness of carbetocin relative to oxytocin in preventing PPH in CS.  This may have resulted in carbetocin appearing **more cost-effective** relative to oxytocin in this analysis. |
|  | Relative risk of needing additional uterotonics in carbetocin group compared to oxytocin in CS | 0.70 (0.51 - 0.98) | Sheldon et al., 2014 [26] | The RR point estimate used is **higher** than the RR published in Gallos et al., 2018 and Kalafat et al., 2021 (0.45, 0.43 respectively) for additional uterotonic requirement in CS. The RR used is within the 95% CI for Gallos et al., 2018 but is **outside the 95% CI** for Kalafat et al., 2021. | The discrepancies between the effectiveness estimates used and the latest figures in systematic reviews may have resulted in this study **underestimating** the effectiveness of carbetocin relative to oxytocin in preventing the need for additional uterotonics.  This may have resulted in carbetocin appearing **less cost-effective** relative to oxytocin in this analysis. |
| **Briones et al., 2020 [27]**  Interventions:  1. Carbetocin (100 μg) 2. Oxytocin (10 IU) | RR of PPH >1000ml in carbetocin group compared to oxytocin group in CS | 0.62 | Gallos et al., 2018 [5] | The RR used is **identical** to the RR published in Gallos et al., 2018 and similar to those published in Gallos et al., 2019 (0.73) and Kalafat et al., 2021 (0.83). |  |
|  | Probability of requiring additional uterotonics in CS | Carbetocin: 0.1437  Oxytocin: 0.2318  Calculated RR: 0.62 | Gallos et al., 2018 [5] | The RR used is **higher** than the published point estimates in Gallos et al., 2018 (0.45), but within the 95% CI.  The RR used is **higher** than the published point estimates in Kalafat et al., 2021 (0.43), and **is outside the 95% CI.** | The discrepancies between the effectiveness estimates used and the latest figures in systematic reviews may have resulted in this study **underestimating** the effectiveness of carbetocin relative to oxytocin in preventing the need for additional uterotonics in CS.  This may have resulted in carbetocin appearing **less** **cost-effective** relative to oxytocin in this analysis. |
|  | Probability of requiring blood transfusion in CS | Carbetocin: 0.0178  Oxytocin: 0.0287  Calculated RR: 0.62 | Gallos et al., 2018 [5] | The RR used is **similar** to those published in Gallos et al., 2019 (0.50) and Kalafat et al., 2021 (0.57) and is within the 95% CI published in both. |  |
|  | RR of PPH >500ml in carbetocin group compared to oxytocin group in VB | 0.67 | Gallos et al., 2018 [5] | The RR used is **identical** to the RR published in Gallos et al., 2018 and **similar** to those published in Gallos et al., 2019 (0.61). |  |
|  | Probability of requiring additional uterotonics in VB | Carbetocin: 0.0715  Oxytocin: 0.1067  Calculated RR: 0.67 | Gallos et al., 2018 [5] | The RR used is **higher** than those published in Gallos et al., 2018 (0.54) and Kalafat et al., 2021 (0.52) but is within the 95% CI published in both. |  |
|  | Probability of requiring blood transfusion in VB | Carbetocin: 0.0091  Oxytocin: 0.0134  Calculated RR: 0.68 | Gallos et al., 2018 [5] | The RR used is **identical** to the RR published in Gallos et al., 2018 for mixed delivery.  The RR used is very **similar** to the RR published in Kalafat et al., 2021 for transfusion requirement in VB (0.67).  The RR used is **lower** than the RR published in Gallos et al., 2018 for VB (1.04) but is within the 95% CI. |  |
| **Luni et al., 2017 [28]**  Interventions:  1. Carbetocin  2. Oxytocin | Proportion of patients with PPH>500ml in CS | Carbetocin: 0.28  Oxytocin: 0.43  Calculated RR: 0.65 | Authorship team’s cohort study data. | The observed RR is **similar** to the RR published in Gallos et al., 2018 (0.71) and Gallos et al., 2019 (0.77). |  |
|  | Proportion requiring additional uterotonics in CS | Carbetocin: 0.07  Oxytocin: 0.48  Calculated RR: 0.15 | Authorship team’s cohort study data. | The observed RR in this sample is **lower** than the RR published in Gallos et al., 2018 (0.45) and Kalafat et al., 2021 (0.43) and is **outside the 95% CI of both.** | The discrepancies between the effectiveness data observed and the latest figures in systematic reviews may have resulted in this study **overstating** the effectiveness of carbetocin relative to oxytocin in preventing the need for additional uterotonics in CS.  This may have resulted in carbetocin appearing **more cost-effective** relative to oxytocin in this analysis. |
| **van der Nelson et al., 2017 [29]**  Interventions:  1. Carbetocin (100μg IV)  2. Oxytocin (5 IU IV) | Probability of PPH >500ml in CS | Carbetocin: 0.39  Oxytocin: 0.59  Calculated RR: 0.66 | Su et al., 2012 [30] | The RR used is **similar** to the RR published in Gallos et al., 2018 (0.71) and Gallos et al., 2019 (0.77). |  |
|  | Probability of requiring additional uterotonics in CS | Carbetocin: 0.136  Oxytocin: 0.215  Calculated RR: 0.63 | Su et al., 2012 [30] | The RR used is **higher** than the RR published in Gallos et al., 2018 (0.45) but is within the stated 95% CI.  The RR used is higher than the RR published in Kalafat et al., 2021 (0.43) and is **outside of the 95% CI.** | The discrepancies between the effectiveness estimates used and the latest figures in systematic reviews may have resulted in this study **underestimating** the effectiveness of carbetocin relative to oxytocin in preventing the need for additional uterotonics in CS.  This may have resulted in carbetocin appearing **less cost-effective** relative to oxytocin in this analysis. |
| **Wohling et al., 2019 [31]**  Interventions:  1. Carbetocin (100 μg IV)  2. Oxytocin (5-10 IU, IV slow push) | Proportion with PPH>500ml in CS | Carbetocin: 0.273  Oxytocin: 0.394  Calculated RR: 0.69 | Authorship team’s retrospective cohort study data. | The observed RR is **similar** to the RR published in Gallos et al., 2018 (0.71) and Gallos et al., 2019 (0.77). |  |
|  | Proportion with PPH>1000ml in CS | Carbetocin: 0.078  Oxytocin: 0.097  Calculated RR: 0.80 | Authorship team’s retrospective cohort study data. | The observed RR is **higher** than RR published in Gallos et al., 2018 (0.62), and is **similar** to the RR published in Gallos et al., 2019 (0.73) and Kalafat et al., 2021 (0.83) and is within the 95% CI of all three estimates. |  |
|  | Proportion requiring additional uterotonics in CS | Carbetocin: 0.269  Oxytocin: 0.469  Calculated RR: 0.57 | Authorship team’s retrospective cohort study data. | The observed RR is **higher** than the RR published in Gallos et al., 2018 (0.45) and Kalafat et al., 2021 (0.43) but is within the 95% CI of both estimates. |  |
|  | Proportion requiring blood transfusion in CS | Carbetocin: 0.012  Oxytocin: 0.016  Calculated RR: 0.75 | Authorship team’s retrospective cohort study data. | The observed RR is **higher** than the RR published in Gallos et al., 2018 (0.50) and in Kalafat et al., 2021 (0.57) but is within the 95% CI of both studies. |  |
| **Caceda et al., 2018 [32]**  Interventions:  1. Carbetocin as first line for prevention  2. Oxytocin as first line for prevention | Proportion with PPH (volume not listed) in CS | Carbetocin: 3.398 per 1000 deliveries (0.00398)  Oxytocin: 13.729 per 1000 deliveries (0.013729)  Calculated RR: 0.25 | Su et al., 2012 [30] | The RR used is **lower** than the RR published in Gallos et al., 2018 (0.71), and Gallos et al., 2019 (0.77) for PPH>500ml in CS and is **outside of the 95% CIs of both**.  The RR used is **lower** than the RR published in Gallos et al., 2018 (0.62), Gallos et al., 2019 (0.73), and Kalafat et al., 2021 (0.83) for PPH>1000ml in CS and is **outside of the 95% CIs stated in all three analyses**. | The discrepancies between the effectiveness estimates used and the latest figures in systematic reviews may have resulted in this study **overestimating** the effectiveness of carbetocin relative to oxytocin in preventing PPH in CS.  This may have resulted in carbetocin appearing **more cost-effective** relative to oxytocin in this analysis. |
|  | Proportion requiring blood transfusion in CS | Carbetocin: 0.072 per 1000 deliveries  Oxytocin: 0.363 per 1000 deliveries  Calculated RR: 0.20 | Su et al., 2012 [30] | The RR used is **lower** than the RR published in Gallos et al., 2018 (0.50) and Kalafat et al., 2021 (0.57) and is **outside the 95% CI of both**. | The discrepancies between the effectiveness estimates used and the latest figures in systematic reviews may have resulted in this study **overestimating** the effectiveness of carbetocin relative to oxytocin in preventing the need for blood transfusion in CS.  This may have resulted in carbetocin appearing **more cost-effective** relative to oxytocin in this analysis. |
| **Henriquez-Trujillo et al., 2017 [33]**  Interventions:  1. Carbetocin  2. Oxytocin | Probability of requiring additional uterotonic in CS | Carbetocin: 0.1365 (0.1111 - 0.1667)  Oxytocin: 0.2147 (0.1833 - 0.2497)  Calculated RR: 0.64 | Su et al., 2012 [30] | The RR used is **higher** than the RR published in Gallos et al., 2018 (0.45) but within the stated 95% CI.  The RR used is **higher** than the RR published Kalafat et al., (0.43) and is **outside of the stated 95% CI.** | The discrepancies between the effectiveness estimates used and the latest figures in systematic reviews may have resulted in this study **underestimating** the effectiveness of carbetocin relative to oxytocin in preventing the need for additional uterotonics in CS.  This may have resulted in carbetocin appearing **less cost-effective** relative to oxytocin in this analysis. |
| **Voon et al., 2018 [34]**  Interventions:  1. Carbetocin (100μg IV)  2. Oxytocin (5 IU IV) | Probability of PPH In CS | Carbetocin: 0.142  Oxytocin: 0.178  Calculated RR: 0.80 | Boucher et al., 1998 [35]  Dansereau et al., 1999 [36]  Borruto et al., 2009 [25]  Attilakos et al., 2010 [37]  Razali et al., 2016 [38]  El Behery et al., 2016 [39]  Whigham et al., 2016 [40] | The RR used is **similar** to the RR published in Gallos et al., 2018 (0.71) and Gallos et al., 2019 (0.77) for PPH>500ml in CS.  The RR used is **similar** to the RR published in Gallos et al., 2018 (0.62), Gallos et al., 2019 (0.73), and Kalafat et al., (0.83) for PPH >1000ml in CS. |  |
|  | Probability of requiring additional uterotonics in CS | Carbetocin: 0.199  Oxytocin: 0.352  Calculated RR: 0.56 | Boucher et al., 1998 [35]  Dansereau et al., 1999 [36]  Borruto et al., 2009 [25]  Attilakos et al., 2010 [37]  Razali et al., 2016 [38]  El Behery et al., 2016 [39]  Whigham et al., 2016 [40] | The RR used is **similar** to the RR published in Gallos et al., 2018 (0.45) and Kalafat et al., 2021 (0.43) and is within the 95% CI of both. |  |
|  | Probability of requiring transfusion in CS | Carbetocin: 0.0149  Oxytocin: 0.0497  Calculated RR: 0.30 | Boucher et al., 1998 [35]  Dansereau et al., 1999 [36]  Borruto et al., 2009 [25]  Attilakos et al., 2010 [37]  Razali et al., 2016 [38]  El Behery et al., 2016 [39]  Whigham et al., 2016 [40] | The RR used is **lower** than the RR published in Gallos et al., 2018 (0.50) but is within the 95% CI.  The RR used is **lower** than the RR published in Kalafat et al., 2021 (0.57) and **is outside of the 95% CI.** | The discrepancies between the effectiveness estimates used and the latest figures in systematic reviews may have resulted in this study **overestimating** the effectiveness of carbetocin relative to oxytocin in preventing the need for blood transfusions in CS.  This may have resulted in carbetocin appearing **more cost-effective** relative to oxytocin in this analysis. |
| **Higgins et al., 2011 [41]**  Interventions:  1. Carbetocin (100μg IV)  2. Oxytocin (5IU) | Proportion of patients with PPH in CS | Carbetocin: 63.55%  Oxytocin: 68.63%  Calculated RR: 0.93 | Data from authorship team’s matched cohort study. | The observed RR is **higher** than the RR published in Gallos et al., 2018 (0.71), Gallos et al., 2019 (0.77) for PPH >500ml in CS but is within the stated 95% CIs.  The observed RR is **higher** than the RR published in Gallos et al., 2018 (0.62), Gallos et al., 2019 (0.73), and Kalafat et al., 2021 (0.83) for PPH >1000ml but is within the stated 95% CIs. |  |
|  | Proportion requiring additional uterotonic in CS | Carbetocin: 34.26%  Oxytocin: 46%  Calculated RR:0.75 | Data from authorship team’s matched cohort study. | The observed RR is **higher** than the RR published in Gallos et al., 2018 (0.45) and Kalafat et al., 2021 (0.43) and **is outside of the stated 95% CIs for both.** | The discrepancies between the effectiveness data observed and the latest figures in systematic reviews may have resulted in this study **understating** the effectiveness of carbetocin relative to oxytocin in preventing the need for additional uterotonics in CS.  This may have resulted in carbetocin appearing **less cost-effective** relative to oxytocin in this analysis. |
|  | Proportion of patients requiring transfusion | Carbetocin: 5.66%  Oxytocin: 4.08%  Calculated RR: 1.39 | Data from authorship team’s matched cohort study. | The observed RR is **higher** than the RR published in Gallos et al., 2018 (0.50) and Kalafat et al., 2021 (0.57) and is **outside the stated 95% CI for both.** | The discrepancies between the effectiveness data observed and the latest figures in systematic reviews may have resulted in this study **understating** the effectiveness of carbetocin relative to oxytocin in preventing the need for transfusions following CS.  This may have resulted in carbetocin appearing **less cost-effective** relative to oxytocin in this analysis. |
| **Matthijsse et al., 2022 [42]**  Interventions:  1. Carbetocin (100μg IM)  2. Oxytocin (10IU bolus) | Proportion experiencing mild/moderate PPH in VB | Carbetocin: 0.0617  Oxytocin: 0.092  Calculated RR: 0.67 | Gallos et al., 2018 [5] | The RR used is **identical** to the RR published in Gallos et al., 2018 for PPH>500ml in VB. |  |
|  | Proportion experiencing a severe PPH event in VB | Carbetocin: 0.0261  Oxytocin: 0.0300  Calculated RR: 0.87 | Gallos et al., 2018 [5] | The RR used is **higher** than the RR published in Gallos et al., 2018 (0.68) and Gallos et al., 2019 (0.53) for PPH>1000ml in VB but is within the 95% CI of both. |  |
|  | Proportion requiring additional uterotonic in VB | Carbetocin: 0.052  Oxytocin: 0.116  Calculated RR: 0.45 | Gallos et al., 2018 [5] | The RR used is **similar** to the RR published in Gallos et al., 2018 (0.54) and Kalafat et al., 2021 (0.52). |  |
|  | Proportion requiring blood transfusion in VB | Carbetocin: 0.012  Oxytocin: 0.015  Calculated RR: 0.8 | Gallos et al., 2018 [5] | The RR used is **between** the point estimates stated in Gallos et al., 2018 (1.04) and Kalafat et al., 2021 (0.67) and is within the 95% CI of both. |  |
| **Vlassof et al., 2016 [43]**  Interventions:  1. Oxytocin Uniject (10IU IM)  2. Misoprostol (600μg PO)  3. Standard care | Referral to health centre for PPH management (proxy for number of PPHs) | Misoprostol: 74 per 150,000 patients  Oxytocin: 490 per 150,000 patients  Calculated RR: 0.15 | Diop et al., 2016 [44] | The RR used is **lower** than the RR point estimates published in Gallos et al., 2018, and Gallos et al., 2019 for both PPH>500ml, and PPH>1000ml, in either mixed delivery or VB only subgroup analyses and is **outside all published 95% CIs.** | The discrepancies between the effectiveness estimates used and the latest figures in systematic reviews may have resulted in this study **overestimating** the effectiveness of misoprostol relative to oxytocin in preventing PPH.  This may have resulted in misoprostol appearing **more cost-effective** relative to oxytocin in this analysis. |
| **Diaz et al., 2009 [45]**  Interventions:  1. Implementation of AMTSL with oxytocin, equipment upgrades, and staff training.   2. No active implementation of AMTSL, equipment upgrades or staff training. | Proportion of deliveries with PPH | AMTSL with Oxytocin: 6.8%  No Uterotonic: 16.5%  Calculated RR: 0.41 | Rogers et al., 1998 [46] | The RR used is **lower** than the RR point estimates published in Gallos et al., 2018 and Gallos et al., 2019, for both PPH>500ml, and PPH>1000ml in either mixed populations or only VB, and is **outside the stated 95% CIs** | The discrepancies between the effectiveness estimates used and the latest figures in systematic reviews may have resulted in this study **overestimating** the effectiveness of AMTSL using oxytocin relative to no uterotonic in preventing PPH.  This may have resulted in AMTSL using oxytocin appearing **more cost-effective** relative to no uterotonic in this analysis. |
| **Tsu et al., 2009 [47]**  Interventions:  1. Oxytocin Uniject (10 IU)  2. Oxytocin (10 IU drawn from ampoules)  3. No AMTSL | Proportion with moderate PPH (>500ml) in community health centre | AMTSL with Oxytocin: 2.5%  No uterotonic: 3.6%  Calculated RR: 0.69 | Tsu et al., 2006 [48] | **There is limited data specific to the community setting.**  The RR used is **similar** to the RR published in Gallos et al., 2018 for PPH >500ml for VB (0.61) and is within the 95% CI.  The RR used is **higher** than the RR published in Gallos et al., 2019 for PPH >500ml for VB (0.55) and **is just outside the 95% CI.** | The discrepancies between the effectiveness estimates used and the latest figures in systematic reviews may have resulted in this study **underestimating** the effectiveness of oxytocin relative to no uterotonic in preventing PPH>500ml in the community setting.  This may have resulted in oxytocin appearing **less cost-effective** relative to no uterotonic in this analysis. |
|  | Proportion with severe PPH (>1000ml) in community health centre | AMTSL with Oxytocin: 0.34%  No uterotonic: 0.43%  Calculated RR: 0.79 | Tsu et al., 2006 [48] | **There is limited data specific to the community setting.**  The RR used is **higher** than the RR published in Gallos et al., 2018 for PPH >1000ml for VB (0.61) and **outside the 95% CI.**  The RR used is **higher** than the RR published in Gallos et al., 2019 for PPH >1000ml for VB (0.59) and **outside the 95% CI.** | The discrepancies between the effectiveness estimates used and the latest figures in systematic reviews may have resulted in this study **underestimating** the effectiveness of oxytocin relative to no uterotonic in preventing PPH>1000ml in the community setting.  This may have resulted in oxytocin appearing **less cost-effective** relative to no uterotonic in this analysis. |
|  | Proportion with moderate PPH (>500ml) in district hospital | AMTSL with Oxytocin: 0.9%  No uterotonic: 4.4%  Calculated RR: 0.20 | Tsu et al., 2006 [48] | The RR used is **lower** than the RR published in Gallos et al., 2018 and Gallos et al., 2019 for PPH >500ml in VB (0.61 & 0.55 respectively) and is **outside the 95% CI of both.** | The discrepancies between the effectiveness estimates used and the latest figures in systematic reviews may have resulted in this study **overestimating** the effectiveness of oxytocin relative to no uterotonic in preventing PPH>500ml in the hospital setting.  This may have resulted in oxytocin appearing **more cost-effective** relative to no uterotonic in this analysis. |
|  | Proportion with severe PPH (>1000ml) in in district hospital | AMTSL with Oxytocin: 0.17%  No uterotonic: 0.72%  Calculated RR: 0.24 | Tsu et al., 2006 [48] | The RR used is **lower** than the RR published in Gallos et al., 2018 and Gallos et al., 2019 for PPH >1000ml for VB (0.61 & 0.59 respectively) and is **outside the 95% CI of both.** | The discrepancies between the effectiveness estimates used and the latest figures in systematic reviews may have resulted in this study **overestimating** the effectiveness of oxytocin relative to no uterotonic in preventing PPH>1000ml in the hospital setting.  This may have resulted in oxytocin appearing **more cost-effective** relative to no uterotonic in this analysis. |
| **Pichon-Riviere et al., 2015 [49,50]**  Interventions:  1. Oxytocin (10 IU IM or 5 IU IV drawn from ampoules)  2. Oxytocin Uniject (10 IU IM) | RR of PPH when receiving oxytocin compared to no oxytocin | 0.50 (0.43 - 0.57) | Cotter et al., 2001 [51] | The RR used is **lower** than the RR published in Gallos et al., 2018 for PPH>500ml (0.61) and PPH>1000ml (0.61) for mixed births and is **outside the 95% CI for both**.  The RR used is **lower** than the RR published in Gallos et al., 2019 for PPH>500ml (0.56) but is within the 95% CI.  The RR used is **lower** than the RR published in Gallos et al., 2019 for PPH>1000ml (0.60) and is **just outside of the 95% CI.** | The discrepancies between the effectiveness estimates used and the latest figures in systematic reviews may have resulted in this study **overestimating** the effectiveness of both oxytocin formulations in preventing PPH.  This may have resulted in oxytocin Uniject appearing **more cost-effective** relative to oxytocin (ampoules) in this analysis as it was also assumed to be used more accessible (see below). |
|  | Introducing Uniject oxytocin would reduce the gap in uterotonic coverage by: | 30.23% (12.03% – 53.75%) | Authorship team’s Delphi panel for this study | No systematic review evidence identified. | It is not clear if this key assumption underpinning the model, that adopting the Uniject formulation increases access to oxytocin, is supported in the literature. |
| **Carvalho et al., 2020 [52]**  Interventions:  1. Inhaled oxytocin (note: not yet a licensed product)  2. Standard of care in that country and setting (different uterotonics depending on the setting) | RR of mild PPH with inhaled oxytocin compared to no uterotonic | 0.61 | Assumption (assumed to be the same as injectable oxytocin) | It is not yet established whether inhaled oxytocin is equivalent to injected oxytocin [53]. | The key assumption underpinning this model is not yet supported in the literature. |
| **Sutherland et al., 2009 [54]**  Interventions:  1. Misoprostol (600 μg administered by VHW).  2. VHW attendance but no uterotonic. | Reduction in number of PPH>500ml by introducing misoprostol (administered by trained attendant) for home births compared to no uterotonic. | 50%  Calculated RR: 0.50 | Derman et al., 2006 [55] | The RR used is **lower** than the published RR in Gallos et al., 2018 (0.75) for PPH>500ml in VB and is **outside of the 95% CI**.  The RR used is **lower** than the published RR in Gallos et al., 2019 (0.60) for PPH>500ml in VB but is within the 95% CI. | The discrepancies between the effectiveness estimates used and the latest figures in systematic reviews may have resulted in this study **overestimating** the effectiveness of misoprostol relative to no uterotonic in preventing PPH> 500ml in VB.  This may have resulted in misoprostol appearing **more cost-effective** relative to no uterotonic in this analysis. |
|  | Reduction in number of PPH>1000ml by introducing misoprostol (administered by trained attendant) for home births compared to no uterotonic | 80%  Calculated RR: 0.20 | Derman et al., 2006 [55] | The RR used is **lower** than the published RR is Gallos et al., 2018 (0.73) and Gallos et al., 2019 (0.76) for PPH >1000ml in VB and is **outside the 95% CI of both.** | The discrepancies between the effectiveness estimates used and the latest figures in systematic reviews may have resulted in this study **overestimating** the effectiveness of misoprostol relative to no uterotonic in preventing PPH> 1000ml in VB.  This may have resulted in misoprostol appearing **more cost-effective** relative to no uterotonic in this analysis. |
| **Sutherland et al., 2010 [56]**  Interventions:  1. Misoprostol Prevention: 600μg oral misoprostol, if they haemorrhage >1000ml they have 75% chance of referral to health centre.   2. Misoprostol Treatment: 800μg sublingual misoprostol after 700ml blood loss.  3. Standard care with unskilled assistant and no medication | Reduction in number of PPH>500ml by introducing misoprostol (administered by trained attendant) for home births compared to no uterotonic. | 50%  Calculated RR: 0.50 | Derman et al., 2006 [55] | The RR used is **lower** than the published RR in Gallos et al., 2018 (0.75) for PPH>500ml in VB and is **outside of the 95% CI**.  The RR used is **lower** than the published RR in Gallos et al., 2019 (0.60) for PPH>500ml in VB but is within the 95% CI. | The discrepancies between the effectiveness estimates used and the latest figures in systematic reviews may have resulted in this study **overestimating** the effectiveness of misoprostol relative to no uterotonic in preventing PPH> 500ml in VB.  This may have resulted in misoprostol appearing **more cost-effective** relative to no uterotonic in this analysis. |
|  | Reduction in number of PPH>1000ml by introducing misoprostol (administered by trained attendant) for home births compared to no uterotonic | 80%  Calculated RR: 0.20 | Derman et al., 2006 [55] | The RR used is **lower** than the published RR is Gallos et al., 2018 (0.73) and Gallos et al., 2019 (0.76) for PPH >1000ml in VB and is **outside the 95% CI of both.** | The discrepancies between the effectiveness estimates used and the latest figures in systematic reviews may have resulted in this study **overestimating** the effectiveness of misoprostol relative to no uterotonic in in preventing PPH> 1000ml in VB.  This may have resulted in misoprostol appearing **more cost-effective** relative to no uterotonic in this analysis. |
| **Goldie et al., 2010 [57]**  Interventions:  1. Misoprostol distribution in community (in home and birthing centres) in addition to general infrastructure and service upgrades.  2. Implementing service and infrastructure upgrades without adding misoprostol distribution. | Reduction in PPH rates by introducing community administration of misoprostol (administered by trained attendant) as an adjunct to service improvements, compared to no misoprostol | 50%  Calculated RR: 0.50 | Hofmeyr & Gulmezoglu 2008 [58] | The RR used is **lower** than the published RR in Gallos et al., 2018 (0.75) for PPH>500ml in VB and is **outside of the 95% CI**.  The RR used is **lower** than the published RR in Gallos et al., 2019 (0.60) for PPH>500ml in VB but is within the 95% CI.  The RR used is **lower** than the published RR in Gallos et al., 2018 (0.73) for PPH>1000ml in VB and is **outside of the 95% CI**.  The RR used is **lower** than the published RR in Gallos et al., 2019 (0.76) for PPH>1000ml in VB and is **outside of the 95% CI**. | The discrepancies between the effectiveness estimates used and the latest figures in systematic reviews may have resulted in this study **overestimating** the effectiveness of misoprostol relative to no misoprostol in preventing PPH.  This may have resulted in misoprostol appearing **more cost-effective** relative to no misoprostol in this analysis. |
| **Lubinga et al., 2015 [59,60]**  Interventions:  1. Misoprostol (600 μg PO distributed to women in their antenatal visit or as part of a safe delivery kit).  2. Oxytocin (10 IU IM but limited to only those they deliver in a facility). | RR of PPH with oxytocin compared to no uterotonic | 0.53 (0.38 - 0.74) | Westhoff et al., 2013 [61] | The RR used is **similar** to the RR published in Gallos et al., 2018 (0.61 & 0.61) and Gallos et al., 2019 (0.56 & 0.60) for PPH>500ml or >1000ml respectively in mixed delivery. |  |
|  | RR of PPH with misoprostol in skilled delivery compared to no uterotonic | 0.84 (0.73 - 0.97) | Authorship team’s meta-analysis | The RR used is **higher** than the published RR in Gallos et al., 2018 (0.75) for PPH>500ml in VB but is within the 95% CI.  The RR used is **higher** than the published RR in Gallos et al., 2019 (0.60) for PPH>500ml in VB **and is outside the 95% CI.**  The RR used is **higher** than the RR published in Gallos et al., 2018 (0.73) and Gallos et al., 2018 (0.76) for PPH >1000ml in VB but is within the 95% CI of both. | The discrepancies between the effectiveness estimates used and the latest figures in systematic reviews may have resulted in this study **underestimating** the effectiveness of misoprostol relative to no uterotonic in preventing PPH when given by a skilled provider.  This may have resulted in misoprostol appearing **less cost-effective** relative to no uterotonic in this analysis. |
|  | RR of PPH with misoprostol in unskilled delivery (advanced distribution of misoprostol) compared to no uterotonic. | 0.53 (0.39 - 0.74) | Derman et al., 2006 [55] | The benefits and harms of advanced distribution of misoprostol and unskilled administration are unclear [62].  If we assume the same efficacy as administration by a skilled provider:  The RR used is **lower** than the published RR in Gallos et al., 2018 (0.75) for PPH>500ml in VB and is **outside of the 95% CI**.  The RR used is **lower** than the published RR in Gallos et al., 2019 (0.60) for PPH>500ml in VB but is within the 95% CI.  The RR used is **lower** than the published RR in Gallos et al., 2018 (0.73) for PPH>1000ml in VB and is **outside of the 95% CI**.  The RR used is **lower** than the published RR in Gallos et al., 2019 (0.76) for PPH>1000ml in VB and is **outside of the 95% CI**. | If we assume the same efficacy as administration by a skilled provider:  The discrepancies between the effectiveness estimates used and the latest figures in systematic reviews may have resulted in this study **overestimating** the effectiveness of misoprostol relative to no uterotonic in preventing PPH in the community.  This may have resulted in advanced distribution of misoprostol appearing **more cost-effective** relative to no uterotonic in this analysis. |
| **Prata et al., 2010 [63]**  Interventions:  Note: Only the comparison of ANC vs ANC-miso interventions met inclusion criteria for this review.  1. Antenatal and postpartum care as outlined in the "WHO Mother Baby Package".   2. Implementing the same as above, plus community distribution of misoprostol for home births. | Number of maternal deaths averted per 500,000 deliveries in low infrastructure setting | Standard antenatal care + Misoprostol: 30 per 500,000  Standard antenatal care alone: 7 per 500,000 | No clear source cited | Reduction in maternal mortality from misoprostol use compared to no uterotonic use is not supported in systematic reviews.  RR: 1 (0.1 – 9.59) Gallos et al., 2018  RR: 0.98 (0.23 - 4.12) Gallos et al., 2019 | It is unclear whether misoprostol reduces maternal mortality compared to no uterotonic, and therefore unclear whether the economic analysis based on this is valid. |
|  | Number of maternal deaths averted per 500,000 deliveries in medium infrastructure setting | Standard antenatal care + Misoprostol: 30 per 500,000  Standard antenatal care alone: 7 per 500,000 | No clear source cited | Reduction in maternal mortality from misoprostol use compared to no uterotonic use is not supported in systematic reviews.  RR: 1 (0.1 – 9.59) Gallos et al., 2018  RR: 0.98 (0.23 - 4.12) Gallos et al., 2019 | It is unclear whether misoprostol reduces maternal mortality compared to no uterotonic, and therefore unclear whether the economic analysis based on this is valid. |
|  | Number of maternal deaths averted per 500,000 deliveries in high infrastructure setting | Standard antenatal care + Misoprostol: 19 per 500,000  Standard antenatal care alone: 4 per 500,000 | No clear source cited | Reduction in maternal mortality from misoprostol use compared to no uterotonic use is not supported in systematic reviews.  RR: 1 (0.1 – 9.59) Gallos et al., 2018  RR: 0.98 (0.23 - 4.12) Gallos et al., 2019 | It is unclear whether misoprostol reduces maternal mortality compared to no uterotonic, and therefore unclear whether the economic analysis based on this is valid. |
| **Lang et al., 2015 [64]**  Interventions:  Scenario 1:  1. Oxytocin (in hospitals) and misoprostol (in community).  2. Oxytocin (in hospitals and no treatment in the community)  Scenario 2:  1. Misoprostol in both hospital and community.  2. No uterotonics in either hospitals or community settings. | RR of PPH with misoprostol vs placebo | 0.77 (0.6 - 0.99) | Tunçalp et al., 2012 [65] | The RR used is **similar** to the RR published in Gallos et al., 2018 (0.75) for PPH>500ml for mixed cohorts of births.  The RR used is **higher** than the RR published in Gallos et al., 2019 (0.61) for PPH>500ml for mixed cohorts of births and is just **outside the 95% CI**. | The discrepancies between the effectiveness estimates used and the latest figures in systematic reviews may have resulted in this study **underestimating** the effectiveness of misoprostol relative to placebo in preventing PPH>500ml in the main analysis.  This may have resulted in misoprostol appearing **less cost-effective** relative to placebo in this analysis. |
|  | RR of severe PPH with misoprostol vs placebo | 0.91 (0.51 - 1.63) | Tunçalp et al., 2012 [65] | The RR used is **higher** than the RR published in Gallos et al., 2018 (0.73) for PPH>1000ml for mixed cohorts of births but is within the 95% CI.  The RR used is **higher** than the RR published in Gallos et al., 2019 (0.73) for PPH>1000ml for mixed cohorts of births and is just **outside the 95% CI**. | The discrepancies between the effectiveness estimates used and the latest figures in systematic reviews may have resulted in this study **underestimating** the effectiveness of misoprostol relative to placebo in preventing PPH>1000ml in the main analysis.  This may have resulted in misoprostol appearing **less cost-effective** relative to placebo in this analysis. |
|  | RR of requiring additional uterotonics with misoprostol vs placebo | 0.86 (0.66 - 1.13) | Tunçalp et al., 2012 [65] | The RR used is **higher** than the RR published in Gallos et al., 2018 (0.67) for requiring additional uterotonics for mixed cohorts of births but is just within the 95% CI.  The RR used is **higher** than the RR published in Gallos et al., 2019 (0.43) for requiring additional uterotonics for mixed cohorts of births and is **outside the 95% CI.** | The discrepancies between the effectiveness estimates used and the latest figures in systematic reviews may have resulted in this study **underestimating** the effectiveness of misoprostol relative to placebo in preventing the need for additional uterotonics.  This may have resulted in misoprostol appearing **less** **cost-effective** relative to placebo in this analysis. |
|  | RR of requiring blood transfusion with misoprostol vs placebo | 0.24 (0.06 - 0.94) | Tunçalp et al., 2012 [65] | The RR used is **lower** than the RR published in Gallos et al., 2018 (0.46) for requiring transfusion for mixed cohorts of births but is within the 95% CI.  The RR used is **lower** than the RR published in Gallos et al., 2019 (0.49) for requiring additional uterotonics for mixed cohorts of births and is **outside the 95% CI**. | The discrepancies between the effectiveness estimates used and the latest figures in systematic reviews may have resulted in this study **overestimating** the effectiveness of misoprostol relative to placebo in preventing the need for transfusion in the main analysis.  This may have resulted in misoprostol appearing **more cost-effective** relative to placebo in this analysis. |
|  | Alternative RR used in scenario analysis:  RR of PPH with misoprostol vs placebo | 0.58 (0.38 - 0.87) | Hundley et al., 2013 [66] | The RR used is **lower** than the RR published in Gallos et al., 2018 (0.75) for PPH>500ml for mixed cohorts of birth and is just **outside the 95% CI.**  The RR used is **similar** than the RR published in Gallos et al., 2019 (0.61) for PPH>500ml for mixed cohorts of births. | The discrepancies between the effectiveness estimates used and the latest figures in systematic reviews may have resulted in this study **overestimating** the effectiveness of misoprostol relative to placebo in preventing PPH in the sensitivity analysis which used data from Hundley et al., 2013 [66].  This may have resulted in misoprostol appearing **more cost-effective** relative to placebo in this sensitivity analysis. |
|  | Alternative RR used in scenario analysis:  RR of severe PPH with misoprostol vs placebo | 0.20 (0.04 - 0.91) | Hundley et al., 2013 [66] | The RR used is **lower** than the RR published in Gallos et al., 2018 (0.73) for PPH>1000ml for mixed cohorts of births and is **outside the 95% CI.**  The RR used is **lower** than the RR published in Gallos et al., 2019 (0.73) for PPH>1000ml for mixed cohorts of births and is **outside the 95% CI**. | The discrepancies between the effectiveness estimates used and the latest figures in systematic reviews may have resulted in this study **overestimating** the effectiveness of misoprostol relative to placebo in preventing PPH>1000ml in the sensitivity analysis which used data from Hundley et al., 2013 [66].  This may have resulted in misoprostol appearing **more cost-effective** relative to placebo in this sensitivity analysis. |
|  | Alternative RR used in scenario analysis:  RR of requiring additional uterotonics with misoprostol vs placebo | 0.34 (0.16 - 0.73) | Hundley et al., 2013 [66] | The RR used is **lower** than the RR published in Gallos et al., 2018 (0.67) for requiring additional uterotonics for mixed cohorts of births and is **outside the 95% CI.**  The RR used is **lower** than the RR published in Gallos et al., 2019 (0.43) for requiring additional uterotonics for mixed cohorts of births but is within the 95% CI. | The discrepancies between the effectiveness estimates used and the latest figures in systematic reviews may have resulted in this study **overestimating** the effectiveness of misoprostol relative to placebo in preventing the requirement for additional uterotonics in the sensitivity analysis which used data from Hundley et al., 2013 [66].  This may have resulted in misoprostol appearing **more cost-effective** relative to placebo in this sensitivity analysis. |
|  | Alternative RR used in scenario analysis:  RR of requiring blood transfusion with misoprostol vs placebo | 0.16 (0.07 - 0.38) | Hundley et al., 2013 [66] | The RR used is **lower** than the RR published in Gallos et al., 2018 (0.46) for requiring transfusion for mixed cohorts of births but is just within the 95% CI.  The RR used is **lower** than the RR published in Gallos et al., 2019 (0.49) for requiring additional uterotonics for mixed cohorts of births and is **outside the 95% CI**. | The discrepancies between the effectiveness estimates used and the latest figures in systematic reviews may have resulted in this study **overestimating** the effectiveness of misoprostol relative to placebo in preventing the need for transfusion in the sensitivity analysis which used data from Hundley et al., 2013 [66].  This may have resulted in misoprostol appearing **more cost-effective** relative to placebo in this sensitivity analysis. |
| **Fullerton et al., 2006 [67]**  Interventions:  1. AMTSL (uterotonic not specified)  2. EMTSL | Rate of PPH with AMTSL vs no AMTSL | AMTSL (no uterotonic specified): 5%  EMTSL: 10%  Calculated RR: 0.5 | McCormick et al., 2002 [68]  Prendiville et al., 1988 [69]  Rogers et al., 1998 [46]  Kahn et al., 1997 [70] | Active management may reduce the risk of PPH, but this is based on very low quality evidence [71]. | As no specific uterotonic is listed we cannot compare with RRs published in other Cochrane reviews. |
| **Dazelle et al., 2023 [72]**  Interventions:  1. Prophylactic tranexamic acid (1g) to all women  2. Prophylactic tranexamic acid (1g) to women at high risk of PPH  3. Prophylactic tranexamic acid (1g) to women at high or moderate risk of PPH  4. Routine care | RR of PPH with prophylactic tranexamic acid, VB | 0.55 (0.27 - 0.83) | Sentilhes et al., 2018 [73]  Gungorduk et al., 2013 [74]  Mirghafourvand et al., 2015 [75]  Bouet et al., 2015 [76]  Sadek et al., 2019 [77] | The RR used is **consistent** with the RR published in Novikova et al., 2015 (0.52) for PPH>500ml in mixed delivery.  The RR used is **higher** than the RR published in Li et al., 2017 (0.37) for PPH in VB but within the 95% CI. |  |
|  | RR of PPH with prophylactic tranexamic acid, CS | 0.40 (0.36 - 0.43) | Sadek et al., 2019 [77]  Gungorduk et al., 2011 [78]  Abd El-Gaber et al., 2018 [79] | The RR used is **higher** than the RR published in Li et al., 2017 for PPH (0.32), and severe PPH (0.31), and in Wang et al., 2019 for massive PPH (0.39) but is within the 95% CI of all three. |  |
| **Durand-Zaleski et al., 2021 [80]**  Interventions:  1. Tranexamic acid (1 gram slowly IV over 2 mins) in addition to standard care.   2. Placebo and standard care. | Observed number of PPH>500ml in VB | Tranexamic acid: 156 / 1918  Placebo: 188 / 1918  Calculated RR: 0.83 | Sentilhes et al., 2018 [73] | The RR used is **higher** than the RR published in Novikova et al., 2015 for mixed delivery (0.52) and the RR specifically for PPH in VB published in Li et al., 2018 (0.37) and is **outside of the 95% CI of both.** | The discrepancies between the effectiveness estimates used and the latest figures in systematic reviews may have resulted in this study **underestimating** the effectiveness of tranexamic acid relative to placebo in preventing PPH>500ml in VB.  This may have resulted in tranexamic acid appearing **less cost-effective** relative to placebo in this analysis. |
|  | Observed number of PPH>1000ml in VB | Tranexamic acid: 47 / 1918  Placebo: 57 / 1918  Calculated RR: 0.82 | Sentilhes et al., 2018 [73] | The RR used is **higher** than the RR published in Novikova et al., 2015 for PPH>1000ml in mixed delivery (0.40) and is outside the 95% CI.  The RR used is **higher** than the RR specifically for “severe PPH” in VB published in Li et al., 2018 (0.33) however this estimate had considerable uncertainty (95% CI 0.3 - 3.17). | The discrepancies between the effectiveness estimates used and the latest figures in systematic reviews may have resulted in this study **underestimating** the effectiveness of tranexamic acid relative to placebo in preventing PPH>1000ml in VB.  This may have resulted in tranexamic acid appearing **less cost-effective** relative to placebo in this analysis. |
| **Sentilhes et al., 2023 [81]**  Interventions:  1. TXA in addition to standard care  2. Placebo in addition to standard care | The proportion of women without a complication up to day 90.  Complications included blood loss >1L, requiring red blood cell transfusion, deep vein thrombosis, pulmonary embolism, myocardial infarction, renal failure, or requiring re-admission. | Tranexamic acid: 70.7%  Placebo: 66.0% | Sentilhes et al., 2021 [82] | The composite complication outcome is not comparable to any outcomes listed in systematic reviews. |  |
| **Denison et al., 2019 [13,83]**  Intervention:  1.  Two puffs of GTN (400µg sublingual)  2. Placebo spray (sublingual) | N/A – GTN did not result in reduced requirement for manual removal of placenta or a reduction in blood loss. | N/A – GTN did not result in reduced requirement for manual removal of placenta or a reduction in blood loss. | Authorship team’s RCT – the GOT-IT trial [13,83]. | Non-randomised trials previously suggested that GTN may be effective in the treatment of retained placenta, a cause of PPH. However, the GOT-IT trial, which this economic evaluation is based on, showed that GTN was ineffective [13,83]. |  |
| **Sharma et al., 2023 [84]**  Intervention:  1. Negative intrauterine pressure suction device  integrated with AMTSL (with oxytocin)  2. AMTSL (with oxytocin) alone | Proportion with PPH | Negative intrauterine pressure suction device: 0.49%  AMTSL (with oxytocin) alone: 1.81% | Authorship team’s qualitive improvement study | There is uncertainty around the effectiveness of prophylactic use of negative intrauterine pressure suction devices. Large multicentre RCTs are required. |  |
| **Hong et al., 2022 [85]**  Interventions:  1. Internal iliac artery balloon occlusion prior to CS in patients with placenta accreta. Balloon inflated once baby delivered. Deflated after haemostasis achieved.   2. No occlusion before CS. | Estimated blood loss | Internal iliac artery balloon occlusion group: 2500ml (1750ml – 5750ml)  Control Group: 2000ml (1500ml – 3350ml) | Authorship team’s retrospective data collection | There is uncertainty around the effectiveness of prophylactic endovascular interventions to control haemorrhage in patients with placental implantation abnormalities [16,17].  One systematic review and meta-analysis on the topic concluded that endovascular intervention was effective at reducing haemorrhage in deliveries complicated by abnormal placental implantation [18], but the vast majority of included studies were not randomized and the conclusions may be open to significant bias. |  |
|  | Proportion requiring caesarean hysterectomy | Internal iliac artery balloon occlusion group: 43.48%  Control Group: 31.43% | Authorship team’s retrospective data collection | There is uncertainty around the effectiveness of prophylactic endovascular interventions to control haemorrhage in patients with placental implantation abnormalities [16,17]. |  |
| **Niola et al., 2017 [86]**  Interventions:  1. Intravascular uterine artery occlusion immediately before delivery in patients with placental implant abnormalities  2. Surgery or embolization after delivery | Proportion of patients requiring transfusion | Uterine artery occlusion group: 36%  Control Group: 100% | Authorship team’s retrospective data collection | There is uncertainty around the effectiveness of prophylactic endovascular interventions to control haemorrhage in patients with placental implantation abnormalities [16,17]. |  |
|  | Proportion requiring hysterectomy | Uterine artery occlusion group: 26%  Control Group: 43.4% | Authorship team’s retrospective data collection | There is uncertainty around the effectiveness of prophylactic endovascular interventions to control haemorrhage in patients with placental implantation abnormalities [16,17]. |  |
| **Xue et al., 2019 [87]**  Interventions:  1. MDT-ERAS intervention (a multimodal perioperative care pathway to achieve enhanced recovery after surgery) in patients undergoing CS.  2. Traditional perioperative care | Proportion with PPH | MDT-ERAS Group: 5.94%  Control Group: 17.13% | Authorship team’s RCT data | Enhanced recovery after surgery (ERAS) guidelines recommend certain surgical techniques to minimise surgical blood loss during CS but report the evidence as ‘moderate’ and the recommendation grade as ‘weak’[88-90].  Enhanced recovery after surgery (ERAS) guidelines postulate that controlling nausea and vomiting may help reduce post-surgical bleeding, but this is not referenced in the guideline [90].  No systematic review data on ERAS reducing PPH was identified to compare the effectiveness data of this study to. |  |

Calculated RR: The RR calculated from data in publication, not listed by the publication.

Abbreviations: AMTSL: Active management of the third stage of labour. ANC: Antenatal care. CI: Confidence interval. CS: Caesarean section. EMTSL: Expectant management of the third stage of labour. GTN: Glyceryl trinitrate. IM: Intramuscular. IU: International unit. IV: Intravenous. MDT-ERAS: Multidisciplinary enhanced recovery after surgery. PHC: Primary health care. PO: Per oral. PPH: Postpartum haemorrhage. RCT: Randomised control trial. RR: Risk ratio. TXA: Tranexamic acid. VB: Vaginal birth. VHW: Village health worker. WHO: World Health Organization. μg: Micrograms.

## **Diagnosis of PPH**

**Current WHO Recommendations on the diagnosis of PPH**

- Postpartum abdominal uterine tonus assessment for early identification of uterine atony for all women [3].
- For all women giving birth, routine objective measurement of postpartum blood loss is recommended to improve the detection and prompt treatment of postpartum haemorrhage. Methods to objectively quantify blood loss, such as calibrated drapes for women having vaginal birth, can achieve this [91].

**Effectiveness Evidence for diagnostic interventions included in this review**

**Gravimetric and Colorimetric measurement of blood loss vs visual estimation**

- One Cochrane review on the diagnosis of PPH was identified: Diaz et al., 2018 [92], however the only comparisons able to be included in the review were between direct measurement with calibrated drapes vs visual estimation and direct measurement with calibrated drapes vs gravimetric technique.
- The search for this systematic review was re-run on 14 June 2023, and although 1 additional study was identified, the overall estimates or certainty of effects did not change [91].
- No other relevant systematic reviews covering colorimetric or gravimetric measurement of blood loss (or combinations of the two methods) compared to visual estimation in PPH were identified.

Table P: Comparison of the effectiveness measures used in included economic evaluations on PPH diagnosis with the latest effectiveness data from systematic reviews and meta-analyses in the literature.

| **Study (Intervention/s & Comparator/s)** | **Outcome** | **Figure observed in study, or parameter used in model**  (variation or ranges used) | **Source of evidence cited in article** | **Consistency with current effectiveness evidence** | **Comments on significant discrepancies** |
| --- | --- | --- | --- | --- | --- |
| Katz et al., 2020 [93]  Intervention:  1. Triton system for blood measurement (gravimetric and colorimetric).   2. Visual estimation by obstetrician, nursing staff or anaesthetist | Proportion of deliveries diagnosed with PPH in VB | Triston system of blood measurement: 2.2%  Visual estimation: 0.5% | Authorship team’s retrospective data collection. | No systematic reviews were identified comparing gravimetric or colorimetric blood loss measurement (or combinations of both methods) with visual estimation in PPH. |  |
|  | Proportion of deliveries diagnosed with PPH in CS | Triston system of blood measurement: 12.6%  Visual estimation: 6.4% | Authorship team’s retrospective data collection. | No systematic reviews were identified comparing gravimetric or colorimetric blood loss measurement (or combinations of both methods) with visual estimation in PPH. |  |

Abbreviations: CS: caesarean section. PPH: postpartum haemorrhage. VB: Vaginal birth.

## **Treatment of PPH**

**Current WHO Recommendations on the treatment of PPH**

**Treatment (Initial)**

- A standardized and timely approach to the management of postpartum haemorrhage (PPH), comprising an objective assessment of blood loss and use of a treatment bundle supported by an implementation strategy, is recommended for all women having a vaginal birth. The care bundle for first-line treatment of PPH should include rapid institution of uterine massage, administration of an oxytocic agent and tranexamic acid, intravenous fluids, examination of the genital tract and escalation of care [91].
- IV oxytocin as first-line treatment for PPH [3].
- If IV oxytocin is unavailable, or if the bleeding does not respond to oxytocin, the use of intravenous ergometrine, oxytocin-ergometrine fixed dose, or a prostaglandin drug (including sublingual misoprostol, 800 μg) is recommended [3].
- Early IV tranexamic acid (within 3 hours of birth) in addition to standard care for women with PPH for all births [94].
- IV/IM oxytocin (10 IU) in combination with controlled cord traction if the placenta is not expelled spontaneously [3].
- Single dose of antibiotics (ampicillin or first-generation cephalosporin) if manual removal of the placenta is practiced [3].
- Isotonic crystalloids in preference to colloids for the initial intravenous fluid resuscitation of women with PPH [3].

**Treatment (Refractory)**

- Uterine massage for the treatment of PPH [3].
- Bimanual uterine compression as a temporizing measure until appropriate care is available for PPH due to uterine atony after vaginal birth [3].
- External aortic compression as a temporizing measure until appropriate care is available for PPH due to uterine atony after vaginal birth [3].
- Uterine balloon tamponade (UBT) for treatment of postpartum haemorrhage due to uterine atony after vaginal birth in women who do not respond to standard first-line treatment provided certain conditions are met [95].
- Non-pneumatic anti-shock garment as a temporizing measure until appropriate care is available [3].
- Uterine artery embolization for treatment for PPH due to uterine atony if other measures have failed and resources are available [3].
- Surgical interventions if bleeding does not stop despite treatment with uterotonic treatment and other available conservative interventions (e.g., uterine massage, balloon tamponade) [3].
- Umbilical vein injection of oxytocin is recommended for the treatment of retained placenta only in the context of rigorous research [96].

**Health System Interventions**

- Formal protocols by health facilities for the prevention and treatment of PPH [3].
- Formal protocols for referral of women to a higher level of care for health facilities [3].
- Simulations of PPH treatment for pre-service and in-service training programmes [3].
- Monitoring use of uterotonics after birth for the prevention of PPH as a process indicator for programmatic evaluation [3].

**Effectiveness evidence for treatment interventions included in this review**

**Misoprostol vs Placebo for treatment of PPH**

- Two Cochrane reviews include an analysis on the effectiveness of misoprostol for the treatment of PPH:
  - Mousa et al., 2014: Treatment for primary postpartum haemorrhage [97].
  - Parry Smith et al., 2020: Uterotonic agents for first‐line treatment of postpartum haemorrhage: a network meta‐analysis [98].

However, both reviews compared misoprostol to the use of other uterotonics, and mainly in women who had already received conventional uterotonic therapy as prevention. The economic evaluations we identified assessing the use of misoprostol for first line PPH treatment were in women that had no received prophylaxis, and compared to those with no uterotonic for treatment.

**Tranexamic Acid**

- One Cochrane review on the effectiveness of tranexamic acid for the treatment of PPH was identified:
  - Shakur et al., 2018: Antifibrinolytic drugs for treating primary postpartum haemorrhage [99].
  - The majority of the data in this review is from the WOMAN trial [100].
- Effectiveness estimates from this review are presented below:

Table Q: Effectiveness estimates from systematic reviews - Tranexamic acid plus standard care vs standard care alone

| **Outcome** | **RR (95% CI)** | **Source** |
| --- | --- | --- |
| RR of death due to PPH | 0.81 (0.66 - 1.0) | Shakur et al., 2018 [99] |
| RR of death due to PPH  (early administration <1 hour) | 0.80 (0.55 - 1.16) * | Shakur et al., 2018 [99] |
| RR of death due to PPH  (early administration 1-3 hours) | 0.6 (0.41 - 0.88) * | Shakur et al., 2018 [99] |
| RR of all-cause mortality | 0.88 (0.74 - 1.05) | Shakur et al., 2018 [99] |
| RR of all-cause mortality  (early administration <1 hour) | 0.98 (0.72 - 1.33) * | Shakur et al., 2018 [99] |
| RR of all-cause mortality  (early administration 1 - 3 hours) | 0.69 (0.49 - 0.96) * | Shakur et al., 2018 [99] |
| RR of requiring laparotomy to control bleeding | 0.64 (0.49 - 0.85) * | Shakur et al., 2018 [99] |
| RR of requiring brace sutures | 1.19 (1.01 - 1.41) * | Shakur et al., 2018 [99] |

* All data in this calculation was from the WOMAN trial. [100]
Abbreviations: CI: Confidence Interval. PPH: Postpartum haemorrhage. RR: Relative risk.

**Non-pneumatic Anti-shock Garment (NASG)**

- There are no Cochrane reviews on the effectiveness of NASG for the management of PPH or obstetric haemorrhage.
- A systematic review on the effectiveness of NASG for the management of PPH was published in 2015 and included 6 studies, one of which was a randomised control trial [101].
- Effectiveness estimates from this review are presented below:

Table R: Effectiveness estimates from systematic reviews - Non-pneumatic anti-shock garment vs standard care

| **Outcome** | **RR (95% CI)** | **Source** |
| --- | --- | --- |
| RR of mortality in NASG vs standard care | 0.52 (0.36 - 0.77) | Pileggi-Castro et al., 2015 [101]  (Estimate based on non-randomised trials) |
|  | 0.43 (0.14 - 1.33) | Pileggi-Castro et al., 2015 [101]  (Estimate based on 1 cluster RCT) |

Abbreviations: CI: Confidence Interval. NASG: Non-pneumatic anti-shock garment. RCT: Randomised control trial RR: Relative risk.

**Uterine Balloon Tamponade**

- One Cochrane review including an analysis of the effectiveness of uterine balloon tamponade devices was identified [102].
  - Kellie et al., 2020: Mechanical and surgical interventions for treating primary postpartum haemorrhage [102].
    - Only randomised control trials were included in the analysis.
    - Meta-analysis could not be performed due to heterogeneity.
    - There was insufficient evidence to determine the relative effectiveness of mechanical and surgical interventions for PPH, including uterine balloon tamponade.
- An alternative systematic review, which included non-randomised trial data was identified:
  - Suarez et al., 2020: Uterine balloon tamponade for the treatment of postpartum haemorrhage: a systematic review and meta-analysis [103].
    - Data from 91 studies was included: 6 randomized trials, 1 cluster randomized trial, 15 nonrandomized studies, and 69 case series.
    - Effectiveness estimates from this review are presented below:

Table S: Effectiveness estimates from systematic reviews – Uterine balloon tamponade

| **Outcome** | **Measure (95% CI)** | **Source** |
| --- | --- | --- |
| Success rate of uterine balloon tamponade in arresting PPH | 85.9% (83.9 - 87.9) | Suarez et al., 2020 [103] |
| Subgroup Analysis: Success rate of Condom UBT in arresting PPH | 90.4% (87.7 - 92.8%) | Suarez et al., 2020 [103] |
| Subgroup Analysis: Success rate of Bakri UBT in arresting PPH | 83.2% (80.5 - 85.8%) | Suarez et al., 2020 [103] |

Abbreviations: CI: Confidence Interval. PPH: Postpartum haemorrhage. RR: Relative risk. UBT: Uterine balloon tamponade.

**Thromboelastography (TEG) and rotational thromboelastometry (ROTEM) guided resuscitation**

- There are no Cochrane reviews on TEG or ROTEM use in obstetrics.
- One Cochrane review on TEG and ROTEM use in adult trauma patients was published in 2015 [104]. There was insufficient evidence at the time to promote the use of TEG or ROTEM outside of research [104].
- A systematic review on TEG and ROTEM use specifically in obstetrics was published in 2020 [105].
  - The review identified three studies which supported ROTEM guided blood product resuscitation for the management of PPH, reporting a reduction in the volume of blood products transfused when using ROTEM guided resuscitation [106-108].
- An additional systematic review and metanalysis of TEG and ROTEM use specifically in primary postpartum haemorrhage management was published in 2023 [109].
  - This review included 5 studies and provided pooled estimates of the odds of requiring hysterectomy, ICU, and various transfusions when being treatment is informed by viscoelastic testing (see below).

Table T: Impact of viscoelastic guided resuscitation in PPH

| **Outcome** | **Measure (95% CI)** | **Source** |
| --- | --- | --- |
| Odds or requiring hysterectomy | OR: 0.55 (0.32 - 0.95) | Khanna et al., 2023 [109] |
| Odds of requiring ICU | OR: 0.77 (0.46 - 1.29) | Khanna et al., 2023 [109] |

Abbreviations: CI: Confidence Interval. ICU: Intensive care unit. OR: Odds ratio.

**Blood Type and Screen Strategies**

- There are no Cochrane reviews on the effectiveness of type and screen strategies in obstetrics.
- No other systematic reviews summarising the effectiveness of selective or universal type and screen testing before delivery were identified.

**Blood transfusion for PPH or postpartum Anaemia**

- A 2015 Cochrane review on the management of postpartum anaemia only identified a single study for the comparison between conservative management and blood transfusion [110]. The study included in the Cochrane review is the same study that the included cost-effectiveness analysis in our review (Prick et al., 2014 [111]) used for effectiveness data.
- Another systematic review published in 2024 [112], also compared the use of blood transfusion to conservative management, or iron supplementation, but as with the previous review, the only effectiveness data for the comparison between transfusion and conservative management came from the non-inferiority trial completed by Prick and colleagues [113].

**Cell salvage**

- There are no Cochrane reviews specifically on obstetric cell salvage.
- A 2023 Cochrane review on cell salvage during elective surgery [114] included only one RCT in the obstetric population, the SALVO trial [115].
  - The review suggested that there may be no difference in the risk of receiving an allogenic transfusion among those undergoing cell salvage compared to those who do.
- A 2022 systematic review on cell salvage in women at high risk of PPH undergoing caesarean section found that cell salvage significantly reduced the rates of allogenic blood transfusion reactions (but did not report the overall number of blood transfusions avoided by introducing cell salvage [116].
- Another systematic review on the use of cell salvage during caesarean section reported that patients undergoing cell salvage had significantly lower odds of receiving an allogenic transfusion (OR 0.32, 95% CI 0.23 - 0.46) [117]. This review pooled data from the SALVO trial and three other significantly smaller trials to produce this estimate.

**Treatment algorithms**

- There are no Cochrane reviews on the effectiveness of introducing PPH management guidelines.
- A 2014 systematic review explored whether introducing management guidelines had an impact on the prevention, diagnosis or treatment of PPH [118]. It identified 6 cohort studies and 1 RCT.
  - 4 of the 7 included studies reported that introducing management guidelines reduced the incidence of PPH, however the contents of the guidelines, the context of what interventions were already in place, and the definitions of PPH differed among the studies.

**Transfer to higher level care**

- There are no Cochrane reviews on the effectiveness of transferring PPH patients to higher level care.
- We did not identify any systematic reviews on the impact of specific transfer models.

Table U: Comparison of the effectiveness measures used in included economic evaluations on PPH treatment with the latest effectiveness data from systematic reviews and meta-analyses in the literature.

| **Study (Intervention/s & Comparator/s)** | **Outcome** | **Figure observed in study, or parameter used in model**  (variation or ranges used) | **Source of evidence cited in article** | **Consistency with current effectiveness evidence** | | **Comments on significant discrepancies** |
| --- | --- | --- | --- | --- | --- | --- |
| **Bradley et al., 2007 [119]**  Intervention:  1. Training of TBAs to recognize PPH and treat with misoprostol (1000µg PR).   2. TBA attends birth but refers patient to hospital if PPH occurs. | Proportion with PPH that progress to “severe PPH >750ml” | Treated with misoprostol: 1.8% (0.9% - 5.0%)  Standard care: 18.2% (9% - 34.2%)  Calculated RR: 0.10 | Hofmeyr et al., 2004 [120]  Prata et al., 2005 [121]  Walraven et al., 2004 [122] | No systematic review evidence to compare to.  There were no comparisons of misoprostol vs placebo in women who had not already received conventional uterotonic therapy in Cochrane reviews published in 2014 and 2020 [97,98]. | |  |
|  | Proportion with severe PPH that require blood transfusion | Treated with misoprostol: 12.5% (10.0% - 15.0%)  Standard care: 22.2% (17.8% - 26.7%)  Calculated RR: 0.56 | Prata et al., 2005 [121] | No systematic review evidence to compare to.  There were no comparisons of misoprostol vs placebo in women who had not already received conventional uterotonic therapy in Cochrane reviews published in 2014 and 2020 [97,98]. | |  |
| **Sutherland et al., 2010 [56]**  Intervention:  1. Misoprostol Prevention:  600μg PO. If patient has haemorrhage >1000ml they have 75% chance of referral to health centre.   2. Misoprostol Treatment:  800μg sublingual after 700ml blood loss.  3. Standard care with unskilled assistant and no medication | Number of women that die from PPH | Misoprostol treatment: 3.97 deaths per 10,000 deliveries  Unskilled assistant with no medication: 13.38 deaths per 10,000 deliveries  Calculated RR: 0.30 | Blum 2009 [123] | No systematic review evidence to compare to.  There were no comparisons of misoprostol vs placebo in women who had not already received conventional uterotonic therapy in Cochrane reviews published in 2014 and 2020 [97,98]. | |  |
|  | Number of women with severe anaemia following delivery | Misoprostol treatment given if PPH: 405 per 10,000 deliveries  Unskilled assistant with no medication: 428 per 10,000 deliveries  Calculated RR: 0.95 | Unclear source | No systematic review evidence to compare to.  There were no comparisons of misoprostol vs placebo in women who had not already received conventional uterotonic therapy in Cochrane reviews published in 2014 and 2020 [97,98]. | |  |
| **Howard et al., 2022 [124]**  Intervention:  1. Tranexamic acid (1g) plus standard management   2. Standard care | RR of death due to bleeding in tranexamic acid group compared to standard care | 0.78  Or if early administration: 0.69 | WOMAN trial [100] | The RR used in the model for both timings of administration are **similar** to the RR estimates in Shakur et al., 2018 [99] (0.81 & 0.60 respectively) and are within the 95% CI of both. | |  |
|  | RR of requiring surgical intervention to control PPH (exploratory laparotomy) in tranexamic acid group compared to standard care | 0.64  Or if early administration: 0.50 | WOMAN trial [100] | The RR used in the model is identical to the RR published in Shakur et al., 2018 [99] | |  |
| **Sudhof et al., 2019 [125]**  Intervention:  1. Tranexamic acid given at any time.  2. Tranexamic acid given within 3 hours of delivery.  3. Standard care (no tranexamic acid) | RR of laparotomy with tranexamic acid | 0.64 (0.49 - 0.85) | WOMAN trial [100] | The RR used in the model is identical to the RR published in Shakur et al., 2018 [99] | |  |
|  | RR of laparotomy with tranexamic acid in <3 h | 0.51 (0.29 - 0.95) | WOMAN trial [100] | The RR used in the model is not reported in the systematic review Shakur et al., 2018 [99]. It appears to be from a calculation combining the RR of requiring laparotomy in tranexamic acid given <1 h since delivery (0.48) and between 1-3 hours since delivery (0.54) with the data taken from Figure 4 in the WOMAN trial [100]. | |  |
|  | RR of brace sutures with tranexamic acid | 1.19 (1.01 - 1.41) | WOMAN trial [100] | The RR used in the model is identical to the RR published in Shakur et al., 2018 [99] | |  |
|  | RR of brace sutures with tranexamic acid  in <3 h | 1.19 (1.01 - 1.41) | WOMAN trial [100] | The RR used in the model is not reported in the systematic review Shakur et al., 2018 [99]. They appear to have used the general RR of requiring brace sutures for both early administration and any administration of tranexamic acid. | |  |
|  | RR of PPH mortality with tranexamic acid | 0.81 (0.65 - 1.0) | WOMAN trial [100] | The RR used in the model is **identical** to the RR published in Shakur et al., 2018 [99]. | |  |
|  | RR of PPH mortality with tranexamic acid  in <3 h | 0.69 (0.52–0.91) | WOMAN trial [100] | The RR used in the model is **consistent** with the RR estimates published in Shakur et al., 2018 [99] – a combination of the RR reported for early administration <1 hour (0.80) and the RR reported for administration 1-3 hours (0.60). | |  |
| **Joshi et al., 2023 [126]**  Intervention:  1. Tranexamic acid (1g IV within 3 hours of birth) plus standard care. An additional dose of tranexamic acid was given if bleeding continued after 30 min or if it restarted within 24 h.   2. Standard care | RR of death due to bleeding  in tranexamic acid group compared to standard care | 0.69 (0.52 - 0.91) | Calculated from WOMAN trial [100] | The RR used in the model is **consistent** with the RR estimates published in Shakur et al., 2018 [99] – a combination of the RR reported for early administration <1 hour (0.80) and the RR reported for administration 1-3 hours (0.60). The calculation performed in the supplementary file of Joshi et al., 2023 [126] is based on the same data as the systematic review: the WOMAN trial [100]. | |  |
|  | RR of all-cause mortality in tranexamic acid group compared to standard care | 0.88 (0.74 - 1.05) | Calculated from WOMAN trial [100] | The RR used in the model is **consistent** with the RR estimates published in Shakur et al., 2018 [99] – a combination of the RR reported for early administration <1 hour (0.98) and the RR reported for administration 1-3 hours (0.69). The calculation performed in the supplementary file of Joshi et al., 2023 [126] is based on the same data as the systematic review: the WOMAN trial [100]. | |  |
| **Li et al., 2018 [127]**  Intervention:  1. Tranexamic acid plus routine care  2. Placebo plus routine care | RR of death due to bleeding  with tranexamic acid given within 3 h | 0.69 (0.52 - 0.91) | WOMAN trial [100] | The RR used in the model is **consistent** with the RR estimates published in Shakur et al., 2018 [99] – a combination of the RR reported for early administration <1 hour (0.80) and the RR reported for administration 1-3 hours (0.60). | |  |
| **Downing et al., 2015 [128]**  Intervention:  1. Application of the NASG at the primary health care centre.  2. Delaying application of NASG until the patient arrives at the referral hospital. | Mortality | Early Application at primary health: 3 of 366  Delayed application: 12 of 466 | El Ayadi et al., 2014 [129] | No systematic review evidence to compare to.  The systematic review identified on NASG effectiveness did not report on the comparative effectiveness of early vs late application [101]. | |  |
| **Sutherland et al., 2013 [130]**  Intervention:  1. Adding NASG to standard management of women with severe hypovolemic shock (MAP<60mmHg) due to obstetric haemorrhage.   2. Adding NASG to standard management of women with any degree of shock due to obstetric haemorrhage.   3. Standard care with no NASG | Rate of emergency hysterectomy (per 1000 women) | **Egypt**  All patients  NASG: 32.26  No NASG: 34.01  Patients with severe shock  NASG 56.60  No NASG 180.56  **Nigeria**  All patients  NASG: 3.62  No NASG: 0  Patients with severe shock  NASG 4.65  No NASG 0 | Miller et al., 2010 [131] | There is limited data on the effectiveness of NASG on reducing hysterectomy rates.  The relevant systematic review (Pileggi-Castro et al., 2015 [101]) did not publish a RR for this outcome. | |  |
|  | Proportion with severe morbidity | **Egypt**  All patients  NASG: 0.9%  No NASG: 4.1%  Calculated RR: 0.22  Patients with severe shock  NASG 3.1%  No NASG 16.7%  Calculated RR: 0.19  **Nigeria**  All patients  NASG: 0.4%  No NASG: 2.7%  Calculated RR: 0.15  Patients with severe shock  NASG 0%  No NASG 4.7%  Calculated RR: 0 | Miller et al., 2010 [131] | There is limited data on the effect of NASG on severe maternal morbidity.  The relevant systematic review (Pileggi-Castro et al., 2015 [101]) did not publish a RR for this outcome. | |  |
|  | **Proportion mortality** | **Egypt**  All patients  NASG: 1.1%  No NASG: 2.3%  Calculated RR: 0.48  Patients with severe shock  NASG 5.7%  No NASG 12.5%  Calculated RR: 0.46  **Nigeria**  All patients  NASG: 8.7%  No NASG: 16.0%  Calculated RR: 0.54  Patients with severe shock  NASG 9.3%  No NASG 22.0%  Calculated RR: 0.42 | Miller et al., 2010 [131] | | There is limited data on the effect of NASG on maternal mortality.  The mortality data used from Miller et al., 2010 [131] to inform the model in this study is **similar** to the RRs published in Pileggi-Castro et al., 2015 [101], whether comparing to the RR combining estimates from 5 non-randomised trials (0.52) or from one cluster randomised control trial (0.43). |  |
| **Mvundura et al., 2017 [132]**  Intervention:  1. ESM-UBT plus standard care  2. Standard care without UBT or uterine packing   3. Standard care with uterine packing available | Effectiveness of uterine packing for stopping PPH if uterotonics and mechanical interventions fail | 60% (30% - 70%) | Assumption | | There is limited data on the effectiveness of uterine packing in the management of PPH.  A Cochrane review of RCTs on mechanical and surgical interventions for PPH, Kellie et al., 2020 was unable to produce pooled estimates on the effectiveness or relative effectiveness of intrauterine tamponade practices, including packing [102]. |  |
|  | Effectiveness of ESM-UBT for stopping PPH if uterotonics and mechanical interventions fail | 97% (85 – 99%) | Burke et al., 2016 [133] | | There is limited data on the effectiveness of uterine balloon tamponade in the management of PPH.  A Cochrane review of RCTs on mechanical and surgical interventions for PPH, Kellie et al., 2020, was unable to produce pooled estimates on the effectiveness or relative effectiveness of intrauterine tamponade devices [102].  An alternative systematic review, which included data from non-randomised studies estimated the effectiveness of intrauterine balloon tamponade to be 85.9% (95% CI: 83.9 - 87.9) [103]. | The discrepancies between the effectiveness estimates used and the latest figures in systematic reviews may have resulted in this study **overestimating** the effectiveness of UBTs in treating PPH.  This may have resulted in ESM-UBTs appearing **more cost-effective** relative to uterine packing. |
| **Joshi et al., 2021 [134]**  Intervention:  1. ESM-UBT  2. Bakri®-UBT  3. Condom-UBT (improvised) which is standard care | Proportion of atonic PPH cases controlled after UBT insertion | Condom UBT: 0.923  Bakri UBT: 0.843  ESM UBT: 0.953 | Authorship team’s calculations from targeted literature review | | There is limited data on the effectiveness of uterine balloon tamponade in the management of PPH.  A Cochrane review of RCTs on mechanical and surgical interventions for PPH, Kellie et al., 2020, was unable to produce pooled estimates on the effectiveness or relative effectiveness of intrauterine tamponade devices [102].  An alternative systematic review was identified, which included data from non-randomised studies [103]. This review included a sub analysis on the effectiveness of Bakri balloon and condom UBT. Both the overall effectiveness figures for Condom UBT and Bakri UBT used in this cost-effectiveness analysis are **similar** to the pooled effectiveness estimates published in the systematic review (90.4% & 83.2% respectively) [103]. However, it is worth noting that there were discrepancies between the pooled estimated from RCTs and pooled estimates from non-randomized studies. |  |
| **Edwards et al., 2023 [135]**  Intervention:  1. Butterfly device (facilitates compression of uterus as an alternative to bimanual compression)   2. Standard care | Number of participants with >1000ml blood loss after device insertion | Butterfly device: 1 of 57  Standard care: 9 of 113 | Phase II clinical device study (Weeks et al., 2023 [136]) | | The effectiveness of this device has not yet been evaluated in a randomised control trial. |  |
| **Snegovskikh et al., 2018 [107]**  Intervention:  1. Blood product resuscitation guided by point of care viscoelastic testing (PCVT)  2. Empiric blood product resuscitation | Average estimated blood loss | PCVT: 2000ml  Standard care: 3000ml | Authorship team’s retrospective data collection | | A systematic review and metanalysis on TEG and ROTEM use in primary PPH management was identified but did not report on differences in blood loss [109]. |  |
|  | Number requiring hysterectomy | PCVT: 7 of 28  Standard care: 31 of 58  Calculated RR: 0.46  Calculated OR: 0.29 | Authorship team’s retrospective data collection | | The observed odds ratio of requiring hysterectomy is **lower** than the reported OR in Khanna et al., 2023 [109] (0.55), and is **outside the 95% CI.** | The discrepancies between the effectiveness data observed in this sample and the latest figures in systematic reviews may have resulted in this study **overstating** the effectiveness of PCVT in managing PPH.  This may have resulted in PCVT appearing **more cost-effective** relative to standard care. |
|  | Average length of hospitalisation after delivery | PCVT: 4 days  Standard care: 5 days | Authorship team’s retrospective data collection | | There is limited data on the impact of PCVT guided resuscitation on length of stay.  A systematic review and metanalysis on TEG and ROTEM use in primary PPH management was identified, but did not report on differences in average length of stay [109]. |  |
|  | Number requiring ICU admission | PCVT:1 of 28  Standard care: 25 of 58  Calculated OR: 0.05 | Authorship team’s retrospective data collection | | The observed odds ratio of requiring ICU admission in this study is **lower** than the reported OR in Khanna et al., 2023 [109] (0.77) and **outside of the 95% CI.** | The discrepancies between the effectiveness data observed in this study and the latest figures in systematic reviews may have resulted in this study **overstating** the effectiveness of viscoelastic guided resuscitation relative to empiric resuscitation in management of PPH.    This may have resulted in the intervention appearing **more cost-effective** relative to empiric management in this analysis. |
| **Einerson et al., 2017 [137]**  Intervention:  1. Universal type and screen plus cross match for high-risk patients   2. Universal type and screen only   3. Universal hold clot plus cross match for high-risk patients   4. Selective type and screen only in high-risk patients   5. No routine admission testing | RR of requiring an emergency release transfusion compared to those with a type and screen test | No routine testing group: 2.7  Hold clot group: 1.4  Crossmatch group: 0.15 | Mix of published data and authorship team’s patient level data from University of Utah. | | There is limited data on the effectiveness of transfusion preparedness in obstetrics. No systematic reviews on the effectiveness of type and screen strategies, or transfusion preparedness in obstetrics were identified to benchmark the effectiveness estimates used in this study. |  |
| **Prick et al., 2014 [111]**  Intervention:  1. RBC transfusion aiming for target Hb 8.9g/dl.  2. Conservative management (iron and or folic acid supplementation and only utilizing transfusion if clinically indicated). | HRQoL scores (physical fatigue, EQ-5D, and EQ-VAS scores) per day | RBC transfusion group compared to conservative management:  Physical fatigue: -0.58 per day  EQ-5D: +3.6 per day  EQ-VAS: +1.3 per day | Authorship team’s data from a multicentre non-inferiority  randomised trial [113]. | | There is limited data on the effectiveness of blood transfusions on the management of postpartum haemorrhage or postpartum anaemia.  Aside from the trial this model is based on, no further effectiveness evidence comparing RBC transfusion to conservative management was identified in systematic reviews [110]. |  |
| **Khan et al., 2018 [115]**  Intervention:  1. CS with cell salvage  2. CS without cell salvage | Probability that donor blood transfusion is avoided | Cell salvage group: 0.975  No cell salvage group: 0.965 | Authorship team’s RCT - The SALVO trial [115]. | | Estimates of the effectiveness of cell salvage in obstetrics found range depending on whether they are based only on high quality, large RCTs (which reports little to no difference in probability of receiving transfusion) [114] or they include data from smaller trials (cell salvage reduces the odds of transfusion - OR 0.32) [117]. |  |
| **Lim et al., 2018 [138]**  Intervention:  1. Cell salvage for all CS.  2. Cell salvage only for deliveries at high risk for haemorrhage (including placenta previa, placenta accreta, repeat CS or multiparity, chorioamnionitis, placental abruption, hypertensive disorders during pregnancy etc.)  3. No utilization of cell salvage. | Number of allogenic blood transfusions | Cell salvage group: -0.68 (‐0.88 to ‐0.49) units of RBC per patient compared to the standard care group. | Carless et al., 2010 [139]  (A 2010 Cochrane review on cell salvage during surgery. This version of the review included only cardiac, orthopaedic and vascular surgery patients. This review has since been updated in 2023 to include obstetric patients. | | Estimates of the effectiveness of cell salvage in obstetrics found range depending on whether they are based only on high quality, large RCTs (which reports little to no difference in probability of receiving transfusion) [114] or they include data from smaller trials (cell salvage reduces the odds of transfusion - OR 0.32) [117]. |  |
| **Ries et al., 2020 [140]**  Intervention:  1. Implementation of the “D-A-CH Handlungsalgorithmus Postpartale Blutung” algorithm for the management of PPH  2. PPH management prior to the implementation of the D-A-C-H algorithm. | Incidence of severe PPH | Intervention group: 2.55%  Control Group: 2.21% | Authorship team’s data collection – single centre retrospective case series. | | There is limited data on the effectiveness of introducing PPH management protocols.  One systematic review on the topic was identified [118], but no pooled estimate on the impact of treatment algorithms on the incidence of severe PPH was published.  Of the five included studies that analysed the number of severe PPH cases diagnosed before and after the management protocol intervention, three stated the intervention increased the proportion, and two studies stated that the interventions decreased the proportion [118]. |  |
|  | Number requiring RBC transfusion | Intervention group: 31 of 141  Control Group: 25 of 176 | Authorship team’s data collection – single centre retrospective case series. | | There is limited data on the effectiveness of introducing PPH management protocols.  One systematic review on the topic was identified [118], but no pooled estimate on the risk of transfusion was published. |  |
|  | Number requiring ICU | Intervention group: 23 of 141  Control Group: 14 of 176 | Authorship team’s data collection – single centre retrospective case series. | | There is limited data on the effectiveness of introducing PPH management protocols.  One systematic review on the topic was identified [118], but no pooled estimate on the risk of requiring ICU was published. |  |
| **Franke et al., 2024 [141]**  Intervention:  1. Referral and transport to secondary care facility  2. No referral (primary care only) | Probability of survival | Intervention group: 80% (range: 70%-90%)  Comparator group: 30% (range: 20% - 80%) | Besaina et al., 2019 [142]  And expert opinion [141] | | There is limited data on the effectiveness of interfacility transfer to higher level care.  No systematic reviews on the effectiveness of transfer strategies were identified to benchmark the effectiveness estimates used in this study. |  |

Calculated RR: The RR calculated from data in publication, not listed by the publication.
Calculated OR: The OR calculated from data in publication, not listed by the publication.
Abbreviations: CI: Confidence Interval. CS: Caesarean section. EQ-5D: EuroQol-5 Dimension. EQ-VAS: EuroQol Visual Analogue Scale. ESM-UBT: “Every second matters” uterine balloon tamponade. Hb: Haemoglobin. HRQoL: Health-Related Quality of Life. ICU: Intensive care unit. IV: Intravenous. MAP: Mean Arterial Pressure. NASG: Non-pneumatic Anti-shock Garment. OR: Odds ratio. PCVT: Point of Care Viscoelastic Testing. PO: Per oral. PPH: Postpartum haemorrhage. PR: Per Rectum. RBC: Red Blood Cell. RCT: Randomised control trial. ROTEM: Rotational thromboelastometry. RR: Relative risk. TBA: Traditional birth attendant. TEG: Thromboelastogram. UBT: Uterine balloon tamponade.

## **Bundles for the management of PPH**

**Current WHO Recommendations on PPH Management Bundles**

- A standardized and timely approach to the management of postpartum haemorrhage (PPH), comprising an objective assessment of blood loss and use of a treatment bundle supported by an implementation strategy, is recommended for all women having a vaginal birth. The care bundle for first-line treatment of PPH should include rapid institution of uterine massage, administration of an oxytocic agent and tranexamic acid, intravenous fluids, examination of the genital tract and escalation of care [91].

**Effectiveness Evidence for management bundles included in this review.**

A recent review of the effectiveness of management bundles was completed by Vogel and colleagues [143] prior to the publication of the *WHO recommendations on the assessment of postpartum blood loss and use of a treatment bundle for postpartum haemorrhage* in late 2023 [91]. The review identified evidence for two types of bundles:

- Treatment Bundles
  - 2 controlled studies and 7 uncontrolled studies were identified.
  - No meta-analysis was possible due to heterogeneity of bundle components, patient populations, and study designs.
  - There was high certainty evidence that the E-MOTIVE bundle [144] reduced the risk of severe PPH, composite severe maternal morbidity, the requirement for blood transfusions, the risk of PPH>500ml, and mean blood loss 2 hours and 24 hours postpartum.
  - Another combined management bundle evaluated in Barinov et al., 2017 provided low certainty evidence that the intervention reduced blood loss, and very low certainty evidence of reducing the rate of hysterectomy women undergoing caesarean section in Russia [145].
- Prevention and Treatment Bundles
  - Eleven studies of prevention and treatment bundles were identified – one controlled and 10 uncontrolled.
  - The cluster based, non-randomised study assessing the National Maternal Safety Consensus Bundle implemented by the California Maternal Quality Care Collaborative, produced low certainty evidence that the bundle reduces severe maternal morbidity (RR0.64, 95% CI 0.57 - 0.72) [146].
  - The 10 uncontrolled studies differed in bundle components, and study design, and none used statistical methods to account for pre-post correlation.

**Table V: Comparison of the effectiveness measures used in included economic evaluations on PPH management bundles with the latest effectiveness data from systematic reviews and meta-analyses in the literature.**

| **Study (Intervention/s & Comparator/s)** | **Outcome** | **Figure observed in study, or parameter used in model**  (variation or ranges used) | **Source of evidence cited in article** | **Consistency with current effectiveness evidence** | **Comments on significant discrepancies** |
| --- | --- | --- | --- | --- | --- |
| **Seim et al., 2023 [147]**  Intervention:  1. Standard care, plus: health worker education, misoprostol distribution for home deliveries, oxytocin for use in hospitals/health centres, semi-quantitative blood loss measurement, three step treatment for PPH and transfer for definitive management  2. Standard care same setting prior to the above nationwide intervention being launched. | Case fatality rate per 100 PPH cases | Intervention: 2.58% (2.18 - 3.03)  Control: 5.05% (3.36 - 7.30) | Authorship teams’ longitudinal data | There is currently only low certainty evidence that prevention plus treatment bundles for PPH lower maternal morbidity, and this is evidence is for the CMQCC bundle.  According to a recent systematic review on the effectiveness of PPH bundles, the effectiveness evidence used in this economic evaluation is from a non-randomised study, that did not use statistical methods to account for pre-post correlation [143]. |  |
|  | All cause maternal mortality per 100,000 births | Intervention: 446 (424 - 469)  Control: 681 (629 - 737) | Authorship teams’ longitudinal data | There is currently only low certainty evidence that prevention plus treatment bundles for PPH lower maternal morbidity, and this is evidence is for the CMQCC bundle.  According to a recent systematic review on the effectiveness of PPH bundles, the effectiveness evidence used in this economic evaluation is from a non-randomised study, that did not use statistical methods to account for pre-post correlation [143]. |  |
| **Wiesehan et al., 2023 [148]**  Intervention:  1. California's statewide perinatal quality collaborative initiative (see complete list of components in Table 1 of this study).  2. Standard care in Californian hospitals not taking part in the quality collaborative. | Absolute reduction in PPH-related severe maternal morbidity risk due to intervention | 0.142 or 14.2% | Main et al., 2017 [146] | The effectiveness evidence that this economic evaluation is based, from Main et al., 2017 [146] was the only controlled study of prevention plus treatment bundles identified in a recent systematic review [143]. The review reported that this study produced low certainty evidence that this bundle (the CMQCC bundle) reduces severe maternal morbidity. The study was rated as “high risk” for bias on the ROBINS-I tool. |  |
| **Dale et al., 2022 [149]**  Intervention:  1. Standard care, plus universal risk assessments for PPH, quantitative blood loss measurement, multidisciplinary team management of PPH, point of care coagulation blood testing after 1000mL blood loss to guide resuscitation.    2. Standard care in Wales prior to the above nationwide process improvements. | Number of PPH cases >2500ml | After intervention: 5.39/1000 maternities  Standard care (before intervention): 7.72/1000 maternities | Data from national quality improvement initiative – Bell et al., 2021 [150] | A recent systematic review of PPH management bundles reported that the interrupted time series study, which informed this cost-effectiveness analysis, ranked High on a risk of bias assessment using the ROBINS-I tool [143].  Results from other studies on treatment bundles were not able to be pooled due to heterogeneity of study population, bundle components, and study design, and therefore there is no benchmark to compare the reported effectiveness data to. |  |
|  | Number of women requiring RBC transfusion | After intervention: 19.91%  Standard care (before intervention): 26.42% | Data from national quality improvement initiative – Bell et al., 2021 [150] | A recent systematic review of PPH management bundles reported that the interrupted time series study, which informed this cost-effectiveness analysis, ranked High on a risk of bias assessment using the ROBINS-I tool [143].  Results from other studies on treatment bundles were not able to be pooled due to heterogeneity of study population, bundle components, and study design, and therefore there is no benchmark to compare the reported effectiveness data to. |  |
| **Williams et al., 2024 [151]**  Intervention:  1. Early PPH detection and treatment with: quantitative blood loss measurement, uterine massage, oxytocin, TXA, IV fluids, examination, and escalation to definitive management if needed, staff training and auditing, dedicated PPH-response trolley/case  2. Standard care in the respective hospitals and countries | Difference in risk of severe PPH between intervention and control groups | -2.6% (-3.1% to -2.1%) | Authorship teams’ RCT, the E-MOTIVE trial [144]. | A recent systematic review of PPH management bundles [143] reported that the E-MOTIVE trial, which provided the data for this cost-effectiveness analysis, provided high certainty evidence of reducing:   - The risk of severe PPH - The risk of composite severe maternal morbidity - The risk of requiring blood transfusion - Mean blood loss 2 and 24 hours postpartum.   Results from other studies on treatment bundles were not able to be pooled with data from the E-MOTIVE trial due to heterogeneity of study population, bundle components, and study design [143].  The authors of the review rated the risk of bias for this trial as Low using the RoB 2 tool [143]. |  |

Abbreviations: CMQCC: California Maternal Quality Care Collaborative. E-MOTIVE: Early Management of Postpartum Hemorrhage with Tranexamic Acid, Oxytocin, and Other Interventions. IV: Intravenous. PPH: Postpartum haemorrhage. RBC: Red blood cells. RCT: Randomised control trial. RoB2: Revised Cochrane risk-of-bias tool for randomized trials. ROBINS-I: Risk of bias tool for nonrandomised studies for interventions. TXA: Tranexamic acid.

# **References**

1. World Health Organization. WHO recommendations: uterotonics for the prevention of postpartum haemorrhage. Geneva: World Health Organization, 2018.

2. World Health Organization. WHO recommendation on routes of oxytocin administration for the prevention of postpartum haemorrhage after vaginal birth. Geneva: World Health Organization, 2020.

3. World Health Organization. WHO recommendations for the prevention and treatment of postpartum haemorrhage. Geneva: World Health Organization, 2012.

4. World Health Organization. WHO recommendation on Advance misoprostol distribution to pregnant women for prevention of postpartum haemorrhage. Geneva: World Health Organization, 2020.

5. Gallos ID, Papadopoulou A, Man R, Athanasopoulos N, Tobias A, Price MJ, et al. Uterotonic agents for preventing postpartum haemorrhage: a network meta‐analysis. Cochrane Database Syst Rev. 2018;(12). doi: 10.1002/14651858.CD011689.pub3.

6. Gallos I, Williams H, Price M, Pickering K, Merriel A, Tobias A, et al. Uterotonic drugs to prevent postpartum haemorrhage: a network meta-analysis. Health Technol Assess. 2019;23(9). doi: 10.3310/hta23090.

7. Salati JA, Leathersich SJ, Williams MJ, Cuthbert A, Tolosa JE. Prophylactic oxytocin for the third stage of labour to prevent postpartum haemorrhage. Cochrane Database Syst Rev. 2019;4(4):Cd001808. doi: 10.1002/14651858.CD001808.pub3.

8. Kalafat E, Gokce A, O'Brien P, Benlioglu C, Koc A, Karaaslan O, et al. Efficacy of carbetocin in the prevention of postpartum hemorrhage: a systematic review and Bayesian meta-analysis of randomized trials. J Matern Fetal Neonatal Med. 2021;34(14):2303–16. doi: 10.1080/14767058.2019.1664463.

9. Novikova N, Hofmeyr GJ, Cluver C. Tranexamic acid for preventing postpartum haemorrhage. Cochrane Database Syst Rev. 2015;(6). doi: 10.1002/14651858.CD007872.pub3.

10. Li C, Gong Y, Dong L, Xie B, Dai Z. Is prophylactic tranexamic acid administration effective and safe for postpartum hemorrhage prevention?: A systematic review and meta-analysis. Medicine (Baltimore). 2017;96(1):e5653. doi: 10.1097/md.0000000000005653.

11. Wang Y, Liu S, He L. Prophylactic use of tranexamic acid reduces blood loss and transfusion requirements in patients undergoing cesarean section: A meta-analysis. J Obstet Gynaecol Res. 2019;45(8):1562–75. doi: 10.1111/jog.14013.

12. Al-Dardery NM, Abdelwahab OA, Abouzid M, Albakri K, Elkhadragy A, Katamesh BE, et al. Efficacy and safety of tranexamic acid in prevention of postpartum hemorrhage: a systematic review and meta-analysis of 18,649 patients. BMC Pregnancy Childbirth. 2023;23(1):817. doi: 10.1186/s12884-023-06100-8.

13. Denison FC, Carruthers KF, Hudson J, McPherson G, Scotland G, Brook-Smith S, et al. Glyceryl trinitrate to reduce the need for manual removal of retained placenta following vaginal delivery: the GOT-IT RCT. Health Technol Assess. 2019;23(70):1–72. doi: 10.3310/hta23700.

14. Goffman D, Rood KM, Bianco A, Biggio JR, Dietz P, Drake K, et al. Real-World Utilization of an Intrauterine, Vacuum-Induced, Hemorrhage-Control Device. Obstet Gynecol. 2023;142(5). doi: 10.1097/AOG.0000000000005366.

15. Overton E, D’Alton M, Goffman D. Intrauterine devices in the management of postpartum hemorrhage. Am J Obstet Gynecol. 2024;230(3):S1076-S88. doi: 10.1016/j.ajog.2023.08.015.

16. Allen L, Jauniaux E, Hobson S, Papillon-Smith J, Belfort MA, Diagnosis ftFPA, et al. FIGO consensus guidelines on placenta accreta spectrum disorders: Nonconservative surgical management. Int J Gynaecol Obstet. 2018;140(3):281–90. doi: 10.1002/ijgo.12409.

17. Collins SL, Alemdar B, van Beekhuizen HJ, Bertholdt C, Braun T, Calda P, et al. Evidence-based guidelines for the management of abnormally invasive placenta: recommendations from the International Society for Abnormally Invasive Placenta. Am J Obstet Gynecol. 2019;220(6):511–26. doi: 10.1016/j.ajog.2019.02.054.

18. Shahin Y, Pang CL. Endovascular interventional modalities for haemorrhage control in abnormal placental implantation deliveries: a systematic review and meta-analysis. Eur Radiol. 2018;28(7):2713–26. doi: 10.1007/s00330-017-5222-0.

19. Pickering K, Gallos ID, Williams H, Price MJ, Merriel A, Lissauer D, et al. Uterotonic drugs for the prevention of postpartum haemorrhage: a cost-effectiveness analysis. Pharmacoecon Open. 2019;3:163–76. doi: 10.1007/s41669-018-0108-x.

20. Barrett J, Ko S, Jeffery W. Cost implications of using carbetocin injection to prevent postpartum hemorrhage in a Canadian urban Hospital. J Obstet Gynaecol Can. 2022;44(3):272–8. doi: 10.1016/j.jogc.2021.09.022.

21. Cook JR, Saxena K, Taylor C, Jacobs JL. Cost-effectiveness and budget impact of heat-stable carbetocin compared to oxytocin and misoprostol for the prevention of postpartum hemorrhage (PPH) in women giving birth in India. BMC Health Serv Res. 2023;23(1):267. doi: 10.1186/s12913-023-09263-4.

22. You JH, Leung T-y. Cost-effectiveness analysis of carbetocin for prevention of postpartum hemorrhage in a low-burden high-resource city of China. PLoS One. 2022;17(12):e0279130. doi: 10.1371/journal.pone.0279130.

23. Gil-Rojas Y, Lasalvia P, Hernández F, Castañeda-Cardona C, Rosselli D. Cost-effectiveness of Carbetocin versus Oxytocin for Prevention of Postpartum Hemorrhage Resulting from Uterine Atony in Women at high-risk for bleeding in Colombia. Rev Bras Ginecol Obstet. 2018;40:242–50. doi: 10.1055/s-0038-1655747.

24. Boucher M, Nimrod CA, Tawagi GF, Meeker TA, Rennicks White RE, Varin J. Comparison of carbetocin and oxytocin for the prevention of postpartum hemorrhage following vaginal delivery:a double-blind randomized trial. J Obstet Gynaecol Can. 2004;26(5):481–8. doi: 10.1016/s1701-2163(16)30659-4.

25. Borruto F, Treisser A, Comparetto C. Utilization of carbetocin for prevention of postpartum hemorrhage after cesarean section: a randomized clinical trial. Arch Gynecol Obstet. 2009;280(5):707–12. doi: 10.1007/s00404-009-0973-8.

26. Sheldon WR, Blum J, Vogel JP, Souza JP, Gülmezoglu AM, Winikoff B. Postpartum haemorrhage management, risks, and maternal outcomes: findings from the World Health Organization Multicountry Survey on Maternal and Newborn Health. BJOG. 2014;121 Suppl 1:5–13. doi: 10.1111/1471-0528.12636.

27. Briones JR, Talungchit P, Thavorncharoensap M, Chaikledkaew U. Economic evaluation of carbetocin as prophylaxis for postpartum hemorrhage in the Philippines. BMC Health Serv Res. 2020;20:1–12. doi: 10.1186/s12913-020-05834-x.

28. Luni Y, Borakati A, Matah A, Skeats K, Eedarapalli P. A prospective cohort study evaluating the cost-effectiveness of carbetocin for prevention of postpartum haemorrhage in caesarean sections. J Obstet Gynaecol Can. 2017;37(5):601–4. doi: 10.1080/01443615.2017.1284188.

29. Van Der Nelson HA, Draycott T, Siassakos D, Yau CW, Hatswell AJ. Carbetocin versus oxytocin for prevention of post-partum haemorrhage at caesarean section in the United Kingdom: an economic impact analysis. Eur J Obstet Gynecol Reprod Biol. 2017;210:286-91. doi: 10.1016/j.ejogrb.2017.01.004.

30. Su LL, Chong YS, Samuel M. Carbetocin for preventing postpartum haemorrhage. Cochrane Database Syst Rev. 2012;(4):Cd005457. doi: 10.1002/14651858.CD005457.pub4.

31. Wohling J, Edge N, Pena‐Leal D, Wang R, Mol BW, Dekker G. Clinical and financial evaluation of carbetocin as postpartum haemorrhage prophylaxis at caesarean section: A retrospective cohort study. Aust N Z J Obstet Gynaecol. 2019;59(4):501–7. doi: 10.1111/ajo.12907.

32. Caceda SI, Ramos RR, Saborido CM. Pharmacoeconomic study comparing carbetocin with oxytocin for the prevention of hemorrhage following cesarean delivery in Lima, Peru. J Comp Eff Res. 2018;7(1):49-55. doi: 10.2217/cer-2017-0012.

33. Henríquez-Trujillo AR, Lucio-Romero RA, Bermúdez-Gallegos K. Analysis of the cost–effectiveness of carbetocin for the prevention of hemorrhage following cesarean delivery in Ecuador. J Comp Eff Res. 2017;6(6):529–36. doi: 10.2217/cer-2017-0004.

34. Voon HY, Shafie AA, Bujang MA, Suharjono HN. Cost effectiveness analysis of carbetocin during cesarean section in a high volume maternity unit. J Obstet Gynaecol Res. 2018;44(1):109–16. doi: 10.1111/jog.13486.

35. Boucher M, Horbay GL, Griffin P, Deschamps Y, Desjardins C, Schulz M, et al. Double-blind, randomized comparison of the effect of carbetocin and oxytocin on intraoperative blood loss and uterine tone of patients undergoing cesarean section. J Perinatol. 1998;18(3):202–7.

36. Dansereau J, Joshi AK, Helewa ME, Doran TA, Lange IR, Luther ER, et al. Double-blind comparison of carbetocin versus oxytocin in prevention of uterine atony after cesarean section. Am J Obstet Gynecol. 1999;180(3 Pt 1):670–6. doi: 10.1016/s0002-9378(99)70271-1.

37. Attilakos G, Psaroudakis D, Ash J, Buchanan R, Winter C, Donald F, et al. Carbetocin versus oxytocin for the prevention of postpartum haemorrhage following caesarean section: the results of a double-blind randomised trial. BJOG. 2010;117(8):929–36. doi: 10.1111/j.1471-0528.2010.02585.x.

38. Razali N, Md Latar IL, Chan YK, Omar SZ, Tan PC. Carbetocin compared to oxytocin in emergency cesarean section: a randomized trial. Eur J Obstet Gynecol Reprod Biol. 2016;198:35–9. doi: 10.1016/j.ejogrb.2015.12.017.

39. El Behery MM, El Sayed GA, El Hameed AA, Soliman BS, Abdelsalam WA, Bahaa A. Carbetocin versus oxytocin for prevention of postpartum hemorrhage in obese nulliparous women undergoing emergency cesarean delivery. J Matern Fetal Neonatal Med. 2016;29(8):1257–60. doi: 10.3109/14767058.2015.1043882.

40. Whigham CA, Gorelik A, Loughnan TE, Trivedi A. Carbetocin versus oxytocin to reduce additional uterotonic use at non-elective caesarean section: a double-blind, randomised trial. J Matern Fetal Neonatal Med. 2016;29(23):3866–9. doi: 10.3109/14767058.2016.1149564.

41. Higgins L, Mechery J, Tomlinson A. Does carbetocin for prevention of postpartum haemorrhage at caesarean section provide clinical or financial benefit compared with oxytocin? J Obstet Gynaecol. 2011;31(8):732–9. doi: 10.3109/01443615.2011.595982.

42. Matthijsse S, Andersson FL, Gargano M, Yip Sonderegger YL. Cost-effectiveness analysis of carbetocin versus oxytocin for the prevention of postpartum hemorrhage following vaginal birth in the United Kingdom. J Med Econ. 2022;25(1):129–37. doi: 10.1080/13696998.2022.2027669.

43. Vlassoff M, Diallo A, Philbin J, Kost K, Bankole A. Cost-effectiveness of two interventions for the prevention of postpartum hemorrhage in Senegal. Int J Gynaecol Obstet. 2016;133(3):307–11. doi: 10.1016/j.ijgo.2015.10.015.

44. Diop A, Daff B, Sow M, Blum J, Diagne M, Sloan NL, et al. Oxytocin via Uniject (a prefilled single-use injection) versus oral misoprostol for prevention of postpartum haemorrhage at the community level: a cluster-randomised controlled trial. Lancet Glob Health. 2016;4(1):e37–44. doi: 10.1016/s2214-109x(15)00219-3.

45. Jose Diaz J, Jaramillo M. Evaluating interventions to reduce maternal mortality: evidence from Peru's PARSalud programme. J Dev Effect. 2009;1(4):387–412. doi: 10.1080/19439340903380872.

46. Rogers J, Wood J, McCandlish R, Ayers S, Truesdale A, Elbourne D. Active versus expectant management of third stage of labour: the Hinchingbrooke randomised controlled trial. Lancet. 1998;351(9104):693–9. doi: 10.1016/s0140-6736(97)09409-9.

47. Tsu VD, Levin C, Tran MP, Hoang MV, Luu HT. Cost-effectiveness analysis of active management of third-stage labour in Vietnam. Health Policy Plan. 2009;24(6):438–44. doi: 10.1093/heapol/czp020.

48. Tsu VD, Mai TT, Nguyen YH, Luu HT. Reducing postpartum hemorrhage in Vietnam: assessing the effectiveness of active management of third-stage labor. J Obstet Gynaecol Res. 2006;32(5):489–96. doi: 10.1111/j.1447-0756.2006.00436.x.

49. Pichon-Riviere A, Glujovsky D, Garay OU, Augustovski F, Ciapponi A, Serpa M, et al. Oxytocin in uniject disposable auto-disable injection system versus standard use for the prevention of postpartum hemorrhage in latin America and the Caribbean: a cost-effectiveness analysis. PLoS One. 2015;10(6):e0129044. doi: 10.1371/journal.pone.0129044.

50. Pichon-Riviere A, Glujovsky D, Garay OU, Augustovski F, Ciapponi A, Serpa M, et al. Correction: Oxytocin in Uniject Disposable Auto-Disable Injection System versus Standard Use for the Prevention of Postpartum Hemorrhage in Latin America and the Caribbean: A Cost-Effectiveness Analysis. PLOS ONE. 2015;10(7):e0133344. doi: 10.1371/journal.pone.0133344.

51. Cotter AM, Ness A, Tolosa JE. Prophylactic oxytocin for the third stage of labour. Cochrane Database Syst Rev. 2001;(4). doi: 10.1002/14651858.CD001808.

52. Carvalho N, Hoque ME, Oliver VL, Byrne A, Kermode M, Lambert P, et al. Cost-effectiveness of inhaled oxytocin for prevention of postpartum haemorrhage: a modelling study applied to two high burden settings. BMC Med. 2020;18(1):1–18. doi: 10.1186/s12916-020-01658-y.

53. Gajewska-Knapik K, Kumar S, Sutton-Cole A, Palmer KR, Cahn A, Gibson RA, et al. Pharmacokinetics and safety of inhaled oxytocin compared with intramuscular oxytocin in women in the third stage of labour: A randomized open-label study. Br J Clin Pharmacol. 2023;89(12):3681–9. doi: 10.1111/bcp.15860.

54. Sutherland T, Bishai DM. Cost-effectiveness of misoprostol and prenatal iron supplementation as maternal mortality interventions in home births in rural India. Int J Gynaecol Obstet. 2009;104(3):189–93. doi: 10.1016/j.ijgo.2008.10.011.

55. Derman RJ, Kodkany BS, Goudar SS, Geller SE, Naik VA, Bellad MB, et al. Oral misoprostol in preventing postpartum haemorrhage in resource-poor communities: a randomised controlled trial. Lancet. 2006;368(9543):1248–53. doi: 10.1016/s0140-6736(06)69522-6.

56. Sutherland T, Meyer C, Bishai DM, Geller S, Miller S. Community-based distribution of misoprostol for treatment or prevention of postpartum hemorrhage: cost-effectiveness, mortality, and morbidity reduction analysis. Int J Gynaecol Obstet. 2010;108(3):289–94. doi: 10.1016/j.ijgo.2009.11.007.

57. Goldie SJ, Sweet S, Carvalho N, Natchu UCM, Hu D. Alternative strategies to reduce maternal mortality in India: a cost-effectiveness analysis. PLoS Med. 2010;7(4):e1000264. doi: 10.1371/journal.pmed.1000264.

58. Hofmeyr GJ, Gülmezoglu AM. Misoprostol for the prevention and treatment of postpartum haemorrhage. Best Pract Res Clin Obstet Gynaecol. 2008;22(6):1025–41. doi: 10.1016/j.bpobgyn.2008.08.005.

59. Lubinga SJ, Atukunda EC, Wasswa-Ssalongo G, Babigumira JB. Potential cost-effectiveness of prenatal distribution of misoprostol for prevention of postpartum hemorrhage in Uganda. PLoS One. 2015;10(11):e0142550. doi: 10.1371/journal.pone.0142550.

60. Lubinga SJ, Atukunda EC, Wasswa-Ssalongo G, Babigumira JB. Correction: Potential Cost-Effectiveness of Prenatal Distribution of Misoprostol for Prevention of Postpartum Hemorrhage in Uganda. PLoS One. 2016;11(3):e0152955. doi: 10.1371/journal.pone.0152955.

61. Westhoff G, Cotter AM, Tolosa JE. Prophylactic oxytocin for the third stage of labour to prevent postpartum haemorrhage. Cochrane Database Syst Rev. 2013;(10). doi: 10.1002/14651858.CD001808.pub2.

62. Oladapo OT, Blum J, Abalos E, Okusanya BO. Advance misoprostol distribution to pregnant women for preventing and treating postpartum haemorrhage. Cochrane Database Syst Rev. 2020;6(6):Cd009336. doi: 10.1002/14651858.CD009336.pub2.

63. Prata N, Sreenivas A, Greig F, Walsh J, Potts M. Setting priorities for safe motherhood interventions in resource-scarce settings. Health Policy. 2010;94(1):1–13. doi: 10.1016/j.healthpol.2009.08.012.

64. Lang DL, Zhao F-L, Robertson J. Prevention of postpartum haemorrhage: cost consequences analysis of misoprostol in low-resource settings. BMC Pregnancy Childbirth. 2015;15(1):1–9. doi: 10.1186/s12884-015-0749-z.

65. Tunçalp Ö, Hofmeyr GJ, Gülmezoglu AM. Prostaglandins for preventing postpartum haemorrhage. Cochrane Database Syst Rev. 2012;2012(8):Cd000494. doi: 10.1002/14651858.CD000494.pub4.

66. Hundley VA, Avan BI, Sullivan CJ, Graham WJ. Should oral misoprostol be used to prevent postpartum haemorrhage in home-birth settings in low-resource countries? A systematic review of the evidence. BJOG. 2013;120(3):277–85; discussion 86–7. doi: 10.1111/1471-0528.12049.

67. Fullerton JT, Frick KD, Fogarty LA, Fishel JD, Vivio DM. Active management of third stage of labour saves facility costs in Guatemala and Zambia. J Health Popul Nutr. 2006;24(4):540.

68. McCormick ML, Sanghvi HC, Kinzie B, McIntosh N. Preventing postpartum hemorrhage in low-resource settings. Int J Gynaecol Obstet. 2002;77(3):267–75. doi: 10.1016/s0020-7292(02)00020-6.

69. Prendiville WJ, Harding JE, Elbourne DR, Stirrat GM. The Bristol third stage trial: active versus physiological management of third stage of labour. BMJ. 1988;297(6659):1295–300. doi: 10.1136/bmj.297.6659.1295.

70. Khan GQ, John IS, Wani S, Doherty T, Sibai BM. Controlled cord traction versus minimal intervention techniques in delivery of the placenta: a randomized controlled trial. Am J Obstet Gynecol. 1997;177(4):770–4. doi: 10.1016/s0002-9378(97)70266-7.

71. Begley CM, Gyte GML, Devane D, McGuire W, Weeks A, Biesty LM. Active versus expectant management for women in the third stage of labour. Cochrane Database of Syst Rev. 2019;(2). doi: 10.1002/14651858.CD007412.pub5.

72. Dazelle WD, Ebner MK, Kazma J, Potarazu SN, Ahmadzia HK. Tranexamic acid for the prevention of postpartum hemorrhage: a cost-effectiveness analysis. J Thromb Thrombolysis. 2023:1–9. doi: 10.1007/s11239-023-02814-w.

73. Sentilhes L, Winer N, Azria E, Sénat MV, Le Ray C, Vardon D, et al. Tranexamic Acid for the Prevention of Blood Loss after Vaginal Delivery. N Engl J Med. 2018;379(8):731–42. doi: 10.1056/NEJMoa1800942.

74. Gungorduk K, Asıcıoğlu O, Yıldırım G, Ark C, Tekirdağ A, Besımoglu B. Can intravenous injection of tranexamic acid be used in routine practice with active management of the third stage of labor in vaginal delivery? A randomized controlled study. Am J Perinatol. 2013;30(5):407–13. doi: 10.1055/s-0032-1326986.

75. Mirghafourvand M, Mohammad-Alizadeh S, Abbasalizadeh F, Shirdel M. The effect of prophylactic intravenous tranexamic acid on blood loss after vaginal delivery in women at low risk of postpartum haemorrhage: a double-blind randomised controlled trial. Aust N Z J Obstet Gynaecol. 2015;55(1):53–8. doi: 10.1111/ajo.12262.

76. Bouet PE, Ruiz V, Legendre G, Gillard P, Descamps P, Sentilhes L. High-dose tranexamic acid for treating postpartum haemorrhage after vaginal delivery. Br J Anaesth. 2015;114(2):339–41. doi: 10.1093/bja/aeu468.

77. Sadek S, Mahesan AM, Ramadan H, Dad N, Movva V, Kanaan C. Prophylactic tranexamic acid usage in prevention of post-partum hemorrhage a prospective cohort study2019.

78. Gungorduk K, Yıldırım G, Asıcıoğlu O, Gungorduk OC, Sudolmus S, Ark C. Efficacy of intravenous tranexamic acid in reducing blood loss after elective cesarean section: a prospective, randomized, double-blind, placebo-controlled study. Am J Perinatol. 2011;28(3):233–40. doi: 10.1055/s-0030-1268238.

79. Abd El-Gaber AE-N, Ahmed HH, Khodry MM, Abbas AM. Effect of tranexamic acid in prevention of postpartum hemorrhage in elective caesarean delivery: a randomized controlled study. Int J Reprod Contracept Obstet Gynecol. 2018;8(1):2. doi: 10.18203/2320-1770.ijrcog20185401.

80. Durand‐Zaleski I, Deneux‐Tharaux C, Seco A, Malki M, Frenkiel J, Sentilhes L, et al. An economic evaluation of tranexamic acid to prevent postpartum haemorrhage in women with vaginal delivery: the randomised controlled TRAAP trial. BJOG. 2021;128(1):114–20. doi: 10.1111/1471-0528.16456.

81. Sentilhes L, Bénard A, Madar H, Froeliger A, Petit S, Deneux-Tharaux C. Tranexamic acid for reduction of blood loss after Caesarean delivery: a cost-effectiveness analysis of the TRAAP2 trial. Br J Anaesth. 2023;131(5):893-900. doi: 10.1016/j.bja.2023.07.028.

82. Sentilhes L, Sénat MV, Le Lous M, Winer N, Rozenberg P, Kayem G, et al. Tranexamic Acid for the Prevention of Blood Loss after Cesarean Delivery. N Engl J Med. 2021;384(17):1623-34. doi: 10.1056/NEJMoa2028788.

83. Denison FC, Carruthers KF, Hudson J, McPherson G, Chua GN, Peace M, et al. Nitroglycerin for treatment of retained placenta: A randomised, placebo-controlled, multicentre, double-blind trial in the UK. PLoS Med. 2019;16(12):e1003001. doi: 10.1371/journal.pmed.1003001.

84. Sharma JC, Kollabathula P, Jindal S, Anupma A, Sarkar A, Jaggarwal S, et al. Application of a Negative Intrauterine Pressure Suction Device for Prophylactic Management of Atonic Postpartum Hemorrhage: A Quality Improvement Study. Cureus. 2023;15(7):e42631. doi: 10.7759/cureus.42631.

85. Hong L, Chen A, Chen J, Li X, Zhuang W, Shen Y, et al. The clinical evaluation of IIA balloon occlusion in caesarean delivery for patients with PAS: a retrospective study. BMC Pregnancy Childbirth. 2022;22(1):103. doi: 10.1186/s12884-022-04434-3.

86. Niola R, Giurazza F, Torbica A, Schena E, Silvestre M, Maglione F. Predelivery uterine arteries embolization in patients with placental implant anomalies: a cost-effective procedure. Radiol Med. 2017;122:77–9. doi: 10.1007/s11547-016-0690-x.

87. Xue L, Zhang J, Shen H, Hou Y, Ai L, Cui X. The application of rapid rehabilitation model of multidisciplinary cooperation in cesarean section and the evaluation of health economics. Zhonghua Yi Xue Za Zhi. 2019;99(42):3335–9. doi: 10.3760/cma.j.issn.0376-2491.2019.42.012.

88. Wilson RD, Caughey AB, Wood SL, Macones GA, Wrench IJ, Huang J, et al. Guidelines for Antenatal and Preoperative care in Cesarean Delivery: Enhanced Recovery After Surgery Society Recommendations (Part 1). Am J Obstet Gynecol. 2018;219(6):523.e1–.e15. doi: 10.1016/j.ajog.2018.09.015.

89. Caughey AB, Wood SL, Macones GA, Wrench IJ, Huang J, Norman M, et al. Guidelines for intraoperative care in cesarean delivery: Enhanced Recovery After Surgery Society Recommendations (Part 2). Am J Obstet Gynecol. 2018;219(6):533–44. doi: 10.1016/j.ajog.2018.08.006.

90. Macones GA, Caughey AB, Wood SL, Wrench IJ, Huang J, Norman M, et al. Guidelines for postoperative care in cesarean delivery: Enhanced Recovery After Surgery (ERAS) Society recommendations (part 3). Am J Obstet Gynecol. 2019;221(3):247.e1–.e9. doi: 10.1016/j.ajog.2019.04.012.

91. World Health Organization. WHO recommendations on the assessment of postpartum blood loss and use of a treatment bundle for postpartum haemorrhage. Geneva: World Health Organization, 2023.

92. Diaz V, Abalos E, Carroli G. Methods for blood loss estimation after vaginal birth. Cochrane Database Syst Rev. 2018;9(9):Cd010980. doi: 10.1002/14651858.CD010980.pub2.

93. Katz D, Wang R, O'Neil L, Gerber C, Lankford A, Rogers T, et al. The association between the introduction of quantitative assessment of postpartum blood loss and institutional changes in clinical practice: an observational study. Int J Obstet Anesth. 2020;42:4–10. doi: 10.1016/j.ijoa.2019.05.006.

94. World Health Organization. WHO recommendation on tranexamic acid for the treatment of postpartum haemorrhage. Geneva: World Health Organization, 2017.

95. World Health Organization. WHO recommendation on Uterine balloon tamponade for the treatment of postpartum haemorrhage. Geneva: World Health Organization, 2021.

96. World Health Organization. WHO recommendation on Umbilical vein injection of oxytocin for the treatment of retained placenta. Geneva: World Health Organization, 2020.

97. Mousa HA, Blum J, Abou El Senoun G, Shakur H, Alfirevic Z. Treatment for primary postpartum haemorrhage. Cochrane Database Syst Rev. 2014;2014(2):Cd003249. doi: 10.1002/14651858.CD003249.pub3.

98. Parry Smith WR, Papadopoulou A, Thomas E, Tobias A, Price MJ, Meher S, et al. Uterotonic agents for first-line treatment of postpartum haemorrhage: a network meta-analysis. Cochrane Database Syst Rev. 2020;11(11):Cd012754. doi: 10.1002/14651858.CD012754.pub2.

99. Shakur H, Beaumont D, Pavord S, Gayet-Ageron A, Ker K, Mousa HA. Antifibrinolytic drugs for treating primary postpartum haemorrhage. Cochrane Database Syst Rev. 2018;2(2):Cd012964. doi: 10.1002/14651858.Cd012964.

100. WOMAN Trial Collaborators. Effect of early tranexamic acid administration on mortality, hysterectomy, and other morbidities in women with post-partum haemorrhage (WOMAN): an international, randomised, double-blind, placebo-controlled trial. Lancet. 2017;389(10084):2105–16. doi: 10.1016/s0140-6736(17)30638-4.

101. Pileggi-Castro C, Nogueira-Pileggi V, Tunçalp Ö, Oladapo OT, Vogel JP, Souza JP. Non-pneumatic anti-shock garment for improving maternal survival following severe postpartum haemorrhage: a systematic review. Reprod Health. 2015;12:28. doi: 10.1186/s12978-015-0012-0.

102. Kellie FJ, Wandabwa JN, Mousa HA, Weeks AD. Mechanical and surgical interventions for treating primary postpartum haemorrhage. Cochrane Database Syst Rev. 2020;7(7):Cd013663. doi: 10.1002/14651858.Cd013663.

103. Suarez S, Conde-Agudelo A, Borovac-Pinheiro A, Suarez-Rebling D, Eckardt M, Theron G, et al. Uterine balloon tamponade for the treatment of postpartum hemorrhage: a systematic review and meta-analysis. Am J Obstet Gynecol. 2020;222(4):293.e1–.e52. doi: 10.1016/j.ajog.2019.11.1287.

104. Hunt H, Stanworth S, Curry N, Woolley T, Cooper C, Ukoumunne O, et al. Thromboelastography (TEG) and rotational thromboelastometry (ROTEM) for trauma induced coagulopathy in adult trauma patients with bleeding. Cochrane Database Syst Rev. 2015;2015(2):Cd010438. doi: 10.1002/14651858.CD010438.pub2.

105. Amgalan A, Allen T, Othman M, Ahmadzia HK. Systematic review of viscoelastic testing (TEG/ROTEM) in obstetrics and recommendations from the women's SSC of the ISTH. J Thromb Haemost. 2020;18(8):1813–38. doi: 10.1111/jth.14882.

106. Mallaiah S, Barclay P, Harrod I, Chevannes C, Bhalla A. Introduction of an algorithm for ROTEM-guided fibrinogen concentrate administration in major obstetric haemorrhage. Anaesthesia. 2015;70(2):166–75. doi: 10.1111/anae.12859.

107. Snegovskikh D, Souza D, Walton Z, Dai F, Rachler R, Garay A, et al. Point-of-care viscoelastic testing improves the outcome of pregnancies complicated by severe postpartum hemorrhage. J Clin Anesth. 2018;44:50–6. doi: 10.1016/j.jclinane.2017.10.003.

108. McNamara H, Kenyon C, Smith R, Mallaiah S, Barclay P. Four years' experience of a ROTEM(®) -guided algorithm for treatment of coagulopathy in obstetric haemorrhage. Anaesthesia. 2019;74(8):984–91. doi: 10.1111/anae.14628.

109. Khanna P, Sinha C, Singh AK, Kumar A, Sarkar S. The role of point of care thromboelastography (TEG) and thromboelastometry (ROTEM) in management of Primary postpartum haemorrhage: A meta-analysis and systematic review. Saudi J Anaesth. 2023;17(1):23–32. doi: 10.4103/sja.sja_529_22.

110. Markova V, Norgaard A, Jørgensen KJ, Langhoff‐Roos J. Treatment for women with postpartum iron deficiency anaemia. Cochrane Database of Systematic Reviews. 2015;(8). doi: 10.1002/14651858.CD010861.pub2.

111. Prick B, Duvekot J, Van Der Moer P, van Gemund N, Van Der Salm P, Jansen A, et al. Cost‐effectiveness of red blood cell transfusion vs. non‐intervention in women with acute anaemia after postpartum haemorrhage. Vox Sang. 2014;107(4):381–8. doi: 10.1111/vox.12181.

112. Caljé E, Groom KM, Dixon L, Marriott J, Foon R, Oyston C, et al. Intravenous iron versus blood transfusion for postpartum anemia: a systematic review and meta-analysis. Syst Rev. 2024;13(1):9. doi: 10.1186/s13643-023-02400-4. PubMed Central PMCID: PMCPMC10759729.

113. Prick BW, Jansen AJ, Steegers EA, Hop WC, Essink-Bot ML, Uyl-de Groot CA, et al. Transfusion policy after severe postpartum haemorrhage: a randomised non-inferiority trial. BJOG. 2014;121(8):1005–14. doi: 10.1111/1471-0528.12531.

114. Lloyd TD, Geneen LJ, Bernhardt K, McClune W, Fernquest SJ, Brown T, et al. Cell salvage for minimising perioperative allogeneic blood transfusion in adults undergoing elective surgery. Cochrane Database Syst Rev. 2023;(9). doi: 10.1002/14651858.CD001888.pub5.

115. Khan KS, Moore P, Wilson M, Hooper R, Allard S, Wrench I, et al. A randomised controlled trial and economic evaluation of intraoperative cell salvage during caesarean section in women at risk of haemorrhage: the SALVO (cell SALVage in Obstetrics) trial. Health Technol Assess. 2018;22(2):1–88. doi: 10.3310/hta22020.

116. Obore N, Liuxiao Z, Haomin Y, Yuchen T, Wang L, Hong Y. Intraoperative Cell Salvage for Women at High Risk of Postpartum Hemorrhage During Cesarean Section: a Systematic Review and Meta-analysis. Reprod Sci. 2022;29(11):3161–76. doi: 10.1007/s43032-021-00824-8.

117. Iyer NS, Khanuja K, Roman A, Al-Kouatly HB. Use of cell salvage at the time of cesarean delivery: a meta-analysis of randomized controlled trials. Am J Obstet Gynecol MFM. 2023;6(2):101257. doi: 10.1016/j.ajogmf.2023.101257.

118. Nadisauskiene RJ, Kliucinskas M, Dobozinskas P, Kacerauskiene J. The impact of postpartum haemorrhage management guidelines implemented in clinical practice: a systematic review of the literature. Eur J Obstet Gynecol Reprod Biol. 2014;178:21–6. doi: 10.1016/j.ejogrb.2014.03.051.

119. Bradley SE, Prata N, Young-Lin N, Bishai D. Cost-effectiveness of misoprostol to control postpartum hemorrhage in low-resource settings. Int J Gynaecol Obstet. 2007;97(1):52–6. doi: 10.1016/j.ijgo.2006.12.005.

120. Hofmeyr GJ, Ferreira S, Nikodem VC, Mangesi L, Singata M, Jafta Z, et al. Misoprostol for treating postpartum haemorrhage: a randomized controlled trial BMC Pregnancy Childbirth. 2004;4(1):16. doi: 10.1186/1471-2393-4-16.

121. Prata N, Mbaruku G, Campbell M, Potts M, Vahidnia F. Controlling postpartum hemorrhage after home births in Tanzania. Int J Gynaecol Obstet. 2005;90(1):51–5. doi: 10.1016/j.ijgo.2005.03.007.

122. Walraven G, Dampha Y, Bittaye B, Sowe M, Hofmeyr J. Misoprostol in the treatment of postpartum haemorrhage in addition to routine management: a placebo randomised controlled trial. BJOG. 2004;111(9):1014–7. doi: 10.1111/j.1471-0528.2004.00217.x.

123. Blum J. Development of evidence-based regimens on misoprostol. Current evidence: Misoprostol for PPH treatment. Seattle: Gates Foundation; 2009.

124. Howard DC, Jones AE, Skeith A, Lai J, D'Souza R, Caughey AB. Tranexamic acid for the treatment of postpartum hemorrhage: a cost-effectiveness analysis. Am J Obstet Gynecol MFM. 2022;4(3):100588. doi: 10.1016/j.ajogmf.2022.100588.

125. Sudhof LS, Shainker SA, Einerson BD. Tranexamic acid in the routine treatment of postpartum hemorrhage in the United States: a cost-effectiveness analysis. Am J Obstet Gynecol. 2019;221(3):275. e1–. e12. doi: 10.1016/j.ajog.2019.06.030.

126. Joshi BN, Shetty SS, Moray KV, Chaurasia H, Sachin O. Cost-effectiveness and budget impact of adding tranexamic acid for management of post-partum hemorrhage in the Indian public health system. BMC Pregnancy Childbirth. 2023;23(1):9. doi: 10.1186/s12884-022-05308-4.

127. Li B, Miners A, Shakur H, Roberts I. Tranexamic acid for treatment of women with post-partum haemorrhage in Nigeria and Pakistan: a cost-effectiveness analysis of data from the WOMAN trial. Lancet Glob Health. 2018;6(2):e222–e8. doi: 10.1016/S2214-109X(17)30467-9.

128. Downing J, El Ayadi A, Miller S, Butrick E, Mkumba G, Magwali T, et al. Cost-effectiveness of the non-pneumatic anti-shock garment (NASG): evidence from a cluster randomized controlled trial in Zambia and Zimbabwe. BMC Health Serv Res. 2015;15(1):1–10. doi: 10.1186/s12913-015-0694-6.

129. El Ayadi A, Gibbons L, Bergel E, Butrick E, Huong NT, Mkumba G, et al. Per-protocol effect of earlier non-pneumatic anti-shock garment application for obstetric hemorrhage. Int J Gynaecol Obstet. 2014;126(1):95–6. doi: 10.1016/j.ijgo.2014.02.005.

130. Sutherland T, Downing J, Miller S, Bishai DM, Butrick E, Fathalla MM, et al. Use of the non-pneumatic anti-shock garment (NASG) for life-threatening obstetric hemorrhage: a cost-effectiveness analysis in Egypt and Nigeria. PloS One. 2013;8(4):e62282. doi: 10.1371/journal.pone.0062282.

131. Miller S, Fathalla MM, Ojengbede OA, Camlin C, Mourad-Youssif M, Morhason-Bello IO, et al. Obstetric hemorrhage and shock management: using the low technology Non-pneumatic Anti-Shock Garment in Nigerian and Egyptian tertiary care facilities. BMC Pregnancy Childbirth. 2010;10:64. doi: 10.1186/1471-2393-10-64.

132. Mvundura M, Kokonya D, Abu‐Haydar E, Okoth E, Herrick T, Mukabi J, et al. Cost‐effectiveness of condom uterine balloon tamponade to control severe postpartum hemorrhage in Kenya. Int J Gynaecol Obstet. 2017;137(2):185–91. doi: 10.1002/ijgo.12125.

133. Burke TF, Ahn R, Nelson BD, Hines R, Kamara J, Oguttu M, et al. A postpartum haemorrhage package with condom uterine balloon tamponade: a prospective multi-centre case series in Kenya, Sierra Leone, Senegal, and Nepal. BJOG. 2016;123(9):1532–40. doi: 10.1111/1471-0528.13550.

134. Joshi BN, Shetty SS, Moray KV, Sachin O, Chaurasia H. Cost-effectiveness of uterine balloon tamponade devices in managing atonic post-partum hemorrhage at public health facilities in India. PLoS One. 2021;16(8):e0256271. doi: 10.1371/journal.pone.0256271.

135. Edwards RT, Ezeofor V, Bryning L, Anthony BF, Charles JM, Weeks A. Prevention of postpartum haemorrhage: Economic evaluation of the novel butterfly device in a UK setting. Eur J Obstet Gynecol Reprod Biol. 2023;283:149–57. doi: 10.1016/j.ejogrb.2023.02.020.

136. Weeks AD, Cunningham C, Taylor W, Rosala-Hallas A, Watt P, Bryning L, et al. A mixed method, phase 2 clinical evaluation of a novel device to treat postpartum haemorrhage. Eur J Obstet Gynecol Reprod Biol. 2023;283:142–8. doi: 10.1016/j.ejogrb.2023.01.018.

137. Einerson BD, Stehlikova Z, Nelson RE, Bellows BK, Kawamoto K, Clark EA. Transfusion preparedness strategies for obstetric hemorrhage: a cost-effectiveness analysis. Obstet Gynecol. 2017;130(6):1347–55. doi: 10.1097/AOG.0000000000002359.

138. Lim G, Melnyk V, Facco FL, Waters JH, Smith KJ. Cost-effectiveness analysis of intraoperative cell salvage for obstetric hemorrhage. Anesthesiology. 2018;128(2):328–37. doi: 10.1097/ALN.0000000000001981.

139. Carless PA, Henry DA, Moxey AJ, O'Connell D, Brown T, Fergusson DA. Cell salvage for minimising perioperative allogeneic blood transfusion. Cochrane Database Syst Rev. 2010;2010(4):Cd001888. doi: 10.1002/14651858.CD001888.pub4.

140. Ries J-J, Jeker L, Neuhaus M, Vogt DR, Girard T, Hoesli I. Implementation of the D-A-CH postpartum haemorrhage algorithm after severe postpartum bleeding accelerates clinical management: A retrospective case series. Eur J Obstet Gynecol Reprod Biol. 2020;247:225–31. doi: 10.1016/j.ejogrb.2020.01.001.

141. Franke MA, Nordmann K, Frühauf A, Ranaivoson RM, Rebaliha M, Rapanjato Z, et al. Inter-facility transfers for emergency obstetrical and neonatal care in rural Madagascar: a cost-effectiveness analysis. BMJ Open. 2024;14(4):e081482. doi: 10.1136/bmjopen-2023-081482.

142. Besaina R, Romuald R, Laingo R, Tanjona R, A. RJ. Maternal mortality related to postpartum hemorrhage: a case-control study at the Befelatanana maternity of Madagascar. Int J Reprod Contracept Obstet Gynecol. 2019;8(1):121-6. doi: <https://doi.org/10.18203/2320-1770.ijrcog20185406>.

143. Vogel JP, Nguyen P-Y, Ramson J, De Silva MS, Pham MD, Sultana S, et al. Effectiveness of care bundles for prevention and treatment of postpartum hemorrhage: a systematic review. Am J Obstet Gynecol. 2024. doi: 10.1016/j.ajog.2024.01.012.

144. Gallos I, Devall A, Martin J, Middleton L, Beeson L, Galadanci H, et al. Randomized Trial of Early Detection and Treatment of Postpartum Hemorrhage. N Engl J Med. 2023;389(1):11–21. doi: 10.1056/NEJMoa2303966.

145. Barinov SV, Zhukovsky YG, Dolgikh VT, Medyannikova IV. Novel combined strategy of obstetric haemorrhage management during caesarean section using intrauterine balloon tamponade. J Matern Fetal Neonatal Med. 2017;30(1):29–33. doi: 10.3109/14767058.2015.1126242.

146. Main EK, Cape V, Abreo A, Vasher J, Woods A, Carpenter A, et al. Reduction of severe maternal morbidity from hemorrhage using a state perinatal quality collaborative. Am J Obstet Gynecol. 2017;216(3):298.e1–.e11. doi: 10.1016/j.ajog.2017.01.017.

147. Seim AR, Alassoum Z, Souley I, Bronzan R, Mounkaila A, Ahmed LA. The effects of a peripartum strategy to prevent and treat primary postpartum haemorrhage at health facilities in Niger: a longitudinal, 72-month study. Lancet Glob Health. 2023;11(2):e287–e95. doi: 10.1016/S2214-109X(22)00518-6.

148. Wiesehan EC, Keesara SR, Krissberg JR, Main EK, Goldhaber-Fiebert JD. State perinatal quality collaborative for reducing severe maternal morbidity from hemorrhage: a cost-effectiveness analysis. Obstet Gynecol. 2023;141(2):387–94. doi: 10.1097/AOG.0000000000005060.

149. Dale M, Bell SF, O’Connell S, Scarr C, James K, John M, et al. What is the economic cost of providing an all Wales postpartum haemorrhage quality improvement initiative (OBS Cymru)? A cost-consequences comparison with standard care. Pharmacoecon Open. 2022;6(6):847–57. doi: 10.1007/s41669-022-00362-2

150. Bell SF, Collis RE, Pallmann P, Bailey C, James K, John M, et al. Reduction in massive postpartum haemorrhage and red blood cell transfusion during a national quality improvement project, Obstetric Bleeding Strategy for Wales, OBS Cymru: an observational study. BMC Pregnancy Childbirth. 2021;21(1):377. doi: 10.1186/s12884-021-03853-y.

151. Williams EV, Goranitis I, Oppong R, Perry SJ, Devall AJ, Martin JT, et al. A cost-effectiveness analysis of early detection and bundled treatment of postpartum hemorrhage alongside the E-MOTIVE trial. Nat Med. 2024. doi: 10.1038/s41591-024-03069-5.
