# Supplementary material for: The cost-effectiveness of preventing, diagnosing, and treating postpartum haemorrhage: A systematic review of economic evaluations
Source: PLoS Med. 2024 Sep 13;21(9):e1004461. doi: 10.1371/journal.pmed.1004461 (PMC11433145; doi:10.1371/journal.pmed.1004461)
Supplement: S9 Appendix — (DOCX) [file pmed.1004461.s009.docx]

# **S9 Appendix: Economic evaluations mapped to WHO recommendations for the prevention, diagnosis and treatment of PPH**

Table A: Economic evaluations mapped to WHO recommendations on the use of uterotonics for the prevention of PPH

| **Recommendations** | | | |
| --- | --- | --- | --- |
| ***Administration of an effective uterotonic after birth in women following vaginal birth or caesarean section [1].***  ***Oxytocin (10 IU, IM/IV) is the agent of choice when multiple uterotonic options are available [2].***  ***In settings where oxytocin is unavailable (or its quality cannot be guaranteed), the use of other injectable uterotonics (carbetocin, or if appropriate ergometrine/methylergometrine, or oxytocin and ergometrine fixed-dose combination) or oral misoprostol is recommended [1].*** | | | |
| **World Bank Income Group** | **Country** | **Study** | **Aspects of study applicable to these recommendations** |
| Low Income | Uganda | Lubinga et al., 2015 [3] | Examines the cost-effectiveness of prenatal community distribution of misoprostol for prevention of PPH. |
| Lower-middle Income | India | Cook et al., 2023 [4] | Evaluates the cost-effectiveness of prophylactic carbetocin, oxytocin and misoprostol. |
|  | India | Sutherland et al., 2009 [5] | Includes assessment of cost-effectiveness of prophylactic misoprostol use in home births. |
|  | India | Sutherland et al., 2010 [6] | Includes assessment of cost-effectiveness of prophylactic misoprostol use in home births. |
|  | India | Goldie et al., 2010 [7] | Includes assessment of cost-effectiveness of prophylactic misoprostol use in homes and birth centers. |
|  | Philippines | Briones et al., 2020 [8] | Evaluates the cost-utility and budget impact of prophylactic carbetocin compared with oxytocin. |
|  | Senegal | Vlassoff et al., 2015 [9] | Compares the use of prophylactic oxytocin and misoprostol in a community-based setting. |
|  | Vietnam | Tsu et al., 2009 [10] | Estimates the costs and cost-effectiveness of introducing routine AMTSL with oxytocin. |
| Upper middle Income | China | You et al., 2022 [11] | Examines the cost-effectiveness of carbetocin versus oxytocin for the prevention of PPH. |
|  | Ecuador | Henriquez-Trujillo et al., 2017 [12] | Compares the cost-effectiveness of prophylactic carbetocin and oxytocin. |
|  | Peru | Diaz et al., 2009 [13] | Assesses the addition of oxytocin and AMTSL as part of the PARSalud program in Peru. |
|  | Peru | Caceda et al., 2018 [14] | Assesses the cost-effectiveness of carbetocin versus oxytocin for the prevention of PPH. |
|  | Colombia | Gil-Rojas et al., 2018 [15] | Assesses the cost-effectiveness of carbetocin versus oxytocin for prevention of PPH. |
|  | Malaysia | Voon et al., 2018 [16] | Assesses the cost-effectiveness of carbetocin versus oxytocin for prevention of PPH. |
| High Income | Australia | Wohling et al., 2019 [17] | Assesses the cost-effectiveness of carbetocin versus oxytocin for prevention of PPH. |
|  | United Kingdom | van der Nelson et al., 2017 [18] | Assesses the cost-effectiveness of carbetocin versus oxytocin for prevention of PPH. |
|  | United Kingdom | Pickering et al., 2019 [19] | Compares the relative cost -effectiveness of the full range of uterotonic drugs available (including oxytocin) for the prevention of PPH in VB. |
|  | United Kingdom | Gallos et al., 2019 [20] | Ranks the most effective and cost-effective uterotonic drug(s) to prevent PPH (including oxytocin). |
|  | United Kingdom | Luni et al., 2017 [21] | Evaluates of the use of carbetocin against oxytocin for prevention of PPH. |
|  | United Kingdom | Higgins et al., 2011 [22] | Evaluates of the use of carbetocin against oxytocin for prevention of PPH. |
|  | United Kingdom | Matthijsse et al., 2022 [23] | Evaluates of the use of carbetocin against oxytocin for prevention of PPH. |
|  | Canada | Barrett et al., 2022 [24] | Evaluates of the use of carbetocin against oxytocin for prevention of PPH. |
| Multiple Income Levels | All Latin America and the Caribbean countries. | Pichon-Riviere et al., 2015 [25] | Evaluates the cost-effectiveness of oxytocin administered via ampoules vs. Uniject administration in all Latin America and the Caribbean countries. |
|  | 34 countries in Sub-Saharan Africa | Prata et al., 2010 [26] | Includes assessment of the addition of misoprostol to the standard WHO mother-baby package of interventions. |
|  | “International” | Lang et al., 2015 [27] | Evaluate the costs and health outcomes of oral misoprostol to prevent PPH in settings where injectable uterotonics are not available. |

Abbreviations: AMTSL: Active Management of the Third Stage of Labor. IM: Intramuscular. IU: International Units. IV: Intravenous. PPH: Postpartum haemorrhage. VB: Vaginal birth

Table B: Economic evaluations mapped to WHO recommendations on the use of misoprostol for the prevention of PPH by community health workers

| **Recommendation** | | | |
| --- | --- | --- | --- |
| ***In settings where skilled health personnel are not present to administer injectable uterotonics, the administration of misoprostol (400 μg or 600 μg, PO) by community health workers and lay health workers is recommended for the prevention of PPH [1].*** | | | |
| **World Bank Income Group** | **Country** | **Study** | **Aspects of study relevant applicable to this recommendation** |
| Low Income | Uganda | Lubinga et al., 2015 [3] | Examines the cost-effectiveness of prenatal community distribution of misoprostol for prevention of PPH. |
| Lower middle-income | India | Sutherland et al., 2009 [5] | Includes assessment of cost-effectiveness of prophylactic misoprostol use in home births. |
|  | India | Sutherland et al., 2010 [6] | Includes assessment of cost-effectiveness of prophylactic misoprostol use in home births. |
|  | India | Goldie et al., 2010 [7] | Includes assessment of cost-effectiveness of prophylactic misoprostol use in homes and birth centres. |

Abbreviations: PPH: Postpartum haemorrhage. PO: per oral. μg: micrograms.

Table C: Economic evaluations mapped to WHO recommendations on the use of controlled cord traction for the prevention of PPH following vaginal birth

| **Recommendation** | | | |
| --- | --- | --- | --- |
| ***Controlled cord traction by skilled health professionals for vaginal births [28].*** | | | |
| **World Bank Income Group** | **Country** | **Study** | **Aspects of study relevant applicable to this recommendation** |
| Lower middle-income | Vietnam | Tsu et al., 2009 [10] | Estimates the costs and cost-effectiveness of introducing routine AMTSL with oxytocin. (Note: CCT only considered as a component of AMTSL, not as standalone intervention) |
| Upper middle Income | Peru | Diaz et al., 2009 [13] | Assesses the addition of oxytocin and AMTSL as part of the PARSalud program in Peru. (Note: CCT only considered as a component of AMTSL, not as standalone intervention) |
| Multiple Income Levels | Guatemala and Zambia | Fullerton et al., 2006 [29] | Evaluates the use of AMTSL (uterotonic not specified) rather than EMTSL for mothers in Guatemala and Zambia. (Note: CCT only considered as a component of AMTSL, not as standalone intervention) |

Abbreviations: AMTSL: Active management of the third stage of labour. CCT: controlled cord traction.

Table D: Economic evaluations mapped to WHO recommendations on the use of controlled cord traction for the prevention of PPH following caesarean delivery

| **Recommendation** | | | |
| --- | --- | --- | --- |
| ***Controlled cord traction for removal of the placenta in caesarean section [28].*** | | | |
| **World Bank Income Group** | **Country** | **Study** | **Aspects of study relevant applicable to this recommendation** |
| No studies identified | | | |

Table E: Economic evaluations mapped to WHO recommendations on the use of late cord clamping

| **Recommendation** | | | |
| --- | --- | --- | --- |
| ***Late cord clamping (performed after 1 to 3 minutes after birth) for all births (early cord clamping is not recommended, unless the neonate is asphyxiated and needs to move immediately for resuscitation) [28].*** | | | |
| **World Bank Income Group** | **Country** | **Study** | **Aspects of study relevant applicable to this recommendation** |
| No studies identified | | | |

Table F: Economic evaluations mapped to WHO recommendations on the use of antenatal misoprostol distribution for the prevention of PPH

| **Recommendation** | | | |
| --- | --- | --- | --- |
| ***In settings where women give birth outside of a health facility and in the absence of skilled health personnel, a strategy of antenatal distribution of misoprostol to pregnant women for self-administration is recommended for prevention of postpartum haemorrhage, only with targeted monitoring and evaluation [30].*** | | | |
| **World Bank Income Group** | **Country** | **Study** | **Aspects of study relevant applicable to this recommendation** |
| Low Income | Uganda | Lubinga et al., 2015 [3] | Examines the cost-effectiveness of prenatal community distribution of misoprostol for prevention of PPH. |
| Multiple Income Levels | 34 countries in Sub-Saharan Africa | Prata et al., 2010 [26] | Includes assessment of the addition of misoprostol to the standard WHO mother-baby package of interventions. |

Abbreviations: PPH: Postpartum haemorrhage. WHO: World Health Organization.

**Diagnosis Recommendations**

Table G: Economic evaluations mapped to WHO recommendations on the use of postpartum abdominal uterine tonus assessments

| **Recommendation** | | | |
| --- | --- | --- | --- |
| ***Postpartum abdominal uterine tonus assessment for early identification of uterine atony for all women*** *[28].* | | | |
| **World Bank Income Group** | **Country** | **Study** | **Aspects of study relevant applicable to this recommendation** |
| No studies identified | | | |

Table H: Economic evaluations mapped to WHO recommendations on the use of routine measurement of postpartum blood loss

| **Recommendation** | | | |
| --- | --- | --- | --- |
| ***For all women giving birth, routine objective measurement of postpartum blood loss is recommended to improve the detection and prompt treatment of postpartum haemorrhage. Methods to objectively quantify blood loss, such as calibrated drapes for women having vaginal birth, can achieve this*** *[31].* | | | |
| **World Bank Income Group** | **Country** | **Study** | **Aspects of study relevant applicable to this recommendation** |
| High Income | United States of America | Katz et al., 2020 [32] | Assesses the addition of quantitative measurement of blood loss using a gravimetric and colorimetric method to routine PPH care. |
|  | United States of America | Wiesehan et al 2023 [33] | Assesses the cost-effectiveness of the statewide perinatal quality collaborative initiative to reduce severe maternal morbidity from PPH. The bundle includes PPH blood loss measurement. |
|  | Wales | Dale et al., 2022 [34] | Compares the incremental cost and outcomes of a quality improvement initiative (bundle) to standard PPH care in Wales.  The bundle includes PPH blood loss measurement. |
| Multiple Income Levels | Kenya, Nigeria, South Africa, Tanzania | Williams et al., 2024 [35] | Assesses cost-effectiveness of early diagnosis of PPH with calibrated drapes and management of PPH using the WHO first-response treatment bundle. |

Abbreviations: PPH: Postpartum haemorrhage. WHO: World Health Organization.

**Treatment (1^st^ Response) Recommendations**

Table I: Economic evaluations mapped to WHO recommendations on the use PPH treatment bundles

| **Recommendation** | | | |
| --- | --- | --- | --- |
| ***A standardized and timely approach to the management of postpartum haemorrhage (PPH), comprising an objective assessment of blood loss and use of a treatment bundle supported by an implementation strategy, is recommended for all women having a vaginal birth. The care bundle for first-line treatment of PPH should include rapid institution of uterine massage, administration of an oxytocic agent and tranexamic acid, intravenous fluids, examination of the genital tract and escalation of care [31].*** | | | |
| **World Bank Income Group** | **Country** | **Study** | **Aspects of study relevant applicable to this recommendation** |
| Low Income | Niger | Seim et al., 2023 [36] | Describes the outcomes of the national implementation of a strategy (bundle) to reduce death from bleeding at childbirth. |
| High Income | United States of America | Wiesehan et al 2023 [33] | Assesses the cost-effectiveness of the statewide perinatal quality collaborative initiative (including a bundle) to reduce severe maternal morbidity from PPH. |
|  | Wales | Dale et al., 2022 [34] | Compares the incremental cost and outcomes of a quality improvement initiative (including a bundle) to standard PPH care in Wales.  The bundle includes PPH blood loss measurement. |
| Multiple Income Levels | Kenya, Nigeria, South Africa, Tanzania | Williams et al., 2024 [35] | Assesses cost-effectiveness of early diagnosis of PPH with calibrated drapes and management of PPH using the WHO first-response treatment bundle. |

Abbreviations: PPH: Postpartum haemorrhage. WHO: World Health Organization.

Table J: Economic evaluations mapped to WHO recommendations on the use oxytocin for PPH treatment

| **Recommendation** | | | |
| --- | --- | --- | --- |
| ***IV oxytocin as first-line treatment for postpartum haemorrhage [28].*** | | | |
| **World Bank Income Group** | **Country** | **Study** | **Aspects of study relevant applicable to this recommendation** |
| No studies identified | | | |

Table K: Economic evaluations mapped to WHO recommendations on the use of other uterotonics for PPH treatment

| **Recommendation** | | | |
| --- | --- | --- | --- |
| ***If IV oxytocin is unavailable, or if the bleeding does not respond to oxytocin, the use of intravenous ergometrine, oxytocin-ergometrine fixed dose, or a prostaglandin drug (including sublingual misoprostol, 800 μg) is recommended [28].*** | | | |
| **World Bank Income Group** | **Country** | **Study** | **Aspects of study relevant applicable to this recommendation** |
| Lower middle-income | India | Sutherland et al., 2010 [6] | Includes assessment of cost-effectiveness of misoprostol as treatment for PPH occurring in home births. |
| Multiple Income Levels | Sub-Saharan Africa (no country specified) | Bradley et al., 2007 [37] | Assesses the cost-effectiveness of training TBAs to recognize PPH and administer a rectal dose of misoprostol in areas with low access to modern delivery facilities. |

Abbreviations: IV: Intravenous. PPH: Postpartum haemorrhage. TBAs: Traditional birth attendant. μg: micrograms.

Table L: Economic evaluations mapped to WHO recommendations on the use tranexamic acid for the treatment of PPH

| **Recommendation** | | | |
| --- | --- | --- | --- |
| ***Early IV tranexamic acid (within 3 hours of birth) in addition to standard care for women with PPH for all births [38].*** | | | |
| **World Bank Income Group** | **Country** | **Study** | **Aspects of study relevant applicable to this recommendation** |
| Lower middle-income | India | Joshi et al., 2023 [39] | Assesses the cost-effectiveness of administering IV tranexamic acid to women experiencing PPH within 3h of birth in addition to existing management strategies. |
|  | Nigeria & Pakistan | Li et al., 2018 [40] | Evaluates the cost-effectiveness of tranexamic acid for treatment of PPH |
| High Income | United States of America | Howard et al., 2022 [41] | Assesses the cost-effectiveness of administering IV tranexamic acid to women experiencing PPH |
|  | United States of America | Sudhof et al., 2019 [42] | Assesses the cost-effectiveness of administering tranexamic acid TXA to women experiencing PPH |

Abbreviations: IV: Intravenous. PPH: Postpartum haemorrhage.

Table M: Economic evaluations mapped to WHO recommendations on the use uterotonics and controlled cord traction for retained placenta

| **Recommendation** | | | |
| --- | --- | --- | --- |
| ***IV/IM oxytocin (10 IU) in combination with controlled cord traction if the placenta is not expelled spontaneously [28].*** | | | |
| **World Bank Income Group** | **Country** | **Study** | **Aspects of study relevant applicable to this recommendation** |
| No studies identified | | | |

Abbreviations: IM: Intramuscular. IU: International Units. IV: Intravenous.

Table N: Economic evaluations mapped to WHO recommendations on the use antibiotics if manual removal of placenta is practiced

| **Recommendation** | | | |
| --- | --- | --- | --- |
| ***Single dose of antibiotics (ampicillin or first-generation cephalosporin) if manual removal of the placenta is practiced [28].*** | | | |
| **World Bank Income Group** | **Country** | **Study** | **Aspects of study relevant applicable to this recommendation** |
| No studies identified | | | |

Table O: Economic evaluations mapped to WHO recommendations on the use intravenous fluids for treating PPH

| **Recommendation** | | | |
| --- | --- | --- | --- |
| ***Isotonic crystalloids in preference to colloids for the initial intravenous fluid resuscitation of women with postpartum haemorrhage [28].*** | | | |
| **World Bank Income Group** | **Country** | **Study** | **Aspects of study relevant applicable to this recommendation** |
| No studies identified | | | |

**Treatment (refractory) Recommendations**

Table P: Economic evaluations mapped to WHO recommendations on the use uterine massage, uterine compression, or aortic compression for the treatment of PPH

| **Recommendation** | | | |
| --- | --- | --- | --- |
| ***Uterine massage for the treatment of postpartum haemorrhage*** *[28].*  ***Bimanual uterine compression as a temporizing measure until appropriate care is available for postpartum haemorrhage due to uterine atony after vaginal birth [28].***  ***External aortic compression as a temporizing measure until appropriate care is available for postpartum haemorrhage due to uterine atony after vaginal birth [28].*** | | | |
| **World Bank Income Group** | **Country** | **Study** | **Aspects of study relevant applicable to these recommendations** |
| No studies identified | | | |

Table Q: Economic evaluations mapped to WHO recommendations on the use of uterine balloon tamponade for PPH treatment

| **Recommendation** | | | |
| --- | --- | --- | --- |
| ***Uterine balloon tamponade for treatment of postpartum haemorrhage due to uterine atony after vaginal birth in women who do not respond to standard first-line treatment provided certain conditions are met [43].*** | | | |
| **World Bank Income Group** | **Country** | **Study** | **Aspects of study relevant applicable to this recommendation** |
| Lower middle-income | Kenya | Mvundura et al., 2017 [44] | Evaluates the cost-effectiveness of condom-UBT versus standard care for control of severe PPH due to uterine atony. |
|  | India | Joshi et al., 2021 [45] | Compares the cost-effectiveness of multiple UBT devices for the management of atonic PPH. |

Abbreviations: PPH: postpartum haemorrhage. UBT: uterine balloon tamponade.

Table R: Economic evaluations mapped to WHO recommendations on the use of non-pneumatic anti-shock garment for PPH treatment

| **Recommendation** | | | |
| --- | --- | --- | --- |
| ***Non-pneumatic anti-shock garment as a temporizing measure until appropriate care is available [28].*** | | | |
| **World Bank Income Group** | **Country** | **Study** | **Aspects of study relevant applicable to this recommendation** |
| Lower middle-income | Zambia & Zimbabwe | Downing et al., 2015 [46] | Evaluate the cost-effectiveness of early NASG application at the primary health care level prior to transport compared to later NASG application at the referral hospital. |
|  | Egypt & Nigeria | Sutherland et al., 2013 [47] | Assesses the cost-effectiveness of NASG for obstetric haemorrhage in tertiary hospitals. |

Abbreviations: NASG: Non-pneumatic anti-shock garment.

Table S: Economic evaluations mapped to WHO recommendations on the use of uterine artery embolization or surgical intervention for PPH treatment

| **Recommendation** | | | |
| --- | --- | --- | --- |
| ***Uterine artery embolization for treatment for PPH due to uterine atony if other measures have failed and resources are available [28].***  ***Surgical interventions if bleeding does not stop despite treatment with uterotonic treatment and other available conservative interventions (e.g., uterine massage, balloon tamponade) [28].*** | | | |
| **World Bank Income Group** | **Country** | **Study** | **Aspects of study relevant applicable to these recommendations** |
| No studies identified | | | |

Abbreviations: PPH: Postpartum haemorrhage.

Table T: Economic evaluations mapped to WHO recommendations on the use of umbilical vein injection for the treatment of retained placenta

| **Recommendation** | | | |
| --- | --- | --- | --- |
| ***Umbilical vein injection of oxytocin is recommended for the treatment of retained placenta only in the context of rigorous research [48].*** | | | |
| **World Bank Income Group** | **Country** | **Study** | **Aspects of study relevant applicable to this recommendation** |
| No studies identified | | | |

**Health System Recommendations**

Table U: Economic evaluations mapped to WHO recommendations on the use of formal protocols for PPH prevention and treatment

| **Recommendation** | | | |
| --- | --- | --- | --- |
| ***Formal protocols by health facilities for the prevention and treatment of PPH [28].*** | | | |
| **World Bank Income Group** | **Country** | **Study** | **Aspects of study relevant applicable to this recommendation** |
| High Income | Switzerland | Ries et al., 2020 [49] | Evaluates the outcomes of PPH clinical management and cost before and after the introduction of a treatment algorithm. |

Abbreviations: PPH: Postpartum haemorrhage.

Table V: Economic evaluations mapped to WHO recommendations on the use of formal protocols for referring women to higher levels of care

| **Recommendation** | | | |
| --- | --- | --- | --- |
| ***Formal protocols for referral of women to a higher level of care for health facilities [28].*** | | | |
| **World Bank Income Group** | **Country** | **Study** | **Aspects of study relevant applicable to this recommendation** |
| No studies identified | | | |

Table W: Economic evaluations mapped to WHO recommendations on the use of simulation training for PPH management

| **Recommendation** | | | |
| --- | --- | --- | --- |
| ***Simulations of PPH treatment for pre-service and in-service training programmes [28].*** | | | |
| **World Bank Income Group** | **Country** | **Study** | **Aspects of study relevant applicable to this recommendation** |
| No studies identified | | | |

Table X: Economic evaluations mapped to WHO recommendations on the use of uterotonic provision as a process indicator for evaluation

| **Recommendation** | | | |
| --- | --- | --- | --- |
| ***Monitoring use of uterotonics after birth for the prevention of PPH as a process indicator for programmatic evaluation [28].*** | | | |
| **World Bank Income Group** | **Country** | **Study** | **Aspects of study relevant applicable to this recommendation** |
| No studies identified | | | |

Abbreviations: PPH: Postpartum haemorrhage.

# **References**

1. World Health Organization. WHO recommendations: uterotonics for the prevention of postpartum haemorrhage. Geneva: World Health Organization, 2018.

2. World Health Organization. WHO recommendation on routes of oxytocin administration for the prevention of postpartum haemorrhage after vaginal birth. Geneva: World Health Organization, 2020.

3. Lubinga SJ, Atukunda EC, Wasswa-Ssalongo G, Babigumira JB. Potential cost-effectiveness of prenatal distribution of misoprostol for prevention of postpartum hemorrhage in Uganda. PLoS One. 2015;10(11):e0142550. doi: 10.1371/journal.pone.0142550.

4. Cook JR, Saxena K, Taylor C, Jacobs JL. Cost-effectiveness and budget impact of heat-stable carbetocin compared to oxytocin and misoprostol for the prevention of postpartum hemorrhage (PPH) in women giving birth in India. BMC Health Serv Res. 2023;23(1):267. doi: 10.1186/s12913-023-09263-4.

5. Sutherland T, Bishai DM. Cost-effectiveness of misoprostol and prenatal iron supplementation as maternal mortality interventions in home births in rural India. Int J Gynaecol Obstet. 2009;104(3):189–93. doi: 10.1016/j.ijgo.2008.10.011.

6. Sutherland T, Meyer C, Bishai DM, Geller S, Miller S. Community-based distribution of misoprostol for treatment or prevention of postpartum hemorrhage: cost-effectiveness, mortality, and morbidity reduction analysis. Int J Gynaecol Obstet. 2010;108(3):289–94. doi: 10.1016/j.ijgo.2009.11.007.

7. Goldie SJ, Sweet S, Carvalho N, Natchu UCM, Hu D. Alternative strategies to reduce maternal mortality in India: a cost-effectiveness analysis. PLoS Med. 2010;7(4):e1000264. doi: 10.1371/journal.pmed.1000264.

8. Briones JR, Talungchit P, Thavorncharoensap M, Chaikledkaew U. Economic evaluation of carbetocin as prophylaxis for postpartum hemorrhage in the Philippines. BMC Health Serv Res. 2020;20:1–12. doi: 10.1186/s12913-020-05834-x.

9. Vlassoff M, Diallo A, Philbin J, Kost K, Bankole A. Cost-effectiveness of two interventions for the prevention of postpartum hemorrhage in Senegal. Int J Gynaecol Obstet. 2016;133(3):307–11. doi: 10.1016/j.ijgo.2015.10.015.

10. Tsu VD, Levin C, Tran MP, Hoang MV, Luu HT. Cost-effectiveness analysis of active management of third-stage labour in Vietnam. Health Policy Plan. 2009;24(6):438–44. doi: 10.1093/heapol/czp020.

11. You JH, Leung T-y. Cost-effectiveness analysis of carbetocin for prevention of postpartum hemorrhage in a low-burden high-resource city of China. PLoS One. 2022;17(12):e0279130. doi: 10.1371/journal.pone.0279130.

12. Henríquez-Trujillo AR, Lucio-Romero RA, Bermúdez-Gallegos K. Analysis of the cost–effectiveness of carbetocin for the prevention of hemorrhage following cesarean delivery in Ecuador. J Comp Eff Res. 2017;6(6):529–36. doi: 10.2217/cer-2017-0004.

13. Jose Diaz J, Jaramillo M. Evaluating interventions to reduce maternal mortality: evidence from Peru's PARSalud programme. J Dev Effect. 2009;1(4):387–412. doi: 10.1080/19439340903380872.

14. Caceda SI, Ramos RR, Saborido CM. Pharmacoeconomic study comparing carbetocin with oxytocin for the prevention of hemorrhage following cesarean delivery in Lima, Peru. J Comp Eff Res. 2018;7(1):49-55. doi: 10.2217/cer-2017-0012.

15. Gil-Rojas Y, Lasalvia P, Hernández F, Castañeda-Cardona C, Rosselli D. Cost-effectiveness of Carbetocin versus Oxytocin for Prevention of Postpartum Hemorrhage Resulting from Uterine Atony in Women at high-risk for bleeding in Colombia. Rev Bras Ginecol Obstet. 2018;40:242–50. doi: 10.1055/s-0038-1655747.

16. Voon HY, Shafie AA, Bujang MA, Suharjono HN. Cost effectiveness analysis of carbetocin during cesarean section in a high volume maternity unit. J Obstet Gynaecol Res. 2018;44(1):109–16. doi: 10.1111/jog.13486.

17. Wohling J, Edge N, Pena‐Leal D, Wang R, Mol BW, Dekker G. Clinical and financial evaluation of carbetocin as postpartum haemorrhage prophylaxis at caesarean section: A retrospective cohort study. Aust N Z J Obstet Gynaecol. 2019;59(4):501–7. doi: 10.1111/ajo.12907.

18. Van Der Nelson HA, Draycott T, Siassakos D, Yau CW, Hatswell AJ. Carbetocin versus oxytocin for prevention of post-partum haemorrhage at caesarean section in the United Kingdom: an economic impact analysis. Eur J Obstet Gynecol Reprod Biol. 2017;210:286-91. doi: 10.1016/j.ejogrb.2017.01.004.

19. Pickering K, Gallos ID, Williams H, Price MJ, Merriel A, Lissauer D, et al. Uterotonic drugs for the prevention of postpartum haemorrhage: a cost-effectiveness analysis. Pharmacoecon Open. 2019;3:163–76. doi: 10.1007/s41669-018-0108-x.

20. Gallos I, Williams H, Price M, Pickering K, Merriel A, Tobias A, et al. Uterotonic drugs to prevent postpartum haemorrhage: a network meta-analysis. Health Technol Assess. 2019;23(9). doi: 10.3310/hta23090.

21. Luni Y, Borakati A, Matah A, Skeats K, Eedarapalli P. A prospective cohort study evaluating the cost-effectiveness of carbetocin for prevention of postpartum haemorrhage in caesarean sections. J Obstet Gynaecol Can. 2017;37(5):601–4. doi: 10.1080/01443615.2017.1284188.

22. Higgins L, Mechery J, Tomlinson A. Does carbetocin for prevention of postpartum haemorrhage at caesarean section provide clinical or financial benefit compared with oxytocin? J Obstet Gynaecol. 2011;31(8):732–9. doi: 10.3109/01443615.2011.595982.

23. Matthijsse S, Andersson FL, Gargano M, Yip Sonderegger YL. Cost-effectiveness analysis of carbetocin versus oxytocin for the prevention of postpartum hemorrhage following vaginal birth in the United Kingdom. J Med Econ. 2022;25(1):129–37. doi: 10.1080/13696998.2022.2027669.

24. Barrett J, Ko S, Jeffery W. Cost implications of using carbetocin injection to prevent postpartum hemorrhage in a Canadian urban Hospital. J Obstet Gynaecol Can. 2022;44(3):272–8. doi: 10.1016/j.jogc.2021.09.022.

25. Pichon-Riviere A, Glujovsky D, Garay OU, Augustovski F, Ciapponi A, Serpa M, et al. Oxytocin in uniject disposable auto-disable injection system versus standard use for the prevention of postpartum hemorrhage in latin America and the Caribbean: a cost-effectiveness analysis. PLoS One. 2015;10(6):e0129044. doi: 10.1371/journal.pone.0129044.

26. Prata N, Sreenivas A, Greig F, Walsh J, Potts M. Setting priorities for safe motherhood interventions in resource-scarce settings. Health Policy. 2010;94(1):1–13. doi: 10.1016/j.healthpol.2009.08.012.

27. Lang DL, Zhao F-L, Robertson J. Prevention of postpartum haemorrhage: cost consequences analysis of misoprostol in low-resource settings. BMC Pregnancy Childbirth. 2015;15(1):1–9. doi: 10.1186/s12884-015-0749-z.

28. World Health Organization. WHO recommendations for the prevention and treatment of postpartum haemorrhage. Geneva: World Health Organization, 2012.

29. Fullerton JT, Frick KD, Fogarty LA, Fishel JD, Vivio DM. Active management of third stage of labour saves facility costs in Guatemala and Zambia. J Health Popul Nutr. 2006;24(4):540.

30. World Health Organization. WHO recommendation on Advance misoprostol distribution to pregnant women for prevention of postpartum haemorrhage. Geneva: World Health Organization, 2020.

31. World Health Organization. WHO recommendations on the assessment of postpartum blood loss and use of a treatment bundle for postpartum haemorrhage. Geneva: World Health Organization, 2023.

32. Katz D, Wang R, O'Neil L, Gerber C, Lankford A, Rogers T, et al. The association between the introduction of quantitative assessment of postpartum blood loss and institutional changes in clinical practice: an observational study. Int J Obstet Anesth. 2020;42:4–10. doi: 10.1016/j.ijoa.2019.05.006.

33. Wiesehan EC, Keesara SR, Krissberg JR, Main EK, Goldhaber-Fiebert JD. State perinatal quality collaborative for reducing severe maternal morbidity from hemorrhage: a cost-effectiveness analysis. Obstet Gynecol. 2023;141(2):387–94. doi: 10.1097/AOG.0000000000005060.

34. Dale M, Bell SF, O’Connell S, Scarr C, James K, John M, et al. What is the economic cost of providing an all Wales postpartum haemorrhage quality improvement initiative (OBS Cymru)? A cost-consequences comparison with standard care. Pharmacoecon Open. 2022;6(6):847–57. doi: 10.1007/s41669-022-00362-2

35. Williams EV, Goranitis I, Oppong R, Perry SJ, Devall AJ, Martin JT, et al. A cost-effectiveness analysis of early detection and bundled treatment of postpartum hemorrhage alongside the E-MOTIVE trial. Nat Med. 2024. doi: 10.1038/s41591-024-03069-5.

36. Seim AR, Alassoum Z, Souley I, Bronzan R, Mounkaila A, Ahmed LA. The effects of a peripartum strategy to prevent and treat primary postpartum haemorrhage at health facilities in Niger: a longitudinal, 72-month study. Lancet Glob Health. 2023;11(2):e287–e95. doi: 10.1016/S2214-109X(22)00518-6.

37. Bradley SE, Prata N, Young-Lin N, Bishai D. Cost-effectiveness of misoprostol to control postpartum hemorrhage in low-resource settings. Int J Gynaecol Obstet. 2007;97(1):52–6. doi: 10.1016/j.ijgo.2006.12.005.

38. World Health Organization. WHO recommendation on tranexamic acid for the treatment of postpartum haemorrhage. Geneva: World Health Organization, 2017.

39. Joshi BN, Shetty SS, Moray KV, Chaurasia H, Sachin O. Cost-effectiveness and budget impact of adding tranexamic acid for management of post-partum hemorrhage in the Indian public health system. BMC Pregnancy Childbirth. 2023;23(1):9. doi: 10.1186/s12884-022-05308-4.

40. Li B, Miners A, Shakur H, Roberts I. Tranexamic acid for treatment of women with post-partum haemorrhage in Nigeria and Pakistan: a cost-effectiveness analysis of data from the WOMAN trial. Lancet Glob Health. 2018;6(2):e222–e8. doi: 10.1016/S2214-109X(17)30467-9.

41. Howard DC, Jones AE, Skeith A, Lai J, D'Souza R, Caughey AB. Tranexamic acid for the treatment of postpartum hemorrhage: a cost-effectiveness analysis. Am J Obstet Gynecol MFM. 2022;4(3):100588. doi: 10.1016/j.ajogmf.2022.100588.

42. Sudhof LS, Shainker SA, Einerson BD. Tranexamic acid in the routine treatment of postpartum hemorrhage in the United States: a cost-effectiveness analysis. Am J Obstet Gynecol. 2019;221(3):275. e1–. e12. doi: 10.1016/j.ajog.2019.06.030.

43. World Health Organization. WHO recommendation on Uterine balloon tamponade for the treatment of postpartum haemorrhage. Geneva: World Health Organization, 2021.

44. Mvundura M, Kokonya D, Abu‐Haydar E, Okoth E, Herrick T, Mukabi J, et al. Cost‐effectiveness of condom uterine balloon tamponade to control severe postpartum hemorrhage in Kenya. Int J Gynaecol Obstet. 2017;137(2):185–91. doi: 10.1002/ijgo.12125.

45. Joshi BN, Shetty SS, Moray KV, Sachin O, Chaurasia H. Cost-effectiveness of uterine balloon tamponade devices in managing atonic post-partum hemorrhage at public health facilities in India. PLoS One. 2021;16(8):e0256271. doi: 10.1371/journal.pone.0256271.

46. Downing J, El Ayadi A, Miller S, Butrick E, Mkumba G, Magwali T, et al. Cost-effectiveness of the non-pneumatic anti-shock garment (NASG): evidence from a cluster randomized controlled trial in Zambia and Zimbabwe. BMC Health Serv Res. 2015;15(1):1–10. doi: 10.1186/s12913-015-0694-6.

47. Sutherland T, Downing J, Miller S, Bishai DM, Butrick E, Fathalla MM, et al. Use of the non-pneumatic anti-shock garment (NASG) for life-threatening obstetric hemorrhage: a cost-effectiveness analysis in Egypt and Nigeria. PloS One. 2013;8(4):e62282. doi: 10.1371/journal.pone.0062282.

48. World Health Organization. WHO recommendation on Umbilical vein injection of oxytocin for the treatment of retained placenta. Geneva: World Health Organization, 2020.

49. Ries J-J, Jeker L, Neuhaus M, Vogt DR, Girard T, Hoesli I. Implementation of the D-A-CH postpartum haemorrhage algorithm after severe postpartum bleeding accelerates clinical management: A retrospective case series. Eur J Obstet Gynecol Reprod Biol. 2020;247:225–31. doi: 10.1016/j.ejogrb.2020.01.001.
