# Supplementary material for: The cost-effectiveness of preventing, diagnosing, and treating postpartum haemorrhage: A systematic review of economic evaluations
Source: PLoS Med. 2024 Sep 13;21(9):e1004461. doi: 10.1371/journal.pmed.1004461 (PMC11433145; doi:10.1371/journal.pmed.1004461)
Supplement: S10 Appendix — (DOCX) [file pmed.1004461.s010.docx]

# **S10 Appendix: Results presented by region**

## Table A: Results from studies in the East Asia and Pacific region

| **Preventative Interventions** | | | | | |
| --- | --- | --- | --- | --- | --- |
| Study | Country | Intervention/s & Comparator/s   (dose and route if specified) | Results | Dominance / Cost-effectiveness | Summary of study conclusions |
| Wohling et al., 2019 [1] | Australia | 1. Carbetocin (100 μg IV)  2. Oxytocin (5-10 IU, IV slow push) | - Average cost per patient was reduced by $63.46 AUD, year not stated ($57.88 USD 2023) - Reduced rates of PPH >1000ml, 7.8% vs 9.7% (OR 0.79, 95% CI 0.59–1.05) - Significantly reduced rates of PPH >500ml, 27.3% vs 39.4% (OR 0.57, 95% CI 0.49– 0.68) - Reduced rates of additional treatment (26.9% vs 46.9%) - No difference in transfusions   First year of data collection (2008) used for cost conversion calculation. | Carbetocin dominant compared to oxytocin. | Carbetocin conferred an absolute cost reduction, but more detailed cost analyses should be completed in the future. |
| You et al., 2022 [2] | China | 1. Carbetocin (100 μg IV)  2. Oxytocin (10 IU IV bolus) | Base case:   - Reduced cost by $29 per birth, USD 2022 ($29.60 USD 2023). - Saved 0.00059 QALY per birth. - Reduced rates of PPH >500ml, 1000ml, hysterectomy, and maternal deaths.   OWSA:   - Carbetocin gained more QALYs than oxytocin in every analysis, but expected cost savings were sensitive to changes in relative effectiveness of carbetocin vs oxytocin.   PSA:   - Carbetocin dominated oxytocin in 99.7% of iterations and was cost-effective in 100%. | Carbetocin dominated oxytocin in 99.7% of iterations | From the public healthcare perspective in Hong Kong, carbetocin appeared to save total direct medical cost and QALYs for VB or CS. |
| Hong et al., 2022 [3] | China | 1. Internal iliac artery balloon occlusion prior to CS. Balloon inflated once baby delivered. Deflated after haemostasis achieved.   2. No occlusion before CS. | - The intervention was not effective at reducing the rates of any of the health outcomes of interest including blood loss or rate of hysterectomy but did increase the duration of surgery and the cost of hospitalization: ¥45,117 ± ¥9,359 vs ¥30,615 ± ¥11,587, CNY, year not stated ($14,839 ± $3,078 vs $10,069 ± $3,811 USD 2023).   First year of data collection (2015) used for cost conversion calculation. | Not cost-effective (No threshold set by author, but intervention not clinically effective). | Conclusions were not able to be drawn due to the heterogeneity of cases involved, retrospective study design, and results that showed little difference between intervention and comparator. |
| Xue et al., 2019 [4] | China | 1. MDT-ERAS intervention (a multimodal perioperative care pathway to achieve enhanced recovery after surgery).  2. Traditional perioperative care | - The intervention group experienced statistically significant lower proportion of PPH (5.94% vs 17.13%, P<0.01) - Average cost of hospitalization was also reduced in the intervention group, ¥3,552.39 vs ¥3,880.47 CNY, year not stated ($1,118.22 vs $1,221.49 USD 2023).   First year of data collection (2018) used for cost conversion calculation. | MDT-ERAS dominated standard care (however, hospitalization was the only cost considered). | MDT-ERAS for CS may improve clinical outcomes and the costs of admission. |
| Voon et al., 2018 [5] | Malaysia | 1. Carbetocin (100μg IV)  2. Oxytocin (5 IU IV) | Base Case:   - Carbetocin would avert 108 episodes of PPH, 104 episodes of transfusion and the need for 455 patients to receive additional uterotonics (per cohort of 3,000). - Carbetocin more expensive than oxytocin, ICER of $278.70 USD 2016 ($320.90 USD 2023) to avert one episode of PPH. | No threshold stated. | Authors described the ICER as favourable, but stated utilization would depend on the value an institution places on averting re-treatment, and their allocation of human resources. |
| Briones et al., 2020 [6] | Philippines | 1. Carbetocin (100 μg)  2. Oxytocin (10 IU) | Using carbetocin in place of oxytocin resulted in incremental health gains for incremental costs:   - CS: $13,187 USD 2019 ($14,317.25 USD 2023) per QALY gained. - VB: $43,164 ($46,863.55 USD 2023) per QALY gained | Carbetocin not cost-effective for either VB or CS at author's stated threshold of $2,895, USD 2019 (1x GDP per capita).  This is $3,143.13 in USD 2023.  0% chance of cost-effective in VB and 3% chance in CS. | Results suggestive that carbetocin is not cost-effective compared to oxytocin for either VB or CS in the Philippines. |
| Tsu et al., 2009 [7] | Vietnam | 1. Oxytocin Uniject (10 IU)  2. Oxytocin (10 IU drawn from ampoules)  3. No AMTSL | Base Case   - $15.70, USD 2004 ($22.59 USD 2023), per case of PPH averted with ampoules compared to no AMTSL. - $21.68 ($31.19 USD 2023), per case of PPH averted with Uniject compared to no AMTSL.   Best Case Scenario   - $7 ($10.07 USD 2023), per death averted with ampoules compared to no AMTSL. - $260 ($374.07 USD 2023), per death averted with Uniject compared to no AMTSL.   Worst Case Scenario   - $2508 ($3608.29 USD 2023), per death averted with ampoules compared to no AMTSL. - $3463 ($4982.26 USD 2023), per death averted with Uniject compared to no AMTSL. | No threshold stated. | The low net incremental cost of AMTSL suggests that the introduction of AMTSL in primary-level facilities in Vietnam can reduce the incidence of PPH and benefit women’s health without adding much to national health care costs. |
| **Diagnostic Interventions** | | | | | |
| No studies identified | | | | | |
| **Treatment Interventions** | | | | | |
| No studies identified | | | | | |
| **Bundle Interventions** | | | | | |
| No studies identified | | | | | |

Economic results are stated as they appear in the original publications and as a conversion to USD 2023 using an online tool developed by the Campbell and Cochrane Economics Methods Group (CCEMG) and the Evidence for Policy and Practice Information and Coordinating Centre (EPPI-Centre)[8]. Cost conversions were completed in December 2023 and may change slightly depending on final GDP figures.

Abbreviations: AMTSL: Active Management of the Third Stage of Labor. AUD: Australian Dollars. CI: Confidence Interval. CNY: Chinese Yuan. CS: Caesarean Section. GDP: Gross Domestic Product. ICER: Incremental Cost-Effectiveness Ratio. IV: Intravenous. MDT-ERAS: Multidisciplinary Team-Enhanced Recovery After Surgery. OR: Odds ratio. OWSA: One-Way Sensitivity Analysis. PPH: Postpartum Haemorrhage. PSA: Probabilistic Sensitivity Analysis. QALY: Quality-Adjusted Life Year. USD: United States dollar. VB: Vaginal Birth.

## Table B: Results from studies in the Europe & Central Asia region

| **Preventative Interventions** | | | | | |
| --- | --- | --- | --- | --- | --- |
| Study | Country | Intervention/s & Comparator/s   (dose and route if specified) | Results | Dominance / Cost-effectiveness | Summary of study conclusions |
| Pickering et al., 2019 [9]  Note: this analysis was part of a broader HTA - Gallos et al., 2019. Only results from analyses of VB are shown in this row, results on CS are shown below in Gallos et al., 2019. | United Kingdom | 1. Carbetocin 2. Ergometrine 3. Ergometrine plus Oxytocin 4. Misoprostol plus Oxytocin 5. Misoprostol 6. Oxytocin | For VB when considering the costs of SEs:   - Carbetocin was the most effective strategy. - Oxytocin was the least costly intervention. - Switching from oxytocin to carbetocin would incur £928 GBP 2016 ($1,530.93 USD 2023) per extra PPH>500ml averted or £22,900 ($37,778.41 USD 2023) per severe PPH case averted. - All other options were dominated by carbetocin. | No threshold stated | Current practice in this setting (oxytocin, carbetocin and ergometrine plus oxytocin) are all favourable strategies based on their relative cost-effectiveness. There was not sufficient evidence to suggest changing current practice at current prices. |
| Gallos et al., 2019 [10]  Note: analyses 1 and 2 (on VB) from this HTA were also published in Pickering et al., 2019 and are reported in the above row. Only results on CS are presented in this row. | United Kingdom | 1. Carbetocin  2. Ergometrine 3. Ergometrine plus Oxytocin 4. Misoprostol plus Oxytocin  5. Misoprostol  6. Oxytocin | For CS, when considering SEs, and excluding ergometrine or ergometrine plus oxytocin (lack of data):   - Carbetocin was the least costly intervention and second-most effective strategy. - Misoprostol plus oxytocin is the most effective strategy but is more costly than carbetocin. - All other prevention strategies are dominated by carbetocin, as they are both more costly and less effective than carbetocin. - The estimated ICER for prevention with misoprostol plus oxytocin compared with carbetocin is £2,480.19, GBP 2016 ($4,091.60 USD 2023), per case of PPH of ≥ 500 ml avoided. - If the impact of SEs were not taken into consideration (analysis 3), then Misoprostol plus oxytocin dominates all other strategies. | Mixed results depending on consideration of side-effects | The evidence generated in this review were not sufficient to dictate changes to practice in the UK due to the level of uncertainty and mixed results. |
| Higgins et al., 2011 [11] | United Kingdom | 1. Carbetocin (100μg IV)  2. Oxytocin (5IU) | - No difference in frequency of PPH observed. - Increase in average costs per delivery of £18.52 GBP, year not stated ($33.59 USD 2023).   Year of data collection (2010) used for cost conversion calculation. | Carbetocin neither dominant nor cost-effective compared to oxytocin. | Replacing oxytocin with carbetocin for PPH prophylaxis after CS is not beneficial to the patient or the delivery unit. |
| Luni et al., 2017 [12] | United Kingdom | 1. Carbetocin  2. Oxytocin | - Significantly lower rate of PPH than the oxytocin group (28% vs 43%). - No blood products required compared to 17 PRBCs in oxytocin group. - Reduced need for additional uterotonics (7% vs 48%). - Reduced average medication costs: £10.33 vs £33.98 GBP, cost year not stated ($17.47 vs $57.46 USD 2023). - Reduced midwifery costs: £7.69 vs £52.97 ($13.00 vs $89.57 USD 2023)   First year of data collection (2014) used for cost conversion calculation. | Carbetocin dominant compared to oxytocin. | Using carbetocin for PPH prophylaxis in CS reduced the need for blood products, additional uterotonics, and midwifery workload, and has significant potential to provide savings to the delivery suite. |
| van der Nelson et al., 2017 [13] | United Kingdom | 1. Carbetocin (100μg IV)  2. Oxytocin (5 IU IV) | Base Case:   - Carbetocin was less costly, saving £27,518.41 GBP, year not stated ($48,214.13 USD 2023) per cohort of 1500 deliveries, and more effective with 30 PPH events averted.   PSA:   - Carbetocin dominated in 69.4% of scenarios and was cost-effective in 70.5% of scenarios at author's stated threshold of £20,000 ($35,041 USD 2023) per QALY.   Year of NHS reference costs from study (2012) used for cost conversion calculation. | Carbetocin dominant compared to oxytocin (in 69.4% of iterations) | Carbetocin is likely to result in better clinical outcomes and a modest cost-saving compared to oxytocin, although there is uncertainty. |
| Matthijsse et al., 2022 [14] | United Kingdom | 1. Carbetocin (100μg IM)  2. Oxytocin (10IU bolus) | Base case:   - Carbetocin was less costly, -£55 GBP 2019 ($85.55 USD 2023), and more effective with 0.0342 less PPH events, or 0.0001 QALYs gained per woman.   OWSA:   - Results were robust to all variations tested.   PSA:   - Carbetocin dominated in 79.5% of runs. | Carbetocin dominant compared to oxytocin. | Carbetocin was found to cost-effective from a UK NHS perspective for the prevention of PPH following VB. |
| Denison et al., 2019 [15,16] | United Kingdom | 1.  Two puffs of GTN (400µg sublingual)  2. Placebo spray (sublingual) | GTN was not effective at reducing the requirement for manual removal of placenta or the risk of PPH. | Not dominant nor cost-effective. | The GTN group incurred higher costs (not statistically significant) for no improvement in clinical, safety or patient-oriented outcomes. |
| Durand-Zaleski et al., 2021 [17] | France | 1. TXA (1 gram slowly IV over 2 mins) in addition to standard care.   2. Placebo and standard care. | - No statistically significant difference in costs between the intervention and comparator. - No statistically significant difference in rates of PPH>500ml - the number of "provider-assessed clinically significant postpartum haemorrhage" was reduced (7.8% vs. 10.4%) and the proportion requiring secondary uterotonics was also lower (7.2% vs 9.7%)   PSA:   - 65–73% probability that TXA is both cost-saving and event-reducing. | TXA dominated routine care. | Prophylactic use of TXA at VB reduces both costs and bleeding events with a probability greater than 60% but less than 80%. |
| Sentilhes et al., 2023 [18] | France | 1. TXA in addition to standard care  2. Placebo in addition to standard care | - Mean length of stay was slightly lower for intervention group (4.8 days vs 4.9 days) - Proportion of women with no complication up to 90 days was higher in intervention group (70.7% vs 66.0%) - Mean total cost (until the occurrence of complications) were slightly higher in the intervention group, €3,321 vs €3,260, EUR 2019 ($4,820.95 vs $4,732.40, USD 2023) - ICER of €762 ($1,106.16 USD 2023) per additional CS delivery without complication at day 90 | TXA strategy had 99.9% probability of being cost-effective at the author’s stated threshold of €10,000 per additional CS delivery without complication at day 90. | Prophylactic use of tranexamic acid is cost-effective for reducing complications, including PPH, in women undergoing CS. However, the overall difference in effectiveness and costs between the two groups is low. |
| Niola et al., 2017 [19] | Italy | 1. Intravascular uterine artery occlusion immediately before delivery   2. Surgery or embolization after delivery | - Intervention group had lower rates of transfusion (36% vs 100%) and lower rates of hysterectomy (26% vs 43.4%) - Intervention group had lower average costs per patient, €7,607.77 vs €13,925.04, EUR, year not stated ($12,526.40 vs $22,927.95 USD 2023).   First year of data collection (2009) used for cost conversion calculation. | Intervention dominant compared to standard post-delivery embolization. | In this sample of women with placental implant anomalies, predelivery uterine artery embolization was a cost-effective procedure. |
| **Diagnostic Interventions** | | | | | |
| Study | Country | Intervention/s & Comparator/s   (dose and route if specified) | Results | Dominance / Cost-effectiveness | Summary of study conclusions |
| No studies identified | | | | | |
| **Treatment Interventions** | | | | | |
| Study | Country | Intervention/s & Comparator/s   (dose and route if specified) | Results | Dominance / Cost-effectiveness | Summary of study conclusions |
| Edwards et al., 2023 [20] | United Kingdom | 1. Butterfly device (facilitates compression of uterus as an alternative to bimanual compression)   2. Standard care | Base case:   - Treatment with the intervention resulted in reduced PPH progression beyond 1000ml after device use (1.75% vs 7.97%) compared to standard care. - Mean ICER of £3,795.78 GBP 2017 ($6,126.91 USD 2023) per PPH progression avoided (defined as > 1000ml blood loss after point of device use).   PSA:   - At a threshold of £8,500 ($13,720 USD 2023) per PPH progression avoided, the intervention has a probability of being cost-effective of 87%. | No comparable threshold available. | The Butterfly device is a relative low-cost device in a UK NHS setting with a high probability of being cost-effective. |
| Prick et al., 2014 [21] | Netherlands | 1. RBC transfusion aiming for target Hb 8.9g/dl.  2. Conservative management (iron and or folic acid supplementation and only utilizing transfusion if clinically indicated). | Transfusing to target Hb incurred an incremental cost of €431 EUR 2013 ($627 USD 2023) for each 1-point improvement on the Multidimensional Fatigue Inventory (MFI). | No threshold set | In women with acute anaemia after PPH, RBC transfusion is on average €249 ($362 USD 2023) more expensive per woman than non-intervention, with only a small gain in fatigue scores following transfusion. A policy of non-intervention appears justified for women in these circumstances (Hb in range 4.8-7.9 g/dl and no symptoms of severe anaemia). |
| Ries et al., 2020 [22] | Switzerland | 1. Implementation of the “D-A-CH Handlungsalgorithmus Postpartale Blutung” algorithm for the management of PPH  2. PPH management prior to the implementation of the D-A-C-H algorithm. | - There was no significant difference in clinical outcomes (estimated blood loss, ICU transfers and Hb value two days postpartum). - Implementation of the algorithm did however result in the usage of a wider range of pharmacological interventions for PPH treatment within a shorter time interval after delivery. - Treatment in the intervention group was not statistically more expensive on average than before the implementation of the algorithm: control ₣434.7, vs intervention ₣233.9 CHF, year not stated, p=0.571 ($377.16 vs $202.94 USD 2023).   First year of data collection (2009) used for cost conversion calculation. | No threshold set. | Implementation of the treatment algorithm in women after VB with severe PPH did not result in significantly reduced blood loss. Implementation did however accelerate clinical management and induced the application of a wider range of pharmacological interventions closer to the time of delivery and did not generate more costs. |
| **Bundle Interventions** | | | | | |
| Study | Country | Intervention/s & Comparator/s   (dose and route if specified) | Results | Dominance / Cost-effectiveness | Summary of study conclusions |
| Dale et al., 2022 [23] | Wales | 1. Standard care, plus universal risk assessments for PPH, quantitative blood loss measurement, multidisciplinary team management of PPH, point of care coagulation blood testing after 1000mL blood loss to guide resuscitation.    2. Standard care in Wales prior to the above nationwide process improvements. | - No difference in the number of cases of PPH>1000mL. - Reduction in the number of cases progressing from PPH>1000mL to PPH>2500mL from 6.9 per 1000 births to 5.2 per 1000 births. - Decrease in resource use for blood products, critical care, and haematologist time. - Incremental cost of £18.41 GBP 2018 ($29.16 USD 2023) per patient with PPH >1000mL. | No threshold specified | The intervention reduced the occurrence of massive PPH, the quantity of blood products used, and intensive care resources utilised. The incremental costs incurred decrease towards cost-neutrality in medium-large (>3000 annual births) maternity units. |

Economic results are stated as they appear in the original publications and as a conversion to USD 2023 using an online tool developed by the Campbell and Cochrane Economics Methods Group (CCEMG) and the Evidence for Policy and Practice Information and Coordinating Centre (EPPI-Centre) [8]. Cost conversions were completed in December 2023 and may change slightly depending on final GDP figures.

Abbreviations: CHF: Swiss Franc. CS: Caesarean Section. EUR: Euro. GBP: British Pound. GTN: Glyceryl trinitrate. Hb: Haemoglobin. HTA: Health Technology Assessment. ICER: Incremental Cost-Effectiveness Ratio. ICU: Intensive Care Unit. IM: Intramuscular. IV: Intravenous. mL: Millilitre. MFI: Multidimensional Fatigue Inventory. NHS: National Health Service. OR: Odds ratio. OWSA: One-Way Sensitivity Analysis. PPH: Postpartum Haemorrhage. PRBCs: Packed Red Blood Cells. PSA: Probabilistic Sensitivity Analysis. QALY: Quality-Adjusted Life Year. RBC: Red Blood Cell. SEs: Side Effects. TXA: Tranexamic Acid. UK: United Kingdom. USD: United States dollar. VB: Vaginal Birth.

## Table C: Results from studies in the Latin America & Caribbean region

| **Preventative Interventions** | | | | | |
| --- | --- | --- | --- | --- | --- |
| Study | Country | Intervention/s & Comparator/s   (dose and route if specified) | Results | Dominance / Cost-effectiveness | Summary of study conclusions |
| Gil-Rojas et al., 2018 [24] | Colombia | 1. Carbetocin (100 μg)  2. Oxytocin (5-10 IU IM for VB or 5IU followed by 30IU infusion for CS) | CS:  Each PPH prevented saved $94,887 COP 2016 ($86.88 USD 2023).  In 52% of PSA iterations carbetocin dominated and in 15.3% it resulted in increased costs but below the cost-effective threshold.  VB:  Carbetocin was the most effective treatment but was more costly.  ICER of $974,790,719 COP 2016 ($1,091,828 USD 2023) per QALY gained.  In only 27% of iterations carbetocin is the dominant alternative and in 7.3% of iterations it is more costly but below the willingness to pay threshold.   - In 46.8% of iterations oxytocin remained the dominant alternative. | CS:  Carbetocin either dominates or is cost-effective compared to oxytocin (67.3%).  VB:  Carbetocin not cost-effective at author's stated threshold of $53,090,199 COP 2016 (3x GDP per capita).  This is $59,464.47 in USD 2023. | The model for CS showed Carbetocin delivered lower costs and better health outcomes. The model for VB showed carbetocin delivered incremental health gains but at a cost above the specified threshold in Colombia. |
| Henriquez-Trujillo et al., 2017 [25] | Ecuador | 1. Carbetocin  2. Oxytocin | Base Case:   - ICER $2,432.89 USD 2015 ($2831.87 USD 2023) per DALY averted.   PSA:   - Mean ICER $3387.69 ($3943.26 USD 2023) per DALY averted - 95% CI: $3,307.18–3,468.20 ($3849.55 - 4036.97 USD 2023). - 97% of the ICER iterations were deemed very cost-effective. | Carbetocin cost-effective compared to oxytocin at author's stated threshold of $6302 (1x GDP per capita).  In USD 2023 this is $7335.50. | For the primary prevention of PPH after CS in Ecuador, carbetocin is highly cost-effective for both elective and emergency deliveries. |
| Caceda et al., 2018 [26] | Peru | 1. Carbetocin as first line for prevention  2. Oxytocin as first line for prevention | Using carbetocin in place of oxytocin resulted in incremental health gains for incremental costs: Base Case   - S/49,918, PEN 2015, ($37,314.47 USD 2023) per QALY gained.   OWSA   - Results reported as robust.   PSA   - S/119,178 ($89,087.37 USD 2023) per QALY gained. | Carbetocin cost-effective compared to oxytocin at author's stated threshold of S/132,699 (3 x GDP per capita).  In USD 2023 this is $99,194.52. | Carbetocin more cost-effective than oxytocin for prevention of PPH after CS in this setting. |
| Diaz et al., 2009 [27] | Peru | 1. Implementation of AMTSL with oxytocin, equipment upgrades, and staff training.   2. No active implementation of AMTSL, equipment upgrades or staff training. | - Estimated 15,335 cases of PPH averted between 2001 – 2005. - Incremental cost of $3,328 USD, year not stated ($5,087 USD 2023) per averted case >500ml or $29,897 ($45,703 USD 2023) per averted case >1000ml.   First year of data collection (2001) used for cost conversion calculation. | No threshold stated | The programme reduced the incidence of PPH, the main cause of maternal mortality. |
| Fullerton et al., 2006 [28] | Guatemala | 1. AMTSL (uterotonic not specified)  2. EMTSL | Guatemala specific results:   - 100 maternal deaths averted and net cost-saving of $ 18,000 USD 2004 ($25,896.82 USD 2023) per 100,000 births. - Results were robust to a wide range of OWSA scenarios. | AMTSL strategy dominated EMTSL strategy in both settings. | AMTSL is associated with a distinct financial benefit to health facilities in addition to clinical benefits. |
| Pichon-Riviere et al., 2015 [29] | Multiple countries in region | 1. Oxytocin (10 IU IM or 5 IU IV drawn from ampoules)  2. Oxytocin Uniject (10 IU IM) | - Uniject reduced PPH events and deaths, and increased QALYs in all 30 countries analysed. - Incremental QALYs gained per 1,000 institutional deliveries ranged from 0.02 to 0.71. - In 27% of the countries, Uniject was cost saving. In the remaining 22 countries, Uniject was associated with an incremental cost between $ 0.005 to $0.85, USD 2013 ($0.006 to $1.02 USD 2023), per delivery.   (Note: this model assumed that utilizing Uniject would increase the proportion of deliveries with access to Oxytocin). | Uniject was cost-effective compared to ampoules in all 30 countries at the author's stated threshold (3 x GDP per capita of that country) with many likely cost-effective at lower thresholds (1x GDP per capita). | Uniject was modelled as either cost-saving or very cost-effective in almost all countries in Latin America and the Caribbean. Even if countries achieve only small increases in oxytocin use by incorporating Uniject, this strategy could be considered an efficient use of resources. |
| **Diagnostic Interventions** | | | | | |
| No studies identified | | | | | |
| **Treatment Interventions** | | | | | |
| No studies identified | | | | | |
| **Bundle Interventions** | | | | | |
| No studies identified | | | | | |
|  | | | | | |

Economic results are stated as they appear in the original publications and as a conversion to USD 2023 using an online tool developed by the Campbell and Cochrane Economics Methods Group (CCEMG) and the Evidence for Policy and Practice Information and Coordinating Centre (EPPI-Centre) [8]. Cost conversions were completed in December 2023 and may change slightly depending on final GDP figures.

Abbreviations: AMTSL: Active Management of the Third Stage of Labor. CI: Confidence Interval. COP: Colombian Peso. CS: Caesarean Section. DALY: Disability-Adjusted Life Year. EMTSL: Expectant Management of the Third Stage of Labor. GDP: Gross Domestic Product. ICER: Incremental Cost-Effectiveness Ratio. IM: Intramuscular. IU: International Units. IV: Intravenous. ml: Millilitre. OWSA: One-Way Sensitivity Analysis. PEN: Peruvian Sol. PPH: Postpartum Haemorrhage. PSA: Probabilistic Sensitivity Analysis. QALY: Quality-Adjusted Life Year. USD: United States Dollar. VB: Vaginal Birth.

## Table D: Results from studies in the Middle East & North Africa region

| **Preventative Interventions** | | | | | |
| --- | --- | --- | --- | --- | --- |
| No studies Identified | | | | | |
| **Diagnostic Interventions** | | | | | |
| No studies Identified | | | | | |
| **Treatment Interventions** | | | | | |
| Study | Country | Intervention/s & Comparator/s   (dose and route if specified) | Results | Dominance / Cost-effectiveness | Summary of study conclusions |
| Sutherland et al., 2013 [30] | Egypt | 1. Adding NASG to standard management of women with severe hypovolemic shock (MAP<60mmHg) due to obstetric haemorrhage.   2. Adding NASG to standard management of women with any degree of shock due to obstetric haemorrhage.   3. Standard care with no NASG | Egypt specific results:   - Using the NASG for cases of severe shock resulted in decreased deaths, hysterectomies, and severe morbidity (357 DALYs averted) and saved $9,489 international dollars 2010 ($12,042 USD 2023), per 1000 women with shock. - When applying NASG to all women with shock a further 37 DALYs were averted and an extra $21,253 ($26,972 USD 2023) were saved. | Egypt specific analysis:  Both applying NASG to women with any shock or severe shock dominated standard care. | The NASG is either cost saving or highly cost-effective for women in severe hypovolemic shock when administered in a tertiary care setting. |
| **Bundle Interventions** | | | | | |
| No studies Identified | | | | | |

Economic results are stated as they appear in the original publications and as a conversion to USD 2023 using an online tool developed by the Campbell and Cochrane Economics Methods Group (CCEMG) and the Evidence for Policy and Practice Information and Coordinating Centre (EPPI-Centre) [8]. Cost conversions were completed in December 2023 and may change slightly depending on final GDP figures.
Abbreviations: DALYs: Disability-Adjusted Life Years. MAP: Mean Arterial Pressure. NASG: Non-pneumatic Anti-Shock Garment. USD: United States Dollar.

## Table E: Results from studies in the North America region

| **Preventative Interventions** | | | | | |
| --- | --- | --- | --- | --- | --- |
| Study | Country | Intervention/s & Comparator/s   (dose and route if specified) | Results | Dominance / Cost-effectiveness | Summary of study conclusions |
| Barrett et al., 2022 [31] | Canada | 1. Carbetocin as first line prophylactic agent  2. Oxytocin as first line prophylactic agent | In a cohort of 3,242 patients:   - 76 PPHs from low-risk VB averted. - 73 PPH from high-risk VB averted. - 154 PPH in CS averted. - Cost savings of $349,000 CAD 2020 ($307,692.81 USD 2023) | Carbetocin dominant compared to oxytocin. | Replacing carbetocin with oxytocin as first-line PPH prevention would reduce costs in this setting. |
| Dazelle et al., 2023 [32] | United States of America | 1. Prophylactic TXA (1g) to all women  2. Prophylactic TXA (1g) to women at high risk of PPH  3. Prophylactic TXA (1g) to women at high or moderate risk of PPH  4. Routine care | Base Case:   - All TXA strategies were considered superior in cost-savings and outcomes averted relative to routine care. - The most effective strategy was providing TXA to all births which resulted in cost-savings of $690 million USD 2020 ($734 million USD 2023) and prevented 149,505 PPH cases, 19,447 balloon tamponades, 24,079 uterus-sparing surgeries, 2,933 hysterectomies, and 70 maternal deaths per year.   OWSA:   - The results were robust to variation in the value of all assessed variables.   PSA:   - Prophylactic TXA strategies are cost-saving versus the status quo in >99.9% of simulations. | All interventions dominated routine care. | Routine TXA prophylaxis for delivering women in the US is likely to result in substantial cost-savings and reductions in maternal morbidity and mortality. |
| **Diagnostic Interventions** | | | | | |
| Study | Country | Intervention/s & Comparator/s   (dose and route if specified) | Results | Dominance / Cost-effectiveness | Summary of study conclusions |
| Katz et al., 2020 [33] | United States of America | 1. Triton system for blood measurement (gravimetric and colorimetric).   2. Visual estimation by obstetrician, nursing staff or anaesthetist | - The intervention group reported a higher proportion of deliveries diagnosed with PPH in both VB (2.2% vs 0.5%) and CS (12.6% vs 6.4%). - The intervention group recorded less blood loss on average in VB (300 mL vs 258mL) and CS (800mL vs 702mL) - The intervention group reported higher secondary use of uterotonics (22% vs 17.3%) - No difference in the use of blood products between groups - No difference between groups for blood bank costs. - On average laboratory costs were $4 USD, year not stated, cheaper per patient in the intervention ($4.61 USD 2023).   First year of data collection (2016) used for cost conversion calculation. | Not calculated. | Use of quantitative blood measurement in this setting resulted in increased vigilance at VB and CS and improved the identification of PPH and the secondary use of uterotonics. The authors also state that the cost saving from reduced laboratory costs would result in 152% return on investment, but this analysis does not include many other costs. |
| **Treatment Interventions** | | | | | |
| Study | Country | Intervention/s & Comparator/s   (dose and route if specified) | Results | Dominance / Cost-effectiveness | Summary of study conclusions |
| Howard et al., 2022 [34] | United States of America | 1. TXA (1g) plus standard management   2. Standard care | Base Case:   - Intervention would avert 6 exploratory laparotomies following VB, 112 reoperations after CS, and 11 deaths per 100,000 deliveries. - This is a gain of 329 QALYs. - Intervention would save $15.39 million, USD 2019, annually.  ($16.71 million USD 2023) - If administered early (<3h)  intervention would gain 438 QALYs and save $23.15 million ($25.13 million USD 2023) annually per 100,000 deliveries.   PSA:   - Early administration of TXA for the treatment of PPH was dominant in 99.8% of samples. | TXA dominated standard care. | TXA is a cost-effective strategy for reducing morbidity and mortality from PPH in the USA. |
| Sudhof et al., 2019 [35] | United States of America | 1. TXA given at any time.  2. TXA given within 3 hours of delivery.  3. Standard care (no TXA) | Base Case:   - Intervention saved $11.3 million, USD 2018 ($12.49 million USD 2023), prevented 334 laparotomies, and averted 9 maternal deaths in the USA annually assuming 4 million births with a 3% rate of PPH. - Giving TXA <3h from delivery almost tripled the cost savings and improved maternal outcomes much further. For an annual US cohort, it would prevent 924 more laparotomies, 5 additional maternal deaths, and saved an additional $18.8 million ($20.77 million USD 2023).   PSA:   - TXA strategies were cost saving in >99.9% of simulations | Both TXA strategies dominated the alternative with TXA <3h being the most dominant. | Routine TXA early in the treatment of PPH is likely to be cost saving in the United States. |
| Einerson et al., 2017 [36] | United States of America | 1. Universal type and screen plus cross match for high-risk patients   2. Universal type and screen only   3. Universal hold clot plus cross match for high-risk patients   4. Selective type and screen only in high-risk patients   5. No routine admission testing | - Strategy 1 generated an ICER of $115,541 USD 2015 ($134,489 USD 2023) per emergency-release transfusion prevented compared with strategy 3 (the next most effective strategy). - The ICER for strategy 3 was $2,878 ($3350 USD 2023) per emergency-release transfusion prevented compared with strategy 5. | Strategy 5 was the most cost-effective at the author's prespecified threshold of $1,500 ($1,746 USD 2023) to prevent one emergency-release transfusion (76.1% of PSA iterations). | Universal type and screen strategies were not cost-effective in a general obstetric population even when considering a wide range of assumptions, variable ranges, and willingness-to-pay thresholds. The small incremental gains in prevention of emergency-release transfusion were not offset by the added costs of the transfusion preparedness strategies. |
| Snegovskikh et al., 2018 [37] | United States of America | 1. Blood product resuscitation guided by point of care viscoelastic testing (PCVT)  2. Empiric blood product resuscitation | - Estimated blood loss, post-operative ICU admission and the incidence of hysterectomy were significantly lower in the PCVT group. - The average cost of hospitalization was lower for the patients in the PCVT group: $11,802.94 vs $20,419.08 USD, year not stated ($14,672.66 vs $25,383.69 USD 2023).   First year of data collection (2011) used for cost conversion calculation. | Only aggregate hospital costs presented – unclear if overall cost-effective assessment can be made. | PCVT-based protocols conferred a reduction in the need for PRBC, FFP, and platelet concentrate transfusions in the setting of severe PPH. This individualised approach may also result in less intraoperative blood loss, lower rates of puerperal hysterectomy and postoperative ICU admissions, as well as a reduce the length of hospital stay and cost of hospitalization. |
| Lim et al., 2018 [38] | United States of America | 1. Cell salvage for all CS.  2. Cell salvage only for deliveries at high risk for haemorrhage (including placenta previa, placenta accreta, repeat CS or multiparity, chorioamnionitis, placental abruption, hypertensive disorders during pregnancy etc.)  3. No utilization of cell salvage. | Base Case:   - The incremental cost incurred for using cell salvage on high-risk CS cases was $34,881 USD 2012 ($42,546 USD 2023) per QALY gained. - The incremental cost incurred for using cell salvage on all CS cases was $415,488 ($506,787 USD 2023) per QALY gained.   OWSA:   - Results were not sensitive to individual variation of other model parameters.   PSA:   - At the $100,000 ($121,974 USD 2023) per QALY gained threshold, there is more than 85% likelihood that cell salvage use for cases at high risk for haemorrhage is cost-effective. | Cell salvage for high-risk CS:  cost-effective at author's stated threshold of $100,000 ($121,974 USD 2023) per QALY gained.   Cell salvage for all CS: Not cost-effective. | Cell salvage for cases at high risk for haemorrhage is economically reasonable compared to strategies of cell salvage use for all CS or no cell salvage use at all. |
| **Bundle Interventions** | | | | | |
| Study | Country | Intervention/s & Comparator/s   (dose and route if specified) | Results | Dominance / Cost-effectiveness | Summary of study conclusions |
| Wiesehan et al., 2023 [39] | United States of America | 1. California's statewide perinatal quality collaborative initiative, including: haemorrhage cart, rapid access to PPH medications, dedicated haemorrhage response team, massive transfusion protocol, staff training and drills, haemorrhage risk assessments, department wide active management of third stage of labour with oxytocin, and many other elements (complete list in Table 1 of this study).  2. Standard care in Californian hospitals not taking part in the quality collaborative | Base Case   - The intervention was modelled to increase QALYs by 0.000379 and reduce costs by $17.78 USD 2021 ($18.52 USD 2023) per birth.   OWSA:   - Cost-effective in every analysis (at author’s stated threshold of $100,000 ($104,184 USD 2023) per QALY gained).   PSA:   - Cost saving in 83% of samples and cost-effective in 99% of samples (at stated threshold). | Bundle intervention dominant compared to standard care. | The intervention is inexpensive, reduces severe maternal morbidity and mortality and is potentially cost saving. |

Economic results are stated as they appear in the original publications and as a conversion to USD 2023 using an online tool developed by the Campbell and Cochrane Economics Methods Group (CCEMG) and the Evidence for Policy and Practice Information and Coordinating Centre (EPPI-Centre) [8]. Cost conversions were completed in December 2023 and may change slightly depending on final GDP figures.

Abbreviations: CAD: Canadian Dollar. CS: Caesarean Section. FFP: Fresh Frozen Plasma. ICER: Incremental Cost-Effectiveness Ratio. ICU: Intensive Care Unit. mL: Millilitre. OWSA: One-Way Sensitivity Analysis. PCVT: Point of Care Viscoelastic Testing. PPH: Postpartum Haemorrhage. PRBC: Packed Red Blood Cells. PSA: Probabilistic Sensitivity Analysis. QALY: Quality-Adjusted Life Year. TXA: Tranexamic Acid. US: United States. USD: United States Dollar. VB: Vaginal Birth.

## Table F: Results from studies in the South Asia region

| **Preventative Interventions** | | | | | |
| --- | --- | --- | --- | --- | --- |
| Study | Country | Intervention/s & Comparator/s   (dose and route if specified) | Results | Dominance / Cost-effectiveness | Summary of study conclusions |
| Cook et al., 2023 [40] | India | 1. Carbetocin  2. Oxytocin  3. Misoprostol | Base Case 1   - Carbetocin in place of oxytocin reduced PPHs, deaths, DALYs and saved $171,700 USD 2021 ($178,884.78 USD 2023) per 100,000 births.   Base Case 2   - Carbetocin in place of misoprostol reduced PPHs, deaths, DALYs and saved $230,248 ($239,882.72 USD 2023) per 100,000 births.   OWSA   - Results reported as robust.   PSA   - Carbetocin dominant in 98% of iterations against oxytocin and 98.9% against misoprostol. | Carbetocin dominant compared to oxytocin and misoprostol. | Carbetocin is a cost-effective intervention and will result in improved health outcomes and lower costs to the Indian public health system. |
| Sutherland et al., 2009 [41] | India | 1. Misoprostol (600 μg administered by VHW).  2. VHW attendance but no uterotonic. | - Intervention resulted in 38% (95% CI, 5%–73%) reduction in maternal deaths. - ICER of $1,401 USD 2008 ($1,812.44 USD 2023) per life saved. - IQR of ICER $1008–$1848 ($1,304.02 – 2,390.71 USD 2023). | No threshold stated. | Authors conclude that misoprostol is cost-effective and could potentially save tens of thousands of lives each year at low cost. |
| Sutherland et al., 2010 [42]  Note: this study appears in both prevention and treatment sections of this review. | India | 1. Misoprostol Prevention: 600μg oral misoprostol, if they haemorrhage >1000ml they have 75% chance of referral to health centre.   2. Misoprostol Treatment: 800μg sublingual misoprostol after 700ml blood loss.  3. Standard care with unskilled assistant and no medication | - Misoprostol for PPH prevention was the most effective and costly intervention. - In addition to the DALYs averted and costs incurred in the misoprostol for PPH treatment strategy, misoprostol prevention would avert a further 33.6 DALYs and incur an additional cost of $5,721 USD 2009 ($7,345.09 USD 2023) per 10,000 deliveries. - ICER of $170 ($218.26 USD 2023) per DALY averted. | Misoprostol prevention is very cost-effective compared to alternatives at author's stated threshold of $2,600 (1x GDP per capita).  This is $3338.10 in USD 2023. | Misoprostol for prevention is very cost-effective for decreasing mortality and anaemia compared to standard care. |
| Goldie et al., 2010 [43] | India | 1. Misoprostol distribution in community (in home and birthing centres) in addition to general infrastructure and service upgrades.  2. Implementing service and infrastructure upgrades without adding misoprostol distribution. | Intervention resulted in   - Cost savings of $120 - $198 million USD 2006 ($162 to $268 million USD 2023) over a lifetime time horizon. - Reduction in maternal deaths of 6.9% - 12.3%. | Misoprostol strategy dominated alternative. | Although not a substitute for reliable obstetric care, community-based distribution of oral misoprostol in homes and birthing centres is likely to be cost-effective intervention. |
| Sharma et al., 2023 [44] | India | 1. Negative intrauterine pressure suction device  integrated with AMTSL (with oxytocin)  2. AMTSL (with oxytocin) alone | - Women that received the intervention had on average less blood loss (216.66ml vs 389.45ml). - A lower proportion of women receiving the intervention had PPH (0.49% vs 1.81%, p<0.001). - Women treated with AMTSL alone incurred higher costs secondary to blood product use, no other costs were considered. | No threshold stated. | In low resource settings, adding the use of negative intrauterine pressure suction devices can be instrumental in decreasing the incidence of PPH. However, further large multi-centre trials are required before drawing solid conclusions. |
| Carvalho et al., 2020 [45] | Bangladesh | 1. Inhaled oxytocin (note: not yet a licensed product)  2. Standard of care in that country and setting (different uterotonics depending on the setting) | Bangladesh Base Case   - IHO introduction would avert over 18,500 PPH cases and 76 maternal deaths annually and save $716,000, USD 2017 ($809,031.98 USD 2023), per year.   OWSA   - Results were cost saving in all but one of the scenarios tested.   Ethiopia Base Case:   - IHO introduction would avert 3000 PPHs annually and 30 maternal deaths at an incremental cost of $1,443,000 ($1,630,493.22 USD 2023). - $464 per PPH averted  (524.29 USD 2023) - $47,557 per maternal life saved ($53,736.22 USD 2023).   OWSA:   - IHO was not cost-effective in any of the OWSA scenarios. | Bangladesh specific results: Inhaled oxytocin dominant compared to standard care. | In settings like Bangladesh, where there is limited access to oxytocin, Inhaled Oxytocin could be a cost-saving intervention, as health impacts are accompanied by a substantial reduction in spending on PPH treatment. In the Ethiopian context, the product may not be considered a cost-effective intervention until the product is fully integrated into the health system. |
| **Diagnostic Interventions** | | | | | |
| Study | Country | Intervention/s & Comparator/s   (dose and route if specified) | Results | Dominance / Cost-effectiveness | Summary of study conclusions |
| No studies identified | | | | | |
| **Treatment Interventions** | | | | | |
| Study | Country | Intervention/s & Comparator/s   (dose and route if specified) | Results | Dominance / Cost-effectiveness | Summary of study conclusions |
| Sutherland et al., 2010 [42]  Note: this study appears in both prevention and treatment sections of this review. | India | 1. Misoprostol Prevention: 600μg oral misoprostol, if they haemorrhage >1000ml they have 75% chance of referral to health centre.   2. Misoprostol Treatment: 800μg sublingual misoprostol after 700ml blood loss.  3. Standard care with unskilled assistant and no medication | - Misoprostol for PPH prevention was the most effective and costly intervention. - In addition to the DALYs averted and costs incurred in the misoprostol for PPH treatment strategy, misoprostol prevention would avert a further 33.6 DALYs and incur an additional cost of $5,721 USD 2009 ($7,345.09 USD 2023) per 10,000 deliveries. - ICER of $170 ($218.26 USD 2023) per DALY averted. | Misoprostol prevention is very cost-effective compared to alternatives at author's stated threshold of $2,600 (1x GDP per capita).  This is $3338.10 in USD 2023. | Misoprostol for prevention is very cost-effective for decreasing mortality and anaemia compared to standard care. |
| Joshi et al., 2023 [46] | India | 1. TXA (1g IV within 3 hours of birth) plus standard care. An additional dose of TXA was given if bleeding continued after 30 min or if it restarted within 24 h.   2. Standard care | Base Case   - Intervention would incur ₹121 INR 2019/20 ($7.08 USD 2023) and gain 0.082 QALYs per woman treated. - This equates to an ICER of ₹1,470 ($86.03 USD 2023) per QALY gained. - Per annual cohort of 510,915 with PPH, intervention would avert 905 surgeries, 655 ICU admissions, and 1990 maternal deaths compared to standard care.   PSA:   - For an Indian willingness to pay (WTP) threshold value of one-time GDP per capita, the analysis suggested that 94.5% of simulations are cost-effective. | TXA cost-effective compared to standard care at author's stated threshold of 1 x GDP per capita ₹145,742 ($8,529 USD 2023). | Early administration of TXA to women with PPH in Indian public health facilities is recommended from a cost-effectiveness perspective. |
| Joshi et al., 2021 [47] | India | 1. ESM-UBT  2. Bakri®-UBT  3. Condom-UBT (improvised) which is standard care | Base Case:   - ESM-UBT versus condom-UBT has an ICER value of ₹-2,412 INR 2017 (-$153 USD 2023) per DALY averted meaning an incremental cost-saving of ₹2,412 (or $153 USD 2023) occurs per incremental DALY averted. - Bakri®-UBT was less effective and more costly than condom-UBT.   PSA:   - Comparing ESM-UBT versus Condom-UBT: 63.5% of the simulations at the given WTP threshold were cost-effective (52% dominant). | ESM-UBT dominated standard care with condom-UBT in 52% of simulations and was cost-effective in 63.5% at authors stated threshold of ₹24,211 (1,536 USD 2023). This was reported as a high degree of uncertainty. | Condom-UBT device as recommended for atonic PPH management in India offers better value as compared to Bakri®-UBT in this setting. ESM-UBT could be a cost-saving alternative, but this needs further evaluation as the differences in costs and health outcomes are marginal, there is a high degree of uncertainty. |
| Li et al., 2018 [48] | Pakistan | 1. TXA plus routine care  2. Placebo plus routine care | Pakistan specific results  Base Case:   - 0.08 QALYs gained for an additional cost of $6.55 ($7.54 USD 2023) per patient. - This is an ICER of $83 ($95.57 USD 2023) per QALY   PSA:   - At the lower end of the cost-effective threshold range for Pakistan, the probability that TXA is cost-effective is 98%. | TXA cost-effective compared to standard care in both settings at author's stated threshold of $314–$2416 per QALY  In USD 2023 this is $362 – $2,782 per QALY | Early treatment of PPH with TXA is highly cost-effective in Nigeria and Pakistan and is likely to be cost-effective in countries in sub-Saharan Africa and southern Asia with a similar baseline risk of death due to maternal haemorrhage. |
| **Bundle Interventions** | | | | | |
| Study | Country | Intervention/s & Comparator/s   (dose and route if specified) | Results | Dominance / Cost-effectiveness | Summary of study conclusions |
| No studies identified | | | | | |

Economic results are stated as they appear in the original publications and as a conversion to USD 2023 using an online tool developed by the Campbell and Cochrane Economics Methods Group (CCEMG) and the Evidence for Policy and Practice Information and Coordinating Centre (EPPI-Centre) [8]. Cost conversions were completed in December 2023 and may change slightly depending on final GDP figures.

Abbreviations: AMTSL: Active Management of the Third Stage of Labour. CI: Confidence Interval. DALYs: Disability-Adjusted Life Years. ESM-UBT: Every Second Matters for Mothers and Babies-Uterine Balloon Tamponade. GDP: Gross Domestic Product. ICER: Incremental Cost-Effectiveness Ratio. ICU: Intensive Care Unit. IHO: Inhaled Oxytocin. INR: Indian Rupee. IV: Intravenous. OWSA: One-Way Sensitivity Analysis. PPH: Postpartum Haemorrhage. PSA: Probabilistic Sensitivity Analysis. QALY: Quality-Adjusted Life Year. TXA: Tranexamic Acid. UBT: Uterine Balloon Tamponade. USD: United Stated Dollar. VHW: Village Health Worker. WTP: Willingness to pay.

## Table G: Results from studies in the Sub-Saharan Africa region

| **Preventative Interventions** | | | | | |
| --- | --- | --- | --- | --- | --- |
| Study | Country | Intervention/s & Comparator/s   (dose and route if specified) | Results | Dominance / Cost-effectiveness | Summary of study conclusions |
| Vlassoff et al., 2016 [49] | Senegal | 1. Oxytocin Uniject (10 IU IM)  2. Misoprostol (600μg PO)  3. Standard care | - ICER of using misoprostol compared to standard care was $38.96, USD 2013 ($46.70 USD 2023), per PPH averted. - ICER of using oxytocin compared to standard care was $119.15 ($142.83 USD 2023) per PPH case averted.   (Note: this model was based on a single RCT where 0% of women treated with misoprostol had PPH.) | Misoprostol dominant compared to oxytocin (more effective and lower cost).  Misoprostol compared to standard care incurred incremental costs, and no threshold was stated. | In settings where a significant proportion of births take place outside of health facilities, without skilled providers, misoprostol based PPH prevention could be cost-effective and improve maternal health. |
| Carvalho et al., 2020 [45] | Ethiopia | 1. Inhaled oxytocin (note: not yet a licensed product)  2. Standard of care in that country and setting (different uterotonics depending on the setting) | Ethiopia specific results Base Case:   - IHO introduction would avert 3000 PPHs annually and 30 maternal deaths at an incremental cost of $1,443,000 ($1,630,493.22 USD 2023). - $464 per PPH averted  (524.29 USD 2023) - $47,557 per maternal life saved ($53,736.22 USD 2023).   OWSA:   - IHO was not cost-effective in any of the OWSA scenarios. | Ethiopia specific assessment:  Inhaled oxytocin not cost-effective at author's stated thresholds. | In settings like Bangladesh, where there is limited access to oxytocin, Inhaled Oxytocin could be a cost-saving intervention, as health impacts are accompanied by a substantial reduction in spending on PPH treatment. In the Ethiopian context, the product may not be considered a cost-effective intervention until the product is fully integrated into the health system. |
| Lubinga et al., 2015 [50] | Uganda | 1. Misoprostol (600 μg PO distributed to women in their antenatal visit or as part of a safe delivery kit).  2. Oxytocin (10 IU IM but limited to only those they deliver in a facility). | Base case:   - The intervention had an ICER of $181 USD 2012 ($220.77 USD 2023) per DALY averted from a government perspective, and $64 ($78.06 USD 2023) per DALY averted from a modified societal perspective.   PSA:   - The ICER ranged from $81 to $441 ($98.80 to $537.91 USD 2023) per DALY averted from the government and $-84 to $260 ($-102.46 to 317.13 USD 2023) per DALY averted from the societal perspective. - 100% of the iterations were below the authors cost-effective threshold from both perspectives. | Misoprostol strategy cost-effective compared to oxytocin strategy at author's stated threshold of $1,641 (3 x GDP per capita).  This is $2001.59 in USD 2023. | Prenatal distribution of misoprostol could potentially save lives at modest incremental costs in this setting. |
| Prata et al., 2010 [51]  Note: Only the comparison of ANC vs ANC-miso interventions met inclusion criteria for this review. | 34 countries in Sub-Saharan Africa | 1. Antenatal and postpartum care as outlined in the "WHO Mother Baby Package".   2. Implementing the same as above, plus community distribution of misoprostol for home births. | Low infrastructure settings:   - Adding misoprostol to MBP antenatal care would cost an extra $4,900.42 USD 2007 ($6462.86 USD 2023) and avert an extra 23 maternal deaths per cohort of 500,000 (calculated from Table 1).   Medium infrastructure settings:   - Adding misoprostol to MBP antenatal care would cost an extra $5,568.64 ($7344.13 USD 2023) and avert an extra 23 maternal deaths per cohort of 500,000 (calculated from Table 2).   High infrastructure settings:   - Adding misoprostol to MBP antenatal care would cost an extra $5,647.34 ($7447.92 USD 2023) and avert an extra 15 maternal deaths per cohort of 500,000 (calculated from Table 3). | No threshold stated. | Authors conclude that family planning plus safe abortion services and antenatal care which includes the distribution of misoprostol for PPH prevention at home births are the two most cost-effective interventions. |
| Fullerton et al., 2006 [28] | Zambia | 1. AMTSL (uterotonic not specified)  2. EMTSL | Zambia specific results   - 67 maternal deaths averted and net cost-saving of over $145,000 ($208,613.23 USD 2023) per 100,000 births. - Results were robust to a wide range of OWSA scenarios. | AMTSL strategy dominated EMTSL strategy in both settings. | AMTSL is associated with a distinct financial benefit to health facilities in addition to clinical benefits. |
| **Diagnostic Interventions** | | | | | |
| Study | Country | Intervention/s & Comparator/s   (dose and route if specified) | Results | Dominance / Cost-effectiveness | Summary of study conclusions |
| No studies identified | | | | | |
| **Treatment Interventions** | | | | | |
| Study | Country | Intervention/s & Comparator/s   (dose and route if specified) | Results | Dominance / Cost-effectiveness | Summary of study conclusions |
| Bradley et al., 2007 [52] | Multiple countries in region | 1. Training of TBAs to recognize PPH and treat with misoprostol (1000µg PR).   2. TBA attends birth but refers patient to hospital if PPH occurs. | - Training TBAs and giving misoprostol if needed would prevent 1647 cases of severe PPH (810 - 2920 in sensitivity analysis). - This would save $115,336 USD 2005 ($160,922.36 USD 2023) on transport, hospital fees, IV therapy, and blood products. - The uncertainty range for the estimated savings was $13,991 to $1,563,593 ($19,521 – $2,181,600 USD 2023). | Training TBAs and utilizing misoprostol dominated alternative. | This intervention has the potential to save millions of dollars and improve maternal health in settings with limited health resources. |
| Li et al., 2018 [48] | Nigeria | 1. TXA plus routine care  2. Placebo plus routine care | Nigeria specific results  Base Case   - 0.18 QALYs gained for an additional cost of $37.12 USD 2016 ($42.74 USD 2023) per patient. - This is an ICER of $208 ($239.49 USD 2023) per QALY gained.   PSA:   - At the lower end of the threshold range for Nigeria, the probability that TXA is cost-effective is 93%. | TXA cost-effective compared to standard care at author's stated threshold of $446–$2880 per QALY.  In USD 2023 this is $514 – $3,316 per QALY. | Early treatment of PPH with TXA is highly cost-effective in Nigeria and Pakistan and is likely to be cost-effective in countries in sub-Saharan Africa and southern Asia with a similar baseline risk of death due to maternal haemorrhage. |
| Sutherland et al., 2013 [30] | Nigeria | 1. Adding NASG to standard management of women with severe hypovolemic shock (MAP<60mmHg) due to obstetric haemorrhage.   2. Adding NASG to standard management of women with any degree of shock due to obstetric haemorrhage.   3. Standard care with no NASG | Nigeria specific results   - Using the NASG for cases of severe shock resulted in decreased deaths, hysterectomies, and severe morbidity (2,063 DALYs averted) for an incremental cost of $6,460 ($8,198 USD 2023). - This is an ICER of $3.13 ($3.97 USD 2023) per DALY averted. - Using NASG on all cases of shock was less effective and more costly than this. | Nigeria specific assessment: Both applying NASG to women with any shock or severe shock was cost-effective compared to standard care, but applying to severe cases was most effective. | The NASG is either cost saving or highly cost-effective for women in severe hypovolemic shock when administered in a tertiary care setting. |
| Downing et al., 2015 [53] | Zimbabwe & Zambia | 1. Application of the NASG at the primary health care centre.  2. Delaying application of NASG until the patient arrives at the referral hospital. | - Early application group had 0.712 fewer DALYs than those in the later application group for an incremental cost of $15.51 international dollars 2010 ($19.68 USD 2023). - Early NASG application costs $21.78 ($27.64 USD 2023) per DALY averted compared to delayed application.   Sensitivity Analysis   - The ICER of applying the NASG early compared to later was sensitive to the unit cost of blood transfusions and ranged from $9.22-$87.85 ($11.70 – $111.49 USD 2023). | Early application cost-effective compared to late application in Zambia.  Early application not cost-effective in Zimbabwe. | The evidence from Zambia supports the early application of NASG for women with hypovolemic shock from obstetric haemorrhage in the community setting. The evidence from Zimbabwe was suggestive of this too, but not statistically significant. |
| Mvundura et al., 2017 [54] | Kenya | 1. ESM-UBT plus standard care  2. Standard care without UBT or uterine packing   3. Standard care with uterine packing available | - ESM-UBT could prevent 1,255 hospital transfers, 430 hysterectomies, and 44 maternal deaths compared to no standard care, no packing. - The ICER for the UBT device was $26-$40 USD 2015 ($30.26 – $46.56 USD 2023) per DALY averted based on UBT device cost of $5-$15. - If uterine packing is available, the ICER per DALY averted was $164-$199 ($190.90 – $231.64 USD 2023) depending on UBT cost.   OWSA:   - Results in both scenarios were robust to all parameter changes in the OWSA and remained cost-effective. | ESM-UBT was cost-effective compared to both alternatives at the authors stated threshold of $1,358 (1 x GDP per capita in 2014).  This is $1,581 in USD 2023. | ESM-UBT is a cost-effective way to reduce hospital transfers, surgeries, and maternal deaths caused by severe PPH. The results of this study could be used to guide the expansion of this intervention in Kenya and other similar settings. |
| Franke et al., 2024 [55] | Madagascar | 1. Referral and transport to secondary care facility  2. No referral (primary care only) | - 46 women used the referral and transport system due to PPH - The intervention was estimated to incur $17.10, USD 2020 ($19.82 USD 2023) per life year saved for PPH patients | No threshold stated | The intervention was found to be very cost-effective and may assist with public health resource allocation in Madagascar. |
| **Bundle Interventions** | | | | | |
| Study | Country | Intervention/s & Comparator/s   (dose and route if specified) | Results | Dominance / Cost-effectiveness | Summary of study conclusions |
| Seim et al., 2023 [56] | Niger | 1. Standard care, plus: health worker education, misoprostol distribution for home deliveries, oxytocin for use in hospitals/health centres, semi-quantitative blood loss measurement, three step treatment for PPH with additional uterotonics, intrauterine condom tamponade, NASG and transfer for definitive management  2. Standard care in Niger prior to the above nationwide intervention being launched. | - Case fatality rate per 100 primary PPH cases decreased from 5.05% (95% CI 3.36–7.30) to 2.58% (2.18-3.03%) - The intervention was estimated to cost $27.73 - $37.94 USD 2013 ($33.24 – 45.48 USD 2023) per DALY averted. | No threshold specified | The low-cost intervention more than halved the mortality associated with primary PPH within 2 years of implementation |
| Williams et al., 2024[57] | Kenya, Nigeria, South Africa, & Tanzania | 1. Early PPH detection and treatment with: quantitative blood loss measurement, uterine massage, oxytocin, TXA, IV fluids, examination, and escalation to definitive management if needed, staff training and auditing, dedicated PPH-response trolley/case  2. Standard care in the respective hospitals and countries | - The intervention bundle would incur costs of $11.83 USD 2022 ($12.26 USD 2023) for each case of severe PPH averted or $113.91 ($118.10) for each DALY averted. | Cost-effective at threshold based on GDP per capita of $2,816 per DALY averted ($2,920 USD 2023).  Also, cost-effective at threshold based on opportunity cost of $1,690 per DALY ($1,752 USD 2023). | Early detection of PPH using a calibrated blood-loss collection drape and treatment with the WHO first-response bundle is cost-effective compared with usual care. |

Economic results are stated as they appear in the original publications and as a conversion to USD 2023 using an online tool developed by the Campbell and Cochrane Economics Methods Group (CCEMG) and the Evidence for Policy and Practice Information and Coordinating Centre (EPPI-Centre) [8]. Cost conversions were completed in December 2023 and may change slightly depending on final GDP figures.

Abbreviations: AMTSL: Active Management of the Third Stage of Labor. ANC: Antenatal care. CI: Confidence Interval. DALYs: Disability-Adjusted Life Years. EMTSL: Expectant Management of the Third Stage of Labor. ESM-UBT: Every Second Matters for Mothers and Babies-Uterine Balloon Tamponade. GDP: Gross Domestic Product. ICER: Incremental Cost-Effectiveness Ratio. IHO: Inhaled Oxytocin. IM: Intramuscular. IU: International Unit. IV: Intravenous. MAP: Mean Arterial Pressure. MBP: Mother Baby Package. NASG: Non-pneumatic Anti-Shock Garment. OWSA: One-Way Sensitivity Analysis. PO: Per Oral. PPH: Postpartum Haemorrhage. PR: Per Rectum. PSA: Probabilistic Sensitivity Analysis. QALY: Quality-Adjusted Life Year. RCT: Randomized Controlled Trial. TBA: Traditional Birth Attendant. TXA: Tranexamic Acid. UBT: Uterine Balloon Tamponade. USD: United States Dollar. WHO: World Health Organization. Μg: Micrograms.

## Table H: Results from studies in the completed in the international setting

| **Preventative Interventions** | | | | | |
| --- | --- | --- | --- | --- | --- |
| Study | Country | Intervention/s & Comparator/s   (dose and route if specified) | Results | Dominance / Cost-effectiveness | Summary of study conclusions |
| Lang et al., 2015 [58] | “International setting” | Scenario 1:  1. Oxytocin (in hospitals) and misoprostol (in community).  2. Oxytocin (in hospitals and no treatment in the community)  Scenario 2:  1. Misoprostol in both hospital and community.  2. No uterotonics in either hospitals or community settings. | Scenario 1:   - The intervention would avert 22 cases of PPH, 2 cases of severe PPH, the requirement for 6 women to have additional uterotonics, and 4 women to have transfusions. - The intervention would result in an additional 130 women would experience shivering and 42 women fever. - This intervention would save $320, USD 2012, ($390.32 USD 2023) for the cohort of 1000 women.   Scenario 2:   - The intervention would avert 37 cases of PPH, 3 cases of severe PPH, the requirement for 10 women to have additional uterotonics, and 6 women to have transfusions. - The intervention would result in an additional 217 women experiencing shivering and an extra 70 women fever. - The intervention would save $533 ($650.12 USD 2023) per cohort of 1000 women. | Scenario 1: Oxytocin in hospitals and misoprostol in community dominates oxytocin in hospitals alone.   Scenario 2: Using misoprostol in both community and hospital dominates no uterotonic use. | Even though misoprostol is not the optimum choice in the prevention of PPH, it could be an effective and cost-saving choice where oxytocin is not or cannot be used for a variety of reasons. |

Economic results are stated as they appear in the original publications and as a conversion to USD 2023 using an online tool developed by the Campbell and Cochrane Economics Methods Group (CCEMG) and the Evidence for Policy and Practice Information and Coordinating Centre (EPPI-Centre) [8]. Cost conversions were completed in December 2023 and may change slightly depending on final GDP figures.

Abbreviations: PPH: Postpartum Haemorrhage. USD: United States Dollar.

# **References**

1. Wohling J, Edge N, Pena‐Leal D, Wang R, Mol BW, Dekker G. Clinical and financial evaluation of carbetocin as postpartum haemorrhage prophylaxis at caesarean section: A retrospective cohort study. Aust N Z J Obstet Gynaecol. 2019;59(4):501–7. doi: 10.1111/ajo.12907.

2. You JH, Leung T-y. Cost-effectiveness analysis of carbetocin for prevention of postpartum hemorrhage in a low-burden high-resource city of China. PLoS One. 2022;17(12):e0279130. doi: 10.1371/journal.pone.0279130.

3. Hong L, Chen A, Chen J, Li X, Zhuang W, Shen Y, et al. The clinical evaluation of IIA balloon occlusion in caesarean delivery for patients with PAS: a retrospective study. BMC Pregnancy Childbirth. 2022;22(1):103. doi: 10.1186/s12884-022-04434-3.

4. Xue L, Zhang J, Shen H, Hou Y, Ai L, Cui X. The application of rapid rehabilitation model of multidisciplinary cooperation in cesarean section and the evaluation of health economics. Zhonghua Yi Xue Za Zhi. 2019;99(42):3335–9. doi: 10.3760/cma.j.issn.0376-2491.2019.42.012.

5. Voon HY, Shafie AA, Bujang MA, Suharjono HN. Cost effectiveness analysis of carbetocin during cesarean section in a high volume maternity unit. J Obstet Gynaecol Res. 2018;44(1):109–16. doi: 10.1111/jog.13486.

6. Briones JR, Talungchit P, Thavorncharoensap M, Chaikledkaew U. Economic evaluation of carbetocin as prophylaxis for postpartum hemorrhage in the Philippines. BMC Health Serv Res. 2020;20:1–12. doi: 10.1186/s12913-020-05834-x.

7. Tsu VD, Levin C, Tran MP, Hoang MV, Luu HT. Cost-effectiveness analysis of active management of third-stage labour in Vietnam. Health Policy Plan. 2009;24(6):438–44. doi: 10.1093/heapol/czp020.

8. The Campbell and Cochrane Economics Methods Group, Evidence for Policy and Practice Information and Coordinating Centre. CCEMG - EPPI-Centre Cost Converter 2010 [accessed: 20/11/2023]. Available from: <https://eppi.ioe.ac.uk/costconversion/>.

9. Pickering K, Gallos ID, Williams H, Price MJ, Merriel A, Lissauer D, et al. Uterotonic drugs for the prevention of postpartum haemorrhage: a cost-effectiveness analysis. Pharmacoecon Open. 2019;3:163–76. doi: 10.1007/s41669-018-0108-x.

10. Gallos I, Williams H, Price M, Pickering K, Merriel A, Tobias A, et al. Uterotonic drugs to prevent postpartum haemorrhage: a network meta-analysis. Health Technol Assess. 2019;23(9). doi: 10.3310/hta23090.

11. Higgins L, Mechery J, Tomlinson A. Does carbetocin for prevention of postpartum haemorrhage at caesarean section provide clinical or financial benefit compared with oxytocin? J Obstet Gynaecol. 2011;31(8):732–9. doi: 10.3109/01443615.2011.595982.

12. Luni Y, Borakati A, Matah A, Skeats K, Eedarapalli P. A prospective cohort study evaluating the cost-effectiveness of carbetocin for prevention of postpartum haemorrhage in caesarean sections. J Obstet Gynaecol Can. 2017;37(5):601–4. doi: 10.1080/01443615.2017.1284188.

13. Van Der Nelson HA, Draycott T, Siassakos D, Yau CW, Hatswell AJ. Carbetocin versus oxytocin for prevention of post-partum haemorrhage at caesarean section in the United Kingdom: an economic impact analysis. Eur J Obstet Gynecol Reprod Biol. 2017;210:286-91. doi: 10.1016/j.ejogrb.2017.01.004.

14. Matthijsse S, Andersson FL, Gargano M, Yip Sonderegger YL. Cost-effectiveness analysis of carbetocin versus oxytocin for the prevention of postpartum hemorrhage following vaginal birth in the United Kingdom. J Med Econ. 2022;25(1):129–37. doi: 10.1080/13696998.2022.2027669.

15. Denison FC, Carruthers KF, Hudson J, McPherson G, Chua GN, Peace M, et al. Nitroglycerin for treatment of retained placenta: A randomised, placebo-controlled, multicentre, double-blind trial in the UK. PLoS Med. 2019;16(12):e1003001. doi: 10.1371/journal.pmed.1003001.

16. Denison FC, Carruthers KF, Hudson J, McPherson G, Scotland G, Brook-Smith S, et al. Glyceryl trinitrate to reduce the need for manual removal of retained placenta following vaginal delivery: the GOT-IT RCT. Health Technol Assess. 2019;23(70):1–72. doi: 10.3310/hta23700.

17. Durand‐Zaleski I, Deneux‐Tharaux C, Seco A, Malki M, Frenkiel J, Sentilhes L, et al. An economic evaluation of tranexamic acid to prevent postpartum haemorrhage in women with vaginal delivery: the randomised controlled TRAAP trial. BJOG. 2021;128(1):114–20. doi: 10.1111/1471-0528.16456.

18. Sentilhes L, Bénard A, Madar H, Froeliger A, Petit S, Deneux-Tharaux C. Tranexamic acid for reduction of blood loss after Caesarean delivery: a cost-effectiveness analysis of the TRAAP2 trial. Br J Anaesth. 2023;131(5):893-900. doi: 10.1016/j.bja.2023.07.028.

19. Niola R, Giurazza F, Torbica A, Schena E, Silvestre M, Maglione F. Predelivery uterine arteries embolization in patients with placental implant anomalies: a cost-effective procedure. Radiol Med. 2017;122:77–9. doi: 10.1007/s11547-016-0690-x.

20. Edwards RT, Ezeofor V, Bryning L, Anthony BF, Charles JM, Weeks A. Prevention of postpartum haemorrhage: Economic evaluation of the novel butterfly device in a UK setting. Eur J Obstet Gynecol Reprod Biol. 2023;283:149–57. doi: 10.1016/j.ejogrb.2023.02.020.

21. Prick B, Duvekot J, Van Der Moer P, van Gemund N, Van Der Salm P, Jansen A, et al. Cost‐effectiveness of red blood cell transfusion vs. non‐intervention in women with acute anaemia after postpartum haemorrhage. Vox Sang. 2014;107(4):381–8. doi: 10.1111/vox.12181.

22. Ries J-J, Jeker L, Neuhaus M, Vogt DR, Girard T, Hoesli I. Implementation of the D-A-CH postpartum haemorrhage algorithm after severe postpartum bleeding accelerates clinical management: A retrospective case series. Eur J Obstet Gynecol Reprod Biol. 2020;247:225–31. doi: 10.1016/j.ejogrb.2020.01.001.

23. Dale M, Bell SF, O’Connell S, Scarr C, James K, John M, et al. What is the economic cost of providing an all Wales postpartum haemorrhage quality improvement initiative (OBS Cymru)? A cost-consequences comparison with standard care. Pharmacoecon Open. 2022;6(6):847–57. doi: 10.1007/s41669-022-00362-2

24. Gil-Rojas Y, Lasalvia P, Hernández F, Castañeda-Cardona C, Rosselli D. Cost-effectiveness of Carbetocin versus Oxytocin for Prevention of Postpartum Hemorrhage Resulting from Uterine Atony in Women at high-risk for bleeding in Colombia. Rev Bras Ginecol Obstet. 2018;40:242–50. doi: 10.1055/s-0038-1655747.

25. Henríquez-Trujillo AR, Lucio-Romero RA, Bermúdez-Gallegos K. Analysis of the cost–effectiveness of carbetocin for the prevention of hemorrhage following cesarean delivery in Ecuador. J Comp Eff Res. 2017;6(6):529–36. doi: 10.2217/cer-2017-0004.

26. Caceda SI, Ramos RR, Saborido CM. Pharmacoeconomic study comparing carbetocin with oxytocin for the prevention of hemorrhage following cesarean delivery in Lima, Peru. J Comp Eff Res. 2018;7(1):49-55. doi: 10.2217/cer-2017-0012.

27. Jose Diaz J, Jaramillo M. Evaluating interventions to reduce maternal mortality: evidence from Peru's PARSalud programme. J Dev Effect. 2009;1(4):387–412. doi: 10.1080/19439340903380872.

28. Fullerton JT, Frick KD, Fogarty LA, Fishel JD, Vivio DM. Active management of third stage of labour saves facility costs in Guatemala and Zambia. J Health Popul Nutr. 2006;24(4):540.

29. Pichon-Riviere A, Glujovsky D, Garay OU, Augustovski F, Ciapponi A, Serpa M, et al. Oxytocin in uniject disposable auto-disable injection system versus standard use for the prevention of postpartum hemorrhage in latin America and the Caribbean: a cost-effectiveness analysis. PLoS One. 2015;10(6):e0129044. doi: 10.1371/journal.pone.0129044.

30. Sutherland T, Downing J, Miller S, Bishai DM, Butrick E, Fathalla MM, et al. Use of the non-pneumatic anti-shock garment (NASG) for life-threatening obstetric hemorrhage: a cost-effectiveness analysis in Egypt and Nigeria. PloS One. 2013;8(4):e62282. doi: 10.1371/journal.pone.0062282.

31. Barrett J, Ko S, Jeffery W. Cost implications of using carbetocin injection to prevent postpartum hemorrhage in a Canadian urban Hospital. J Obstet Gynaecol Can. 2022;44(3):272–8. doi: 10.1016/j.jogc.2021.09.022.

32. Dazelle WD, Ebner MK, Kazma J, Potarazu SN, Ahmadzia HK. Tranexamic acid for the prevention of postpartum hemorrhage: a cost-effectiveness analysis. J Thromb Thrombolysis. 2023:1–9. doi: 10.1007/s11239-023-02814-w.

33. Katz D, Wang R, O'Neil L, Gerber C, Lankford A, Rogers T, et al. The association between the introduction of quantitative assessment of postpartum blood loss and institutional changes in clinical practice: an observational study. Int J Obstet Anesth. 2020;42:4–10. doi: 10.1016/j.ijoa.2019.05.006.

34. Howard DC, Jones AE, Skeith A, Lai J, D'Souza R, Caughey AB. Tranexamic acid for the treatment of postpartum hemorrhage: a cost-effectiveness analysis. Am J Obstet Gynecol MFM. 2022;4(3):100588. doi: 10.1016/j.ajogmf.2022.100588.

35. Sudhof LS, Shainker SA, Einerson BD. Tranexamic acid in the routine treatment of postpartum hemorrhage in the United States: a cost-effectiveness analysis. Am J Obstet Gynecol. 2019;221(3):275. e1–. e12. doi: 10.1016/j.ajog.2019.06.030.

36. Einerson BD, Stehlikova Z, Nelson RE, Bellows BK, Kawamoto K, Clark EA. Transfusion preparedness strategies for obstetric hemorrhage: a cost-effectiveness analysis. Obstet Gynecol. 2017;130(6):1347–55. doi: 10.1097/AOG.0000000000002359.

37. Snegovskikh D, Souza D, Walton Z, Dai F, Rachler R, Garay A, et al. Point-of-care viscoelastic testing improves the outcome of pregnancies complicated by severe postpartum hemorrhage. J Clin Anesth. 2018;44:50–6. doi: 10.1016/j.jclinane.2017.10.003.

38. Lim G, Melnyk V, Facco FL, Waters JH, Smith KJ. Cost-effectiveness analysis of intraoperative cell salvage for obstetric hemorrhage. Anesthesiology. 2018;128(2):328–37. doi: 10.1097/ALN.0000000000001981.

39. Wiesehan EC, Keesara SR, Krissberg JR, Main EK, Goldhaber-Fiebert JD. State perinatal quality collaborative for reducing severe maternal morbidity from hemorrhage: a cost-effectiveness analysis. Obstet Gynecol. 2023;141(2):387–94. doi: 10.1097/AOG.0000000000005060.

40. Cook JR, Saxena K, Taylor C, Jacobs JL. Cost-effectiveness and budget impact of heat-stable carbetocin compared to oxytocin and misoprostol for the prevention of postpartum hemorrhage (PPH) in women giving birth in India. BMC Health Serv Res. 2023;23(1):267. doi: 10.1186/s12913-023-09263-4.

41. Sutherland T, Bishai DM. Cost-effectiveness of misoprostol and prenatal iron supplementation as maternal mortality interventions in home births in rural India. Int J Gynaecol Obstet. 2009;104(3):189–93. doi: 10.1016/j.ijgo.2008.10.011.

42. Sutherland T, Meyer C, Bishai DM, Geller S, Miller S. Community-based distribution of misoprostol for treatment or prevention of postpartum hemorrhage: cost-effectiveness, mortality, and morbidity reduction analysis. Int J Gynaecol Obstet. 2010;108(3):289–94. doi: 10.1016/j.ijgo.2009.11.007.

43. Goldie SJ, Sweet S, Carvalho N, Natchu UCM, Hu D. Alternative strategies to reduce maternal mortality in India: a cost-effectiveness analysis. PLoS Med. 2010;7(4):e1000264. doi: 10.1371/journal.pmed.1000264.

44. Sharma JC, Kollabathula P, Jindal S, Anupma A, Sarkar A, Jaggarwal S, et al. Application of a Negative Intrauterine Pressure Suction Device for Prophylactic Management of Atonic Postpartum Hemorrhage: A Quality Improvement Study. Cureus. 2023;15(7):e42631. doi: 10.7759/cureus.42631.

45. Carvalho N, Hoque ME, Oliver VL, Byrne A, Kermode M, Lambert P, et al. Cost-effectiveness of inhaled oxytocin for prevention of postpartum haemorrhage: a modelling study applied to two high burden settings. BMC Med. 2020;18(1):1–18. doi: 10.1186/s12916-020-01658-y.

46. Joshi BN, Shetty SS, Moray KV, Chaurasia H, Sachin O. Cost-effectiveness and budget impact of adding tranexamic acid for management of post-partum hemorrhage in the Indian public health system. BMC Pregnancy Childbirth. 2023;23(1):9. doi: 10.1186/s12884-022-05308-4.

47. Joshi BN, Shetty SS, Moray KV, Sachin O, Chaurasia H. Cost-effectiveness of uterine balloon tamponade devices in managing atonic post-partum hemorrhage at public health facilities in India. PLoS One. 2021;16(8):e0256271. doi: 10.1371/journal.pone.0256271.

48. Li B, Miners A, Shakur H, Roberts I. Tranexamic acid for treatment of women with post-partum haemorrhage in Nigeria and Pakistan: a cost-effectiveness analysis of data from the WOMAN trial. Lancet Glob Health. 2018;6(2):e222–e8. doi: 10.1016/S2214-109X(17)30467-9.

49. Vlassoff M, Diallo A, Philbin J, Kost K, Bankole A. Cost-effectiveness of two interventions for the prevention of postpartum hemorrhage in Senegal. Int J Gynaecol Obstet. 2016;133(3):307–11. doi: 10.1016/j.ijgo.2015.10.015.

50. Lubinga SJ, Atukunda EC, Wasswa-Ssalongo G, Babigumira JB. Potential cost-effectiveness of prenatal distribution of misoprostol for prevention of postpartum hemorrhage in Uganda. PLoS One. 2015;10(11):e0142550. doi: 10.1371/journal.pone.0142550.

51. Prata N, Sreenivas A, Greig F, Walsh J, Potts M. Setting priorities for safe motherhood interventions in resource-scarce settings. Health Policy. 2010;94(1):1–13. doi: 10.1016/j.healthpol.2009.08.012.

52. Bradley SE, Prata N, Young-Lin N, Bishai D. Cost-effectiveness of misoprostol to control postpartum hemorrhage in low-resource settings. Int J Gynaecol Obstet. 2007;97(1):52–6. doi: 10.1016/j.ijgo.2006.12.005.

53. Downing J, El Ayadi A, Miller S, Butrick E, Mkumba G, Magwali T, et al. Cost-effectiveness of the non-pneumatic anti-shock garment (NASG): evidence from a cluster randomized controlled trial in Zambia and Zimbabwe. BMC Health Serv Res. 2015;15(1):1–10. doi: 10.1186/s12913-015-0694-6.

54. Mvundura M, Kokonya D, Abu‐Haydar E, Okoth E, Herrick T, Mukabi J, et al. Cost‐effectiveness of condom uterine balloon tamponade to control severe postpartum hemorrhage in Kenya. Int J Gynaecol Obstet. 2017;137(2):185–91. doi: 10.1002/ijgo.12125.

55. Franke MA, Nordmann K, Frühauf A, Ranaivoson RM, Rebaliha M, Rapanjato Z, et al. Inter-facility transfers for emergency obstetrical and neonatal care in rural Madagascar: a cost-effectiveness analysis. BMJ Open. 2024;14(4):e081482. doi: 10.1136/bmjopen-2023-081482.

56. Seim AR, Alassoum Z, Souley I, Bronzan R, Mounkaila A, Ahmed LA. The effects of a peripartum strategy to prevent and treat primary postpartum haemorrhage at health facilities in Niger: a longitudinal, 72-month study. Lancet Glob Health. 2023;11(2):e287–e95. doi: 10.1016/S2214-109X(22)00518-6.

57. Williams EV, Goranitis I, Oppong R, Perry SJ, Devall AJ, Martin JT, et al. A cost-effectiveness analysis of early detection and bundled treatment of postpartum hemorrhage alongside the E-MOTIVE trial. Nat Med. 2024. doi: 10.1038/s41591-024-03069-5.

58. Lang DL, Zhao F-L, Robertson J. Prevention of postpartum haemorrhage: cost consequences analysis of misoprostol in low-resource settings. BMC Pregnancy Childbirth. 2015;15(1):1–9. doi: 10.1186/s12884-015-0749-z.
