# Supplementary material for: The cost-effectiveness of preventing, diagnosing, and treating postpartum haemorrhage: A systematic review of economic evaluations
Source: PLoS Med. 2024 Sep 13;21(9):e1004461. doi: 10.1371/journal.pmed.1004461 (PMC11433145; doi:10.1371/journal.pmed.1004461)
Supplement: S11 Appendix — (DOCX) [file pmed.1004461.s011.docx]

# **S11 Appendix: Summary of economic evaluations of oxytocin for PPH prevention**

Oxytocin appeared in many economic evaluations identified in this systemic review. These can be broadly divided into four groups (Table A).

Table A: Grouped economic evaluations of oxytocin for the prevention of postpartum haemorrhage (PPH)

| **Group** | **Studies** | **Location Discussed** |
| --- | --- | --- |
| Comprehensive assessment of all preventative uterotonics | - Pickering et al., 2019 [1] - Gallos et al., 2019 [2] | In manuscript and below |
| Assessment of carbetocin versus oxytocin | - Barrett et al., 2021 [3] - Cook et al., 2023 [4] - You et al., 2022 [5] - Gil-Rojas et al., 2018 [6] - Briones et al., 2020 [7] - Luni et al., 2017 [8] - van der Nelson et al., 2017 [9] - Wohling et al., 2019 [10] - Caceda et al., 2018 [11] - Henriquez-Trujillo et al., 2017 [12] - Voon et al., 2018 [13] - Higgins et al., 2011 [14] - Matthijsse et al., 2022 [15] | In manuscript |
| Assessment of oxytocin as the intervention compared to no uterotonics, non-injectable uterotonics, or other formulations of oxytocin | - Vlassoff et al., 2016 [16] - Diaz et al., 2009 [17] - Tsu et al., 2009 [18] - Pichon-Riviere et al., 2015 [19] - Carvalho et al., 2020 [20] | Below |
| Assessments of misoprostol, where the standard care involves the use of oxytocin in hospitals, but this is largely inaccessible to the population | - Lubinga et al., 2016 [21] - Prata et al., 2010 [22] - Lang et al., 2015 [23] | In manuscript |

Only a single Health Technology Assessment (HTA) [1,2] completed in the UK compared the comparative effectiveness and costs of using the full range of uterotonics available: Carbetocin, Ergometrine, Ergometrine plus Oxytocin, Misoprostol plus Oxytocin, Misoprostol, Oxytocin. For vaginal birth, oxytocin was the least costly strategy but only the fourth most effective agent for preventing PPH>500ml behind carbetocin, ergometrine plus oxytocin, and misoprostol plus oxytocin. For caesarean sections, oxytocin was the third most effective strategy for preventing PPH >500ml behind misoprostol plus oxytocin and carbetocin. Oxytocin was also more costly than these strategies and was therefore dominated.

Five studies were identified where oxytocin was the intervention being assessed [16-20]. One study compared the preventative use of oxytocin (Uniject), misoprostol, or standard care (without uterotonics) in Senegal, and concluded that utilising misoprostol would be cost-saving and the most effective option compared to the alternatives [16]. This model scored moderate on CHEC-E (10/19) and the effectiveness of misoprostol relative to oxytocin in preventing PPHs differed significantly to the latest estimates in systematic reviews [2,24].

Another study, assessed the implementation of oxytocin as the key component of active management of the third stage of labour (AMTSL) in Peru [17], but did not list a cost-effectiveness threshold, or a year of costing, making interpretation of the incremental cost-effectiveness ratio (ICER) difficult. In addition, the cost-effectiveness conclusions may have been impacted by bias related to treatment effects, as the relative effectiveness of oxytocin used was greater than observed estimates in the latest systematic literature [2,24]. The study also scored low on the CHEC-E tool.

Two studies considered the Uniject formulation against the use of oxytocin using ampoules, needles and syringes [18,19]. The first, conducted in Vietnam, considered both formulations of oxytocin against standard care, and concluded that either formulation of oxytocin for AMTSL would confer health benefits while incurring low incremental costs ($22.59 per case of PPH averted with oxytocin via ampoules, $31.19 per case averted with Uniject) but no cost-effectiveness threshold was stated [18]. The second study considered the two formulations of oxytocin head-to-head, and estimated the net benefit of switching from ampoules to Uniject in each Latin American and Caribbean country, assuming an increase in AMTSL coverage attributable to the ease of use of Uniject [19]. In this analysis they found switching to Uniject was either cost-saving or cost-effective in all 30 countries analysed. Both studies assessing oxytocin given via Uniject scored high on CHEC-E but some effectiveness estimates were found to be inconsistent with the latest estimates found in Cochrane reviews [2,24]. In addition, the assumption that switching to Uniject from ampoule and syringe will increase uterotonic coverage, which is central to one model [19], was based on a Delphi panel rather than an observed effect.

The final study in this group estimated the impact of introducing a heat-stable inhalable formulation of oxytocin (still under development) in Bangladesh and Ethiopia [20]. They reported that the novel product would be cost saving in Bangladesh but would likely exceed cost-effectiveness thresholds in Ethiopia. However, the effectiveness of inhaled oxytocin is yet to be established [25].

Altogether, the most methodologically sound cost-effectiveness evidence on the usage of oxytocin for the prevention of PPH appears to come from the HTA completed in the UK [1,2]. The five studies identified where oxytocin was the intervention (rather than the comparator/standard of care as in the identified carbetocin studies) were heterogenous and based on effectiveness data that is either no longer consistent with the latest estimates or has not yet been established yet in the case of inhaled oxytocin.

# **References**

1. Pickering K, Gallos ID, Williams H, Price MJ, Merriel A, Lissauer D, et al. Uterotonic drugs for the prevention of postpartum haemorrhage: a cost-effectiveness analysis. Pharmacoecon Open. 2019;3:163–76. doi: 10.1007/s41669-018-0108-x.

2. Gallos I, Williams H, Price M, Pickering K, Merriel A, Tobias A, et al. Uterotonic drugs to prevent postpartum haemorrhage: a network meta-analysis. Health Technol Assess. 2019;23(9). doi: 10.3310/hta23090.

3. Barrett J, Ko S, Jeffery W. Cost implications of using carbetocin injection to prevent postpartum hemorrhage in a Canadian urban Hospital. J Obstet Gynaecol Can. 2022;44(3):272–8. doi: 10.1016/j.jogc.2021.09.022.

4. Cook JR, Saxena K, Taylor C, Jacobs JL. Cost-effectiveness and budget impact of heat-stable carbetocin compared to oxytocin and misoprostol for the prevention of postpartum hemorrhage (PPH) in women giving birth in India. BMC Health Serv Res. 2023;23(1):267. doi: 10.1186/s12913-023-09263-4.

5. You JH, Leung T-y. Cost-effectiveness analysis of carbetocin for prevention of postpartum hemorrhage in a low-burden high-resource city of China. PLoS One. 2022;17(12):e0279130. doi: 10.1371/journal.pone.0279130.

6. Gil-Rojas Y, Lasalvia P, Hernández F, Castañeda-Cardona C, Rosselli D. Cost-effectiveness of Carbetocin versus Oxytocin for Prevention of Postpartum Hemorrhage Resulting from Uterine Atony in Women at high-risk for bleeding in Colombia. Rev Bras Ginecol Obstet. 2018;40:242–50. doi: 10.1055/s-0038-1655747.

7. Briones JR, Talungchit P, Thavorncharoensap M, Chaikledkaew U. Economic evaluation of carbetocin as prophylaxis for postpartum hemorrhage in the Philippines. BMC Health Serv Res. 2020;20:1–12. doi: 10.1186/s12913-020-05834-x.

8. Luni Y, Borakati A, Matah A, Skeats K, Eedarapalli P. A prospective cohort study evaluating the cost-effectiveness of carbetocin for prevention of postpartum haemorrhage in caesarean sections. J Obstet Gynaecol Can. 2017;37(5):601–4. doi: 10.1080/01443615.2017.1284188.

9. Van Der Nelson HA, Draycott T, Siassakos D, Yau CW, Hatswell AJ. Carbetocin versus oxytocin for prevention of post-partum haemorrhage at caesarean section in the United Kingdom: an economic impact analysis. Eur J Obstet Gynecol Reprod Biol. 2017;210:286-91. doi: 10.1016/j.ejogrb.2017.01.004.

10. Wohling J, Edge N, Pena‐Leal D, Wang R, Mol BW, Dekker G. Clinical and financial evaluation of carbetocin as postpartum haemorrhage prophylaxis at caesarean section: A retrospective cohort study. Aust N Z J Obstet Gynaecol. 2019;59(4):501–7. doi: 10.1111/ajo.12907.

11. Caceda SI, Ramos RR, Saborido CM. Pharmacoeconomic study comparing carbetocin with oxytocin for the prevention of hemorrhage following cesarean delivery in Lima, Peru. J Comp Eff Res. 2018;7(1):49-55. doi: 10.2217/cer-2017-0012.

12. Henríquez-Trujillo AR, Lucio-Romero RA, Bermúdez-Gallegos K. Analysis of the cost–effectiveness of carbetocin for the prevention of hemorrhage following cesarean delivery in Ecuador. J Comp Eff Res. 2017;6(6):529–36. doi: 10.2217/cer-2017-0004.

13. Voon HY, Shafie AA, Bujang MA, Suharjono HN. Cost effectiveness analysis of carbetocin during cesarean section in a high volume maternity unit. J Obstet Gynaecol Res. 2018;44(1):109–16. doi: 10.1111/jog.13486.

14. Higgins L, Mechery J, Tomlinson A. Does carbetocin for prevention of postpartum haemorrhage at caesarean section provide clinical or financial benefit compared with oxytocin? J Obstet Gynaecol. 2011;31(8):732–9. doi: 10.3109/01443615.2011.595982.

15. Matthijsse S, Andersson FL, Gargano M, Yip Sonderegger YL. Cost-effectiveness analysis of carbetocin versus oxytocin for the prevention of postpartum hemorrhage following vaginal birth in the United Kingdom. J Med Econ. 2022;25(1):129–37. doi: 10.1080/13696998.2022.2027669.

16. Vlassoff M, Diallo A, Philbin J, Kost K, Bankole A. Cost-effectiveness of two interventions for the prevention of postpartum hemorrhage in Senegal. Int J Gynaecol Obstet. 2016;133(3):307–11. doi: 10.1016/j.ijgo.2015.10.015.

17. Jose Diaz J, Jaramillo M. Evaluating interventions to reduce maternal mortality: evidence from Peru's PARSalud programme. J Dev Effect. 2009;1(4):387–412. doi: 10.1080/19439340903380872.

18. Tsu VD, Levin C, Tran MP, Hoang MV, Luu HT. Cost-effectiveness analysis of active management of third-stage labour in Vietnam. Health Policy Plan. 2009;24(6):438–44. doi: 10.1093/heapol/czp020.

19. Pichon-Riviere A, Glujovsky D, Garay OU, Augustovski F, Ciapponi A, Serpa M, et al. Oxytocin in uniject disposable auto-disable injection system versus standard use for the prevention of postpartum hemorrhage in latin America and the Caribbean: a cost-effectiveness analysis. PLoS One. 2015;10(6):e0129044. doi: 10.1371/journal.pone.0129044.

20. Carvalho N, Hoque ME, Oliver VL, Byrne A, Kermode M, Lambert P, et al. Cost-effectiveness of inhaled oxytocin for prevention of postpartum haemorrhage: a modelling study applied to two high burden settings. BMC Med. 2020;18(1):1–18. doi: 10.1186/s12916-020-01658-y.

21. Lubinga SJ, Atukunda EC, Wasswa-Ssalongo G, Babigumira JB. Potential cost-effectiveness of prenatal distribution of misoprostol for prevention of postpartum hemorrhage in Uganda. PLoS One. 2015;10(11):e0142550. doi: 10.1371/journal.pone.0142550.

22. Prata N, Sreenivas A, Greig F, Walsh J, Potts M. Setting priorities for safe motherhood interventions in resource-scarce settings. Health Policy. 2010;94(1):1–13. doi: 10.1016/j.healthpol.2009.08.012.

23. Lang DL, Zhao F-L, Robertson J. Prevention of postpartum haemorrhage: cost consequences analysis of misoprostol in low-resource settings. BMC Pregnancy Childbirth. 2015;15(1):1–9. doi: 10.1186/s12884-015-0749-z.

24. Gallos ID, Papadopoulou A, Man R, Athanasopoulos N, Tobias A, Price MJ, et al. Uterotonic agents for preventing postpartum haemorrhage: a network meta‐analysis. Cochrane Database Syst Rev. 2018;(12). doi: 10.1002/14651858.CD011689.pub3.

25. Gajewska-Knapik K, Kumar S, Sutton-Cole A, Palmer KR, Cahn A, Gibson RA, et al. Pharmacokinetics and safety of inhaled oxytocin compared with intramuscular oxytocin in women in the third stage of labour: A randomized open-label study. Br J Clin Pharmacol. 2023;89(12):3681–9. doi: 10.1111/bcp.15860.
