# Supplementary material for: The cost-effectiveness of preventing, diagnosing, and treating postpartum haemorrhage: A systematic review of economic evaluations
Source: PLoS Med. 2024 Sep 13;21(9):e1004461. doi: 10.1371/journal.pmed.1004461 (PMC11433145; doi:10.1371/journal.pmed.1004461)
Supplement: S12 Appendix — (DOCX) [file pmed.1004461.s012.docx]

# **S12 Appendix: Summary of economic evaluations of surgical interventions for postpartum haemorrhage prevention**

Three studies that assessed surgical interventions for preventing postpartum haemorrhage (PPH) were identified. Two studies assessed invasive procedures (internal iliac artery occlusion, and uterine artery occlusion) performed pre-caesarean section to minimise the risk of PPH in women with placental implant anomalies [1,2]. The third assessed the application of ERAS (enhanced recovery after surgery) principles to the peri-operative care for women undergoing caesarean section in China [3]. Internal iliac artery occlusion was reported as being neither effective nor cost-effective [1], while both uterine artery occlusion and ERAS were reported as reducing the rates of adverse health outcomes and simultaneously reducing costs [2,3]. While all three met inclusion criteria for this review, numerous key information in the economic analyses was omitted and each scored low on CHEC-E. The underlying effectiveness of prophylactic endovascular interventions remains uncertain and both The International Federation of Gynaecology and Obstetrics (FIGO) and the International Society for Abnormally Invasive Placenta (IS-AIP) state that larger prospective controlled trials are needed [4,5]. It is also unclear the extent to which ERAS reduces surgical blood loss in caesarean section [6-8]. The economic findings of these studies should be interpreted with care.

# **References**

1. Hong L, Chen A, Chen J, Li X, Zhuang W, Shen Y, et al. The clinical evaluation of IIA balloon occlusion in caesarean delivery for patients with PAS: a retrospective study. BMC Pregnancy Childbirth. 2022;22(1):103. doi: 10.1186/s12884-022-04434-3.

2. Niola R, Giurazza F, Torbica A, Schena E, Silvestre M, Maglione F. Predelivery uterine arteries embolization in patients with placental implant anomalies: a cost-effective procedure. Radiol Med. 2017;122:77–9. doi: 10.1007/s11547-016-0690-x.

3. Xue L, Zhang J, Shen H, Hou Y, Ai L, Cui X. The application of rapid rehabilitation model of multidisciplinary cooperation in cesarean section and the evaluation of health economics. Zhonghua Yi Xue Za Zhi. 2019;99(42):3335–9. doi: 10.3760/cma.j.issn.0376-2491.2019.42.012.

4. Allen L, Jauniaux E, Hobson S, Papillon-Smith J, Belfort MA, Diagnosis ftFPA, et al. FIGO consensus guidelines on placenta accreta spectrum disorders: Nonconservative surgical management. Int J Gynaecol Obstet. 2018;140(3):281–90. doi: 10.1002/ijgo.12409.

5. Collins SL, Alemdar B, van Beekhuizen HJ, Bertholdt C, Braun T, Calda P, et al. Evidence-based guidelines for the management of abnormally invasive placenta: recommendations from the International Society for Abnormally Invasive Placenta. Am J Obstet Gynecol. 2019;220(6):511–26. doi: 10.1016/j.ajog.2019.02.054.

6. Caughey AB, Wood SL, Macones GA, Wrench IJ, Huang J, Norman M, et al. Guidelines for intraoperative care in cesarean delivery: Enhanced Recovery After Surgery Society Recommendations (Part 2). Am J Obstet Gynecol. 2018;219(6):533–44. doi: 10.1016/j.ajog.2018.08.006.

7. Macones GA, Caughey AB, Wood SL, Wrench IJ, Huang J, Norman M, et al. Guidelines for postoperative care in cesarean delivery: Enhanced Recovery After Surgery (ERAS) Society recommendations (part 3). Am J Obstet Gynecol. 2019;221(3):247.e1–.e9. doi: 10.1016/j.ajog.2019.04.012.

8. Wilson RD, Caughey AB, Wood SL, Macones GA, Wrench IJ, Huang J, et al. Guidelines for Antenatal and Preoperative care in Cesarean Delivery: Enhanced Recovery After Surgery Society Recommendations (Part 1). Am J Obstet Gynecol. 2018;219(6):523.e1–.e15. doi: 10.1016/j.ajog.2018.09.015.
