# Supplementary material for: The cost-effectiveness of preventing, diagnosing, and treating postpartum haemorrhage: A systematic review of economic evaluations
Source: PLoS Med. 2024 Sep 13;21(9):e1004461. doi: 10.1371/journal.pmed.1004461 (PMC11433145; doi:10.1371/journal.pmed.1004461)
Supplement: Attachment — Submitted filename: Response Letter.docx [file pmed.1004461.s013.docx]

Dr Louise Gaynor-Brook
Senior Editor
PLOS Medicine

Dear Dr Gaynor-Brook,

Thank you for taking the time to review our systematic review, and to provide detailed comments and suggestions. Please see below the actions the team have taken to address the specific points raised by the review and editorial teams. We look forward to continuing to work on this submission with your team to create the best possible version of it for the reader.

Kind regards,

Josh Ginnane

**All line numbers in the response column refer to line numbers when viewing in “All Markup” mode.**

| **Reviewer 1** | |
| --- | --- |
| **Comments/Requests** | **Responses** |
| **Comment / Request: a**  *The manuscript mentions that for each study, effectiveness data were extracted and compared with up-to-date estimates from corresponding WHO recommendations and systematic reviews. It would be beneficial for the reader to understand the impact of these up-to-date effectiveness estimates on the overall cost-effectiveness results. Could you please provide examples or a detailed discussion on how significant changes in effectiveness estimates have influenced the economic outcomes in specific studies?* | **Response: a**  We have added another paragraph in the Discussion to address this point (Lines 436 - 450) |
| **Comment / Request: b**  *The paper states that a transferability assessment was not completed due to the absence of an ideal setting for comparison. However, understanding the applicability of these economic evaluations in different healthcare contexts is crucial. Could the authors discuss the potential limitations this poses on the generalizability of the study findings?* | **Response: b**  We have added another limitation to address this. Please see lines 502 – 505. |
| **Comment / Request: c**  *Regarding the mapping of studies to WHO recommendations, the methodology used for this alignment is not clearly outlined. Clarifying how studies were associated with specific WHO recommendations would enhance the transparency of the process.*  *Additionally, it would be insightful if the authors could discuss whether the findings suggest any modifications or reaffirmations to existing WHO recommendations based on the newly reviewed economic data.* | **Response: c**  In response to the first half of this comment, we have altered the tables in Appendix 9. These previously showed each WHO recommendation, alongside whether this recommendation had cost-effectiveness evidence in the form of a systematic review of economic evaluations, and whether we found any evidence in our review.  The middle column covering the content of previous systematic reviews has been removed. We have expanded the details on which studies we identified, their settings, and the study aims that link to the WHO recommendations. Please see pages 97 to 104 in the Supps document.  To make the methods clearer in the manuscript we added lines 207 - 209.  In response to the second half of request c, we agree that it would be insightful to discuss whether the findings indicate that any WHO recommendations require modification or reassessment. However, to do this in a systematic approach we would need to review the Evidence to Decision (EtD) frameworks of each WHO recommendation to see whether our findings or assessment of the economic evidence confirms or undermines the previous evidence used when making the recommendation. We believe this body of work would not be practical at this time for a number of reasons:   - Firstly, there are 29 recommendations made through 8 different WHO publications. Reviewing the EtD for each of these would be beyond the scope of the current review. Our intention with adding this mapping (beyond the scope of a systematic review) was to provide an illustration of how there is limited / insufficient economic evidence even on high-priority PPH-related interventions. - Secondly, the resource/cost section of each EtD is only one of many supportive sections which provide evidence. WHO Guideline panels are expected to make decisions using evidence across multiple domains, including effectiveness, values and preferences, equity, acceptability, feasibility and resource use. It would be extremely difficult to predict whether changes to the resource/cost section alone would be enough to change any recommendations. - Thirdly, we are aware that the incorporation of the findings of this systematic review, along with multiple other sources of evidence, will be deliberated on by an expert panel in the near future. This is in the context of WHO’s work on the PPH roadmap, and consolidated PPH guidelines. We do not think it appropriate to pre-empt the outcomes of that deliberative process. |
| **Comment / Request: d**  *The manuscript could benefit from a more detailed exploration of uncertainties associated with the studies reviewed, particularly how these uncertainties impact the cost-effectiveness of interventions. Were there any specific resource use parameters within these studies that were identified as highly uncertain? How might these uncertainties affect the reliability of the cost-effectiveness results?* | **Response: d**  Thank you for this comment. With such a large group of diverse studies it was extremely difficult to succinctly summarize individual sources of uncertainty, especially as these varied considerably between studies and interventions. We tried to describe the impact of the following major sources of uncertainty in the tables and appendices:   - different study countries/settings (Table 1 & appendix 6) - Study perspective (appendix 6) - Effect estimates used (Appendix 8) - Soundness of study design, parameter choice, time horizon etc (CHEC-E assessments Table 1 and Appendix 7)   Regarding resource use parameters, as part of the CHEC-E assessment each study was assessed as to whether all appropriate costs are identified, measured and valued appropriately (Q8-10). However, we did not go to the same extent of interrogating each resource use parameter as we did for effectiveness parameters – this would be a huge body of work far beyond the scope of the review. To address this and ensure that readers have full transparency that some sources of uncertainty were investigated more extensively than others, we have made an additional limitation. Please see lines 505 - 511. |
| **Comment / Request: e**  *For the economic evaluations that utilized modelling approaches, the manuscript does not provide sufficient details on the validity and reliability of these models. Could the authors include a section discussing the assumptions made within these models, the sources of data used for parameter estimates, and any validation processes undertaken?* | **Response: e**  Thank you for the comment. We agree in principle on the value of discussing modelling approaches and assumptions used. However, as there were 38 modelling studies included in this review, and only 2 studies were based off the same model, this results in 37 unique models each with their own assumptions and individual approaches to validation. As it would be impractical to discuss each of these individually, we elected to use the CHEC-E score as an assessment on whether each study adequately reported validation methods, choice of assumptions, and model structure (Appendix 7).  To ensure readers are clear that we have interrogated some sources of uncertainty to a greater extent than others, we have added lines - 505 - 511 in the limitations section. |
| **Comment / Request: f**  *While the manuscript reviews various economic evaluations, it does not provide a detailed critique of the methodologies used across these studies. A deeper examination of the strengths and limitations of different economic evaluation methods would be beneficial.* | **Response: f**  To address this we have added more details about the type and overall quality of economic evaluations performed – please see lines 199-203.  We also brought some of the details from Table 1 and Appendix 6 into the Results section to add more transparency to each result being presented.   - Added the study setting to the description prefacing results. Examples – Lines 230, 304, 315 - Added, where possible, the description of study design when prefacing results. Examples – Lines 238, 248, 270, 296, 297   Picking discrete methods that are more robust or appropriate in this review is very difficult as there are so many different interventions, settings and methods covered within included studies. Some methods might be entirely appropriate for one study but inappropriate for another. For example, taking a tight time horizon and a narrower perspective might be appropriate for a uterotonic evaluation in a high-income setting where death and disability to PPH is comparatively rarer and the focus is on the short-term burden on the health system – the cost of additional uterotonics, IV fluids, or blood products. This same approach might not be appropriate for a setting where there are considerable long-term impacts from PPH that a short time horizon and narrow perspective won’t capture.  We have chosen to add this in as a limitation as a more detailed critique of included studies may have been possible if the review had a narrower focus. Please see lines 514-520. |

| **Reviewer 2** | |
| --- | --- |
| **Comments/Requests** | **Responses** |
| **Comment / Request: Introduction 1**  *While the health outcomes associated with PPH are made clear, it would be useful to provide an indication of the associated economic burden. Incorporating this would provide readers with a more comprehensive understanding of the overall impact of PPH.* | **Response: Introduction 1**  We were only able to partially address this comment – please see lines 93-95.  We have searched the literature and have not identified any data pertaining to the broader societal / economic impacts of PPH beyond the financial/resource impact on health systems. |
| **Comment / Request: Introduction 2**  *I feel that further justification is needed to demonstrate the relevance of reviewing evidence from high-income countries, particularly when the authors highlight that deaths from PPH have largely been eliminated in HICs, that PPH disproportionately affects patients in LMICs, and that results indicate that cost-effectiveness of such interventions can be highly contextual.* | **Response: Introduction 2**  Our aim for this review was *“to identify, assess, and synthesise all available evidence from economic evaluations of any postpartum intervention for PPH prevention, diagnosis, or treatment”.*  In keeping with this aim we have summarized evidence from all of the studies that met our eligibility criteria. We completely agree that economic evaluation results can be highly contextual (and have emphasised this point throughout the manuscript). We also believe that when interventions demonstrate good effectiveness and cost-effectiveness in high-income settings, it is considerably easier to advocate for assessing whether these could (or should) be implemented and assessed in LMICs.  To ensure transparency, wherever we report a result in the manuscript we have also reported the setting from which that evidence was derived. In addition, in the Implications for policy and practice section, we have only advocated broadly for interventions with good effectiveness, and cost-effectiveness data across a range of settings, including LMICs. |
| **Comment / Request: Introduction 3**  *Cost-effectiveness evidence relating to many interventions for prevention, detection and treatment of PPH is summarised in the paper. Including an introductory section with more detail on these interventions could be useful.* | **Response: Introduction 3**  We have added some more detail to the introduction – please see lines 102 – 116. |
| **Comment / Request: Methods 1**  *More clarity is needed on the eligibility criteria. The authors state that full economic evaluations were included, whilst partial economic evaluations were excluded. However, a number of cost-consequence analyses (which do not permit full incremental analyses) are included. A longitudinal study is also included, which is not a full economic evaluation.* | **Response: Methods 1**  We have added a sentence to clarify our meaning of full economic evaluation. Please see lines 140 – 142.  CCAs (even though they don’t calculate an ICER) and observational studies can be considered full economic evaluations if they present both health outcomes, and economic outcomes against some comparison.  <https://www.ncbi.nlm.nih.gov/books/NBK481593/>  In the case of the longitudinal study from Niger included in our review, the cost and health outcomes were compared from the intervention period to a pre-study period. |
| **Comment / Request: Methods 2**  *More information on the method used to convert costs to 2023 USD would be useful. Was this using exchange rates or a PPP adjustment approach?* | **Response: Methods 2**  Thank you for the comment, the handy tool we used uses both a GDP deflator for the cost year, and then a PPP currency conversion. We have added a sentence to make this clearer for the reader – see lines 173 - 175 |
| **Comment / Request: Results 1**  *It would be useful to summarise more characteristics of the included studies, such as setting (community, hospital), economic evaluation type (model vs trial-based analysis) and perspective (societal, healthcare payer).* | **Response: Results 1**  The study characteristics are summarized in a condensed form in Table 1, and in a more detailed form in Appendix 6.  We added lines 196 -200 to bring some of this into the text. |
| **Comment / Request: Results 2**    *Many studies assessed the cost-effectiveness of prophylactic uterotonics, and consequently that sub-section of the results is quite long. Could it be subdivided further to make it a little easier to digest?* | **Response: Results 2**  We added subheadings to this section to break this section up. |
| **Comment / Request: Results 3**  *It was not clear why the studies assessing oxytocin to no uterotonic, non-injectable uterotonics or other formulations of oxytocin were not detailed in the main text whilst the single study on AMTSL was. Similarly, it was not overly clear why the studies on surgical methods for minimising PPH were not included in the main text. Was it due to their low quality?* | **Response: Results 3**  To respond to this comment, we have made changes to the oxytocin section to make the choice of summarizing some studies in the appendix clearer. Please see lines 262 to 268 for additional detail.  We have also added clarity to the surgical interventions section. Please see lines 310 – 313. |
| **Comment / Request: Results 4**  *At times I felt that more context is needed when summarising the cost-effectiveness findings. The authors acknowledge the considerable heterogeneity between studies in the discussion, but it doesn't really come across in the results section. Including a more critical/comprehensive overview of key study aspects in the study results could enhance understanding and facilitate a more nuanced interpretation of the findings. Though, I appreciate this can be tricky when so many studies are included!* | **Response:**  To try to add more context to each result, without making the results section prohibitively long or complicated we have:   - Added the study setting to the description prefacing results. Examples – Lines 230, 304, 315 - Added, where possible, the description of study design when prefacing results. Examples – Lines 238, 248, 270, 296, 297.   We considered adding the following context, but determined it would make the results section too long:   - Study perspective – even when trying to summarize a small number of studies such as for Carbetocin vs oxytocin in Caesarean Section, there were 4 different perspectives among just 7 studies. We removed these edits and chose to instead list it as a limitation. Please see lines 514 – 520. |
| **Comment / Request: Discussion 1**  *The summary of findings provided in the discussion is appropriate, however it could be more succinct.* | **Response: Discussion 1**  We have removed wordiness and streamlined the summary. Please see lines 423, 426, 427, 429 - 432. |
| **Comment / Request: Discussion 2**  *The conclusion presented in the discussion should also include the detail included in the conclusion of the abstract.* | **Response:**  We have made changes to the discussion conclusion to align both sections better. Please see lines 527 – 535. |

| **Reviewer 3** | |
| --- | --- |
| **Comments/Requests** | **Responses** |
| **Comment / Request: 1**  Thank you for allowing me to read this well conducted systematic review of economic evaluations of diagnostic and therapeutic interventions for post partum haemorrhage. The methods are fully described with the checklists used and selection criteria. The choice of including interventions in all countries makes the article relevant for all readers.  The drawback is that the presentation of the results is difficult, I suppose the authors have struggled to decide whether to present by country/region or, as they did, by intervention. I would suggest to add the regional aspect in the supplemental material (with a table, either a new one by region, or colour code the existing one to allow easy identification of the setting) and… | **Response:**  Thank you for your comments. To address this, we have created a new table in the appendices (pages 105 - 136) with the results of studies organized by region, country, and intervention. |
| **Comment / Request: 2**  …and in the Discussion because interventions that are highly feasible in a high-income setting (eg an iv line for TXA) might be more difficult in other settings. I would be more prudent about modeling to translate results across settings (p12), because this is limited by the availability of resources. | **Response:**  We have edited this sentence and added qualifying statements in the following sentences. Please see lines 487 – 490. |

| **Editorial Team** | |
| --- | --- |
| **Comments/Requests** | **Responses** |
| **Comment / Request:** 1  We would like to see a more thorough discussion of limitations in the Discussion section. | **Response: 1**  We have significantly expanded the limitations section using feedback from the reviewers. Please see lines 502 – 520. |
| **Comment / Request: 2**  In agreement with reviewer 3, we would also be interested in seeing results presented by region in the supplementary files, if this is feasible. | **Response: 2**  We have created appendix 10 so that users interested only in evidence from a particular region or country can quickly identify relevant economic evaluations and be provided with a summary of their results. Please see pages 105 -135 of the appendices. |

**Other changes made to bring manuscript in line with publishing guide:**

- Referencing style changed
- Appendices listed in text as S1 Appendix 1 and so forth.
- Tables have not been moved into place in the manuscript. They are so large it would make finding the following paragraph of text extremely difficult.
- Figures
  - Figure changed to “Fig” in text and file names.
  - Figure Captions re formatted and moved to appropriate place in manuscript.
  - Figure format checked using PACE
