# Supplementary material for: The cost-effectiveness of preventing, diagnosing, and treating postpartum haemorrhage: A systematic review of economic evaluations
Source: PLoS Med. 2024 Sep 13;21(9):e1004461. doi: 10.1371/journal.pmed.1004461 (PMC11433145; doi:10.1371/journal.pmed.1004461)
Supplement: Attachment — Submitted filename: Response Letter 2.docx [file pmed.1004461.s014.docx]

Dr Louise Gaynor-Brook
Senior Editor
PLOS Medicine

Dear Dr Gaynor-Brook,

Thank you for providing detailed comments to help guide our revision. Please see below how we have addressed each of the requests.

Kind regards,

Josh Ginnane

**All line numbers in the response column refer to line numbers when viewing in “All Markup” mode.**

| **Requests from the Editors** | |
| --- | --- |
| **Comments/Requests** | **Responses** |
| **General Requests**  We require that systematic reviews are updated to within roughly 6 months of the expected publication date. Please update your search to the present time. | Actioned. The updated search resulted in the identification of three new studies which have been incorporated into the manuscript and supplementary information.  Lines 329 – 333 Lines 346 – 353.  Lines 444 – 449. |
| **General Requests**  1) Throughout the paper, please adapt reference call-outs to the following style: "... every year [1,2]." (noting the absence of spaces within the square brackets, and placement before the full stop). | Actioned. |
| **General Requests**  2) Please include page numbers and line numbers in the manuscript file. Use continuous line numbers (do not restart the numbering on each page). | Added. |
| **General Requests**  3) To help us extend the reach of your research, please provide any X (formerly known as Twitter) handle(s) that would be appropriate to tag, including your own, your co-authors’, your institution, funder, or lab. Please enter in the submission form any handles you wish to be included when we post about this paper. | @BurnetInstitute  @josh_vogel |
| **ABSTRACT** 1) Please provide the beginning and end dates of your search. | Completed. Please see line 36. |
| **ABSTRACT**  2) In the last sentence of the Abstract Methods and Findings section, please describe 2-3 of the main limitations of the study's methodology. | Completed. Please see lines 49-52. |
| **ABSTRACT**  3) Please begin your Abstract Conclusions with "In this study, we..." or similar, to summarize the main findings from your study, without overstating your conclusions. Please emphasize what is new and address the implications of your study, being careful to avoid assertions of primacy. | Completed. Please see lines 56-60. |
| **ABSTRACT**  4) Please define WHO at first use. | Completed. Please see line 62.  Some journals repeat this first use rule in the main body of the manuscript. We have done this on line 111. If you only require it to be defined once in the whole manuscript, we can edit line 111. |
| **AUTHOR SUMMARY**  1) Thank you for providing an Author Summary. This should be formatted as 2-3 single sentence bullet points for each of the questions. Bullet points should be objective, brief, succinct, specific, accurate, and avoid technical language. | Actioned. Lines 68-106. |
| **AUTHOR SUMMARY**  2) In the final bullet point of ‘What Do These Findings Mean?’, please describe the main limitations of the study in non-technical  language. | Actioned. Line 105 |
| **INTRODUCTION**  If there has been a previous systematic review of the evidence related to your study (or you have conducted one), please refer to and reference that review and indicate whether it supports the need for your study. | Actioned. Please see lines 147 - 150 |
| **METHODS AND RESULTS**  1) Thank you for providing the completed PRISMA checklist. Please revise the checklist and use section and paragraph numbers, rather than page numbers which will likely no longer correspond to the appropriate sections after copy-editing. | Actioned. Please see S1 Appendix. |
| **METHODS AND RESULTS**  2) As stated above, please update your search to the present time and provide the beginning and end dates of your search. | Actioned. |
| **METHODS AND RESULTS**  3) Please report the total number of WHO recommendations (under "Characteristics of included studies"; for comparison purposes). | Added, thank you. Please see lines 234 |
| **METHODS AND RESULTS**  4) Figure 1: Thank you for providing the flow diagram. Please remove the reference from the figure description. It is sufficient to provide the reference in the appendix, as you have already done. There are two asterisks next to "Records excluded" in the figure without any definition in the description, please revise. We also suggest replacing the word "wrong" in the figure with "other" or similar to avoid any negative implications. | Actioned, thank you. Numbers have also been updated to reflect repeated search.  Also addressed the use of “wrong” in S5 Appendix. |
| **METHODS AND RESULTS**  5) Figure 2: Please define IV, PPH, WHO. Please mention the total number of studies identified in the systematic review in the figure description. Also, please add more details about what the top and bottom halves of the figure represent, e.g. “The top half shows the WHO-recommended PPH interventions at different stages postpartum, while the bottom half shows the interventions identified in the economic evaluation studies identified in this systematic review.”. | Updated. Please see lines 249 - 252. |
| **METHODS AND RESULTS**  6) Please reduce the number of subheadings within the Results section. For example, "Prophylactic uterotonics" should only be used as one subheading, with comprehensive paragraph introductions guiding the reader through the sections (e.g. “For evaluations considering carbetocin as the primary intervention, five studies used decision analytic models to compare carbetocin with oxytocin for both vaginal birth and caesarean section [31-35].”). | Apologies. This is how the original subheadings were. We changed these at the request of Reviewer 2 in the previous round of revisions. These have been simplified again. |
| **METHODS AND RESULTS**  7) Please include numerators and denominators when describing the number of studies identified for a particular intervention. For example, "Twenty-seven studies (27/54) focused on..." or "Five studies (5/27) used decision analytic models...". Please revise throughout the main text. | Actioned. Please see lines 193 – 217. |
| **METHODS AND RESULTS**  8) “($98·80-$537·91 per DALY averted)” - please use a consistent presentation format for numbers (i.e. the decimal point). We suggest writing ““($98.80-$537.91 per DALY averted)” - please revise throughout the main text. | Revised throughout. |
| **METHODS AND RESULTS**  9) Please ensure that abbreviations are defined the first time they are used, e.g. 'ICU/intensive care unit', or write the word in full if it is only used a few times. Please revise throughout the manuscript and try to reduce the number of abbreviations used. | Defined at first use added:  DALY – line 306.  UK - Line 261.  USD – 195.  Abbreviations removed from main manuscript:  GTN – see line 344.  AMTSL – see lines 323-325  SDGs Line 118.  HTA – lines 260, 298, 343  ICU – line 420. |
| **METHODS AND RESULTS**  10) Please carefully check the tables and make sure that all abbreviations are defined below the table, such as WHO, ICER, QALY, DALY, IU, IM, IV, S/, NHS, GTN, PO, EMTSL. | Reviewed all tables in manuscript and appendices. |
| **DISCUSSION**  1) Please remove all subheadings within your Discussion, e.g. Implications for policy and practice. | Actioned. |
| **DISCUSSION**  2) Please temper claims of primacy of results by stating, "to our knowledge" or something similar (e.g., “This is, to our knowledge, the first systematic review examining..). | Actioned. Please see line 471. |
| **DISCUSSION**  3) “We identified 54 studies - approximately half were conducted in high-income settings.” - if possible, please provide an exact number instead of “approximately half”. | Actioned. Line 473. |
| **DISCUSSION**  4) Please define abbreviations throughout, such as ‘10IU’. | Actioned. Please see line 495. |
| **REFERENCES**  1) Please ensure that journal name abbreviations match those found in the National Center for Biotechnology Information (NCBI) databases (<http://www.ncbi.nlm.nih.gov/nlmcatalog/journals>), and are appropriately formatted and capitalised. For example, in reference [10], should ”International Journal of Gynecology & Obstetrics“ be “Int J Gynaecol Obstet”. Please also see <https://journals.plos.org/plosmedicine/s/submission-guidelines#loc-references> for further details on reference formatting. | Actioned for manuscript and Supps. |
| **REFERENCES**  2) Where website addresses are cited, please specify the date of access (e.g. [accessed: 10/04/2024]). | Actioned. In the main manuscript this was relevant for one reference – *“The Campbell and Cochrane Economics Methods Group, Evidence for Policy and Practice Information and Coordinating Centre. CCEMG - EPPI-Centre Cost Converter 2010”* |
| **SUPPLEMENTARY MATERIAL**  In the published article, supporting information files are accessed only through a hyperlink attached to the captions. For this reason, you must list captions at the end of your manuscript file. You may include a caption within the supporting information file itself, as long as that caption is also provided in the manuscript file. Do not submit a separate caption file. | Actioned. Supps files are listed after the references, each with the name as the caption for easy identification. If there is other detail required for the caption, please advise us and we can update this. |
| **SUPPLEMENTARY MATERIAL**  a) When SI files are contained with a single file: Please label the file as ‘S1 Supporting Information’. Please apply alphabetical labelling to each table and figure contained within the S1 file. For example, ‘Fig A’ to ‘Fig Z’ and ‘Table A’ to ‘Table Z’. Plain text does not need to be labelled and can just be given a title as necessary. For example, ‘Statistical Analysis Plan’. Please cite tables/figures as ‘Fig A in S1 Supporting Information’ and/or ‘Table A in S1 Supporting Information’, for example. Please cite plain text as, ‘Statistical Analysis Plan in S1 Supporting Information’, for example. | Actioned. The Supp file was split into 12 appendices to facilitate this, as discussed with Dr Gaynor-Brook. |
| **SUPPLEMENTARY MATERIAL**  b) When SI files are uploaded as separate files: Please label tables as ‘S1 Table’ (so on) and figures as ‘S1 Fig’ (and so on). Any additional documents (protocols/analysis plans etc.) can be labelled as ‘S1 Protocol’, for example. Please cite items as exactly as labelled. | Actioned. |

| **Reviewers** | |
| --- | --- |
| **Comments/Requests** | **Responses** |
| **Reviewer #1**  Thank you for addressing comments. one more comment needed to be addressed mentioned in the following:  - The authors mentioned that they have extracted effectiveness data and compared it to an up-to-date effectiveness estimate from the corresponding WHO recommendation. They identified any effectiveness estimate that differed significantly (i.e., outside of 95% confidence intervals) from an effectiveness estimate found in the literature as having a risk of bias. Could you please clarify in which section you have provided the results for this? | The extraction and line by line comparison of effectiveness data is in S8 appendix.  We draw attention to the important results from this analysis throughout the Results section. For example, see lines 277, 289, 309 – 311. |
| **Reviewer #2:**  I am satisfied with the authors' responses and amendments made to the manuscript. I have nothing further to add. Thank you. | Thank you. |
| **Reviewer #3:**  The authors have addressed my comments, added to table by region and strengthened the Discussion, I have no further comment. | Thank you. |

| **General Editorial Requests** | |
| --- | --- |
| **Comments/Requests** | **Responses** |
| 1) We ask every co-author listed on the manuscript to fill in a contributing author statement. If any of the co-authors have not filled in the statement, we will remind them to do so when the paper is revised. If all statements are not completed in a timely fashion this could hold up the re-review process. Should there be a problem getting one of your co-authors to fill in a statement we will be in contact. YOU MUST NOT ADD OR REMOVE AUTHORS UNLESS YOU HAVE ALERTED THE EDITOR HANDLING THE MANUSCRIPT TO THE CHANGE AND THEY SPECIFICALLY HAVE AGREED TO IT. | Co-authors have not yet received the email from the journal office requesting their confirmation of authorship. |
| 2) Please ensure that the paper adheres to the PLOS Data Availability Policy (see <http://journals.plos.org/plosmedicine/s/data-availability>), which requires that all data underlying the study's findings be provided in a repository or as Supporting Information. For data residing with a third party, authors are required to provide instructions with contact information for obtaining the data. PLOS journals do not allow statements supported by "data not shown" or "unpublished results." For such statements, authors must provide supporting data or cite public sources that include it. | All relevant data is contained within the manuscript and supplementary files. |
| 3) Please review your reference list to ensure that it is complete and correct. If you have cited papers that have been retracted, please include the rationale for doing so in the manuscript text, or remove these references and replace them with relevant current references. Any changes to the reference list should be mentioned in the rebuttal letter that accompanies your revised manuscript. | Actioned.  One reference was changed from a preprint to the final publication – Williams et al., 2024. |
